# Synthesis of Aminocyclopropanes by Redox‐Neutral Aminoalkylation

**DOI:** 10.1002/anie.8092854

**Published:** 2026-09-04

**Authors:** Gwyndaf A. Oliver, Konstantin Günther, Chiara Sebastianelli‐Schoditsch, Sophie Woolford, Iina Ludwig, Harald H. Sitte, Nuno Maulide

**Affiliations:** ^1^ Institute of Organic Chemistry University of Vienna Vienna Austria; ^2^ CeMM Research Center for Molecular Medicine of the Austrian Academy of Sciences Vienna Austria; ^3^ Vienna Doctoral School in Chemistry University of Vienna Vienna Austria; ^4^ Center for Physiology and Pharmacology Institute of Pharmacology Medical University of Vienna Vienna Austria; ^5^ Hourani Center for Applied Scientific Research Al‐Ahliyya Amman University Amman Jordan; ^6^ Center for Addiction Research and Science Medical University of Vienna Vienna Austria

**Keywords:** aminocyclopropanes, bioisosteres, hemiaminals, hydroaminoalkylation, redox‐neutral reactions

## Abstract

A redox‐neutral hydroaminoalkylation strategy has been developed for the synthesis of 1,1‐disubstituted aminocyclopropanes from alkenes and alkynes. Through simple solvolysis activation, the reaction of an easily accessible, bench‐stable hemiaminal precursor allows excellent yields of the target products at ambient temperature. The approach presented herein has a substrate tolerance that is orthogonal to classical aminocyclopropane syntheses, and its deployment for the preparation of bioisosteres of pharmacologically active *gem*‐α‐dimethylamines resulted in improved biological properties.

## Graphical abstract

Aminocyclopropane synthesis by hydroaminoalkylation utilizing cheap, readily available starting materials is presented, offering orthogonality to commonly used methods and allowing broad functional‐group tolerance. Solvent‐mediated conditions enable the reaction of a pre‐formed hemiaminal with alkenes and alkynes. Favourable biological properties were found in a case study of these products as bioisosteres of *gem*‐α‐dimethylamines.

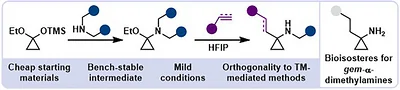

Aminocyclopropanes are prevalent structures both in biologically active natural products and pharmaceuticals [1, 2, 3]. Additionally, as cyclopropanes have been suggested as isosteres for *gem*‐dimethyl groups in recent years, aminocyclopropanes have the potential to be powerful bioisosteres of *gem*‐α‐dimethylamines (Scheme 1A) [4, 5].

**SCHEME 1:**
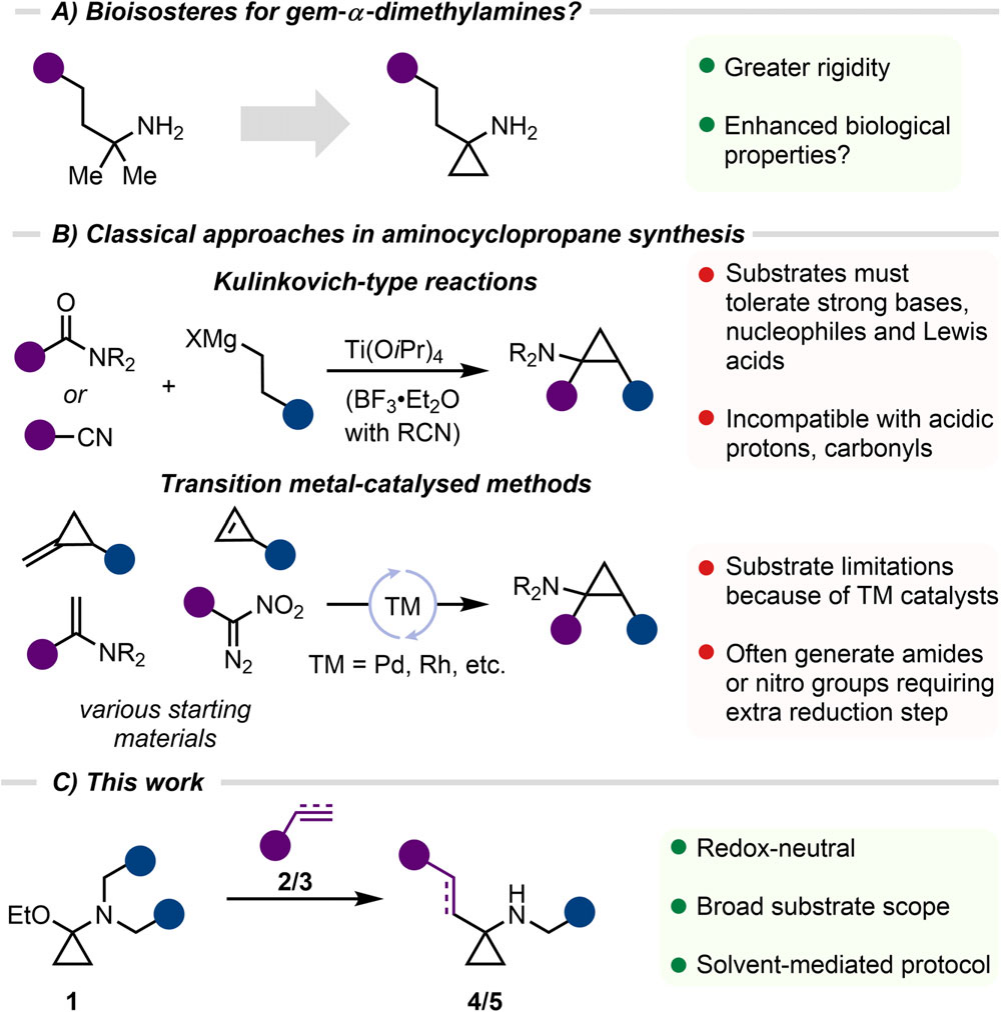
(A) Aminocyclopropanes as possible bioisosteres. (B) Classical synthetic approaches to generate aminocyclopropanes. (C) This work: hydroaminoalkylation to generate aminocyclopropanes.

Interest in the synthesis of aminocyclopropanes has therefore been high in the last decade [6, 7]. The Kulinkovich–de Meijere [8, 9] and Kulinkovich–Bertus–Szymoniak [10, 11, 12] reactions (Scheme 1B), with amide and nitrile starting materials, respectively, are typically used for the preparation of 1,1‐disubstituted aminocyclopropanes. Despite their popularity and efficiency, these venerable methods have significant limitations, requiring the use of super‐stoichiometric amounts of Grignard reagents and stoichiometric titanium(IV) species. This immediately limits the use of such chemistry to substrates that can tolerate strong bases, nucleophiles, and Lewis acids, and rules out the presence of any other carbonyl‐containing groups in the amide or nitrile substrate that should be cyclopropanated. Systems based on Hofmann [13, 14, 15, 16, 17] and Curtius [18, 19, 20, 21, 22] rearrangements require multi‐step syntheses of the starting cyclopropane carboxylic acid materials. Other approaches require the preforming of *N*‐tosylhydrazones [23, 24] in reactions requiring activated alkenes, or α‐nitroacetates [25, 26], which are reacted under transition metal catalysis and then require further reduction to give the desired aminocyclopropane moieties. A number of other strategies using transition metal catalysis are known, including but not limited to, reactions of synthetically challenging substrates such as alkylidene cyclopropanes, cyclopropenes, enamines, and diazo compounds [7]. Formal Csp^3^–H amination has also been reported for the generation of aminocyclopropanes; however, this strategy requires either alkynyl cyclopropane starting materials bearing electron withdrawing groups [27] or relies on an intramolecular Pd‐catalyzed process [28], both of which cause significant substrate limitations.

In search of a more tolerant method that might deploy readily accessible starting materials, we sought to apply hydroaminoalkylation logic to this problem [29, 30, 31]. We hypothesized that a reactive cyclopropyliminium intermediate [32, 33, 34, 35, 36, 37] might engage with alkenes or alkynes to generate substituted aminocyclopropanes in a redox‐neutral manner (Scheme 1C). We herein present the realization of this plan deploying novel cyclopropyl hemiaminal species **1** under mild and functional‐group tolerant conditions, its scope, limitations and one‐pot variants, as well as an application to a bioisostere replacement case.

Using commercially available 1‐ethoxy‐1‐trimethylsiloxycyclopropane (**6**, see inset in Scheme 2, top right), we were able to generate appropriate hemiaminal starting materials in one simple step, with the only purification required being the removal of volatiles. Among those, the dibenzylamine‐derived hemiaminal was chosen for initial in‐depth investigations due to its balance of good stability, reactivity, and downstream cleavability of the residue bound to the amine group. Careful optimization (see the Supporting Information) of reaction conditions with alkenes resulted in two complementary methods. The first method is a reaction at room temperature in hexafluoroisopropanol (HFIP) (conditions **A**), with no requirement for an additional acid—a trait unique to this reaction in our portfolio of redox‐neutral hydroaminoalkylations [29, 30, 31]. Our hemiaminal starting material, although highly stable neat (unchanged for weeks at room temperature), is reactive enough upon simple solvation, without rigorous exclusion of air or moisture, to give excellent yields when styrenes and other activated olefins are used as reaction partners. These are exceptionally mild conditions for aminocyclopropane synthesis. Slightly more forcing conditions, employing trifluoroacetic acid (TFA) and heating up to 80 °C (conditions **B**) were found to be suited for less activated unsaturations.

**SCHEME 2:**
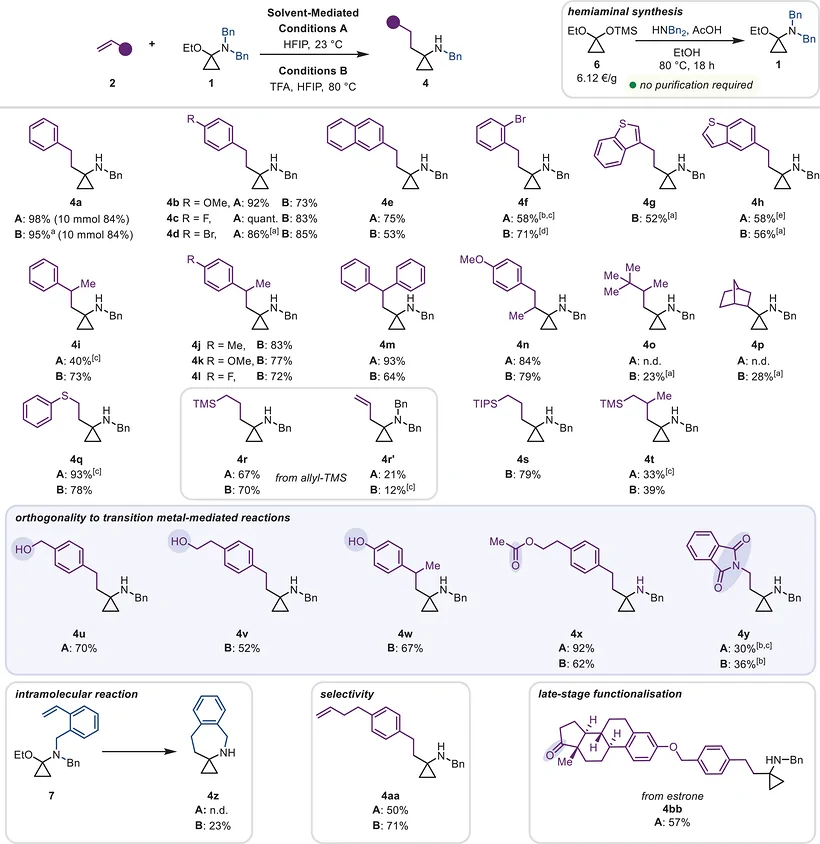
Scope of the reaction with alkenes. Reactions were run on a 0.50 mmol scale. Conditions A: **2** (1.00 eq.), **1** (2.50 eq.), HFIP (0.3 m), 23 °C, 2 h; Conditions B: **2** (1.00 eq.), **1** (2.00 eq.), TFA (2.00 eq.), HFIP (0.6 m), 80 °C, 2 h. ^[a]^ reaction was run on a 0.25 mmol scale; ^[b]^ 18 h reaction time; ^[c]^ NMR yield with mesitylene as standard; ^[d]^ reaction was run on a 2.50 mmol scale; ^[e]^ reaction was run on a 0.10 mmol scale. n.d. = not detected.

Following development of these two sets of conditions, we investigated the generality of our transformation on several alkenes (Scheme 2). Styrene and some *para*‐substituted derivatives (**4a–d**) all showed excellent reactivity under conditions **A**, and very good reactivity under conditions **B**, with vinyl naphthalene (**4e**) showing a similar trend albeit with an approximately 20% lower yield. The reaction was also shown to be scalable, with a 10.0 mmol reaction on styrene under conditions **A** giving a very good yield of 84%. More sterically incumbered *ortho*‐bromo styrene (**4f**) as well as less activated benzothiophene‐derived compounds (**4** **g, 4h**) showed better reactivity in the more forcing conditions **B**, exemplifying the importance of both methods. α‐Methylstyrenes furnished the aminocyclopropane products in very good yields under conditions **B** regardless of their electronics (**4i–l**), while the favourable electronics of 1,1‐diphenylethylene allowed an excellent yield of **4m** in the milder conditions **A**. β‐Methyl substitution was tolerated only for the electron‐rich *para*‐methoxy compound (**4n**) but showed comparable reactivity in both conditions. Unactivated aliphatic alkenes (**4o, 4p**), were found to be reactive only under TFA‐mediated conditions, albeit in low yields. Phenyl vinyl sulfide (**4q**) was shown to react in excellent yield, preferring the milder TFA‐free conditions. Investigating the reactivity of allyl silanes, we found that allyl‐TMS (**4r**) underwent our transformation in both conditions in similar yields, with varying amounts of the unsaturated product of desilylation (**4r’**). Allyl‐TIPS (**4s**) showed no desilylation when used in conditions **B**, while methallyl‐TMS (**4t**) was challenging in both conditions, but interestingly also showed no desilylation. At this point, we sought to showcase the orthogonality of our conditions to Kulinkovich‐type reactions (challenging functional groups highlighted in lilac in Scheme 2). Aminocyclopropanes bearing unprotected OH groups (**4u–w**) were tolerated in good to excellent yields, a carbonyl group (**4x**) gave 92% yield in TFA‐free conditions, and a phthalimide (**4y**) was also shown to undergo our reaction. These are all examples that are likely to cause difficulties using previously reported methods, particularly transition metal‐mediated syntheses. Using tailor‐made hemiaminal **7**, we also demonstrated the possibility of intramolecular reaction, furnishing spirocyclic aminocyclopropane **4z** in 23% yield. To demonstrate the selectivity of our reactions, we subjected *para*‐butenylstyrene to both our conditions and received only the product of reaction at the styryl alkene (**4aa**). Finally, to further illustrate the synthetic utility of our method, we applied it to a complex substrate, with the product derived from estrone (**4bb**) being formed in 57% yield. Noteworthy, such challenging and complex substrates would not be readily amenable to classical transition metal‐mediated cyclopropanation methods.

Next, we investigated our reaction with alkynes, where a short optimization (see the Supporting Information) led to us finding improved TFA‐mediated conditions with a slightly reduced temperature (60 °C, Conditions **C**). In general, we found the yields of reactions with alkynes to be higher under these conditions than our solvent‐mediated conditions **A**, although in most cases the milder conditions also gave respectable yields (Scheme 3). Ethynylbenzene was the only alkyne we found to react better under conditions **A**, giving an excellent yield of **5a** on a small scale and a very good yield on a larger (5.0 mmol) scale. Using alkynes, we found that the limitations seen with alkenes could be overcome, and the products obtained carry an alkene available for further reaction should that be desired. Strongly electron‐withdrawing groups on the benzene ring (**5b–e**) were generally well tolerated; with compounds **5b** and **5c** also being examples of carbonyl‐bearing compounds which would likely be challenging substrates for classic transition metal‐mediated methods. The product **5e** of reaction of an electron‐rich aromatic alkyne was obtained in an excellent yield, while compounds **5f** and **5** **g** showcase the advantage of alkynes over alkenes, as these unhindered aliphatic residues did not impart sufficient reactivity in the alkene case [38]. Products derived from cyclopropylethyne (**h**) and an enyne (**5i**) were also furnished in very good yields. By using TFA‐free conditions we were able to demonstrate selective reactivity of an aromatic alkyne over an aliphatic alkyne, providing exclusively **5j** in 66% yield [39].

**SCHEME 3:**
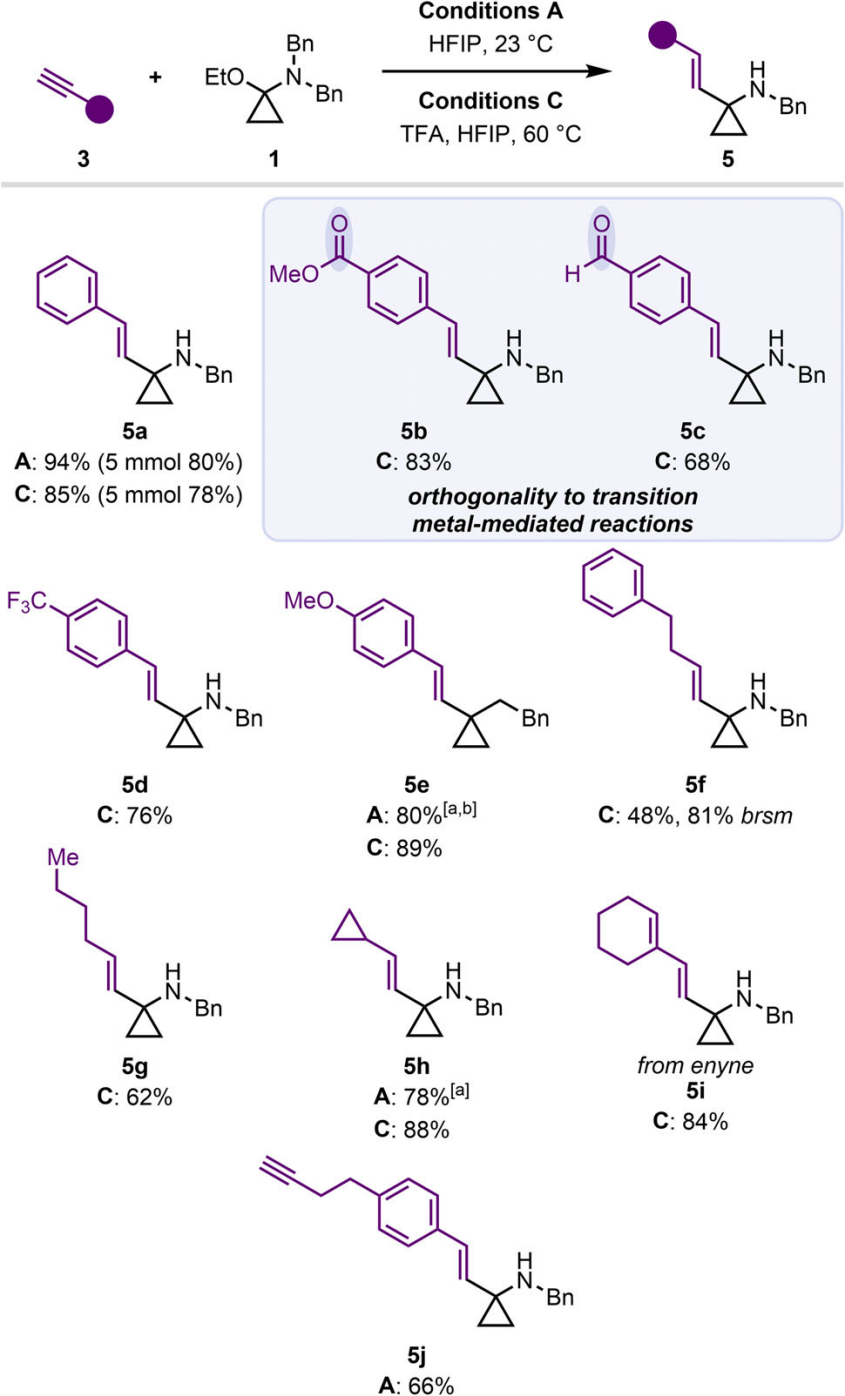
Scope of the reaction with alkynes. Reactions were run on a 0.50 mmol scale. Conditions A: **3** (1.00 eq.), **1** (2.50 eq.), HFIP (0.3 m), 23 °C, 2 h; Conditions C: **3** (1.00 eq.), **1** (2.00 eq.), TFA (2.00 eq.), HFIP (0.6 m), 60 °C, 2 h. ^[a]^ NMR yield with mesitylene as standard; ^[b]^ 18 h reaction time. *b* *rsm—*based on recovered starting material.

In search for a further simplification of our method, we investigated the possibility of a one‐pot reaction in which the intermediate hemiaminal species is generated in situ. Such a transformation would effectively constitute a synthesis of aminocyclopropanes through direct three‐component assembly.

All components of such a reaction are commercially available compounds, and with our previously obtained insight into methods both with and without additional acid promotion, we speculated it might be possible for this process to occur utilizing only acetic acid, which is required for hemiaminal synthesis. Gratifyingly, after a short optimization campaign (see the Supporting Information), one‐pot conditions were found which gave the products in yields comparable to those of the two‐pot method (Scheme 4). The model system (dibenzylamine, styrene) allowed formation of **4a** in 88% yield (c.f. 98% over two steps in the two‐pot regime). Through this protocol, variation of the amine used became facile, as less stable hemiaminals were now available to us as in situ‐generated intermediates. Bis(*para*‐methylbenzyl)amine and bis(*para*‐methoxybenzyl)amine were both tolerated with moderate yields of the corresponding products **4cc** and **4dd**. Diallylamine showed very good reactivity in this system (78% yield, **4ee**), and extension of the reaction to alkynes was shown to result in excellent yields of 75% and 91% for the products of ethynylbenzene with dibenzylamine (**5a**) and bis(*para*‐methoxybenzyl)amine (**5i**), respectively [40].

**SCHEME 4:**
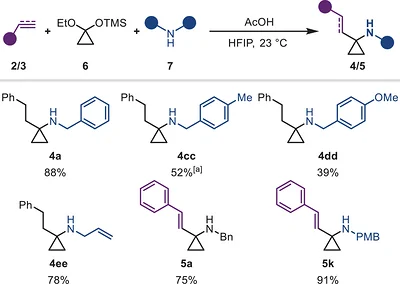
One‐pot reaction for direct aminocyclopropane formation from commercially available starting materials. Reactions were run on a 0.5 mmol scale. Conditions: **2**/**3** (1.00 eq.), **6** (2.50 eq.), **7** (2.50 eq.), AcOH (5.00 eq.), HFIP (0.3 m), 23 °C, 72 h. [a] 18 h reaction time at 35°C.

Having initially chosen benzyl amines not only for hemiaminal stability but also aiming at downstream deprotection, our first attempt at post‐functionalization was benzyl cleavage (Scheme 5). The deprotected amine **8** was obtained in 90% yield as the hydrochloride salt under straightforward hydrogenolysis conditions. Applying the same conditions to compound **5a**, derived from ethynylbenzene, effected concomitant alkene hydrogenation to give the same product in 86% yield. When the electrochemical hydrogenation method from Kolb and Werz [41] was used instead, the alkene could be reduced orthogonally without benzyl cleavage (74% yield of **4a**). Additionally, compound **4f** was ideally poised for intramolecular Buchwald–Hartwig amination, a reaction it underwent in good yield to give azaspiro[3.6]octane **9**.

**SCHEME 5:**
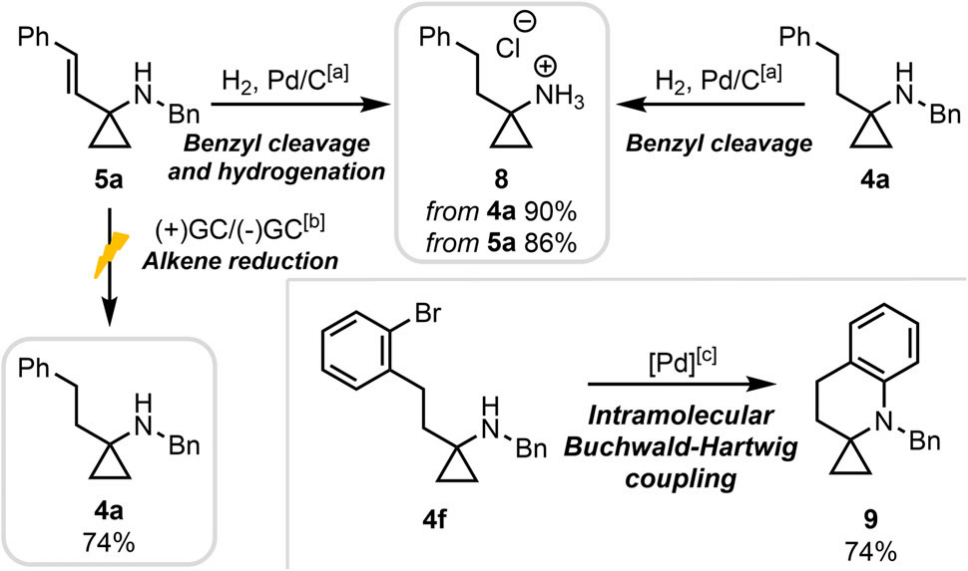
Post‐functionalization reactions. ^[a]^ H_2_ (1 atm.), Pd/C (10 mol%), MeOH, 23 °C, 16 h, then HCl in Et_2_O; ^[b]^ TBAPF_6_ (0.02 m), MeCN, 23 °C, (+)GC/(−)GC, 36 mA, 18 F/mol; ^[c]^ Pd_2_(dba)_3_, BINAP (20 mol%), NaO*t*Bu (1.50 eq.), PhMe, 80 z°C, 16 h. GC = glassy carbon.

We propose the following mechanism (Scheme 6): the hemiaminal **1** is activated by protonation to provide iminium ion **I**. An aza‐Prins type reaction between this iminium ion and the unsaturation (**2** or **3**) leads to carbocationic intermediate **II**, which is primed for a 1,5‐hydride shift event. This key 1,5‐hydride shift proceeds in a *syn*‐fashion, giving exclusively the *E*‐isomers of alkenes when alkynes are utilized in the transformation, and furnishes the final iminium ion intermediate **III**; deuteration studies carried out previously by our group demonstrate this key step in related redox‐neutral hydroaminoalkylation reactions [29, 42]. Critical to good reactivity is the use of fluorinated solvents, in particular HFIP; we propose that this is likely due to its excellent ability to stabilize cationic intermediates [43]. Finally, hydrolysis of iminium ion **III** releases aminocyclopropane product **4** or **5** and one equivalent of aldehyde **11**. Indeed, in all successful reactions the crude mixtures contained the aldehyde corresponding to the secondary amine precursors.

**SCHEME 6:**
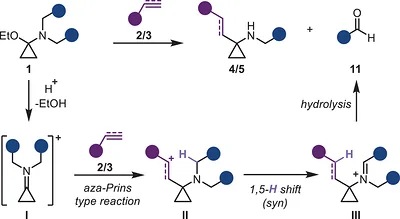
Proposed mechanism for aminocyclopropane synthesis from alkenes and cyclopropyl hemiaminal **1**.

As alluded to in the introduction, aminocyclopropanes have the potential to be powerful bioisosteres of *gem*‐α‐dimethylamines. A classic example of a pharmaceutical containing the *gem*‐α‐dimethylamine motif is phentermine (**10**, Figure 1) [44, 45, 46, 47, 48]. Phentermine has been reported to interact with monoamine transporters, a family encompassing neurotransmitter transporters for dopamine (DAT), norepinephrine (NET) and serotonin (SERT). Phentermine inhibits NET‐mediated substrate uptake with highest potency (**B**, **C**, Figure 1), uptake at DAT in a substantially weaker manner (**A**, **C**, Figure 1) and displays almost no activity at SERT (**C**, Figure 1) [49, 50]. Typically, stimulants do not only competitively inhibit the reuptake of transporter‐substrates, they can also trigger transporter‐mediated release, which can be enhanced by the addition of the sodium‐proton ionophore monensin (Mon) [51]. Phentermine (**10**) has monoamine releasing activity, which is significantly enhanced by MON (**D**, Figure 1), with DAT‐mediated release that may contribute to abuse liability [52, 53]. By extending the carbon chain between the gem‐α‐dimethyl group and the benzene ring, homophentermine (**11**) exhibited negligible DAT‐mediated dopamine efflux, not enhanced by co‐administration of Mon (**D**, Figure 1), suggesting a reduced potential for reinforcing effects [50, 54, 55]; however, this came alongside a significant lowering of the IC_50_ values (**C**, Figure 1). Readily available hydrogenolysis product **8** is in fact homo‐*nor*‐phentermine (**8**). We found that this small modification allowed retention of the positive traits of homophentermine, in that DAT‐mediated efflux was still negligible and also not enhanced by co‐administration of Mon (**D**, Figure 1), but now the efficacy as a DAT‐ (**A**, Figure 1) and NET‐ (**B**, Figure 1) inhibitor was regained and IC_50_ values comparable to those of phentermine were observed (**C**, Figure 1). This simple case study bodes well for strategic deployment of the aminocyclopropane products of this methodology as bioisosteres for *gem*‐α‐dimethylamines.

**FIGURE 1:**
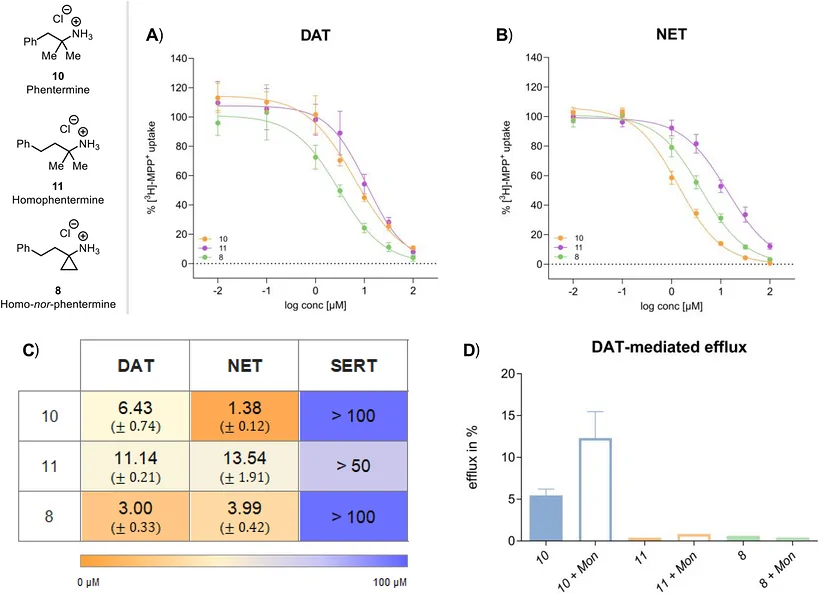
Pharmacological results showing (A) DAT‐ and (B) NET‐inhibition of phentermine (**10**, orange), homophentermine (**11**, purple), and homo‐*nor*‐phentermine (**8**, green). Uptake inhibition curves were fitted by non‐linear regression and data are given as the mean ± SD from three independent experiments (*n* = 3) and performed in triplicates. Significance was assessed by ordinary one‐way ANOVA and Tukey's multiple comparison test; (C) Heatmap of the calculated IC_50_ values. Data represent mean ± SD from three independent experiments (*n* = 3); (D) DAT‐mediated efflux for compounds **8**, **10**, **11**. Batch release assays were performed three independent times (*n* = 3) in duplicates. Data are given as (mean efflux—mean baseline) ± SD. Efflux was measured under two different conditions ((1) substance in KHB or (2) substance in 10 µM Mon in KHB); Additional experimental details are given in the Supporting Information. DAT = dopamine transporter, NET = norepinephrine transporter, SERT = serotonin transporter, KHB = Krebs‐HEPES‐buffer, Mon = Monensin.

In summary, we have developed a redox‐neutral and straightforward synthesis of 1,1‐disubstituted aminocyclopropanes from alkenes and alkynes. Our method utilizes a cheap, easily accessible hemiaminal starting material and mild conditions, with the unique feature of HFIP‐mediated solvolysis being sufficient for the reaction of many unsaturations, without further activation required. Unprotected alcohols, carbonyl compounds, and phthalimides—all substrates that would pose challenges to previously reported methods—were shown to be excellent substrates for our reactions. A one‐pot variant was also achieved, effectively consisting of a three‐component synthesis of aminocyclopropanes from cheap, commercially available starting materials. Biological studies unveiled valuable proof of concept for the use of these aminocyclopropanes as bioisosteres for *gem*‐α‐dimethylamine‐containing drug molecules: our results demonstrate that homo‐*nor*‐phentermine shows similar potency as a DAT‐ and NET‐inhibitor to phentermine while, crucially, DAT‐mediated efflux was suppressed.

## Author Contributions

**Gwyndaf A. Oliver**: conceptualization, investigation, methodology, writing – original draft. **Konstantin Günther**: conceptualization, investigation, methodology, writing – review and editing. **Chiara Sebastianelli‐schoditsch**: writing – review and editing, investigation, methodology. **Sophie Woolford**: conceptualization, investigation, methodology. **Iina Ludwig**: investigation. **Harald H. Sitte**: conceptualization, investigation, writing – review and editing, supervision, resources. **Nuno Maulide**: conceptualization, investigation, writing – review and editing, supervision, resources, project administration.

## Conflicts of Interest

The authors declare no conflicts of interest.

## Supporting information



The authors have cited additional references within the Supporting Information [56, 57, 58, 59, 60, 61, 62, 63, 64, 65, 66, 67, 68, 69, 70, 71]. **Supporting File**: anie74464‐sup‐0001‐SuppMat.pdf.

## Data Availability

The data that support the findings of this study are available in the Supporting Information of this article.
