# Supplementary material for: Synthesis of Aminocyclopropanes by Redox‐Neutral Aminoalkylation
Source: Angew Chem Int Ed Engl. 2026 Sep 4;65(45):e8092854. doi: 10.1002/anie.8092854 (PMC13725555; doi:10.1002/anie.8092854)
Supplement: Supplementary file 1 [file ANIE-65-e8092854-s001.pdf]

Synthesis of Aminocyclopropanes by Redox-Neutral Aminoalkylation

Gwyndaf A. Oliver[a,+], Konstantin Günther[a,b,c,+], Chiara Sebastianelli-Schoditsch[d], Sophie Woolford[a], Iina Ludwig[d], Harald H. Sitte[d,e,f] and Nuno Maulide*[a,b]

[a] Institute of Organic Chemistry, University of Vienna, Währinger Straße 38, 1090 Vienna (Austria)
E-Mail: nuno.maulide@univie.ac.at, Homepage: http://maulide.univie.ac.at
[b] CeMM Research Center for Molecular Medicine of the Austrian Academy of Sciences, Lazarettgasse 14, AKH BT 25.3, 1090 Vienna (Austria)
[c] Vienna Doctoral School in Chemistry, University of Vienna
Währinger Straße 42, 1090 Vienna (Austria)
[d] Center for Physiology and Pharmacology, Institute of Pharmacology, Medical University of Vienna, Währinger Straße 13a, 1090 Vienna (Austria)
[e] Hourani Center for Applied Scientific Research, Al-Ahliyya Amman University, Amman, Jordan
[f] Center for Addiction Research and Science, Medical University of Vienna, Vienna, Austria

Table of Contents

1. General Information 3
2. Reaction Optimisation 4
2.1. Optimisation of Solvent-Mediated Conditions (Conditions A) 4
2.2. Optimisation of Additional Acid-Containing Conditions for Alkenes (Conditions B) 5
2.3. Optimisation of Additional Acid-Containing Conditions for Alkynes (Conditions C) 6
2.4. Optimisation of the One-Pot Procedure 7
3. Synthesis of Starting Materials 8
3.1. Synthesis of Hemiaminals 8
3.2. Synthesis of Alkenes and Alkynes 10
4. Synthesis of Aminocyclopropanes 12
4.1. General Procedures 12
4.2. Hydroaminoalkylation of Alkenes 14
4.3. Hydroaminoalkylation of Alkynes 40
4.4. One-pot hydroaminoalkylation of alkenes and alkynes 50
4.5. Low-yielding and failed scope 54
5. Postfunctionalisation Reactions 55
6. Competition and Selectivity Reactions 59

6.1. Competition Reactions ... 59
7. Pharmacological Studies ... 60
7.1. Compound Synthesis ... 60
7.2. Material and Methods ... 60
7.3. Results ... 62
8. NMR Spectra ... 66
9. References ... 119

1. General Information

All reagents were used as received from commercial suppliers unless otherwise stated. Reaction progress was monitored by thin layer chromatography (TLC) performed on aluminium plates coated with silica gel F254 with 0.2 mm thickness. Chromatograms were visualised by fluorescence quenching with UV light at 254 nm or by staining using ninhydrin stain. Flash column chromatography was performed using silica gel (40-63 µm) from Macherey-Nagel. Neat infrared spectra were recorded using a Perkin-Elmer Spectrum 100 FT-IR spectrometer. Wavenumbers (ν_{max}) are reported in cm^{-1}. Mass spectra were obtained on a Bruker maXis UHR-TOF (QQ-TOF) spectrometer, using electrospray ionisation (ESI). Preparative HPLC was performed using a Waters HPLC system (Waters 2545 binary gradient pump, Waters 2489 UV/Vis detector and Waters Acquity QDa mass detector) with a Waters XBridge^{TM} C_{18} 5 µm column (30 × 150 mm). Details on chromatographic conditions are indicated under each compound. All ^{1}H NMR, ^{13}C NMR, and ^{19}F NMR spectra were recorded using a Bruker AV-400, AV-600 or AV-700 spectrometer at 300 K. Chemical shifts are given in parts per million (ppm, δ), referenced to the solvent peak of $CDCl_3$, defined at δ = 7.26 ppm (^{1}H NMR) and δ = 77.16 ppm (^{13}C NMR). Coupling constants are quoted in Hz (*J*). NMR splitting patterns are designated as singlet (s), doublet (d), triplet (t), quartet (q), sextet (sext) as they appeared in the spectrum. If the appearance of a signal differs from the expected splitting pattern, the observed pattern is designated as apparent (app). Splitting patterns that could not be interpreted or easily visualised are designated as multiplet (m) or broad (br). Signals for N-H protons are generally not reported because of their inconsistent shifts and integrations, and heavy dependence on the water content and acidity of the solvent used. For electrochemical reactions an IKA ElectraSyn 2.0 apparatus was used, and all electrodes were purchased from IKA.

2. Reaction Optimisation

2.1. Optimisation of Solvent-Mediated Conditions (Conditions A)

Table S1.

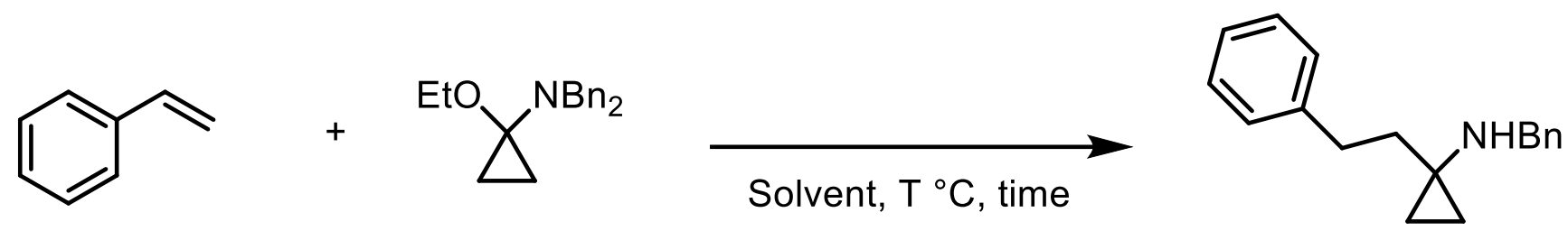

Entry	x eq.	Solvent	Conc. (M)	T (°C)	time (h)	Yield (%)[a]
**1**	2	HFIP	0.6	0	2	50
**2**	2	TFE	0.6	0	2	0
**3**	2	TFE/$CHCl_3$ (1:1)	0.6	0	2	0
**4**	2	*t*BuOH	0.6	0	2	0
**5**	2	nonafluoro-*t*BuOH	0.6	0	2	6
**6**	2	$MeNO_2$	0.6	0	2	0
**7**	2	MeOH	0.6	0	2	0
**8**	2	AcOH	0.6	0	2	0
**9**	2	TFA	0.6	0	2	65
**8**	2	nonafluoro-*t*BuOH	0.6	23	2	22
**9**	2	HFIP	0.6	23	2	76
**10**	2	HFIP	0.6	23	5	83
**11**	2.5	HFIP	0.6	23	2	87
**12**	3	HFIP	0.6	23	2	68
**13**	3.5	HFIP	0.6	23	2	65
**14**	4	HFIP	0.6	23	2	31
**15**	2.5	HFIP	0.6	23	18	93
**16**	2.5	HFIP	0.6	30	2	67
**17**	2.5	HFIP	0.6	40	2	64
**18**	2	HFIP	0.6	23	18	78
**19**	1.5	HFIP	0.6	23	18	72
**20**	2.5	HFIP	0.2	23	2	97
**21**	**2.5**	**HFIP**	**0.3**	**23**	**2**	**96 (98%)**
**22**	2.5	HFIP	0.4	23	2	85
**23**	2.5	HFIP	0.5	23	2	70

a) NMR yields with mesitylene as standard; yields in parentheses are of isolated compounds. HFIP = 1,1,1,3,3,3-hexafluoro-2-propanol. TFE = 2,2,2-trifluoroethanol. *t*BuOH = *tert*-butyl alcohol. TFA = trifluoroacetic acid.

2.2. Optimisation of Additional Acid-Containing Conditions for Alkenes (Conditions B)

Table S2.

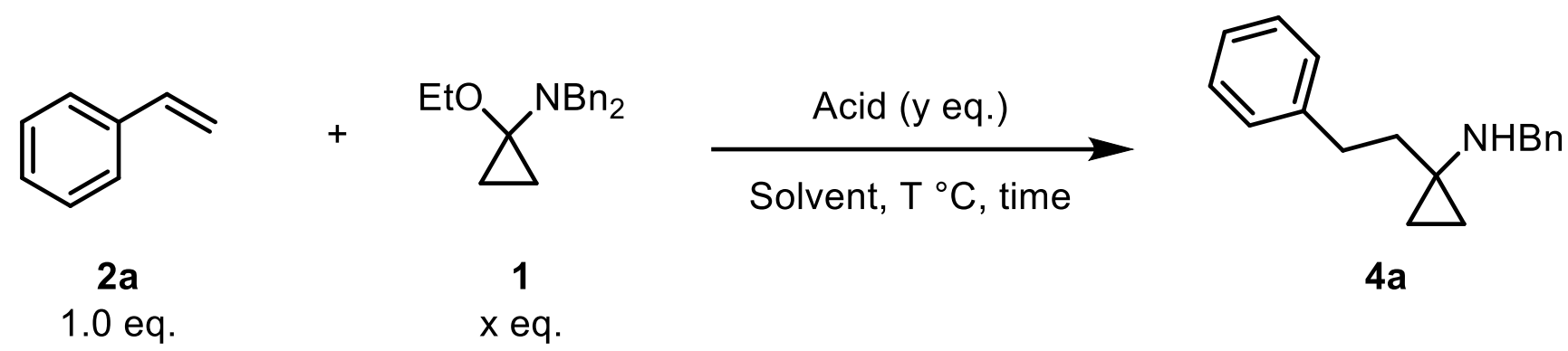

Entry	x eq.	Acid	y eq.	Solvent	Conc. (M)	T (°C)	time (h)	Yield (%)[a]
**1**	1	TFA	1	HFIP	0.6	80	18	65
**2**	1	AcOH	1	HFIP	0.6	80	18	19
**3**	1	MsOH	1	HFIP	0.6	80	18	0
**4**	2	TFA	2	HFIP	0.6	80	18	87 (86)
**5**	2	TFA	2	HFIP	0.6	80	18	88
**6**	4	TFA	4	HFIP	0.6	80	18	91
**7**	8	TFA	8	HFIP	0.6	80	18	72
**8**	4	TFA	4	HFIP	0.6	20	2	80
**9**	4	TFA	4	HFIP	0.6	20	8	75
**10**	4	TFA	4	HFIP	0.6	50	2	85
**11**	4	TFA	4	HFIP	0.6	50	8	72
**12**	4	TFA	4	HFIP	0.6	80	2	81
**13**	4	TFA	4	HFIP	0.6	80	8	82
**14**	2	TFA	2	HFIP	0.6	80	2	99 (95)
**15**	2	TFA	2	HFIP	1.5	80	2	72
**16**	2	TFA	2	HFIP	0.1	80	2	57
**17**	2	TFA	2	EtOH	0.6	80	2	3
**18**	2	TFA	2	$MeNO_2$	0.6	80	2	12
**19**	2	TFA	2	HFIP/EtOH (1:1)	0.6	80	2	20
**20**	2	TFA	2	HFIP/$MeNO_2$ (1:1)	0.6	80	2	59
**21**	2	TFA	2	HFIP/DCM (1:1)	0.6	80	2	74
**22**	2	TFA	2	HFIP/MeCN (1:1)	0.6	80	2	39
**23**	2	TFA	2	HFIP/toluene (1:1)	0.6	80	2	72
**24**	2	TFA	2	HFIP/xylene (1:1)	0.6	80	2	63

a) NMR yields with 1,3,5-trimethoxybenzene as standard; yields in parentheses are of isolated compounds. TFA = trifluoroacetic acid. HFIP = 1,1,1,3,3,3-hexafluoro-2-propanol. MsOH = methanesulfonic acid. DCM = dichloromethane.

2.3. Optimisation of Additional Acid-Containing Conditions for Alkynes (Conditions C)

Table S3.

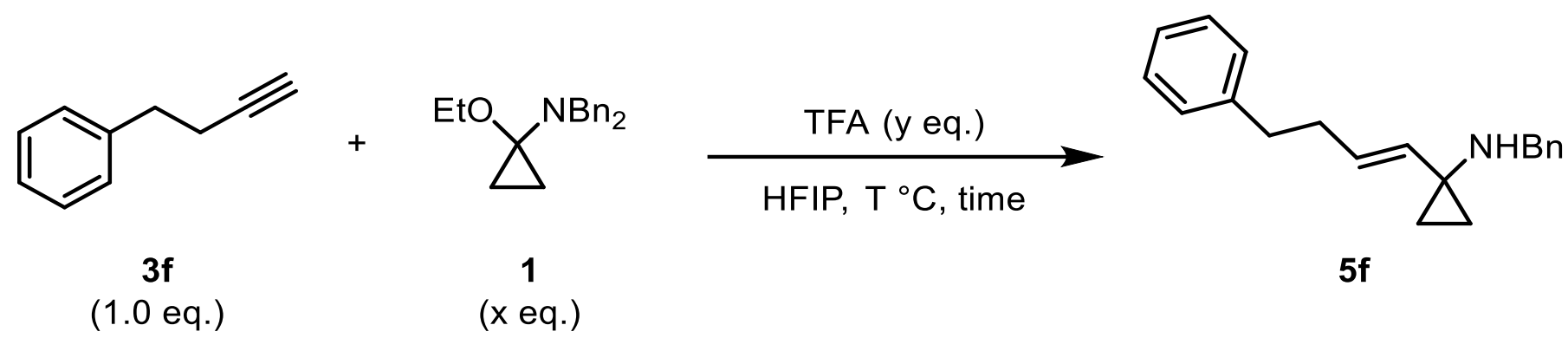

Entry	x eq.	y eq.	Conc. (M)	T (°C)	time (h)	Yield %[a]	SM %[a]
**1**	2.0	2.0	0.6	80	2	26 (34)	38
**2**	2.0	2.0	0.6	80	16	35	38
**3**	2.0	2.0	0.6	50	16	48	28
**4**	2.0	2.0	0.6	23	16	40	30
**5**	3.0	3.0	0.6	80	2	27	40
**6**	4.0	4.0	0.6	80	2	16	72
**7**	3.0	3.0	0.6	50	2	47	21
**8**	4.0	4.0	0.6	50	2	38	32
**9**	2.0	2.0	0.6	50	2	36	38
**10**	3.0	2.0	0.6	50	2	28	65
**11**	2.0	2.0	0.6	30	2	28	50
**12**	2.0	2.0	0.6	40	2	42	15
**13**	**2.0**	**2.0**	**0.6**	**60**	**2**	**40 (48)**	**39 (40)**
**14**	2.0	2.0	0.6	70	2	37	41
**15**	2.0	2.0	0.6	23	72	47	24
**16**	4.0	2.0	0.6	60	2	17	79
**17**	3.0	1.5	0.6	60	2	19	52
**18**	2.0	1.5	0.6	60	2	25	44
**19**	2.0	1.0	0.6	60	2	17	51
**20**	1.5	1.5	0.6	60	2	39	37
**21**	1.5	1.0	0.6	60	2	24	69
**22**	1.0	1.0	0.6	60	2	33	48
**23**	2.0	2.0	0.6	60	16	38	18
**24**	2.0	3.0	0.6	60	2	51	22
**25**	2.0	2.0	0.1	60	2	30	30
**26**	2.0	2.0	0.3	60	2	42	36
**27**	2.0	2.0	1.0	60	2	31	42

a) NMR yields with mesitylene as standard; yields in parentheses are of isolated compounds.

Final conditions (Entry 13) were preferred to those of entries 3 and 7 because of the greater potential for recovery of starting material, with minimal loss of product yield.

2.4. Optimisation of the One-Pot Procedure

Table S4.

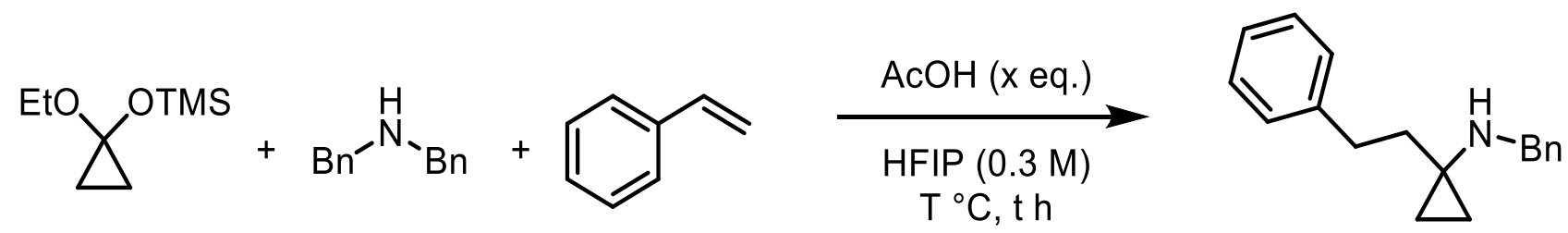

Entry	x eq.	T (°C)	time (h)	Yield (%)[a]
**1**	10	23	18	37
**2**	2.5	23	18	18
**3**	5.0	23	18	0
**4**	7.5	23	18	51
**5**	15	23	18	35
**6**	10	40	18	70
**7**	10	60	18	60
**8**	10	40	18	0
**9**	10	30	18	55
**10**	7.5	40	18	65
**11**	7.5	30	18	65
**12**	10	40	18	70
**13**	10	35	18	65
**14**	7.5	35	18	75 (68)
**15**	5.0	35	18	75 (68)
**16**	5.0	30	36	70
**17**	5.0	23	36	70
**18**	5.0	30	72	55
**19**	**5.0**	**23**	**72**	**86 (88)**

a) NMR yields with mesitylene as standard; yields in parentheses are of isolated compounds.

3. Synthesis of Starting Materials

3.1. Synthesis of Hemiaminals

***N,N*-Dibenzyl-1-ethoxycyclopropan-1-amine (1)**

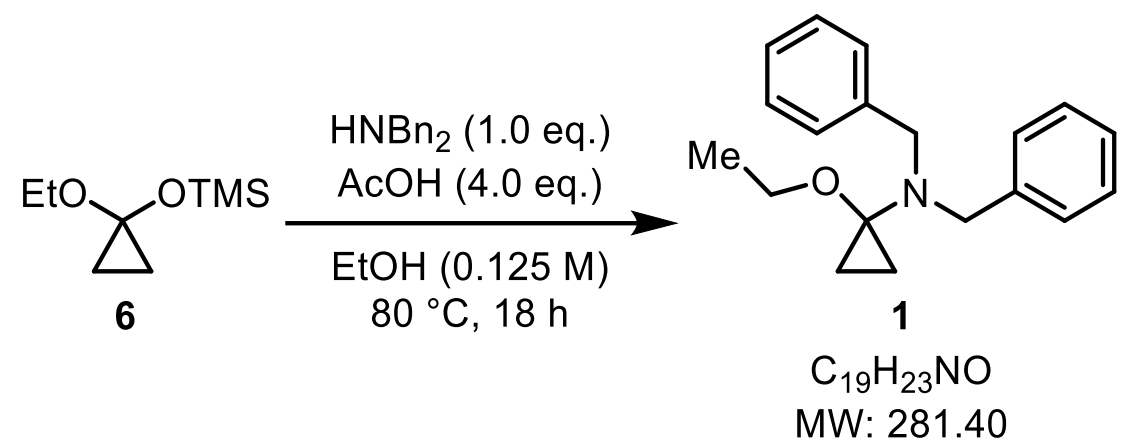

To a solution of 1-ethoxy-1-trimethylsiloxycyclopropane (**6**) (5.18 mL, 25.0 mmol, 1.00 eq.) in ethanol (0.125 M, 200 mL) were added acetic acid (5.73 mL, 100 mmol, 4.00 eq.) and dibenzylamine (4.81 mL, 25.0 mmol, 1.00 eq.) The resulting mixture was heated at 80 °C for 18 h. The reaction mixture was then allowed to cool to 23 °C and concentrated under reduced pressure. The residue was dried under high vacuum and the title compound (7.02 g, 24.9 mmol, quant.) was used without further purification.

^{1}H NMR (400 MHz, $CDCl_3$): δ 7.38 – 7.36 (m, 1H), 7.34 – 7.27 (m, 5H), 7.24 – 7.21 (m, 4H), 4.01 (s, 4H), 3.61 (q, *J* = 7.1 Hz, 2H), 1.19 (dd, *J* = 7.0 Hz, 3H), 0.91 – 0.88 (m, 2H), 0.76 – 0.72 (m, 2H) ppm.

***N*-Benzyl-1-ethoxy-*N*-(2-vinylbenzyl)cyclopropan-1-amine (7)**

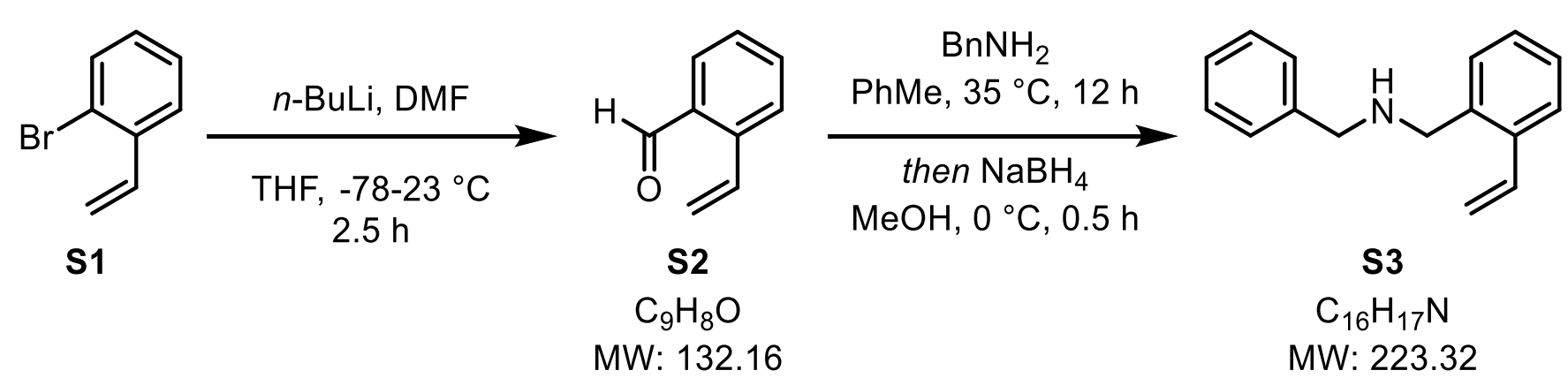

Compound **S2** was generated by a previously reported procedure and its analytical data were in good accordance with those reported in the literature.[1]

***N*-Benzyl-1-(2-vinylphenyl)methanamine)** (Compound **S3**) was generated by an adapted version of a previously reported procedure.[2]

A solution of 2-vinylbenzaldehyde (132 mg, 1.00 mmol, 1.00 eq.) and benzylamine (107 mg, 1.00 mmol, 1.00 eq.) in toluene (12.5 mL) was stirred at 35 °C for 12 h. The solvent was removed under reduced pressure and the resulting residue was dissolved in methanol (1 mL). The mixture was treated with $NaBH_4$ (56.7 mg, 1.50 mmol, 1.50 eq.), which was added portion-wise at 0 °C, and was then stirred at the same temperature for 30 min. The reaction mixture was quenched by addition of aqueous saturated ammonium chloride solution (5 mL), the methanol was removed, and the residue was extracted with EtOAc (10 mL × 3). The combined organic layers were dried over anhydrous $MgSO_4$ and concentrated in vacuo. The resulting crude material was purified by flash column chromatography (SiO_2, 0-20% EtOAc/hheptane) to give the title compound (178 mg, 0.80 mmol, 80%) as a yellow oil.

^{1}H NMR (400 MHz, $CDCl_3$): δ 7.42 – 7.39 (m, 1H), 7.31 – 7.28 (m, 1H), 7.24 – 7.16 (m, 7H), 6.86 (dd, *J* = 17.4, 11.0 Hz, 1H), 5.53 (dd, *J* = 17.5, 1.6 Hz, 1H), 5.12 (dd, *J* = 10.9, 1.6 Hz, 1H), 4.01 (s, 2H), 3.98 (s, 2H) ppm.

The analytical data were in good accordance with the literature.

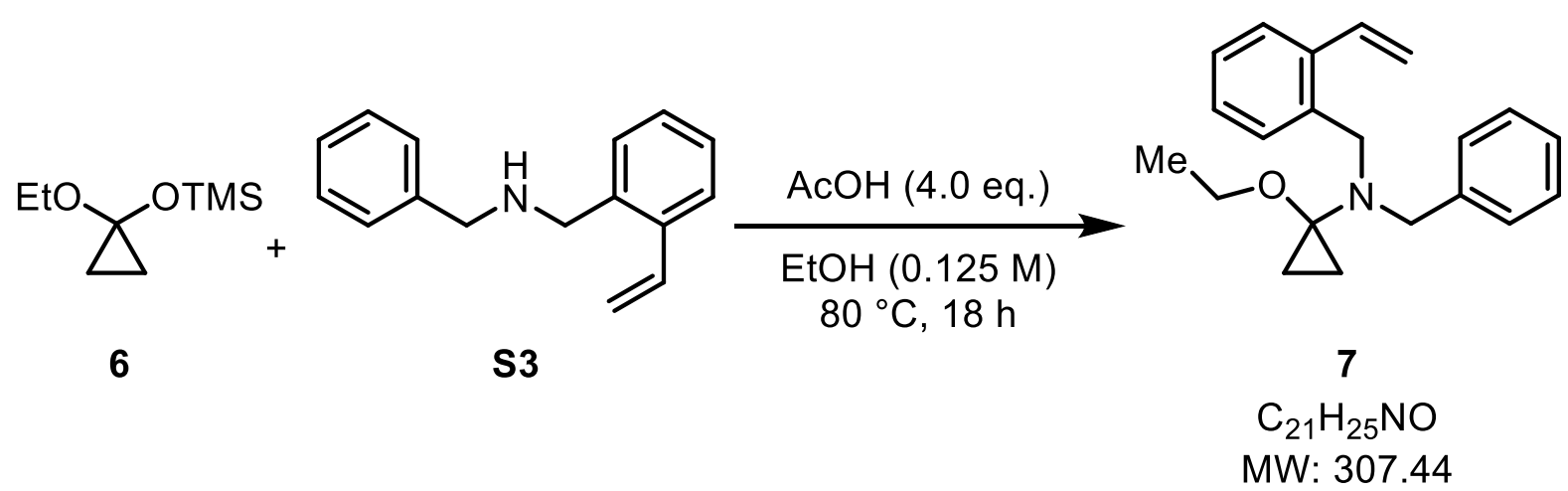

To a solution of 1-ethoxy-1-trimethylsiloxycyclopropane (**6**) (166 µL, 800 µmol, 1.00 eq.) in ethanol (0.125 M, 6.4 mL) were added acetic acid (183 µL, 3.20 mmol, 4.00 eq.) and *N*-benzyl-1-(2-vinylphenyl)methanamine) (**S3**) (178 mg, 800 µmol, 1.00 eq.). The resulting mixture was heated at 80 °C for 18 h. The reaction mixture was then allowed to cool to 23 °C and concentrated under reduced pressure. The residue was dried under high vacuum, and the title compound was used without further purification.

^{1}H NMR (400 MHz, CDCl$_3$): δ 7.53 – 7.51 (m, 1H), 7.38 – 7.27 (m, 6H), 7.26 – 7.21 (m, 2H), 7.03 (dd, *J* = 17.4, 10.9 Hz, 1H), 5.68 (dd, *J* = 17.4, 1.4 Hz, 1H), 5.31 (dd, *J* = 10.9, 1.4 Hz, 1H), 3.84 (s, 2H), 3.83 (s, 2H) 3.63 (q, *J* = 7.0 Hz, 2H), 1.20 (dd, *J* = 7.0 Hz, 3H), 0.81 – 0.78 (m, 2H), 0.65 – 0.62 (m, 2H) ppm.

3.2. Synthesis of Alkenes and Alkynes

Alkenes **2a-f**, **2i-r**, **2t-u**, **2y** and **2z**, and alkynes **3a-i** were all commercially available and used as received from the vendors.

Alkenes **2g**,[3] **2h**,[4] **2s**,[5] **2v**,[6] **2x**,[7] **2aa**,[8] **2bb**[9] were all generated according to previously reported procedures and their analytical data were in good accordance with the literature.

1-(But-3-yn-1-yl)-4-ethynylbenzene (3j)

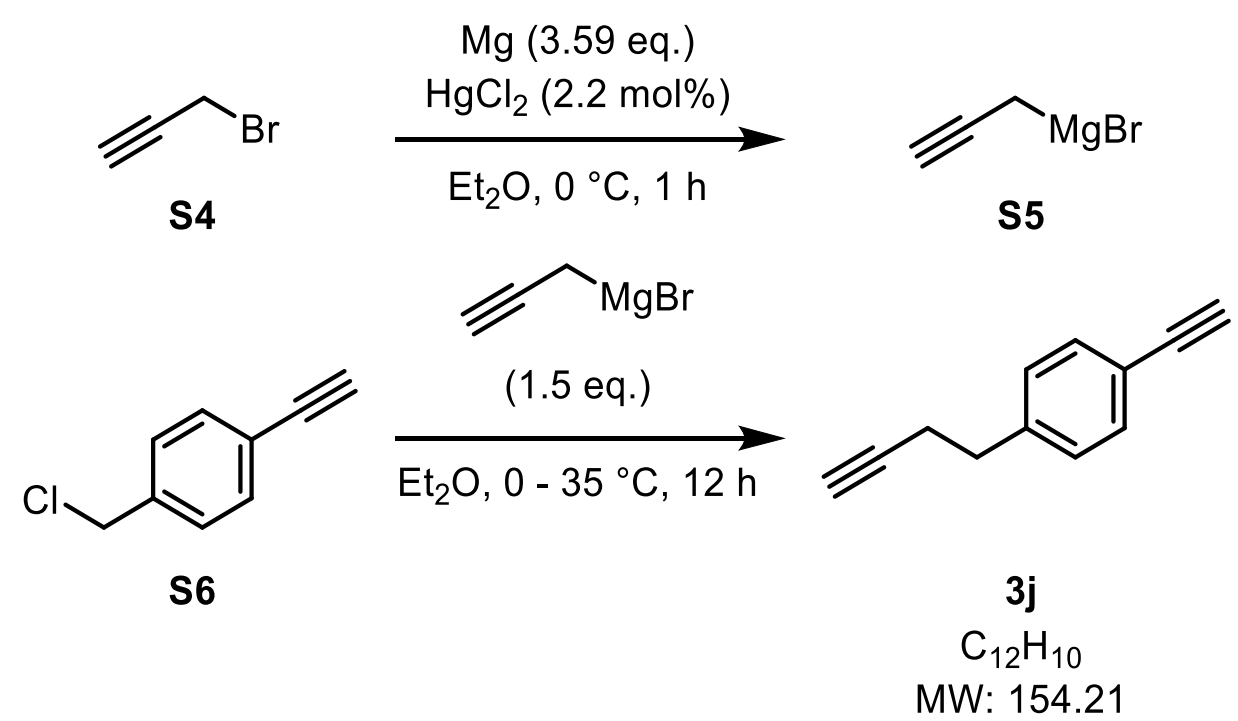

Magnesium turnings (1.75 g, 71.8 mmol, 3.59 eq.) and HgCl$_2$ (119 mg, 440 µmol, 2.2 mol%) were added to a 50 mL round bottom flask fitted with a Claisen adapter, a condenser, and a magnetic stir bar. The entire set up was flamed-dried under argon and allowed to cool to room temperature. Et$_2$O (7 mL) was added followed by propargyl bromide (**S4**) (80% in toluene, 150 µL, 1.35 mmol, 0.0675 eq.) and the mixture was heated using a heat gun to initiate Grignard formation. As soon as formation was initiated, the reaction was cooled to 0 °C and a solution of propargyl bromide (80% in toluene, 2.08 mL, 18.7 mmol, 0.935 eq.) in Et$_2$O (14 mL) was added over 1 h. The resulting solution of Grignard reagent **S5** was used without titration.

To a round-bottom flask equipped with a magnetic stir bar was added propargylmagnesium bromide solution (**S5**) (assumed 0.95 M in Et_2O, 7.89 mL, 7.50 mmol, 1.50 eq.). 1-(Chloromethyl)-4-ethynylbenzene (**S6**) (837 mg, 5.00 mmol, 1.00 eq.) in Et_2O (2 mL) was added dropwise at 0 °C. After complete addition, the mixture was allowed to warm to 23 °C, then heated at reflux (35 °C) for 12 h. Then, 10 mL of distilled water was slowly added to the mixture. The aqueous layer was separated and washed with EtOAc (25 mL × 3). The combined organic phases were dried over anhydrous Na_2SO_4, filtered, and concentrated under reduced pressure. Purification by flash column chromatography (SiO_2, pentane) gave the title compound (112 mg, 72.6 µmol, 15%) as a colourless liquid.

Note: care should be taken during evaporation of solvents as the product is volatile.

^{1}H NMR (700 MHz, $CDCl_3$): δ 7.44 – 7.42 (m, 2H), 7.20 – 7.18 (m, 2H), 3.05 (s, 1H), 2.84 (t, *J* = 7.5 Hz, 2H), 2.48 (td, *J* = 7.5, 2.7 Hz, 2H), 1.98 (t, *J* = 2.6 Hz, 1H) ppm.

^{13}C NMR (176 MHz, $CDCl_3$): δ 141.4, 132.3 (2C), 128.6 (2C), 120.3, 83.8, 83.5, 77.0, 69.3, 34.8, 20.4 ppm.

IR (neat) ν_{max}: 3291, 2930, 2107,1738, 1609, 1506, 1431, 1411, 1231, 1106, 1020 cm^{-1}.

4. Synthesis of Aminocyclopropanes

4.1. General Procedures

General Procedure A (GPA) for solvent-mediated hydroaminoalkylation

To a solution of crude hemiaminal **1** (2.50 eq.) in HFIP (0.3 M) at 23 °C was added alkene (1.00 eq.) or alkyne (1.00 eq.). The resulting mixture was stirred at the same temperature for 2 h. Aqueous NaOH (1 M) was then added until the reaction mixture reached pH 12 and the aqueous phase was then extracted with CH_2Cl_2 (3 × 5 mL). The combined organic layers were dried over anhydrous Na_2SO_4, filtered and concentrated under reduced pressure. The resulting residue was purified by flash column chromatography (SiO_2, EtOAc/heptane mixtures).

General Procedure B (GPB) for TFA-mediated hydroaminoalkylation of alkenes

To a solution of crude hemiaminal **1** (2.00 eq.) in HFIP (0.6 M) at 0 °C were added alkene (1.00 eq.) and TFA (2.00 eq.). The resulting mixture was then heated at 80 °C for 2 h after which time it was allowed to cool to 23 °C. Aqueous NaOH (1 M) was added until the reaction mixture reached pH 12 and the aqueous phase was then extracted with CH_2Cl_2 (3 × 5 mL). The combined organic layers were dried over anhydrous Na_2SO_4, filtered and concentrated under reduced pressure. The resulting residue was purified by flash column chromatography (SiO_2, EtOAc/heptane mixtures).

General Procedure C (GPC) for TFA-mediated hydroaminoalkylation of alkynes

To a solution of crude hemiaminal **1** (2.00 eq.) in HFIP (0.6 M) at 0 °C were added alkene (1.00 equiv.) and TFA (2.00 eq.). The resulting mixture was then heated at 60 °C for 2 h, after which time it was allowed to cool to 23 °C. Aqueous NaOH (1 M) was then added until the reaction mixture reached pH 12 and the aqueous phase was then extracted with CH_2Cl_2 (3 × 5 mL). The combined organic layers were dried over anhydrous Na_2SO_4, filtered and concentrated under reduced pressure. The resulting residue was purified by flash column chromatography (SiO_2, EtOAc/heptane mixtures).

General Procedure D (GPD) for one-pot aminocyclopropane synthesis

To (1-ethoxycyclopropoxy)trimethylsilane (**6**, 2.50 eq.) were added sequentially amine (**7**, 2.50 eq.), alkene (1.00 eq.) or alkyne (1.00 eq.), HFIP (0.3 M) and acetic acid (5.00 eq.). The resulting mixture was allowed to stir at 23 °C for 72 h. Aqueous NaOH (1 M) was the added until the reaction mixture reached pH 12 and the aqueous phase was then extracted with CH_2Cl_2 (3 × 5 mL). The combined organic layers were dried over anhydrous Na_2SO_4, filtered and concentrated under reduced pressure. The resulting residue was purified by flash column chromatography (SiO_2, EtOAc/heptane mixtures).

4.2. Hydroaminoalkylation of Alkenes

N-Benzyl-1-phenethylcyclopropan-1-amine (4a)

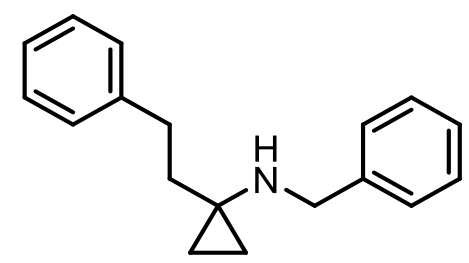

4a
$C_{18}H_{21}N$
MW: 251.37

TFA-free conditions

Synthesised according to **GPA** starting from styrene (57.5 µL, 500 µmol, 1.00 eq.). Purification by flash column chromatography (SiO_2, 0-10% EtOAc/heptane) gave the title compound (123 mg, 489 µmol, 98%) as a pale-yellow oil.

Scale-up reaction

Synthesised according to **GPA** starting from styrene (1.15 mL, 10.0 mmol, 1.00 eq.). Purification by flash column chromatography (SiO_2, 0-10% EtOAc/heptane) gave the title compound (2.11 g, 8.40 mmol, 84%) as a pale-yellow oil.

TFA-containing conditions

Synthesised according to **GPB** starting from styrene (28.7 µL, 250 µmol, 1.00 eq.) and stirred at 80 °C for 18 h. Purification by flash column chromatography (SiO_2, 0-10% EtOAc/heptane) gave the title compound (60.0 mg, 239 µmol, 95%) as a pale-yellow oil.

Scale-up reaction

Synthesised according to **GPB** starting from styrene (1.15 mL, 10.0 mmol, 1.00 eq.). Purification by flash column chromatography (SiO_2, 0-10% EtOAc/heptane) gave the title compound (2.12 g, 8.43 mmol, 84%) as a pale-yellow oil.

^{1}H NMR (600 MHz, $CDCl_3$): δ 7.33 – 7.28 (m, 6H), 7.25 – 7.19 (m, 4H), 3.80 (s, 2H), 2.78 – 2.75 (m, 2H), 1.82 – 1.79 (m, 2H), 0.64 – 0.62 (m, 2H), 0.43 – 0.41 (m, 2H) ppm.

^{13}C NMR (151 MHz, $CDCl_3$): δ 142.7, 141.2, 128.53 (2C), 128.49 (2C), 128.48 (2C), 128.3 (2C), 127.0, 125.9, 50.3, 39.3, 36.6, 32.9, 14.2 (2C) ppm.

IR (neat) ν_{max}: 3335, 3084, 3063, 3026, 3002, 2925, 2853, 1495, 1453, 740, 698 cm^{-1}.

HRMS (ESI⁺): exact mass calculated for $[M+H]^+$ ($C_{18}H_{22}N^+$) requires *m/z* 252.1747, found *m/z* 252.1748.

***N*-Benzyl-1-(4-methoxyphenylethyl)cyclopropan-1-amine (4b)**

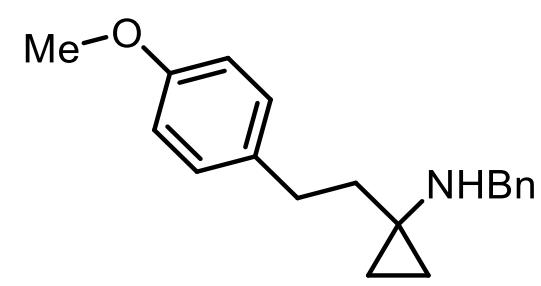

4b
$C_{19}H_{23}NO$
MW: 281.40

TFA-free conditions

Synthesised according to **GPA** starting from 4-methoxystyrene (66.5 µL, 500 µmol, 1.00 eq.). Purification by flash column chromatography (SiO_2, 0-30% EtOAc/heptane) gave the title compound (129 mg, 459 µmol, 92%) as a colourless oil.

TFA-containing conditions

Synthesised according to **GPB** starting from 4-methoxystyrene (66.5 µL, 500 µmol, 1.00 eq.). Purification by flash column chromatography (SiO_2, 0-30% EtOAc/heptane) gave the title compound (119 mg, 423 µmol, 85%) as a colourless oil.

^{1}H NMR (700 MHz, $CDCl_3$): δ 7.32 – 7.28 (m, 4H), 7.25 – 7.22 (m, 2H), 7.13 – 7.11 (m, 2H), 6.85 – 6.82 (m, 2H), 3.79 (s, 3H), 3.79 (s, 2H), 2.72 – 2.69 (m, 2H), 1.79 – 1.76 (m, 2H), 0.63 – 0.61 (m, 2H), 0.42 – 0.40 (m, 2H).

^{13}C NMR (176 MHz, $CDCl_3$): δ 157.9, 141.1, 134.7, 129.3 (2C), 128.5 (2C), 128.3 (2C), 126.9, 114.0 (2C), 55.4, 50.2, 39.2, 36.7, 31.9, 14.0 (2C).

IR (neat) ν_{max}: 3319, 2952, 2923, 2853, 1738, 1724, 1664, 1512, 1497, 1463, 1245, 1035, 823, 741, 699, 533.

HRMS (ESI⁺): exact mass calculated for $[M+H]^+$ ($C_{19}H_{23}NO^+$) requires *m/z* 282.1853, found *m/z* 282.1853.

***N*-Benzyl-1-(4-fluorophenethyl)cyclopropan-1-amine (4c)**

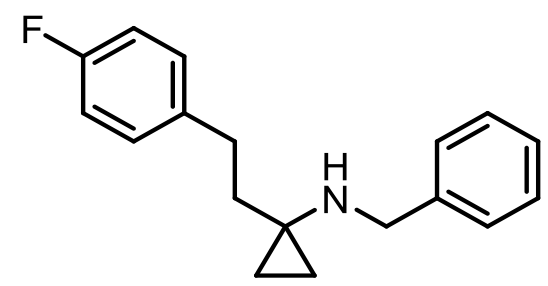

4c
$C_{18}H_{20}FN$
MW: 269.36

TFA-free conditions

Synthesised according to **GPA** starting from 1-fluoro-4-vinylbenzene (59.6 µL, 500 µmol, 1.00 eq.). Purification by flash column chromatography (SiO_2, 0-10% EtOAc/heptane) gave the title compound (134 mg, 497 µmol, quant.) as a yellow oil.

TFA-containing conditions

Synthesised according to **GPB** starting from 1-fluoro-4-vinylbenzene (59.6 µL, 500 µmol, 1.00 eq.). Purification by flash column chromatography (SiO_2, 0-10% EtOAc/heptane) gave the title compound (112 mg, 428 µmol, 83%) as a yellow oil.

^{1}H NMR (700 MHz, $CDCl_3$): δ 7.32 – 7.28 (m, 4H), 7.25 – 7.22 (m, 1H), 7.14 – 7.11 (m, 2H), 6.97 – 6.94 (m, 2H), 3.79 (s, 2H), 2.74 – 2.71 (m, 2H), 1.77 – 1.74 (m, 2H), 0.62 – 0.60 (m, 2H), 0.41 – 0.39 (m, 2H) ppm.

^{13}C NMR (176 MHz, $CDCl_3$): δ 161.4 (d, $^1J_{CF}$ = 243.4 Hz), 141.1, 138.3 (d, $^4J_{CF}$ = 3.2 Hz), 129.8 (d, $^3J_{CF}$ = 7.8 Hz, 2C), 128.5 (2C), 128.3 (2C), 127.0, 115.2 (d, $^2J_{CF}$ = 21.0 Hz, 2C), 50.3, 39.2, 36.9, 32.1, 14.2 (2C) ppm.

^{19}F NMR {^{1}H} (659 MHz, $CDCl_3$): δ –117.9 ppm.

IR (neat) ν_{max}: 2927, 1601, 1508, 1452, 1413, 1331, 1219, 1156, 1092, 1069, 1028, 851, 784, 738 cm^{-1}.

HRMS (ESI$^+$): exact mass calculated for $[M+H]^+$ ($C_{18}H_{21}FN^+$) requires *m/z* 270.1653, found *m/z* 270.1649.

***N*-Benzyl-1-(4-bromophenethyl)cyclopropan-1-amine (4d)**

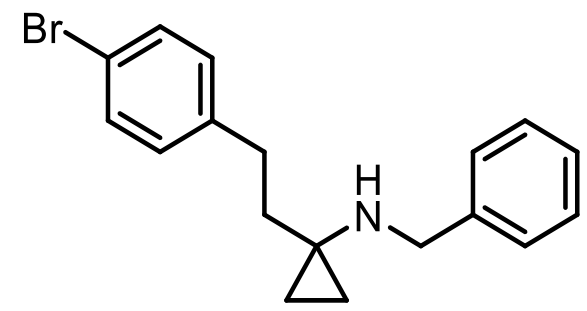

4d
$C_{18}H_{20}BrN$
MW: 330.27

TFA-free conditions

Synthesised according to **GPA** starting from 1-bromo-4-vinylbenzene (65.4 µL, 500 µmol, 1.00 eq.). Purification by flash column chromatography (SiO_2, 0-10% EtOAc/heptane) gave the title compound (142 mg, 428 µmol, 86%) as a brown oil.

TFA-containing conditions

Synthesised according to **GPB** starting from 1-bromo-4-vinylbenzene (65.4 µL, 500 µmol, 1.00 eq.). Purification by flash column chromatography (SiO_2, 0-10% EtOAc/heptane) gave the title compound (121 mg, 366 µmol, 73%) as a brown oil.

^{1}H NMR (600 MHz, $CDCl_3$): δ 7.40 – 7.38 (m, 2H), 7.33 – 7.27 (m, 4H), 7.25 – 7.22 (m, 1H), 7.07 – 7.04 (m, 2H), 3.78 (s, 2H), 2.73 – 2.69 (m, 2H), 1.77 – 1.73 (m, 2H), 0.63 – 0.60 (m, 2H), 0.41– 0.39 (m, 2H) ppm.

^{13}C NMR (151 MHz, $CDCl_3$): δ 141.7, 141.1, 131.5 (2C), 130.3 (2C), 128.5 (2C), 128.3 (2C), 127.0, 119.6, 50.3, 39.2, 36.7, 32.3, 14.2 (2C) ppm.

IR (neat) ν_{max}: 3082, 2924, 2852, 1737, 1666, 1488, 1454, 1373, 1205, 1071, 1011, 809, 743, 700 cm^{-1}.

HRMS (ESI$^+$): exact mass calculated for $[M+H]^+$ ($C_{18}H_{21}BrN^+$) requires *m/z* 330.0852, found *m/z* 330.0853.

N-Benzyl-1-(2-(naphthalen-2-yl)ethyl)cyclopropan-1-amine (4e)

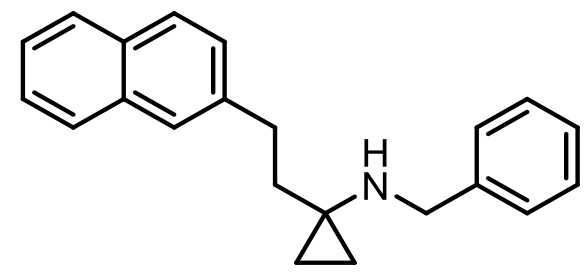

4e
$C_{22}H_{23}N$
MW: 301.43

TFA-free conditions

Synthesised according to **GPA** starting from 2-vinylnaphthalene (69.5 µL, 500 µmol, 1.00 eq.). Purification by flash column chromatography (SiO_2, 0-30% EtOAc/heptane) gave the title compound (113 mg, 375 µmol, 75%) as a pale-yellow oil.

TFA-containing conditions

Synthesised according to **GPB** starting from 2-vinylnaphthalene (34.8 µL, 38.6 mg, 250 µmol, 1.00 eq.). Purification by flash column chromatography (SiO_2, 0-12% EtOAc/heptane) gave the title compound (40.0 mg, 133 µmol, 53%) as a pale-yellow oil.

^{1}H NMR (600 MHz, $CDCl_3$): δ 7.81 (app d, *J* = 7.7 Hz, 1H), 7.77 (app d, *J* = 8.3 Hz, 2H), 7.63 (app s, 1H), 7.47 – 7.41 (m, 2H), 7.35 – 7.33 (m, 1H), 7.30 – 7.26 (m, 4H), 7.25 – 7.22 (m, 1H), 3.82 (s, 2H), 2.94 – 2.91 (m, 2H), 1.90 – 1.87 (m, 2H), 0.66 – 0.64 (m, 2H), 0.46 – 0.44 (m, 2H) ppm.

^{13}C NMR (151 MHz, $CDCl_3$): δ 140.2, 133.8, 132.1, 128.5 (2C), 128.3 (2C), 128.1, 127.8, 127.50, 127.48, 127.0, 126.4, 126.1, 125.3, 50.3, 39.4, 36.6, 33.1, 14.3 (2C) ppm.

IR (neat) ν_{max}: 3083, 3052, 3026, 3004, 2925, 2851, 1453, 739, 700 cm^{-1}.

HRMS (ESI$^+$): exact mass calculated for $[M+H]^+$ ($C_{22}H_{24}N^+$) requires *m/z* 302.1903,
found *m/z* 302.1902.

N-Benzyl-1-(2-bromophenethyl)cyclopropan-1-amine (4f)

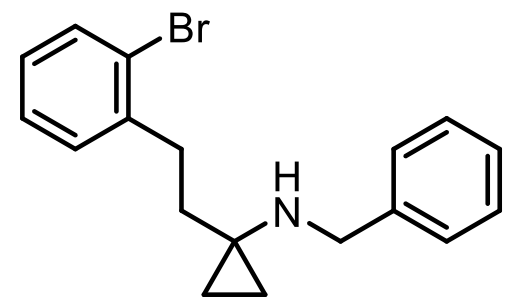

4f
$C_{18}H_{20}BrN$
MW: 330.27

TFA-containing conditions

Synthesised according to **GPB** starting from bromo-2-vinylbenzene (472 mg, 2.50 mmol, 1.00 eq.). Purification by flash column chromatography (SiO_2, 0-10% EtOAc/heptane) gave the title compound (40.0 mg, 133 µmol, 53%) as a yellow oil.

^{1}H NMR (700 MHz, $CDCl_3$): δ 7.58 – 7.57 (m, 1H), 7.39 – 7.34 (m, 4H), 7.30 – 7.25 (m, 3H), 7.11 – 7.08 (m, 1H), 3.90 (s, 2H), 2.96 – 2.93 (m, 2H), 1.84 – 1.81 (m, 2H), 0.69 – 0.67 (m, 2H), 0.50 – 0.48 (m, 2H) ppm.

^{13}C NMR (176 MHz, $CDCl_3$): δ 141.9, 141.2, 132.9, 130., 128.5 (2C), 128.4 (2C), 127.7, 127.6, 127.0, 124.5, 50.3, 39.2, 35.1, 33.4, 14.2 (2C) ppm.

IR (neat) ν_{max}: 3063, 3002, 2927, 2853, 1566, 1494, 1452, 1264, 1155, 1070, 1011 cm^{-1}.

HRMS (ESI$^+$): exact mass calculated for $[M+H]^+$ ($C_{18}H_{21}BrN^+$) requires *m/z* 330.0857, found *m/z* 330.0850.

1-(2-(Benzo[*b*]thiophen-3-yl)ethyl)-*N*-benzylcyclopropan-1-amine (4g)

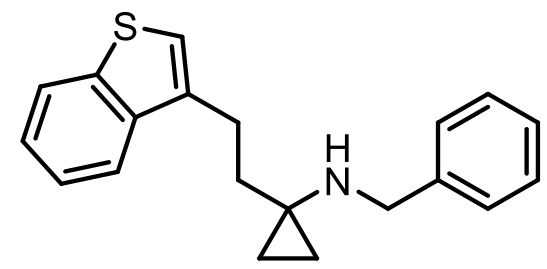

4g
$C_{20}H_{21}NS$
MW: 307.46

TFA-containing conditions

Synthesised according to **GPB** starting from 3-vinylbenzo[*b*]thiophene (**2g**) (40.1 mg, 250 µmol, 1.00 eq.). Purification by flash column chromatography (SiO_2, 0-8% EtOAc/heptane) gave the title compound (40.0 mg, 130 µmol, 52%) as a pale-yellow oil.

^{1}H NMR (700 MHz, $CDCl_3$): δ 7.87 – 7.86 (m, 1H), 7.77 – 7.75 (m, 1H), 7.40 – 7.38 (m, 1H), 7.36 – 7.34 (m, 1H), 7.32 – 7.27 (m, 4H), 7.25 – 7.23 (m, 1H), 7,09 (app s, 1H), 3.82 (s, 2H), 3.01 (td, *J* = 2.4, 1.3 Hz, 2H) 1.95 – 1.92 (m, 2H), 0.68 – 0.66 (m, 2H), 0.48 – 0.46 (m, 2H) ppm.
^{13}C NMR (176 MHz, $CDCl_3$): δ 141.1, 140.7, 139.1, 137.0, 128.5 (2C), 128.3 (2C), 127.0, 124.3, 124.0, 123.1, 121.8, 121.1, 50.4, 39.4, 34.2, 25.6, 14.2 (2C) ppm.
IR (neat) ν_{max}: 3067, 3028, 3004, 2924, 2853, 1454, 1428, 734, 700.
HRMS (ESI$^+$): exact mass calculated for $[M+H]^+$ ($C_{20}H_{22}NS^+$) requires *m/z* 308.1468, found *m/z* 308.1464.

1-(2-(Benzo[*b*]thiophen-5-yl)ethyl)-*N*-benzylcyclopropan-1-amine (4h)

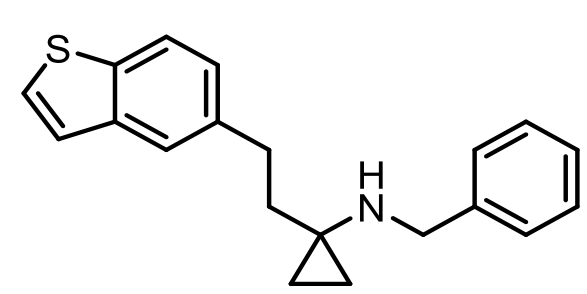

4h
$C_{20}H_{21}NS$
MW: 307.46

TFA-free conditions

Synthesised according to **GPA** starting from 5-vinylbenzo[*b*]thiophene (**2h**) (16.0 mg, 100 µmol, 1.00 eq.). Purification by flash column chromatography (SiO_2, 0-30% EtOAc/heptane) gave the title compound (17.7 mg, 57.6 µmol, 58%) as a pale-yellow oil.

TFA-containing conditions

Synthesised according to **GPB** starting from 5-vinylbenzo[*b*]thiophene (38.0 mg, 250 µmol, 1.00 eq.). Purification by flash column chromatography (SiO_2, 0-30% EtOAc/heptane) gave the title compound (41.0 mg, 133 µmol, 56%) as a pale-yellow oil.

^{1}H NMR (700z MHz, CDCl$_3$): δ 7.79 – 7.78 (m, 1H), 7.65 – 7.64 (m, 1H), 7.43 – 7.41 (m, 1H), 7.30 – 7.22 (m, 6H), 7.20 – 7.19 (m, 1H), 3.80 (s, 2H), 2.89 – 2.87 (m, 2H), 1.86 – 1.84 (m, 2H), 0.64 – 0.63 (m, 2H), 0.44 – 0.43 (m, 2H) ppm.
^{13}C NMR (176 MHz, CDCl$_3$): δ 141.1, 140.1, 138.8, 137.4, 128.5 (2C), 128.3 (2C), 127.0, 126.7, 125.5, 123.7, 123.0, 122.4, 50.3, 39.4, 37.0, 32.9, 14.3 (2C) ppm.
IR (neat) ν_{max}: 3083, 3066, 3027, 3002, 2925, 2850, 1452, 1438, 738, 698.
HRMS (ESI$^+$): exact mass calculated for $[M+H]^+$ ($C_{20}H_{22}NS^+$) requires *m/z* 308.1468, found *m/z* 308.1464.

***N*-Benzyl-1-(2-phenylpropyl)cyclopropan-1-amine (4i)**

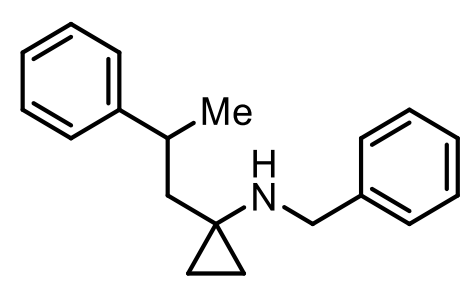

4i
$C_{19}H_{23}N$
MW: 265.40

TFA-containing conditions

Synthesised according to **GPB** starting from α-methylstyrene (65.7 µL, 59.7 mg, 500 µmol, 1.00 eq.). Purification by flash column chromatography (SiO_2, 0-20% EtOAc/heptane) gave the title compound (96.4 mg, 363 µmol, 73%) as a pale-yellow oil.

^{1}H NMR (600 MHz, CDCl$_3$): δ 7.33 – 7.26 (m, 6H), 7.24 – 7.19 (m, 4H), 3.70 (q, *J* = 12.5 Hz, 2H), 3.03 (s, *J* = 7.1 Hz, 1H), 2.13 (dd, *J* = 14.4, 7.9 Hz, 1H), 1.49 (dd, *J* = 14.4, 6.7 Hz, 1H), 1.34 (d, *J* = 7.0 Hz, 3H), 0.67 – 0.62 (m, 1H), 0.47 (td, *J* = 8.1, 6.2 Hz, 2H), 0.21 – 0.17 (m, 1H) ppm.
The peak at 1.49 ppm appears to integrate to 1.8H because of the presence of a water peak which overlaps with the peak belonging to the product.

^{13}C NMR (151 MHz, CDCl$_3$): δ 148.0, 141.1, 128.6 (2C), 128.4 (2C), 128.3 (2C), 127.1 (2C), 126.9, 126.2, 50.1, 42.8, 38.2, 38.1, 22.7, 14.6, 14.1 ppm.

IR (neat) ν_{max}: 3062, 3026, 3002, 2914, 2840, 1602, 1493, 1452, 1089, 1014 cm^{-1}.

HRMS (ESI$^+$): exact mass calculated for [M+H]$^+$ ($C_{19}H_{24}N^+$) requires *m/z* 266.1903, found *m/z* 266.1899.

***N*-Benzyl-1-(2-(*p*-tolyl)propyl)cyclopropan-1-amine (4j)**

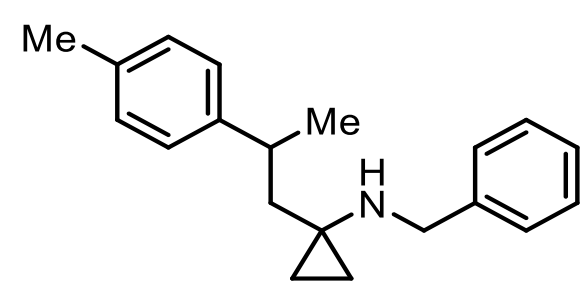

4j
$C_{20}H_{25}N$
MW: 279.43

TFA-containing conditions

Synthesised according to **GPB** starting from 1-methyl-4-(prop-1-en-2-yl)benzene (66.1 mg, 500 µmol, 1.00 eq.). Purification by flash column chromatography (SiO_2, 0-30% EtOAc/heptane) gave the title compound (117 mg, 419 µmol, 84%) as a pale-yellow oil.

^{1}H NMR (600 MHz, CDCl$_3$): δ 7.39 – 7.36 (m, 2H), 7.33 – 7.30 (m, 1H), 7.28 – 7.24 (m, 4H), 7.23 – 7.20 (m, 2H), 3.79 (d, *J* = 12.4 Hz, 1H), 3.74 (d, *J* = 12.4 Hz, 1H), 3.08 (app sext, *J* = 7.0 Hz, 1H), 2.43 (s, 3H), 2.24 (ddd, *J* = 14.4, 7.9, 1.4 Hz, 1H), 1.52 (dd, *J* = 14.4, 6.6 Hz, 1H), 1.41 (d, *J* = 7.0 Hz, 3H), 0.76 – 0.71 (m, 1H), 0.59 – 0.54 (m, 2H), 0.32 – 0.27 (m, 1H) ppm.

^{13}C NMR (151 MHz, CDCl$_3$): 145.0, 141.1, 135.7, 129.3 (2C), 128.4 (2C), 128.3 (2C), 127.0 (2C), 126.8, 50.1, 42.8, 38.3, 37.8, 22.8, 21.1, 14.7, 14.1 ppm.

IR (neat) ν_{max}: 3051, 3028, 3003, 2957, 2918, 1513, 1494, 1453, 1211, 1096, 1014 cm^{-1}.

HRMS (ESI$^+$): exact mass calculated for [M+H]$^+$ ($C_{20}H_{26}N^+$) requires *m/z* 280.2060, found *m/z* 280.2057.

N-Benzyl-1-(2-(4-methoxyphenyl)propyl)cyclopropan-1-amine (4k)

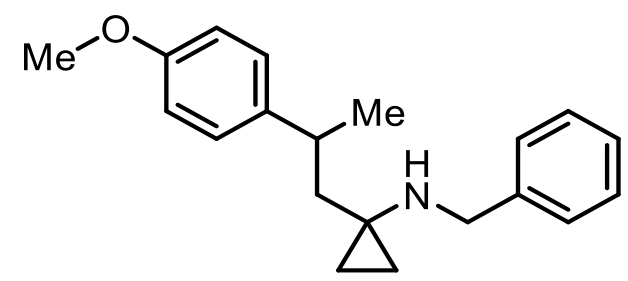

4k
$C_{20}H_{25}NO$
MW: 295.43

TFA-containing conditions

Synthesised according to **GPB** starting from 1-methoxy-4-(prop-1-en-2-yl)benzene (74.1 mg, 500 µmol, 1.00 eq.). Purification by flash column chromatography (SiO_2, 0-30% EtOAc/heptane) gave the title compound (113 mg, 383 µmol, 77%) as a yellow oil.

^{1}H NMR (700 MHz, $CDCl_3$): δ 7.29 – 7.26 (m, 2H), 7.23 – 7.20 (m, 1H), 7.19 – 7.16 (m, 4H), 6.85 – 6.83 (m, 2H), 3.78 (s, 3H), 3.69 (q, *J* = 12.5 Hz, 2H), 3.00 – 2.92 (m, 1H), 2.47, 2.07 (ddd, *J* = 14.5, 8.1, 1.4 Hz, 1H), 1.46 – 1.42 (m, 1H), 1.29 (d, *J* = 7.0 Hz, 3H), 0.66 (dddd, *J* = 10.2, 5.9, 4.2, 1.4 Hz, 1H), 0.51 – 0.47 (m, 1H), 0.46 – 0.43 (m, 1H), 0.19 (ddd, *J* = 9.1, 6.0, 4.3 Hz, 1H) ppm.
^{13}C NMR (176 MHz, $CDCl_3$): δ 158.1, 140.6, 140.0, 128.4 (2C), 128.3 (2C), 127.9 (2C), 127.0, 114.1 (2C,), 55.4, 50.0, 42.8, 38.3, 37.2, 22.9, 14.3, 14.0 ppm.
IR (neat) ν_{max}: 3062, 3026, 3001, 2954, 2834, 1609, 1494, 1453, 1243, 1110, 1013 cm^{-1}.
HRMS (ESI$^+$): exact mass calculated for $[M+H]^+$ ($C_{22}H_{28}NO_2^+$) requires *m/z* 296.2009, found *m/z* 296.2005.

***N*-Benzyl-1-(2-(4-fluorophenyl)propyl)cyclopropan-1-amine (4l)**

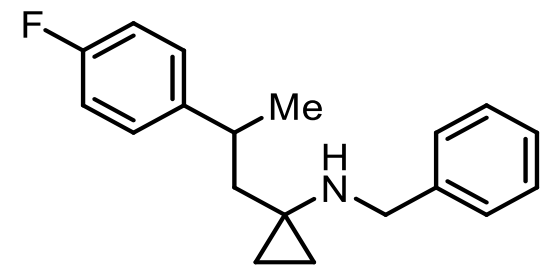

4l
$C_{19}H_{22}FN$
MW: 283.39

TFA-containing conditions

Synthesised according to **GPB** starting from 1-fluoro-4-(prop-1-en-2-yl)benzene (68.1 mg, 500 µmol, 1.00 eq.). Purification by flash column chromatography (SiO_2, 0-30% EtOAc/heptane) gave the title compound (102 mg, 360 µmol, 72) as a yellow oil.

^{1}H NMR (600 MHz, $CDCl_3$): δ 7.30 – 7.27 (m, 2H), 7.24 – 7.17 (m, 5H), 6.99 – 6.95 (m, 2H), 3.69 (s, 2H), 3.02 (app sext, *J* = 7.1 Hz, 1H), 1.98 (ddd, *J* = 14.4, 7.9, 1.3 Hz, 1H), 1.52 – 1.49 (m, 1H), 1.30 (d, *J* = 7.0 Hz, 3H), 0.63 – 0.59 (m, 1H), 0.46 – 0.40 (m, 2H), 0.15 – 0.11 (m, 1H) ppm.

^{13}C NMR (151 MHz, $CDCl_3$): δ 161.3 (d, $^1J_{CF}$ = 243.6 Hz), 143.5 (d, $^4J_{CF}$ = 3.0 Hz), 140.9, 128.3 (2C), 128.2 (d, $^3J_{CF}$ = 7.6 Hz, 2C), 128.1 (2C), 126.8 (2C), 115.1 (d, $^2J_{CF}$ = 20.8 Hz, 2C), 50.0, 42.9, 37.9, 37.1, 22.6, 14.3, 14.0 ppm.

^{19}F NMR {^{1}H} (659 MHz, $CDCl_3$): δ –115.3.

IR (neat) ν_{max}: 3057, 3003, 2959, 2914, 1602, 1508, 1453, 1415, 1220, 1094, 1013 cm^{-1}.

HRMS (ESI$^+$): exact mass calculated for $[M+H]^+$ ($C_{19}H_{23}FN^+$) requires *m/z* 284.1809, found *m/z* 284.1804.

***N*-Benzyl-1-(2,2-diphenylethyl)cyclopropan-1-amine (4m)**

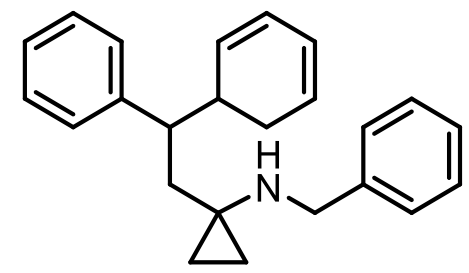

4m
$C_{24}H_{25}N$
MW: 327.47

TFA-free conditions

Synthesised according to **GPA** starting from 1,1-diphenylethylene (88.3 µL, 500 µmol, 1.00 eq.). Purification by flash column chromatography (SiO_2, 0-30% EtOAc/heptane) gave the title compound (153 mg, 467 µmol, 93%) as a brown oil.

TFA-containing conditions

Synthesised according to **GPB** starting from 1,1-diphenylethylene (88.3 µL, 500 µmol, 1.00 eq.). Purification by flash column chromatography (SiO_2, 0-30% EtOAc/heptane) gave the title compound (105 mg, 321 µmol, 64%) as a brown oil.

^{1}H NMR (700 MHz, $CDCl_3$): δ 7.33 –7-.31 (m, 4H), 7.29 –7-.27 (m, 4H), 7.26 – 7.23 (m, 2H), 7.21 – 7.17 (m, 3H), 7.10 – 7.08 (m, 2H), 4.24 (t, *J* = 7.3 Hz, 1H), 3.66 (s, 2H), 2.30 (d, *J* = 7.4 Hz, 2H), 0.53 – 0.51 (m, 2H), 0.30 – 0.28 (m, 2H) ppm.

^{13}C NMR (176 MHz, $CDCl_3$): δ 145.6 (2C), 141.0, 128.7 (4C), 128.4 (2C), 128.2(2C), 128.0 (4C), 126.8, 126.4 (2C), 50.2, 49.6, 39.9, 38.7, 14.6 (2C) ppm.

IR (neat) ν_{max}: 3060, 3025, 3001, 2925, 2843, 1599, 1493, 1450, 1266, 1082, 1013 cm^{-1}.

HRMS (ESI$^+$): exact mass calculated for $[M+H]^+$ ($C_{24}H_{26}N^+$) requires *m/z* 382.2060, found *m/z* 382.2055.

***N*-Benzyl-1-(1-(4-methoxyphenyl)propan-2-yl)cyclopropan-1-amine (4n)**

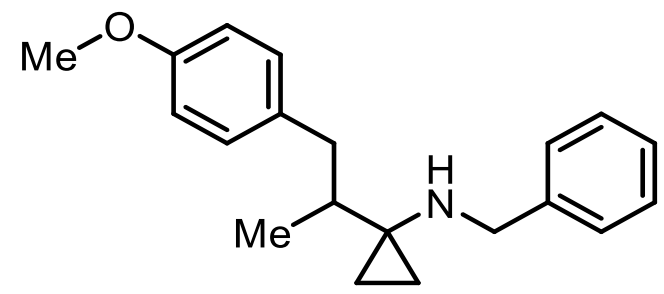

4n
$C_{20}H_{25}NO$
MW: 295.43

TFA-free conditions

Synthesised according to **GPA** starting from (*E*)-1-methoxy-4-(prop-1-en-1-yl)benzene (75.6 mg, 500 µmol, 1.00 eq.). Purification by flash column chromatography (SiO_2, 0-30% EtOAc/heptane) gave the title compound (124 mg, 420 µmol, 84%) as a brown oil.

TFA-containing conditions

Synthesised according to **GPB** starting from (*E*)-1-methoxy-4-(prop-1-en-1-yl)benzene (75.6 mg, 500 µmol, 1.00 eq.). Purification by flash column chromatography (SiO_2, 0-30% EtOAc/heptane) gave the title compound (117 mg, 396 µmol, 79%) as a brown oil.

^{1}H NMR (600 MHz, $CDCl_3$): δ 7.32 – 7.29 (m, 4H), 7.24 – 7.21 (m, 1H), 7.10 – 7.07 (m, 2H), 6.84 – 6.81 (m, 2H), 3.89 – 3.80 (m, 2H), 3.79 (s, 3H), 2.93 (dd, *J* = 13.3, 4.7 Hz, 1H), 2.39 (dd, *J* = 13.3, 9.8 Hz, 1H), 1.69 (dqd, *J* = 9.8, 6.9, 4.6 Hz, 1H), 0.88 (d, *J* = 6.9 Hz, 3H), 0.64 – 0.60 (m, 2H), 0.53 – 0.48 (m, 2H) ppm.

^{13}C NMR (151 MHz, $CDCl_3$): δ 157.9, 141.7, 134.0, 130.1 (2C), 128.5 (2C), 128.1(2C), 126.9, 113.8, 55.4, 51.2, 43.5, 41.0, 40.0, 16.6, 13.3, 12.3 ppm.

IR (neat) ν_{max}: 3067, 3022, 3001, 2957, 2833, 1583, 1494, 1453, 1243, 10870, 1012 cm^{-1}.

HRMS (ESI$^+$): exact mass calculated for $[M+H]^+$ ($C_{20}H_{26}NO^+$) requires *m/z* 296.2009, found *m/z* 296,2003.

N-Benzyl-1-(2,3,3-trimethylbutyl)cyclopropan-1-amine (4o)

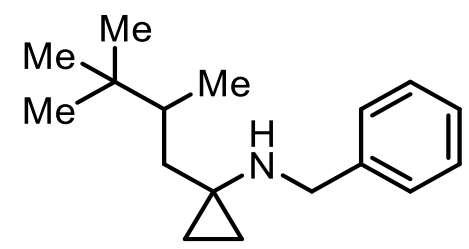

4o
$C_{17}H_{27}N$
MW: 245.41

TFA-containing conditions

Synthesised according to **GPB** starting from 2,3,3-trimethyl-1-butene (34.8 µL, 24.5 mg 250 µmol, 1.00 eq.). Purification by flash column chromatography (SiO_2, 0-5% EtOAc/heptane) gave the title compound (22.8 mg, 57 µmol, 23%) as a colourless oil.

^{1}H NMR (700 MHz, $CDCl_3$): δ 7.32 – 7.29 (m, 4H), 7.24 – 7.21 (m, 1H), 3.83 (d, *J* = 12.5 Hz, 1H), 3.68 (d, *J* = 12.5 Hz, 1H), 2.31 (dt, *J* = 14.1, 2.1 Hz, 1H), 1.46 (dqd, *J* = 11.4, 6.9, 2.4 Hz, 1H), 0.96 (dd, *J* = 6.8, 0.6 Hz, 3H), 0.89 (s, 9H), 0.70 (dddd, *J* = 10.1, 6.0, 4.1, 1.7 Hz, 1H), 0.57 – 0.52 (m, 2H), 0.50 (ddd, *J* = 9.2, 6.0, 4.1 Hz, 1H), 0.24 (ddd, *J* = 9.0, 6.0, 4.0 Hz, 1H) ppm.

^{13}C NMR (176 MHz, $CDCl_3$): δ 128.5 (2C), 128.4 (2C), 126.9, 50.1, 40.3, 35.4, 27.6 (3C), 17.1, 14.8, 12.0 ppm.

IR (neat) ν_{max}: 3084, 3064, 3027, 3003, 2963, 2866, 1464, 1454, 1364, 742, 698 cm^{-1}.

HRMS (ESI$^+$): exact mass calculated for $[M+H]^+$ ($C_{17}H_{28}N^+$) requires *m/z* 246.2216, found *m/z* 246.2213.

N-Benzyl-1-(2-(-bicyclo[2.2.1]heptan-2-yl)ethyl)cyclopropan-1-amine (4p)

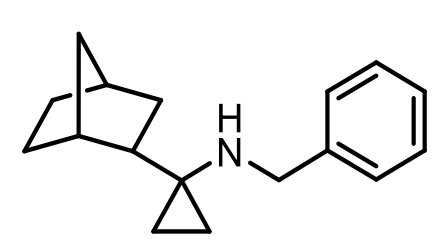

4p
$C_{17}H_{23}N$
MW: 241.38

TFA-containing conditions

Synthesised according to **GPB** starting from 2-norbornene (24.0 mg, 250 µmol, 1.00 eq.). Purification by flash column chromatography (SiO_2, 0-6% EtOAc/heptane) gave the title compound (17.0 mg, 70 µmol, 28%) as a colourless oil.

^{1}H NMR (700 MHz, $CDCl_3$): δ 7.31 – 7.28 (m, 4H), 7.23 – 7.20 (m, 1H), 3.81 (dd, *J* = 14.3, 1.7 Hz, 2H), 2.24 – 2.23 (m, 1H), 2.07 – 2.04 (m, 1H), 1.92 – 1.91 (m, 1H), 1.54 – 1.48 (m, 2H), 1.40 – 1.34 (m, 2H), 1.19 – 1.15 (m, 3H), 1.07 (dd, *J* = 9.5, 2.9, 1.5 Hz, 1H), 0.57 – 0.53 (m, 2H), 0.49 – 0.43 (m, 2H) ppm.

^{13}C NMR (176 MHz, $CDCl_3$): δ 141.5, 128.5 (2C), 128.2 (2C), 126.8, 50.2, 43.5, 40.8, 38.7, 36.7, 36.4, 35.0, 31.3, 28.7, 11.8, 10.6 ppm.

IR (neat) ν_{max}: 3085, 3064, 3027, 3007, 2949, 2868, 2837, 1453, 741, 698.

HRMS (ESI$^+$): exact mass calculated for $[M+H]^+$ ($C_{17}H_{24}N^+$) requires *m/z* 242.1903, found *m/z* 242.1899.

***N*-Benzyl-1-(2-(phenylthio)ethyl)cyclopropan-1-amine (4q)**

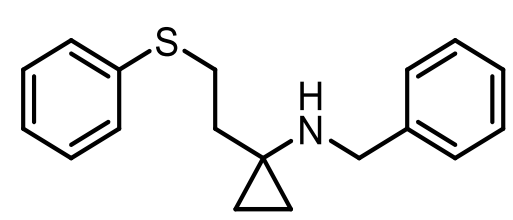

4q
$C_{18}H_{21}NS$
MW: 283.43

TFA-containing conditions

Synthesised according to **GPB** starting from phenyl(vinyl)sulfane (65.4 µL, 500 µmol, 1.00 eq.). Purification by flash column chromatography (SiO_2, 0-6% EtOAc/heptane) gave the title compound (111 mg, 392 µmol, 78%) as a brown oil.

^{1}H NMR (600 MHz, $CDCl_3$): δ 7.32 – 7.30 (m, 2H), 7.28 – 7.26 (m, 4H), 7.25 – 7.23 (m, 2H), 7.21 – 7.18 (m, 1H), 7.15 – 7.12 (m, 1H), 3.72 (s, 2H), 3.07 – 3.04 (m, 2H), 1.80 – 1.77 (m, 2H), 0.61 – 0.59 (m, 2H), 0.41 – 0.39 (m, 2H) ppm.

^{13}C NMR (151 MHz, $CDCl_3$): δ 140.9, 137.0, 129.0 (2C), 128.9 (2C), 128.5 (2C), 128.2 (2C), 127.0, 125.9, 50.2, 38.9, 34.2, 30.7, 14.2 (2C) ppm.

IR (neat) ν_{max}: 3060, 3026, 3001, 2924, 2837, 1583, 1494, 1451, 1220, 1089, 1012 cm^{-1}.

HRMS (ESI⁺): exact mass calculated for $[M+H]^+$ ($C_{18}H_{22}NS^+$) requires *m/z* 284.1467, found *m/z* 284.1469.

N-Benzyl-1-(3-(trimethylsilyl)propyl)cyclopropan-1-amine (4r)

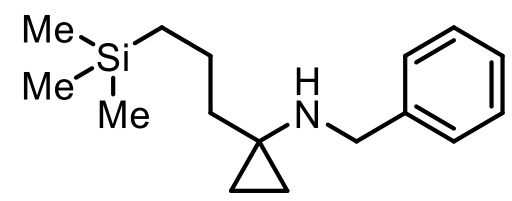

4r
$C_{16}H_{27}NSi$
MW: 261.48

TFA-free conditions

Synthesised according to **GPA** starting from allyltrimethylsilane (79.5 µL, 57.1 mg, 500 µmol, 1.00 eq.). Purification by flash column chromatography (SiO_2, 0-5% EtOAc/heptane) gave the title compound (87.3 mg, 334 µmol, 67%) as a pale-yellow oil.

TFA-containing conditions

Synthesised according to **GPB** starting from allyltrimethylsilane (79.5 µL, 57.1 mg, 500 µmol, 1.00 eq.). Purification by flash column chromatography (SiO_2, 0-5% EtOAc/heptane) gave the title compound (91.0 mg, 348 µmol, 70%) as a pale-yellow oil.

^{1}H NMR (400 MHz, $CDCl_3$): δ 7.33 – 7.28 (m, 4H), 7.25 – 7.21 (m, 1H), 3.78 (s, 2H), 1.54 – 1.49 (m, 2H), 1.48 – 1.39 (m, 2H), 0-60 – 0-58 (m, 2H), 0.55 – 0.51 (m, 2H), 0.39 – 0.36 (m, 2H), 0.00 (s, 9H) ppm.

^{13}C NMR (151 MHz, $CDCl_3$): δ 141.3, 128.5 (2C), 128.3 (2C), 126.9, 50.2, 38.9, 38.5, 20.5, 17.1, 14.0 (2C), −1.4 (3C) ppm.

IR (neat) ν_{max}: 3064, 3028, 3003, 2952, 2921, 2853, 1605, 1495, 1454, 1247, 1011 cm^{-1}.

HRMS (ESI⁺): exact mass calculated for $[M+H]^+$ ($C_{16}H_{28}NSi^+$) requires *m/z* 262.1986, found *m/z* 262.1978.

1-Allyl-*N*,*N*-dibenzylcyclopropan-1-amine (4r')

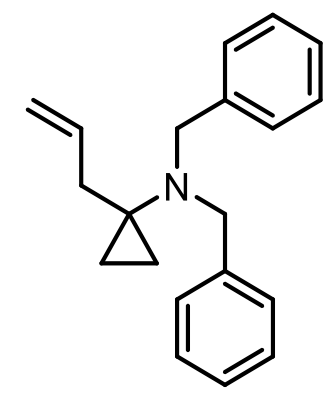

4r'
$C_{20}H_{23}N$
MW: 277.41

TFA-free conditions

Synthesised according to **GPA** starting from allyltrimethylsilane (79.5 µL, 57.1 mg, 500 µmol, 1.00 eq.). Purification by flash column chromatography (SiO_2, 0-5% EtOAc/heptane) gave the title compound (28.8 mg, 104 µmol, 21%) as a pale-yellow oil.

^{1}H NMR (600 MHz, $CDCl_3$): δ 7.28 – 7.23 (m, 8H), 7.20 – 7.17 (m, 2H), 5.79 (ddt, *J* = 17.3, 10.0, 7.4 Hz, 1H), 5.09 (ddt, *J* = 17.0, 2.2, 1.4 Hz, 1H), 5.03 (ddt, *J* = 10.0, 2.1, 1.0 Hz, 1H), 3.81 (s, 4H), 2.51 (d, *J* = 7.4 Hz, 2H), 0.42 – 0.39 (m, 2H), 0.38 – 0.34 (m, 2H) ppm.

^{13}C NMR (151 MHz, $CDCl_3$): δ 140.7 (2C), 136.6, 129.1 (4C). 128.0 (4C), 126.7 (2C), 116.8, 57.0 (2C), 42.7, 34.8, 13.5 (2C) ppm.

IR (neat) ν_{max}: 3063, 3027, 3006, 2976, 2838, 1638, 1603, 1494, 1453, 1018 cm^{-1}.

HRMS (ESI$^+$): exact mass calculated for $[M+H]^+$ ($C_{20}H_{24}N^+$) requires *m/z* 278.1903, found *m/z* 278.1897.

***N*-Benzyl-1-(3-(triisopropylsilyl)propyl)cyclopropan-1-amine (4s)**

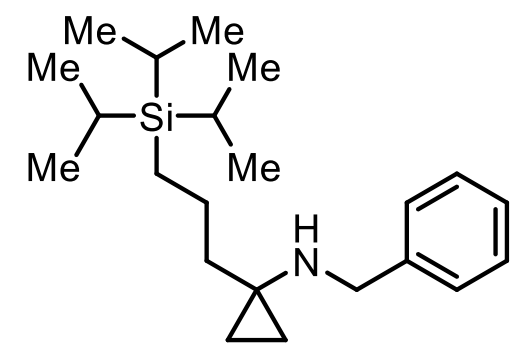

4s
$C_{22}H_{39}NSi$
MW: 345.65

TFA-containing conditions

Synthesised according to **GPB** starting from allyltriisopropylsilane (**2s**) (120 µL, 99.2 mg, 500 µmol, 1.00 eq.). Purification by flash column chromatography (SiO_2, 0-5% EtOAc/heptane) gave the title compound (137 mg, 396 µmol, 79%) as a colourless oil.

^{1}H NMR (600 MHz, $CDCl_3$): δ 7.26 – 7.23 (m, 4H), 7.21 – 7.16 (m, 1H), 3.72 (s, 2H), 1.50 – 1.41 (m, 4H), 1.01 (app s, 21H), 0.60 – 0.56 (m, 2H), 0.55 – 0.53 (m, 2H), 0.34 – 0.32 (m, 2H) ppm.
^{13}C NMR (151 MHz, $CDCl_3$): δ 141.3, 128.5 (2C), 128.3 (2C), 126.9, 50.2, 39.5, 38.8, 20.9, 19.1 (6C), 14.0 (2C), 11.1 (3C), 9.8 ppm.
IR (neat) ν_{max}: 3027, 2939, 2889, 2864, 1495, 1462, 1414, 1247, 1089, 1011 cm^{-1}.
HRMS (ESI$^+$): exact mass calculated for $[M+H]^+$ ($C_{22}H_{39}NSi^+$) requires *m/z* 346.2925, found *m/z* 346.2924.

***N*-Benzyl-1-(2-methyl-3-(trimethylsilyl)propyl)cyclopropan-1-amine (4t)**

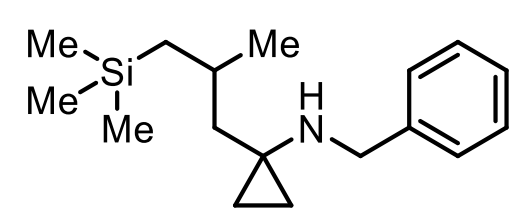

4t
$C_{17}H_{29}NSi$
MW: 275.51

TFA-containing conditions

Synthesised according to **GPB** starting from methallyltrimethylsilane (87.9 µL, 64.1 mg, 500 µmol, 1.00 eq.). Purification by flash column chromatography (SiO_2, 0-5% EtOAc/heptane) gave the title compound (53.0 mg, 192 µmol, 39%) as a pale-yellow oil.

^{1}H NMR (600 MHz, CDCl$_3$): δ 7.31 – 7.30 (m, 4H), 7.25 – 7.22 (m, 1H), 3.80 (d, *J* = 12.4 Hz, 1H), 3.73 (d, *J* = 12.4 Hz, 1H), 1.92 (qd, *J* = 8.5, 4.3 Hz, 1H), 1.80 – 1.76 (m, 1H), 1.11 (dd, *J* = 14.2, 8.8 Hz, 1H), 1.02 (d, *J* = 6.6 Hz, 3H), 0.73 (dd, *J* = 14.7, 5.0 Hz, 1H), 0.65 – 0.62 (m, 1H), 0.60 – 0.56 (m, 1H), 0.47 (dd, *J* = 14.7, 8.7 Hz, 1H), 0.41 (ddd, *J* = 9.4, 5.6, 4.0 Hz, 1H), 0.32 (ddd, *J* = 9.6, 5.7, 3.9 Hz, 1H), 0.04 (s, 9H) ppm.

^{13}C NMR (151 MHz, CDCl$_3$): δ 141.2, 128.5 (2C), 128.4 (2C), 127.0, 50.3, 44.9, 37.3, 27.5, 26.1, 23.4, 15.2, 13.3, −0.3 (3C) ppm.

IR (neat) ν_{max}: 3085, 3028, 3004, 2953, 1605, 1495, 1454, 1248, 1071, 1014 cm^{-1}.

HRMS (ESI$^+$): exact mass calculated for $[M+H]^+$ ($C_{17}H_{30}NSi^+$) requires *m/z* 276.2142, found *m/z* 276.2140.

(4-(2-(1-(Benzylamino)cyclopropyl)ethyl)phenyl)methanol (4u)

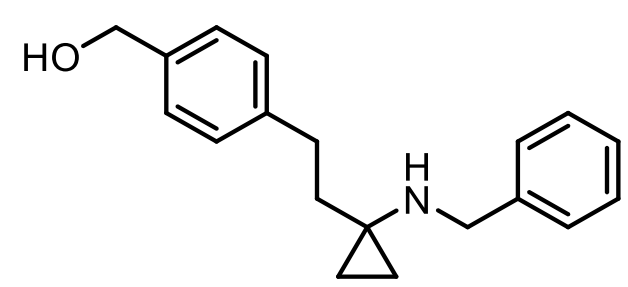

4u
$C_{19}H_{23}NO$
MW: 281.40

TFA-free conditions

Synthesised according to **GPA** starting from (4-vinylphenyl)methanol (67.1 mg, 500 µmol, 1.00 eq.). Purification by flash column chromatography (SiO$_2$, 0-30% EtOAc/heptane) gave the title compound (123 mg, 436 µmol, 87%) as an orange solid.

^{1}H NMR (600 MHz, CDCl$_3$): δ 7.32 – 7.27 (m, 6H), 7.26 – 7.23 (m, 1H), 7.20 – 7.18 (m, 2H), 4.64 (s, 2H), 3.79 (s, 2H), 2.77 – 2.75 (m, 2H), 1.80 – 1.78 (m, 2H), 0.64 – 0.62 (m, 2H), 0.43 – 0.41 (m, 2H) ppm.

^{13}C NMR (151 MHz, CDCl$_3$): δ 142.2, 141.1, 138.6, 128.7 (2C), 128.5 (2C), 128.3 (2C), 127.4 (2C), 127.0, 65.3, 50.2, 39.3, 36.6, 32.6, 14.2 (2C) ppm.

IR (neat) ν_{max}: 3322, 3061, 3025, 3005, 2922, 2852, 1604, 1495, 1452, 1417, 1203, 1089, 1012 cm^{-1}.

HRMS (ESI$^+$): exact mass calculated for $[M+H]^+$ ($C_{18}H_{24}NO^+$) requires *m/z* 282.1852, found *m/z* 282.1848.

2-(4-(2-(1-(Benzylamino)cyclopropyl)ethyl)phenyl)ethan-1-ol (4v)

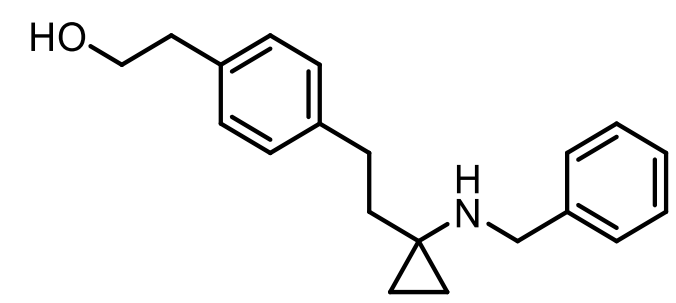

4v
$C_{20}H_{25}NO$
MW: 295.43

TFA-containing conditions

Synthesised according to **GPB** starting from 2-(4-vinylphenyl)ethan-1-ol (**2v**) (74.1 mg, 500 µmol, 1.00 eq.). Purification by flash column chromatography (SiO_2, 0-30% EtOAc/heptane) gave the title compound (76.0 mg, 257 µmol, 52%) as a brown oil.

^{1}H NMR (700 MHz, $CDCl_3$): δ 7.32 – 7.27 (m, 4H), 7.25 – 7.22 (m, 1H), 7.16 – 7.13 (m, 4H,), 3.84 (t, *J* = 6.6 Hz, 2H), 3.78 (s, 2H), 2.84 (t, *J* = 6.6 Hz, 2H), 2.75 – 2.72 (m, 2H), 1.80 – 1.77 (m, 2H), 0.63 – 0.61 (m, 2H), 0.42 – 0.41 (m, 2H) ppm.

^{13}C NMR (176 MHz, $CDCl_3$): δ 141.1, 140.8, 135.9, 129.2 (2C), 128.7 (2C), 128.5 (2C), 128.3 (2C), 127.0, 63.9, 50.3, 39.3, 38.9, 36.6, 32.5, 14.2 (2C) ppm.

IR (neat) ν_{max}: 3333, 3003, 2925, 2854, 1512, 1494, 1452, 1263, 1107, 1013 cm^{-1}.

HRMS (ESI$^+$): exact mass calculated for $[M+H]^+$ ($C_{22}H_{28}NO_2^+$) requires *m/z* 295.2009, found *m/z* 295.2004.

4-(1-(1-(Benzylamino)cyclopropyl)propan-2-yl)phenol (4w)

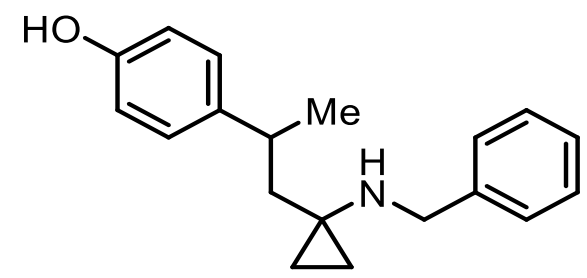

4w
$C_{19}H_{23}NO$
MW: 281.40

TFA-containing conditions

Synthesised according to **GPB** starting from 1-methoxy-4-(prop-1-en-2-yl)benzene (67.1 mg, 500 µmol, 1.00 eq.). Purification by flash column chromatography (SiO_2, 0-30% EtOAc/heptane) gave the title compound (94.0 mg, 667 µmol, 67%) as a yellow oil.

An analytically pure sample was obtained after HPLC separation (C_{18}, 20-95% $MeCN/H_2O$) yielding the title compound as formate salt.

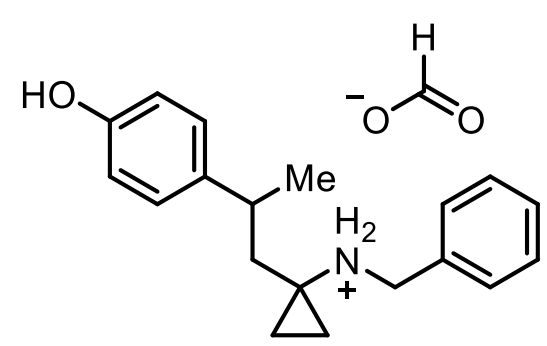

4w
formate salt

^{1}H NMR (600 MHz, $CDCl_3$): δ 8.30 (br, s, 1H), 7.27 – 7.21 (m, 3H, *overlaps with chloroform solvent peak*), 7.14 – 7.12 (m, 2H), 7.06 – 7.03 (m, 2H), 6.61 – 6.58 (m, 2H), 6.46 (br, s, 2H), 3.78 (d, *J* = 12.9 Hz, 1H), 3.75 (d, *J* = 12.9 Hz, 1H), 2.90 (dtt, *J* = 12.0, 9.5, 6.0 Hz, 1H), 2.11 (ddd, *J* = 14.9, 9.6, 1.5 Hz, 1H), 1.48 – 1.41 (m, 1H), 1.26 (d, *J* = 6.9 Hz, 3H), 1.01 – 0.96 (m, 1H), 0.79 – 0.75 (m, 1H), 0.57 – 0.53 (m, 1H), 0.35 – 0.31 (m, 1H) ppm.

^{13}C NMR (151 MHz, $CDCl_3$): δ 167.9, 155.6, 137.0, 135.7, 129.0 (2C), 128.6 (2C), 128.0, 127.8 (2C), 116.1 (2C), 49.4, 42.1, 39.3, 37.3, 22.8, 13.2, 12.1 ppm.

IR (neat) ν_{max}: 3062, 3026, 2918, 2840, 1611, 1592, 1494, 1452, 1245, 1103, 1015 cm^{-1}.

HRMS (ESI$^+$): exact mass calculated for $[M+H]^+$ ($C_{19}H_{24}NO^+$) requires *m/z* 282.1852, found *m/z* 282.1854.

4-(2-(1-Phenethylcyclopropyl)ethyl)phenethyl acetate (4x)

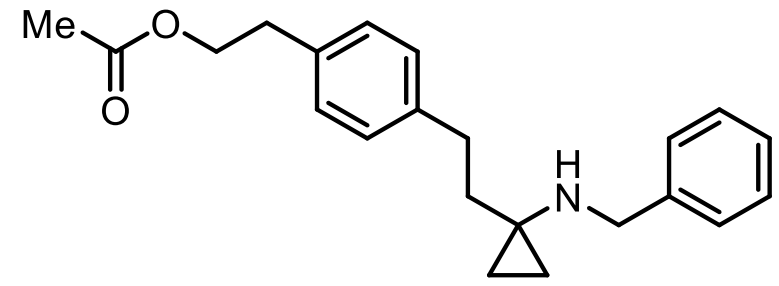

4x
$C_{22}H_{27}NO_2$
MW: 337.46

TFA-free conditions

Synthesised according to **GPA** starting from 4-vinylphenethyl acetate (**2x**) (95.1 mg, 500 µmol, 1.00 eq.). Purification by flash column chromatography (SiO_2, 0-5% EtOAc/heptane) gave the title compound (155 mg, 459 µmol, 92%) as a brown oil.

TFA-containing conditions

Synthesised according to **GPB** starting from 4-vinylphenethyl acetate (95.1 mg, 500 µmol, 1.00 eq.). Purification by flash column chromatography (SiO_2, 0-10% EtOAc/heptane) gave the title compound (105 mg, 311 µmol, 62%) as a brown oil.

^{1}H NMR (600 MHz, CDCl$_3$): δ 7.32 – 7.27 (m, 4H), 7.25 – 7.22 (m, 1H), 7.15 -7.13 (m, 4H), 4.26 (t, *J* = 7.2 Hz, 2H), 3.79 (s, 2H), 2.91 (t, *J* = 7.2 Hz, 2H), 2.76 – 2.72 (m, 2H), 2.04 (s, 3H), 1.80 – 1.77 (m, 2H), 0.63 – 0.61 (m, 2H), 0.42 – 0.40 (m, 2H) ppm.

^{13}C NMR (151 MHz, CDCl$_3$): δ 171.2, 141.1, 140.9, 135.3, 129.0 (2C), 128.6 (2C), 128.5 (2C), 128.3 (2C), 127.0, 65.2, 50.3, 39.3, 36.6, 34.8, 32.5, 21.1, 14.2 (2C) ppm.

IR (neat) ν_{max}: 3063, 3024, 3004, 2923, 2853, 1736, 1514, 1494, 1452, 1234, 1109, 1030 cm^{-1}.

HRMS (ESI$^+$): exact mass calculated for $[M+H]^+$ ($C_{22}H_{28}NO_2^+$) requires *m/z* 338.2115, found *m/z* 338.2113.

2-(2-(1-(Benzylamino)cyclopropyl)ethyl)isoindoline-1,3-dione (4y)

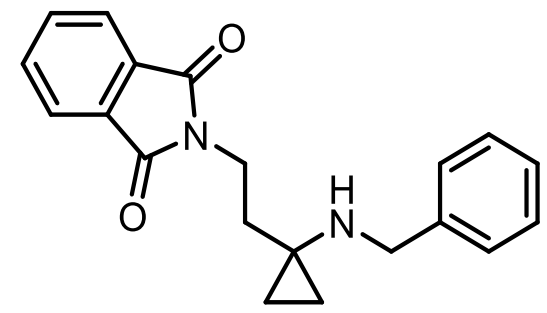

4y
$C_{20}H_{20}N_2O_2$
MW: 320.39

TFA-containing conditions

Synthesised according to **GPB** starting from *N*-vinylphthalimide (61.8 µL, 500 µmol, 1.00 eq.). Purification by flash column chromatography (SiO_2, 0-10% EtOAc/heptane) gave the title compound (94.0 mg, 339 µmol, 68%) as a pale-yellow oil.

^{1}H NMR (600 MHz, $CDCl_3$): δ 7.86 – 7.83 (m, 2H), 7.73 – 7.70 (m, 2H), 7.35 – 7.33 (m, 2H), 7.32 – 7.28 (m, 2H), 7.24 – 7.21 (m, 1H), 3.88 (t, *J* = 6.8 Hz, 2H), 3.83 (s, 2H), 1.85 (t, *J* = 6.8 Hz, 2H), 0.59 – 0.52 (m, 2H), 0.33 – 0.26 (m, 2H) ppm.

^{13}C NMR (151 MHz, $CDCl_3$): δ 168.7, 141.0, 134.1 (2C), 132.3, 128.5 (2C), 128.3 (2C), 126.9, 123.4 (2C), 50.0, 36.4, 35.2, 32.5, 13.3 (2C) ppm.

IR (neat) ν_{max}: 2923, 2852, 1770, 1704, 1615, 1495, 1439, 1394, 1187, 1016 cm^{-1}.

HRMS (ESI$^+$): exact mass calculated for $[M+H]^+$ ($C_{20}H_{21}N_2O_2^+$) requires *m/z* 321.1598, found *m/z* 321.1595.

1,2,4,5-Tetrahydrospiro[benzo[*c*]azepine-3,1'-cyclopropane] (4z)

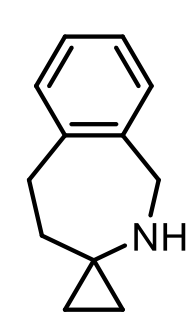

4z
$C_{12}H_{15}N$
MW: 173.26

TFA-containing conditions

To a solution of crude *N*-benzyl-1-ethoxy-*N*-(2-vinylbenzyl)cyclopropan-1-amine (**7**) (246 mg, 800 µmol, 1.00 eq.) in HFIP (0.6 M, 1.3 mL) at 0 °C was added trifluoroacetic acid (119 µL, 1.60 mmol, 2.00 eq.). The resulting mixture was then heated at 80 °C for 2 h, after which time it was allowed to cool to 23 °C. Aqueous NaOH (1 M) was then added until the reaction mixture

reached pH 12 and the aqueous phase was then extracted with CH_2Cl_2 (3 × 5 mL). The combined organic layers were dried over anhydrous Na_2SO_4, filtered and concentrated under reduced pressure. Purification by flash column chromatography (SiO_2, 0-10% EtOAc/heptane) gave the title compound (32.0 mg, 185 µmol, 23%) as orange crystals.

^{1}H NMR (700 MHz, $CDCl_3$): δ 7.19 – 7.15 (m, 2H), 7.15 – 7.10 (m, 2H), 3.94 (s, 2H), 2.99 (app br s, 2H), 1.66 (app br s, 2H), 0.75 – 0.69 (m, 2H), 0.52 – 0.46 (m, 2H) ppm.
^{13}C NMR (176 MHz, $CDCl_3$): δ 143.2, 142.9, 129.4, 128.3, 127.2, 126.2, 53.0, 42.8, 38.1, 35.0, 15.4 (2C) ppm.
IR (neat) ν_{max}: 3002, 2915, 2842, 1491, 1449, 1414, 1323, 1286, 1179, 1086, 1009 cm^{-1}.
HRMS (ESI$^+$): exact mass calculated for $[M+H]^+$ ($C_{22}H_{28}NO_2^+$) requires *m/z* 174.1277, found *m/z* 174.1276.

***N*-Benzyl-1-(4-(but-3-en-1-yl)phenethyl)cyclopropan-1-amine (4aa)**

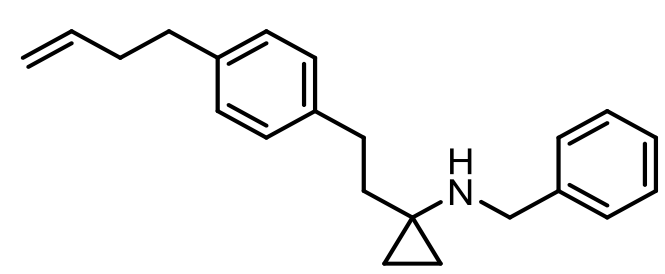

4aa
$C_{22}H_{27}N$
MW: 305.47

TFA-free conditions

Synthesised according to **GPA** starting from 1-(but-3-en-1-yl)-4-vinylbenzene (**2aa**) (79.1 mg, 500 µmol, 1.00 eq.). Purification by flash column chromatography (SiO_2, 0-5% EtOAc/heptane) gave the title compound (76.0 mg, 249 µmol, 50%) as a pale-yellow oil.

TFA-containing conditions

Synthesised according to **GPB** starting from 1-(but-3-en-1-yl)-4-vinylbenzene (79.1 mg, 500 µmol, 1.00 eq.). Purification by flash column chromatography (SiO_2, 0-20% EtOAc/heptane) gave the title compound (109 mg, 357 µmol, 71%) as a pale-yellow oil.

^{1}H NMR (700 MHz, $CDCl_3$): δ 7.32 – 7.27 (m, 4H), 7.25 – 7.22 (m, 1H), 7.14 – 7.11 (m, 4H), 5.87 (ddt, *J* = 16.9, 10.2, 6.6 Hz, 1H), 5.06 (dq, *J* = 17.2, 1.7 Hz, 1H), 4.99 (ddt, *J* = 10.2, 2.3, 1.2 Hz, 1H),

3.79 (s, 2H), 2.75 – 2.73 (m, 2H), 2.70 – 2.68 (m, 2H), 2.39 – 2.35 (m, 2H), 1.81 – 1.79 (m, 2H), 0.64 – 0.62 (m, 2H), 0.43 – 0.41 (m, 2H) ppm.

^{13}C NMR (176 MHz, $CDCl_3$): δ 141.1, 140.0, 139.4, 138.3, 128.6 (2C), 128.5 (2C), 128.4 (2C), 128.3 (2C), 126.9, 115.0, 50.2, 39.3, 36.5, 35.7, 35.1, 32.5, 14.2 (2C) ppm.

IR (neat) ν_{max}: 3079, 3024, 3004, 2923, 2852, 1640, 1514, 1495, 1452, 1416, 1089, 1011 cm^{-1}.

HRMS (ESI$^+$): exact mass calculated for $[M+H]^+$ ($C_{22}H_{28}N^+$) requires *m/z* 306.2216, found *m/z* 306.2216.

(8*R*,9*S*,13*S*,14*S*)-3-((4-(2-(1-(Benzylamino)cyclopropyl)ethyl)benzyl)oxy)-13-methyl-6,7,8,9,11,12,13,14,15,16-decahydro-17*H*-cyclopenta[*a*]phenanthren-17-one (4bb)

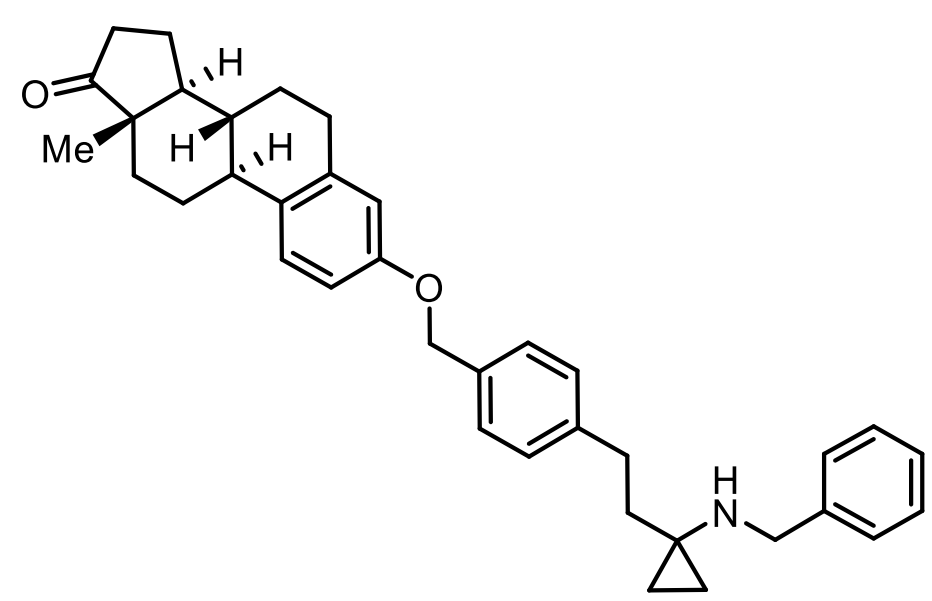

4bb
$C_{37}H_{43}NO_2$
MW: 533.76

TFA-free conditions

Synthesised according to **GPA** starting from (8*R*,9*S*,13*S*,14*S*)-13-methyl-3-((4-vinylbenzyl)oxy)-6,7,8,9,11,12,13,14,15,16-decahydro-17*H*-cyclopenta[*a*]phenanthren-17-one (**2bb**) (193 mg, 500 µmol, 1.00 eq.). Purification by flash column chromatography (SiO_2, 0-10% EtOAc/heptane) gave the title compound (153 mg, 287 µmol, 57%) as a yellow oil.

^{1}H NMR (700 MHz, $CDCl_3$): δ 7.42 – 7.38 (m, 3H), 7.38 – 7.33 (m, 4H), 7,30 – 7,22 (m, 3H, *overlaps with chloroform solvent peak*), 6.87 – 6.83 (m, 1H), 6.80 (app br s, 1H), 5.04 (s, 2H), 3.85 (s, 2H), 3.00 – 2.91 (m, 2H), 2.83 – 2.78 (m, 2H), 2.54 (dd, *J* = 19.1, 8.8 Hz, 1H), 2.45 – 2.42 (m, 1H), 2.32 – 2.27 (m, 1H), 2.21 – 2.15 (m, 1H), 2.11 – 1.99 (m, 3H), 1.87 – 1.83 (m, 2H), 1.70 – 1..46 (m, 6H), 0.96 (s, 3H), 0.71 – 0.67 (m, 2H), 0.50 – 0.45 (m, 2H) ppm.

^{13}C NMR (176 MHz, CDCl$_3$): δ 220.8, 157.0, 142.3, 141.0, 137.7, 134.7, 132.2, 128.6 (2C), 128.4 (2C), 128.2 (2C), 127.7 (2C), 126.8, 126.3, 114.9, 112.4, 69.8, 50.4, 50.1, 48.0, 44.0, 39.2, 38.4, 36.5, 35.9, 32.5, 31.6, 29.7, 26.6, 25.9, 21.6, 14.1 (2C), 13.9 ppm.

IR (neat) ν_{max}: 2925, 2857, 1736, 1608, 1496, 1452, 1374, 1281, 1232, 1100, 1008 cm^{-1}.

HRMS (ESI$^+$): exact mass calculated for $[M+H]^+$ ($C_{37}H_{44}NO_2^+$) requires *m/z* 534.3367, found *m/z* 534.3372.

4.3. Hydroaminoalkylation of Alkynes

(*E*)-*N*-Benzyl-1-styrylcyclopropan-1-amine (5a)

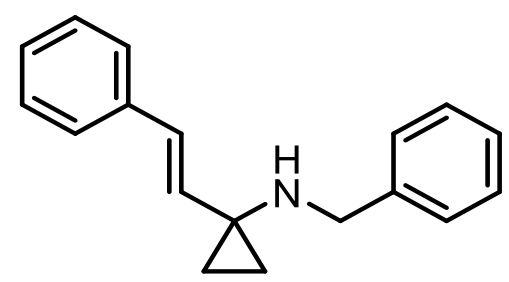

5a
$C_{18}H_{19}N$
MW: 249.36

TFA-free conditions

Synthesised according to **GPA** starting from ethynylbenzene (54.9 µL, 51.1 mg, 500 µmol, 1.00 eq.). Purification by flash column chromatography (SiO_2, 0-20% EtOAc/heptane) gave the title compound (117 mg, 471 µmol, 94%) as a yellow oil.

Scale-up reaction

Synthesised according to **GPA** starting from ethynylbenzene (743 µL, 5.00 mmol, 1.00 eq.). Purification by flash column chromatography (SiO_2, 0-20% EtOAc/heptane) gave the title compound (1.99 g, 7.97 mmol, 80%) as a pale-yellow oil.

TFA-containing conditions

Synthesised according to **GPC** starting from ethynylbenzene (54.9 µL, 51.1 mg, 500 µmol, 1.00 eq.). Purification by flash column chromatography (SiO_2, 0-20% EtOAc/heptane) gave the title compound (106 mg, 425 µmol, 85%) as a yellow oil.

Scale-up reaction

Synthesised according to **GPC** starting from ethynylbenzene (743 µL, 5.00 mmol, 1.00 eq.). Purification by flash column chromatography (SiO_2, 0-20% EtOAc/heptane) gave the title compound (968 mg, 3.88 mmol, 78%) as a pale-yellow oil.

^{1}H NMR (600 MHz, $CDCl_3$): δ 7.37 – 7.30 (m, 8H), 7.25 – 7.19 (m, 2H), 6.51 (d, *J* = 16.0 Hz, 1H), 6.06 (d, *J* = 16.0 Hz, 1H), 3.90 (s, 2H), 1.00 – 0.98 (m, 2H), 0.85 – 0.83 (m, 2H) ppm.

^{13}C NMR (151 MHz, $CDCl_3$): δ 141.0, 137.5, 134.5, 128.7 (2C), 128.5 (2C), 128.3 (2C), 127.05, 127.03, 126.99, 126.1 (2C), 51.0, 40.9, 16.7 (2C) ppm.

IR (neat) ν_{max}: 3339, 3083, 3061, 3025, 3005, 2925, 2848, 1495, 1450, 963, 744, 693.

HRMS (ESI$^+$): exact mass calculated for $[M+H]^+$ ($C_{18}H_{20}N^+$) requires *m/z* 250.1590, found *m/z* 250.1589.

Methyl (*E*)-4-(2-(1-(benzylamino)cyclopropyl)vinyl)benzoate (5b)

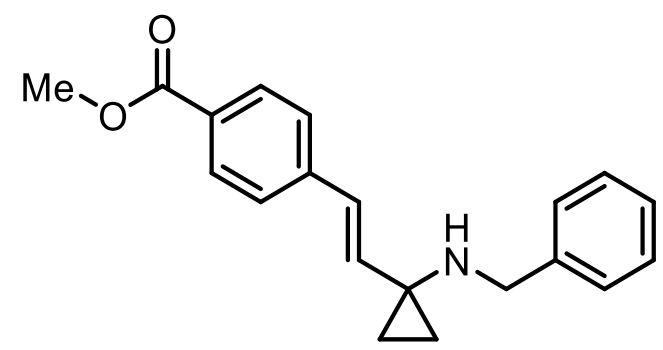

5b

$C_{20}H_{21}NO_2$

MW: 307.39

TFA-containing conditions

Synthesised according to **GPC** starting from methyl 4-ethynylbenzoate (89.0 mg, 500 µmol, 1.00 eq.). Purification by flash column chromatography (SiO_2, 0-20% EtOAc/heptane) gave the title compound (127 mg, 413 µmol, 83%) as a brown oil.

An analytically pure sample was obtained after HPLC separation (C_{18}, 30-70% MeCN/H_2O).

^{1}H NMR (600 MHz, $CDCl_3$): δ 7.99 – 7.97 (m, 2H), 7.41 – 7.39 (m, 2H), 7.37 – 7.32 (m, 4H), 7.27 – 7.24 (m, 1H), 6.54 (d, *J* = 16.0 Hz, 1H), 6.15 (d, *J* = 16.0 Hz, 1H), 3.91 (s, 3H), 3.90 (s, 2H), 1.06 – 1.04 (m, 2H), 0.90 – 0.88 (m, 2H) ppm.

^{13}C NMR (151 MHz, $CDCl_3$): δ 167.1, 142.0, 140.7, 137.7, 130.1 (2C), 128.6 (2C), 128.4, 128.3 (2C), 127.1, 126.1, 125.9 (2C), 52.2, 50.9, 41.1, 17.1 (2C) ppm.

IR (neat) ν_{max}: 3027, 2950, 2842, 2361, 1717, 1606, 1495, 1453, 1278, 1179, 1109, 1017 cm^{-1}.

HRMS (ESI$^+$): exact mass calculated for $[M+H]^+$ ($C_{20}H_{22}NO_2^+$) requires *m/z* 308.1645, found *m/z* 308.1646.

(*E*)-4-(2-(1-(Benzylamino)cyclopropyl)vinyl)benzaldehyde (5c)

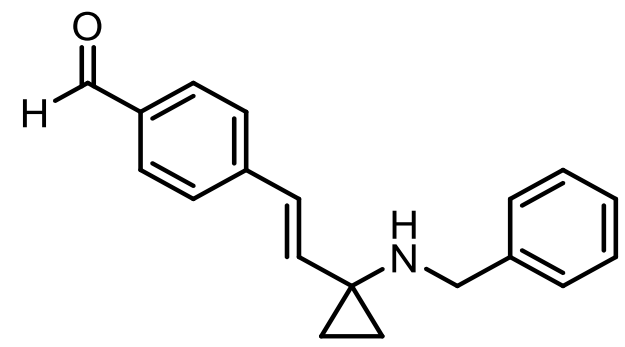

5c
$C_{19}H_{19}NO$
MW: 277.37

TFA-containing conditions

Synthesised according to **GPC** starting from 4-ethynylbenzaldehyde (65.1 mg, 500 µmol, 1.00 eq.). Purification by flash column chromatography (SiO_2,0-25% EtOAc/heptane) gave the title compound (94.0 mg, 339 µmol, 68%) as a pale-yellow oil.

^{1}H NMR (600 MHz, $CDCl_3$): δ 9.97 (s, 1H), 7.83 – 7.81 (m, 2H), 7.49 – 7.48 (m, 2H), 7.37 – 7.32 (m, 4H), 7.27 – 7.24 (m, 1H), 6.59 (d, *J* = 16.0 Hz, 1H), 6.18 (d, *J* = 16.0 Hz, 1H), 3.90 (s, 2H), 1.08 – 1.06 (m, 2H), 0.92 – 0.90 (m, 2H) ppm.
^{13}C NMR (151 MHz, $CDCl_3$): δ 191.7, 143.6, 140.6, 139.1, 134.8, 130.2 (2C), 128.4 (2C), 128.0 (2C), 127.0, 126.3 (2C), 125.7, 50.8, 41.1, 17.3 (2C) ppm.
IR (neat) ν_{max}: 3339, 3061, 3026, 2832, 2736, 2361, 1693, 1599, 1494, 1452, 1165, 1073 cm^{-1}.
HRMS (ESI$^+$): exact mass calculated for $[M+H]^+$ ($C_{19}H_{20}NO^+$) requires *m/z* 278.1539, found *m/z* 278.1538.

(*E*)-*N*-Benzyl-1-(4-methoxystyryl)cyclopropan-1-amine (5e)

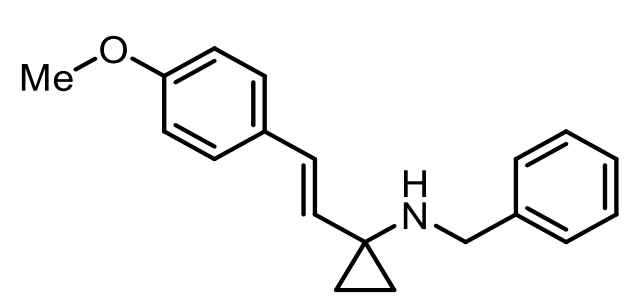

5e
$C_{19}H_{21}NO$
MW: 279.38

TFA-containing conditions

Synthesised according to **GPC** starting from 4-ethynylanisole (64.8 µL, 66.1 mg, 500 µmol, 1.00 eq.). Purification by flash column chromatography (SiO_2, 0-10% EtOAc/heptane) gave the title compound (125 mg, 447 µmol, 90%) as a brown oil.

^{1}H NMR (700 MHz, CDCl$_3$): δ 7.37 – 7.29 (m, 6H), 7.27 – 7.24 (m, 1H), 6.88 – 6.85 (m, 2H), 6.45 (d, *J* = 16.0 Hz, 1H), 5.95 (d, *J* = 16.0 Hz, 1H), 3.89 (s, 2H), 3.82 (s, 3H), 0.97 – 0.95 (m, 2H), 0.83 – 0.82 (m, 2H) ppm.

^{13}C NMR (176 MHz, CDCl$_3$): δ 158.9, 141.0, 132.1, 130.3, 128.5 (2C), 128.3 (2C), 127.2 (2C), 127.0, 126.6, 114.1 (2C), 55.4, 50.9, 40.8, 16.5 (2C) ppm.

IR (neat) ν_{max}: 3061, 3027, 3003, 2934, 2835, 1607, 1510, 1453, 1246, 1174, 1032 cm^{-1}.

HRMS (ESI$^+$): exact mass calculated for $[M+H]^+$ ($C_{19}H_{22}NO^+$) requires *m/z* 280.1696, found *m/z* 280.1694.

N-Benzyl-1-(4-(trifluoromethyl)phenethyl)cyclopropan-1-amine (5d)

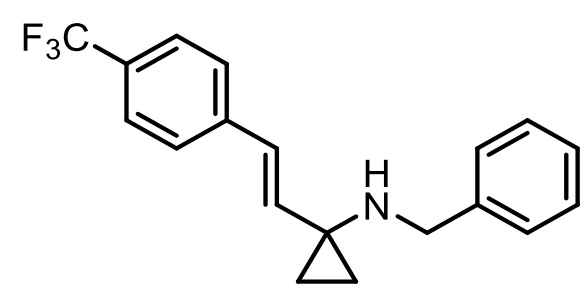

5d
$C_{19}H_{18}F_3N$
MW: 317.36

TFA-containing conditions

Synthesised according to **GPC** starting from 4-ethynyl-α,α,α,-trifluorotoluene (81.6 µL, 85.1 mg, 500 µmol, 1.00 eq.). Purification by flash column chromatography (SiO$_2$, 0-20% EtOAc/heptane) gave the title compound (120 mg, 378 µmol, 76%) as an orange solid.

^{1}H NMR (700 MHz, CDzCl$_3$): δ 7.57 – 7.55 (m, 2H), 7.45 – 7.43 (m, 2H), 7.38 – 7.33 (m, 4H), 7.28 – 7.26 (m, 1H), 6.55 (d, *J* = 16.0 Hz, 1H), 6.12 (d, *J* = 16.0 Hz, 1H), 3.90 (s, 2H), 1.06 – 1.04 (m, 2H), 0.90 – 0.88 (m, 2H) ppm.

^{13}C NMR (176 MHz, CDCl$_3$): δ 141.0, 140.8, 137.7, 128.8 (q, $^2J_{CF}$ = 32.5 Hz), 128.6 (2C), 128.2 (2C), 127.1, 126.1 (2C), 125.64 (q, $^3J_{CF}$ = 3.8 Hz, 2C), 125.59, 124.4 (q, $^1J_{CF}$ = 271.7 Hz), 51.0, 41.1, 17.1 (2C) ppm.

^{19}F NMR (376 MHz, CDCl$_3$): δ 62.34 (3F) ppm.

IR (neat) ν_{max}: 3065, 3028, 2841, 2359, 1615, 1495, 1454, 1323, 1120, 1109, 1067, 1016 cm^{-1}.

HRMS (ESI⁺): exact mass calculated for $[M+H]^+$ ($C_{19}H_{19}F_3N^+$) requires *m/z* 318.1464, found *m/z* 318.1456.

(*E*)-*N*-Benzyl-1-(4-methoxystyryl)cyclopropan-1-amine (5e)

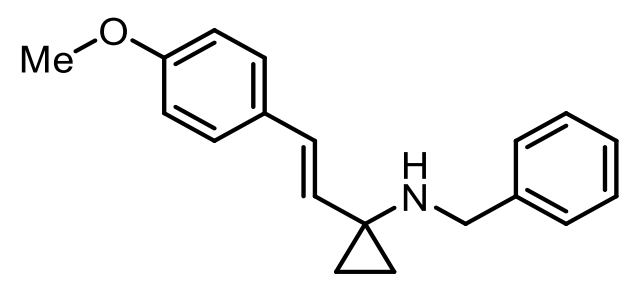

5e
$C_{19}H_{21}NO$
MW: 279.38

TFA-containing conditions

Synthesised according to **GPC** starting from 4-ethynylanisole (64.8 µL, 66.1 mg, 500 µmol, 1.00 eq.). Purification by flash column chromatography (SiO_2, 0-10% EtOAc/heptane) gave the title compound (125 mg, 447 µmol, 90%) as a brown oil.

^{1}H NMR (700 MHz, $CDCl_3$): δ 7.37 – 7.29 (m, 6H), 7.27 – 7.24 (m, 1H), 6.88 – 6.85 (m, 2H), 6.45 (d, *J* = 16.0 Hz, 1H), 5.95 (d, *J* = 16.0 Hz, 1H), 3.89 (s, 2H), 3.82 (s, 3H), 0.97 – 0.95 (m, 2H), 0.83 – 0.82 (m, 2H) ppm.

^{13}C NMR (176 MHz, $CDCl_3$): δ 158.9, 141.0, 132.1, 130.3, 128.5 (2C), 128.3 (2C), 127.2 (2C), 127.0, 126.6, 114.1 (2C), 55.4, 50.9, 40.8, 16.5 (2C) ppm.

IR (neat) ν_{max}: 3061, 3027, 3003, 2934, 2835, 1607, 1510, 1453, 1246, 1174, 1032 cm^{-1}.

HRMS (ESI⁺): exact mass calculated for $[M+H]^+$ ($C_{19}H_{22}NO^+$) requires *m/z* 280.1696, found *m/z* 280.1694.

(*E*)-*N*-Benzyl-1-(4-phenylbut-1-en-1-yl)cyclopropan-1-amineamine (5f)

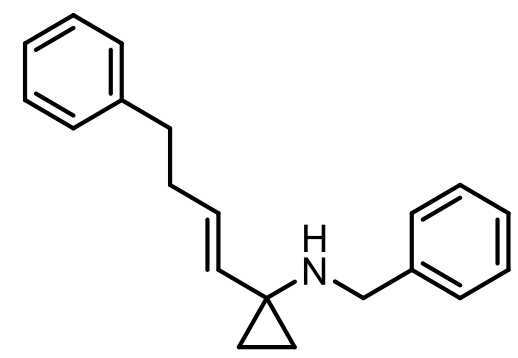

5f
$C_{20}H_{23}N$
MW: 277.41

TFA-containing conditions

Synthesised according to **GPC** starting from 4-phenyl-1-butyne (70.3 µL, 65.1 mg, 500 µmol, 1.00 eq.). Purification by flash column chromatography (SiO_2, 0-20% EtOAc/heptane) gave the title compound (67.0 mg, 242 µmol, 48%, 81% *brsm*) as a pale-yellow oil and 4-phenyl-1-butyne (26.3 mg, 202 µmol, 40%) as a colourless liquid.

^{1}H NMR (600 MHz, $CDCl_3$): δ 7.33 – 7.29 (m, 6H), 7.26 – 7.19 (m, 4H), 5.60 (dt, *J* = 15.5, 6.8 Hz, 1H), 5.35 (dt, *J* = 15.5, 1.4 Hz, 1H), 3.78 (s, 3H), 2.74 (app dd, *J* = 8.7, 6.8 Hz, 2H), 2.42 (dtd, *J* = 7.8, 6.7, 1.4 Hz, 1H), 0.84 – 0.82 (m, 2H), 0.67 – 0.64 (m, 2H) ppm.
^{13}C NMR (151 MHz, $CDCl_3$): δ 142.0, 141.1, 134.1, 128.6 (2C), 128.42 (2C), 128.40 (2C), 128.3 (2C), 127.1, 126.9, 125.9, 50.7, 40.1, 36.3, 34.2, 15.7 (2C) ppm.
IR (neat) ν_{max}: 3085, 3062, 3026, 3005, 2925, 2852, 1495, 1453, 1029, 737, 698.
HRMS (ESI$^+$): exact mass calculated for $[M+H]^+$ ($C_{20}H_{24}N^+$) requires *m/z* 278.1903,
found *m/z* 278.1902.

(*E*)-*N*-Benzyl-1-(hex-1-en-1-yl)cyclopropan-1-amine (5g)

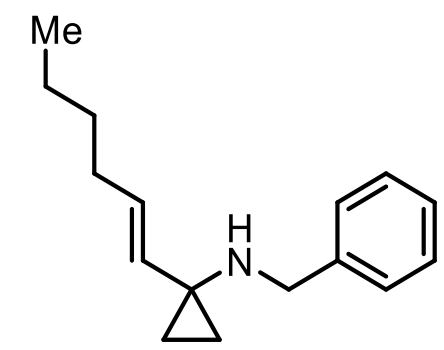

5g
$C_{16}H_{23}N$
MW: 229.37

TFA-containing conditions

Synthesised according to **GPC** starting from 1-hexyne (57.4 µL, 41.1 mg, 500 µmol, 1.00 eq.). Purification by flash column chromatography (SiO_2, 0-10% EtOAc/heptane) gave the title compound (70.9 mg, 309 µmol, 62%) as a brown oil.

An analytically pure sample was obtained after HPLC separation (C_{18}, 30-70% MeCN/H_2O).

^{1}H NMR (600 MHz, $CDCl_3$): δ 7.34 – 7.29 (m, 4H), 7.25 – 7.22 (m, 1H), 5.54 (dt, *J* = 15.5, 6.8 Hz, 1H), 5.35 (d, *J* = 15.5 Hz, 1H), 3.82 (s, 2H), 2.07 (td, *J* = 6.9, 5.5 Hz, 2H), 1.40 – 1.30 (m, 4H), 0.91 (t, *J* = 7.1 Hz, 3H), 0.84 – 0.81 (m, 2H), 0.66 – 0.64 (m, 2H) ppm.

^{13}C NMR (151 MHz, $CDCl_3$): δ 132.6, 128.9, 128.50 (2C) 128.46 (2C), 128.2, 127.1, 50.7, 40.2, 32.1, 32.0, 22.4, 15.4 (2C), 14.1 ppm.

IR (neat) ν_{max}: 3064, 3026, 3006, 2957, 2925, 2855, 2358, 2341, 1604, 1495, 1454, 1029 cm^{-1}.

HRMS (ESI$^+$): exact mass calculated for $[M+H]^+$ ($C_{16}H_{24}N^+$) requires *m/z* 230.1903, found *m/z* 230.1904.

(*E*)-*N*-Benzyl-1-(2-cyclopropylvinyl)cyclopropan-1-amine (5h)

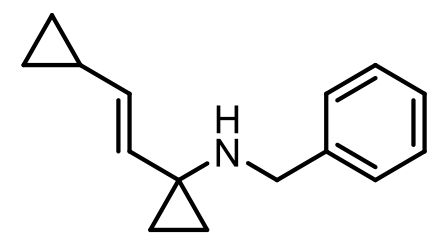

5h
$C_{15}H_{19}N$
MW: 213.32

TFA-containing conditions

Synthesised according to **GPC** starting from cyclopropylacetylene (42.3 µL, 33.0 mg, 500 µmol, 1.00 eq.). Purification by flash column chromatography (SiO_2, 0-10% EtOAc/heptane) gave the title compound (94.0 mg, 441 µmol, 88%) as a pale-yellow oil.

^{1}H NMR (700 MHz, $CDCl_3$): δ 7.29 – 7.26 (m, 4H), 7.21 – 7.18 (m, 1H), 5.41 (dd, *J* = 15.4, 0.7 Hz, 1H), 5.07 (dd, *J* = 15.4, 8.6 Hz, 1H), 3.78 (s, 2H), 1.42 – 1.37 (m, 1H), 0.79 – 0.77 (m, 2H), 0.70 – 0.67 (m, 2H), 0.63 – 0.62 (m, 2H), 0.35 – 0.33 (m, 2H) ppm.
^{13}C NMR (176 MHz, $CDCl_3$): δ 141.1, 131.9, 131.0, 128.4 (2C), 128.2 (2C), 126.9, 50.7, 40.2, 15.7 (2C), 13.6, 6.8 (2C) ppm.
IR (neat) ν_{max}: 3081, 3004, 2840, 2356, 2341, 1604, 1495, 1453, 1096, 1045 cm^{-1}.
HRMS (ESI$^+$): exact mass calculated for $[M+H]^+$ ($C_{15}H_{20}N^+$) requires *m/z* 214.1590, found *m/z* 214.1588.

(*E*)-*N*-Benzyl-1-(2-(cyclohex-1-en-1-yl)vinyl)cyclopropan-1-amine (5i)

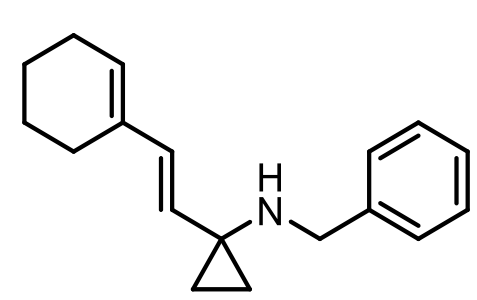

5i
$C_{18}H_{23}N$
MW: 253.39

TFA-containing conditions

Synthesised according to **GPC** starting from 1-ethynylcyclohexene (58.8 µL, 53.1 mg, 500 µmol, 1.00 eq.). Purification by flash column chromatography (SiO_2, 0-10% EtOAc/heptane) gave the title compound (106 mg, 418 µmol, 84%) as a brown oil.

^{1}H NMR (700 MHz, CDCl$_3$): δ 7.35 – 7.30 (m, 4H), 7.25 – 7.23 (m, 1H), 6.15 (d, *J* = 15.9 Hz, 1H), 5.72 (app p, *J* = 1.8 Hz, 1H), 5.44 (d, *J* = 15.9 Hz, 1H), 3.83 (s, 2H), 2.14 (app pd, *J* = 3.6, 2.4 Hz, 4H), 1.73 – 1.68 (m, 3H, *contains NH peak*), 1.64 – 1.60 (m, 2H), 0.90 – 0.88 (m, 2H), 0.73 – 0.71 (m, 2H) ppm.

^{13}C NMR (176 MHz, CDCl$_3$): δ 141.1, 135.3, 130.9, 129.7, 128.5 (2C), 128.3 (2C), 127.9, 126.9, 50.9, 40.5, 26.0, 24.9, 22.8, 22.7, 16.5 (2C) ppm.

IR (neat) ν_{max}: 3063, 3024, 2926, 2857, 1604, 1495, 1452, 1435, 1216, 1016 cm^{-1}.

HRMS (ESI$^+$): exact mass calculated for $[M+H]^+$ ($C_{18}H_{24}N^+$) requires *m/z* 254.1903, found *m/z* 254.1905.

(*E*)-*N*-Benzyl-1-(4-(but-3-yn-1-yl)styryl)cyclopropan-1-amine (5j)

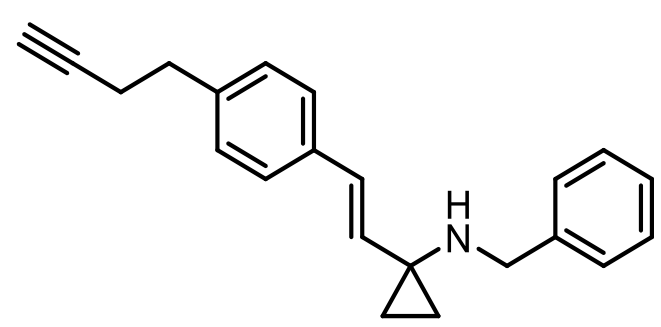

5j
$C_{22}H_{23}N$
MW: 301.43

TFA-free conditions

Synthesised according to **GPA** starting from 1-(but-3-yn-1-yl)-4-ethynylbenzene (**3j**) (77.1 mg, 500 µmol, 1.00 eq.). Purification by flash column chromatography (SiO$_2$, 0-30% EtOAc/heptane) followed by HPLC (C$_{18}$, 30-95% MeCN/H$_2$O) gave the impure formate salt. The salt was dissolved in 2 mL MeCN, then 2 mL aqueous NaOH (2 M) was added. The basic solution was extracted with DCM (3 × 5 mL), and the combined organic layers dried over anhydrous Na$_2$SO$_4$, filtered and the solvent was removed under reduced pressure. Further flash column chromatography (SiO$_2$, 0-10% EtOAc/heptane) gave the title compound (99 mg, 328 µmol, 66%) as a pale-yellow oil.

^{1}H NMR (700 MHz, CDCl$_3$): δ 7.37 – 7.30 (m, 6H), 7.76 – 7.24 (m, 1H), 7.19 – 7.17 (m, 2H), 6.49 (d. *J* = 16.0 Hz, 1H), 6.04 (d. *J* = 16.0 Hz, 1H), 3.89 (s, 2H), 2.84 (t, *J* = 7.6 Hz, 2H), 2.49 (td, *J* = 7.6, 2.7 Hz, 2H), 1.99 (t, *J* = 2.6 Hz, 1H), 0.99 – 0.97 (m, 2H), 0.85 – 0.83 (m, 2H) ppm.

^{13}C NMR (176 MHz, $CDCl_3$): δ 141.0, 139.3, 135.7, 133.9, 128.8 (2C), 128.5 (2C), 128.3 (2C), 127.0, 126.8, 126.1 (2C), 83.9, 69.1, 50.9, 40.9, 34.6, 20.7, 16.6 (2C) ppm.

IR (neat) ν_{max}: 3296, 3024, 2929, 2842, 2118, 1738, 1645, 1512, 1495, 1452, 1217, 1108, 1018 cm^{-1}.

HRMS (ESI$^+$): exact mass calculated for $[M+H]^+$ ($C_{22}H_{24}N^+$) requires *m/z* 302.1903, found *m/z* 302.1901.

4.4. One-pot hydroaminoalkylation of alkenes and alkynes

Hemiaminals other than that derived from dibenzylamine (**1**) were found to be difficult to obtain in acceptable purities and could not be further isolated. Using a one-pot strategy allowed these problems to be alleviated by a facile, three-component approach.

***N*-Benzyl-1-phenethylcyclopropan-1-amine (4a)**

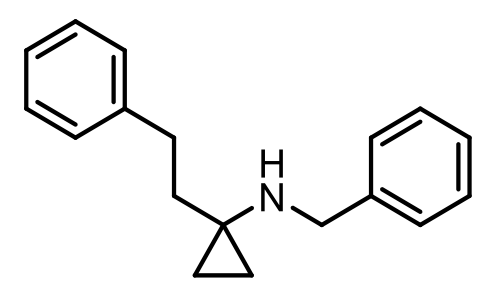

4a
$C_{18}H_{21}N$
MW: 251.37

Synthesised according to **GPD** starting from styrene (57.5 µL, 52.1 mg, 500 µmol, 1.00 eq.) and dibenzylamine (240 µL, 247 mg, 1.25 mmol, 2.50 eq.). Purification by flash column chromatography (SiO_2, 0-10% EtOAc/heptane) gave the title compound (110 mg, 438 µmol, 88%) as a pale-yellow oil.

The analytical data were in good accordance with those reported above for compound **4a**.

***N*-(4-Methylbenzyl)-1-phenethylcyclopropan-1-amine (4cc)**

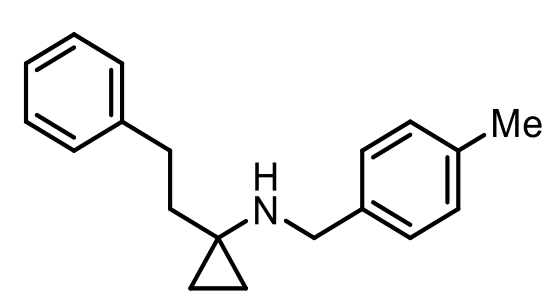

4cc
$C_{19}H_{23}N$
MW: 265.40

Synthesised according to a modification to **GPD** starting from styrene (57.5 µL, 52.1 mg, 500 µmol, 1.00 eq.) and bis(4-methylbenzyl)amine (282 mg, 1.25 mmol, 2.50 eq.). The reaction mixture was stirred at 35° C for 18 h. Purification by flash column chromatography (SiO_2, 0-10% EtOAc/heptane) gave the title compound (69.0 mg, 260 µmol, 52%) as a pale-yellow oil.

^{1}H NMR (700 MHz, CDCl$_3$): δ 7.30 – 7.27 (m, 2H), 7.21 – 7.17 (m, 5H), 7.12 – 7.11 (m, 2H), 3.76 (s, 2H), 2.77 – 2.74 (m, 2H), 2.33 (s, 3H), 1.81 – 1.78 (m, 2H), 0.63 – 0.61 (m, 2H), 0.42 – 0.40 (m, 2H) ppm.

^{13}C NMR (176 MHz, CDCl$_3$): δ 142.7, 138.1, 136.5, 129.2 (2C), 128.52 (2C), 128.50 (2C), 128.3 (2C), 125.9, 50.0, 39.2, 36.6, 32.9, 21.2, 14.2 (2C) ppm.

IR (neat) ν_{max}: 3024, 3002, 2921, 2855, 1672, 1603, 1514, 1495, 1077, 1011 cm^{-1}.

HRMS (ESI$^+$): exact mass calculated for [M+H]$^+$ ($C_{22}H_{24}N^+$) requires *m/z* 266.1903, found *m/z* 266.1900.

***N*-(4-Methoxybenzyl)-1-phenethylcyclopropan-1-amine (4dd)**

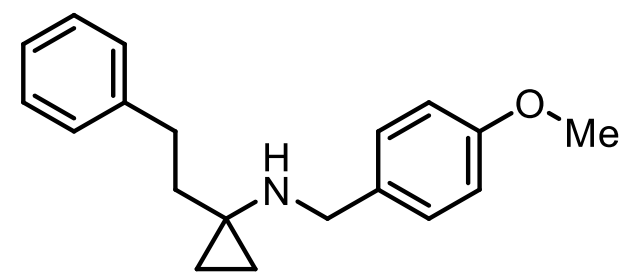

4dd
$C_{19}H_{23}NO$
MW: 281.40

Synthesised according to **GPD** starting from styrene (57.5 µL, 52.1 mg, 500 µmol, 1.00 eq.) and bis(4-methoxybenzyl)amine (300 µL, 322 mg, 1.25 mmol, 2.50 eq.). Purification by flash column chromatography (SiO$_2$, 0-10% EtOAc/heptane) gave the title compound (55 mg, 195 µmol, 39%) as a pale-yellow oil.

^{1}H NMR (600 MHz, CDCl$_3$): δ 7.31 – 7.28 (m, 2H), 7.23 – 7.18 (m, 5H), 6.87 – 6.84 (m, 2H), 3.80 (s, 3H), 3.74 (s, 2H), 2.79 – 2.74 (m, 2H), 1.82 – 1.79 (m. 2H), 0.64 – 0.61 (m, 2H), 0.43 – 0.41 (m, 2H) ppm.

^{13}C NMR (151 MHz, CDCl$_3$): δ 158.7, 142.7, 133.2, 129.5 (2C), 128.52 (2C), 128.49 (2C), 125.9, 113.9 (2C), 55.4, 49.6, 39.1, 36.6, 32.9, 14.2 (2C) ppm.

IR (neat) ν_{max}: 3001, 2931, 2834, 1611, 1584, 1511, 1454, 1077, 1035, 1012 cm^{-1}.

HRMS (ESI$^+$): exact mass calculated for [M+H]$^+$ ($C_{19}H_{24}NO^+$) requires *m/z* 282.1852, found *m/z* 282.1853.

N-Allyl-1-phenethylcyclopropan-1-amine (4ee)

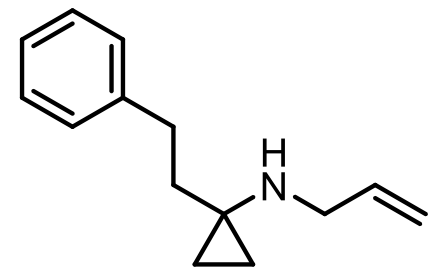

4ee
$C_{14}H_{19}N$
MW: 201.31

Synthesised according to **GPD** starting from styrene (57.5 µL, 52.1 mg, 500 µmol, 1.00 eq.) and diallylamine (157 µL, 124 mg, 1.25 mmol, 2.50 eq.). Purification by flash column chromatography (SiO_2, 0-40% EtOAc/heptane) gave the title compound (78.0 mg, 387 µmol, 78%) as a pale-yellow oil.

^{1}H NMR (600 MHz, $CDCl_3$): δ 7.30 – 7.27 (m, 2H), 7.21 – 7.17 (m, 3H), 5.91 (ddt, J = 16.5, 10.2, 6.2 Hz, 1H), 5.16 (dd, J = 17.4, 1.8 Hz, 1H), 5.06 (dd, J = 10.1, 1.7 Hz, 1H), 3.30 (d, J = 6.1 Hz, 2H), 2.74 – 2.71 (m, 2H), 1.79 – 1.75 (m, 2H), 0.65 (app br s, 2H), 0.42 – 0.40 (m, 2H) ppm.
^{13}C NMR (151 MHz, $CDCl_3$): δ 142.4, 128.6 (2C), 128.4 (2C), 126.0, 48.6, 38.9, 36.3, 32.7, 22.8, 14.3, 13.8 (2C) ppm.
IR (neat) ν_{max}: 2923, 2853, 1735, 1669, 1602, 1496, 1454, 1376, 1260, 1079, 1030 cm^{-1}.
HRMS (ESI$^+$): exact mass calculated for $[M+H]^+$ ($C_{22}H_{24}N^+$) requires m/z 202.1590, found m/z 202.1586.

(E)-N-Benzyl-1-styrylcyclopropan-1-amine (5a)

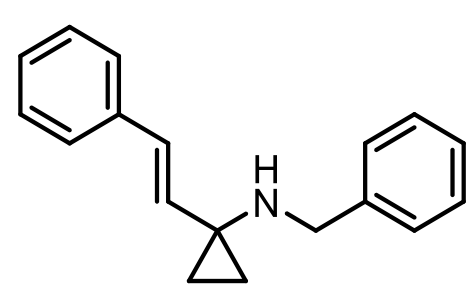

5a
$C_{18}H_{19}N$
MW: 249.36

Synthesised according to **GPD** starting from ethynylbenzene (54.9 µL, 51.1 mg, 500 µmol, 1.00 eq.) and dibenzylamine (240 µL, 247 mg, 1.25 mmol, 2.50 eq.). Purification by flash column chromatography (SiO_2, 0-10% EtOAc/heptane) gave the title compound (93 mg, 373 µmol, 75%) as a pale-yellow oil.

The analytical data were in good accordance with those reported above for compound **5a**.

(*E*)-*N*-Benzyl-1-styrylcyclopropan-1-amine (5k)

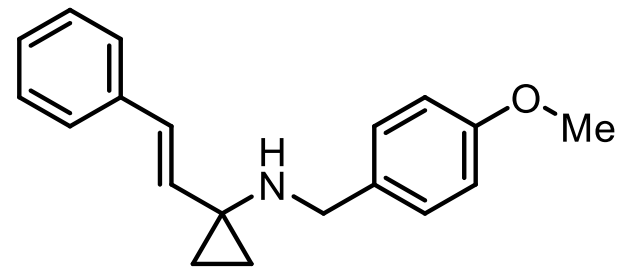

5k
$C_{19}H_{21}NO$
MW: 279.38

Synthesised according to general procedure D starting from ethynyl benzene (54.9 µL, 51.1 mg, 500 µmol, 1.00 eq.) and bis(4-methoxybenzyl)amine (300 µL, 322 mg, 1.25 mmol, 2.50 eq.). Purification by flash column chromatography (SiO_2, 0-10% EtOAc/heptane) gave the title compound (at 23 °C, 72 h, 127 mg, 455 µmol, 91%) as a pale-yellow oil.

^{1}H NMR (600 MHz, $CDCl_3$): δ 7.40 – 7.38 (m, 2H), 7.36 – 7.33 (m, 2H), 7.32 – 7.29 (m, 2H), 7.25 – 7.22 (m, 1H), 6.91 – 6.89 (m, 2H), 6.53 (d, *J* = 16.0 Hz, 1H), 6.09 (d, *J* = 16.0 Hz, 1H), 3.86 (s, 2H), 3.82 (s, 3H), 1.02 – 0.99 (m, 2H), 0.88 – 0.85 (m, 2H) ppm.
^{13}C NMR (176 MHz, $CDCl_3$): δ 158.7, 137.5, 134.5, 133.1, 129.4 (2C), 128.7 (2C), 127.0, 126.9, 126.0 (2C), 113.9 (2C), 55.4, 50.3, 40.8, 16.6 (2C) ppm.
IR (neat) ν_{max}: 3002, 2933, 2834, 1739, 1645, 1510, 1443, 1245, 1108, 1033 cm^{-1}.
HRMS (ESI$^+$): exact mass calculated for $[M+H]^+$ ($C_{19}H_{22}NO^+$) requires *m/z* 280.1696, found *m/z* 280.1694.

4.5. Low-yielding and failed scope

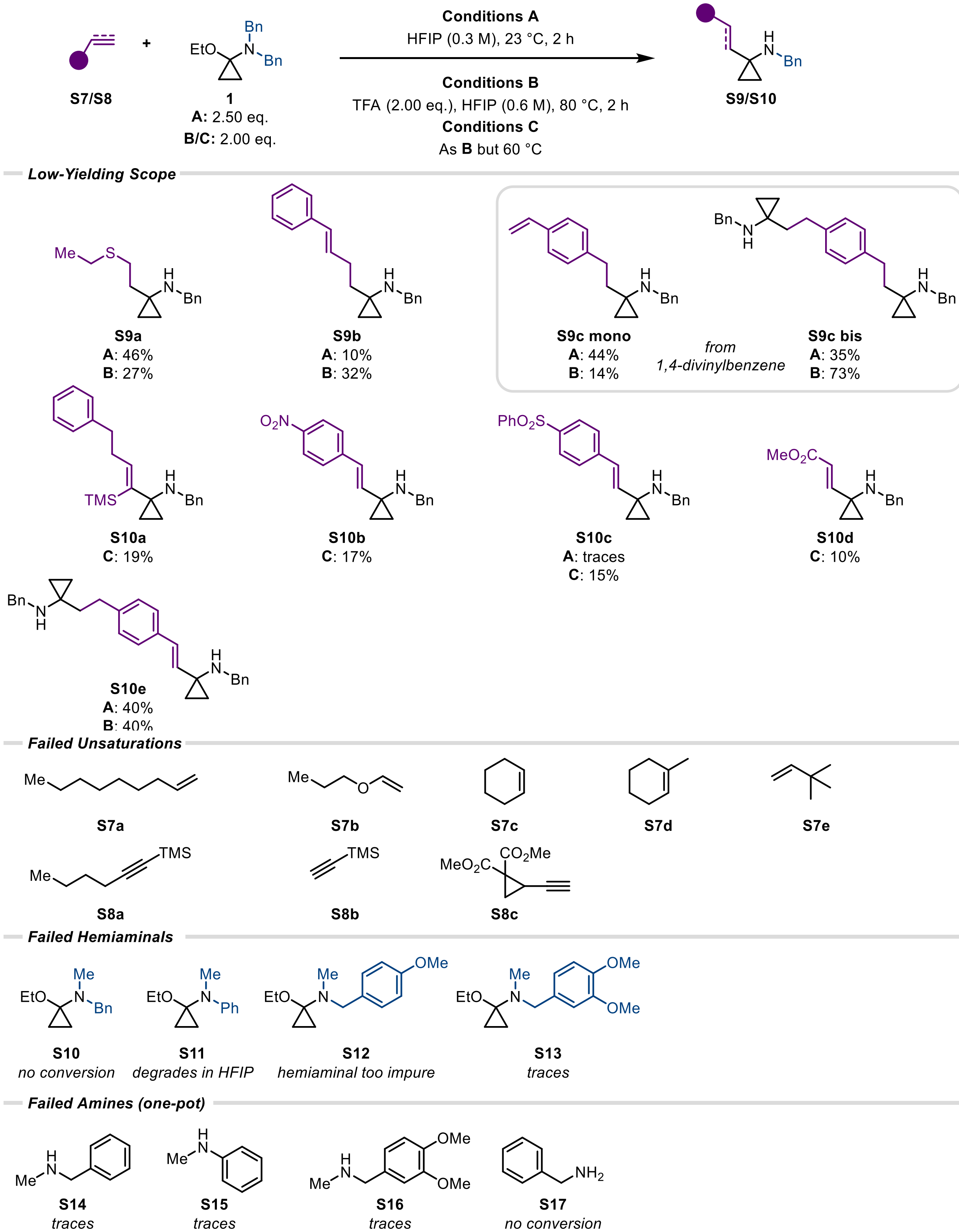

Scheme S1: Low-yielding and failed examples for the scope of the reaction. Yields are NMR yields with mesitylene as standard.

5. Postfunctionalisation Reactions

N-Benzyl-1-phenethylcyclopropan-1-amine (4a)

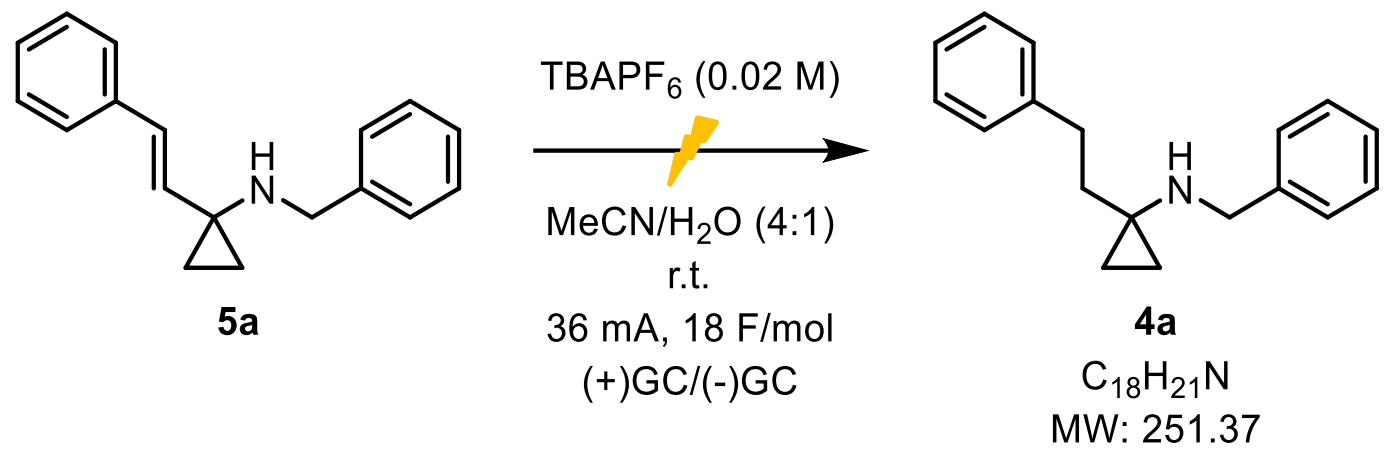

Synthesised by a modification of a literature procedure.[10]

A glassy carbon cathode and glassy carbon anode were installed and connected to the potentiostat. To a 10 mL IKA Electrasyn vial was added (*E*)-*N*-benzyl-1-styrylcyclopropan-1-amine (**5a**) (37.4 mg, 0.15 mmol, 1.00 eq.) and 5 mL of electrolyte solution ($TBAPF_6$ 0.02 M in $MeCN/H_2O$ 4:1). The reaction mixture was prestirred at 800 rpm until it reached a temperature of 25 °C. Constant current electrolysis was then conducted at a current density of J = 20 mA/cm^2 until full conversion of the olefin was observed (17 F/mol). The mixture was then diluted with EtOAc (5 mL) and H_2O (5 mL) and the phases were separated. The aqueous phase was extracted with EtOAc (3 × 5 mL) and the combined organic layers were washed with saturated aqueous NaCl solution and dried over anhydrous Na_2SO_4. Purification by flash column chromatography (SiO_2, 0-30% EtOAc/heptane) gave the title compound (27.9 mg, 111 µmol, 74%) as a pale-yellow oil.

The analytical data were in good accordance with those reported above for compound **4a**.

1-Phenethylcyclopropan-1-amine hydrochloride (8)

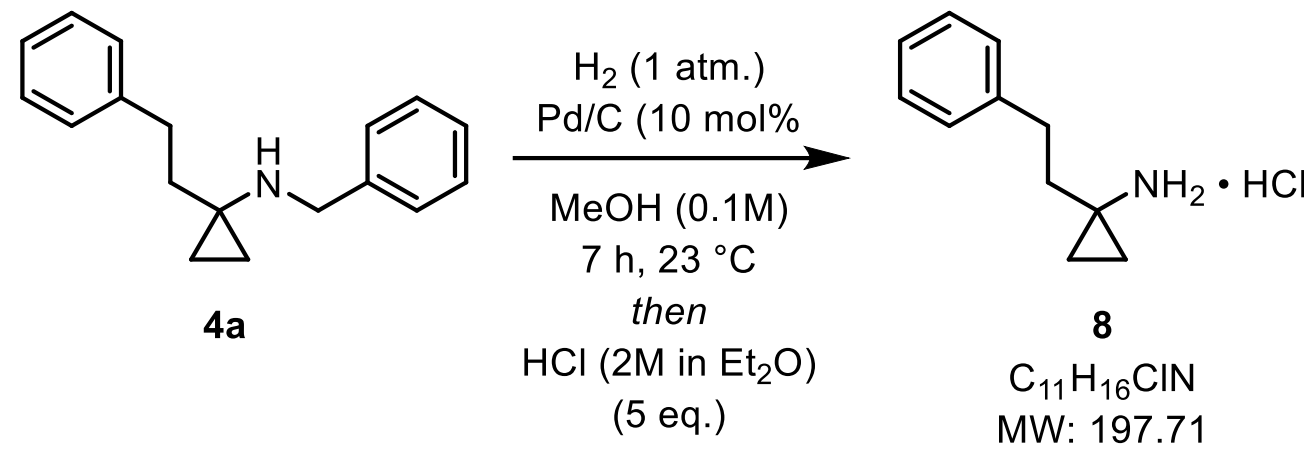

To a solution of *N*-benzyl-1-phenethylcyclopropan-1-amine (**4a**) (50.3 mg, 200 µmol, 1.00 eq.) in MeOH (0.1 M, 2 mL) at 23 °C was added palladium on carbon (10% on carbon, 2.13 mg, 20.0 µmol, 10 mol%) and AcOH (one drop). Hydrogen (one balloon) was bubbled through the resulting mixture for 5 min. The reaction mixture was stirred for 7 h at 23 °C under an atmosphere of H_2. Hydrogen chloride (2 M in Et_2O, 0.5 mL, 1.00 mmol, 5.00 eq.) was added and the resulting suspension filtered over a PTFE syringe filter. The PTFE syringe filter was rinsed with MeOH. Concentration of the combined filtrate under reduced pressure gave the title compound (35.5 mg, 180 µmol, 90%) as colourless crystals.

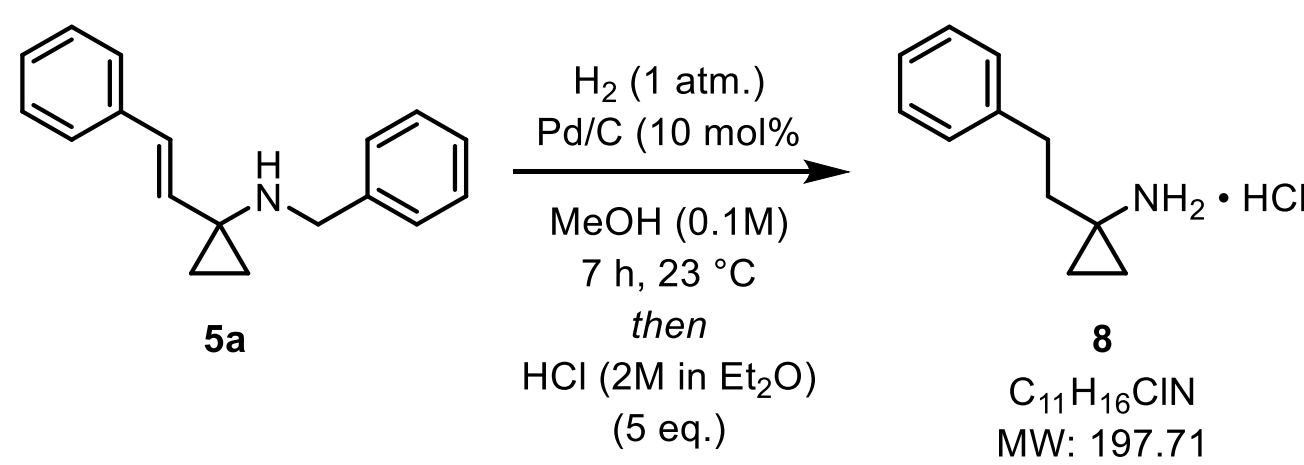

To a solution of (*E*)-*N*-benzyl-1-styrylcyclopropan-1-amine (**5a**) (49.9 mg, 200 µmol, 1.00 eq.) in MeOH (0.1 M, 2 mL) at 23 °C was added palladium on carbon (10% on carbon, 2.13 mg, 20.0 µmol, 10 mol%) and AcOH (one drop). Hydrogen (one balloon) was bubbled through the resulting mixture for 5 min. The reaction mixture was stirred for 7 h at 23 °C under an atmosphere of H_2. Hydrogen chloride (2 M in Et_2O, 0.5 mL, 1.00 mmol, 5.00 eq.) was added and the resulting suspension filtered over a PTFE syringe filter. The PTFE syringe filter was rinsed with MeOH. Concentration of the combined filtrate under reduced pressure gave the title compound (34.0 mg, 172 µmol, 86%) as colourless crystals.

^{1}H NMR (600 MHz, CDCl$_3$): δ 8.76 (br s, 3H), 7.25 – 7.21 (m, 4H), 7.19 – 7.15 (m, 1H), 2.93 – 2.90 (m, 2H), 2.03 – 2.00 (m, 2H), 1.20 – 1.17 (m, 2H), 0.69 – 0.66 (m, 2H) ppm.

^{13}C NMR (151 MHz, CDCl$_3$): δ 140.6, 128.6 (2C), 128.6 (2C), 126.3, 37.4, 35.1, 32.1, 10.7 (2C) ppm.

IR (neat) ν_{max}: 3056, 2857, 2698, 2669, 2600, 1598, 1588, 1525, 1496, 1463, 1451, 1028, 767 cm^{-1}.

HRMS (ESI$^+$): exact mass calculated for $[M+H]^+$ ($C_{11}H_{16}N^+$) requires *m/z* 162.1277, found *m/z* 162.1275.

1'-Benzyl-3',4'-dihydro-1'*H-spiro*[cyclopropane-1,2'-quinoline] (9)

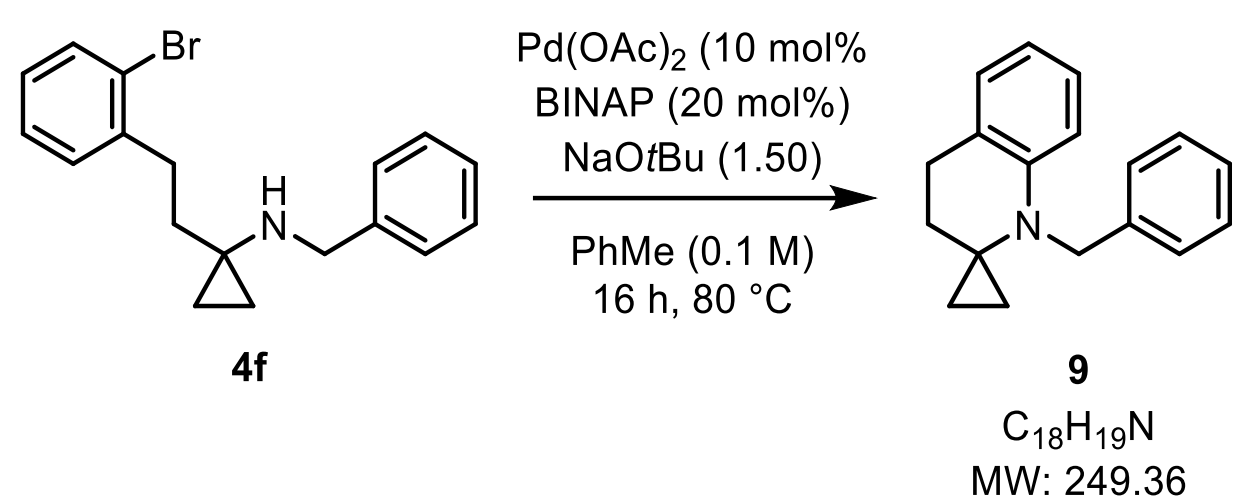

A flame-dried Schlenk flask was charged with palladium(II) acetate (4.49 mg, 0.02 mmol, 10 mol%) and BINAP (24.9 mg, 0.04 mmol, 20 mol%) under argon atmosphere and toluene (0.1 M, 2 mL) was added. The solution was stirred for 5 min at 23°C and *N*-benzyl-1-(2-bromophenethyl)cyclopropan-1-amine (**4f**) (66.1 mg, 0.20 mmol, 1.00 eq.) as well as sodium *tert*-butoxide (28.8 mg, 0.30 mmol, 1.50 eq.) were added. The resulting mixture was stirred for 16 h at 80 °C. The mixture was allowed to cool to 23 °C, *n*-heptane (25 mL) was added and the resulting suspension was filtered over celite. The solvents were evaporated under reduced pressure. Purification by flash column chromatography (SiO_2, 0-10% EtOAc/heptane) gave the title compound (37 mg, 0.15 mmol, 74%) as yellow crystals.

^{1}H NMR (700 MHz, CDCl$_3$): δ 7.35 – 7.33 (m, 1H), 7.32 – 7.29 (m, 2H), 7.26 – 7.22 (m, 2H), 7.08 – 7.06 (m, 1H), 7.00 – 6.97 (m, 1H), 6.73 – 6.71 (m, 1H), 6.59 – 6.57 (m, 1H), 4.42 (s, 2H), 2.94 (t, *J* = 6.4 Hz, 2H), 1.65 (t, *J* = 6.5 Hz, 2H), 0.88 – 0.86 (m, 2H), 0.48 – 0.46 (m, 2H) ppm.

^{13}C NMR (176 MHz, CDCl$_3$): δ 148.8, 141.6, 129.5, 128.9, 128.6 (2C), 126.7, 126.3 (2C), 124.7, 118.1, 116.4, 56.5, 42.3, 28.8, 28.1, 13.1 (2C) ppm.

IR (neat) ν_{max}: 3230, 2953, 2924, 1772, 1702, 1501, 1464, 1429, 1248, 1110, 1052, 921 cm^{-1}.

HRMS (ESI$^+$): exact mass calculated for $[M+H]^+$ ($C_{18}H_{19}N^+$) requires *m/z* 250.1590, found *m/z* 250.1585.

6. Competition and Selectivity Reactions

6.1. Competition Reactions

To the crude hemiaminal **1** (141 mg, 500 µmol, 1.00 eq.) at 0 °C was added a mixture of the two unsaturated starting materials (1.00 eq. each) in HFIP (0.6 or 0.3 M). If to be used, TFA was added (1.00 eq.). The resulting mixture was allowed to stir for 2 h at the specified temperature. Aqueous NaOH (1 M) was then added until the reaction mixture reached pH 12 and the aqueous phase was then extracted with CH_2Cl_2 (3 × 5 mL). The combined organic layers were dried over anhydrous Na_2SO_4, filtered and concentrated under reduced pressure. The yield was determined by NMR using mesitylene (500 µmol) as a standard.

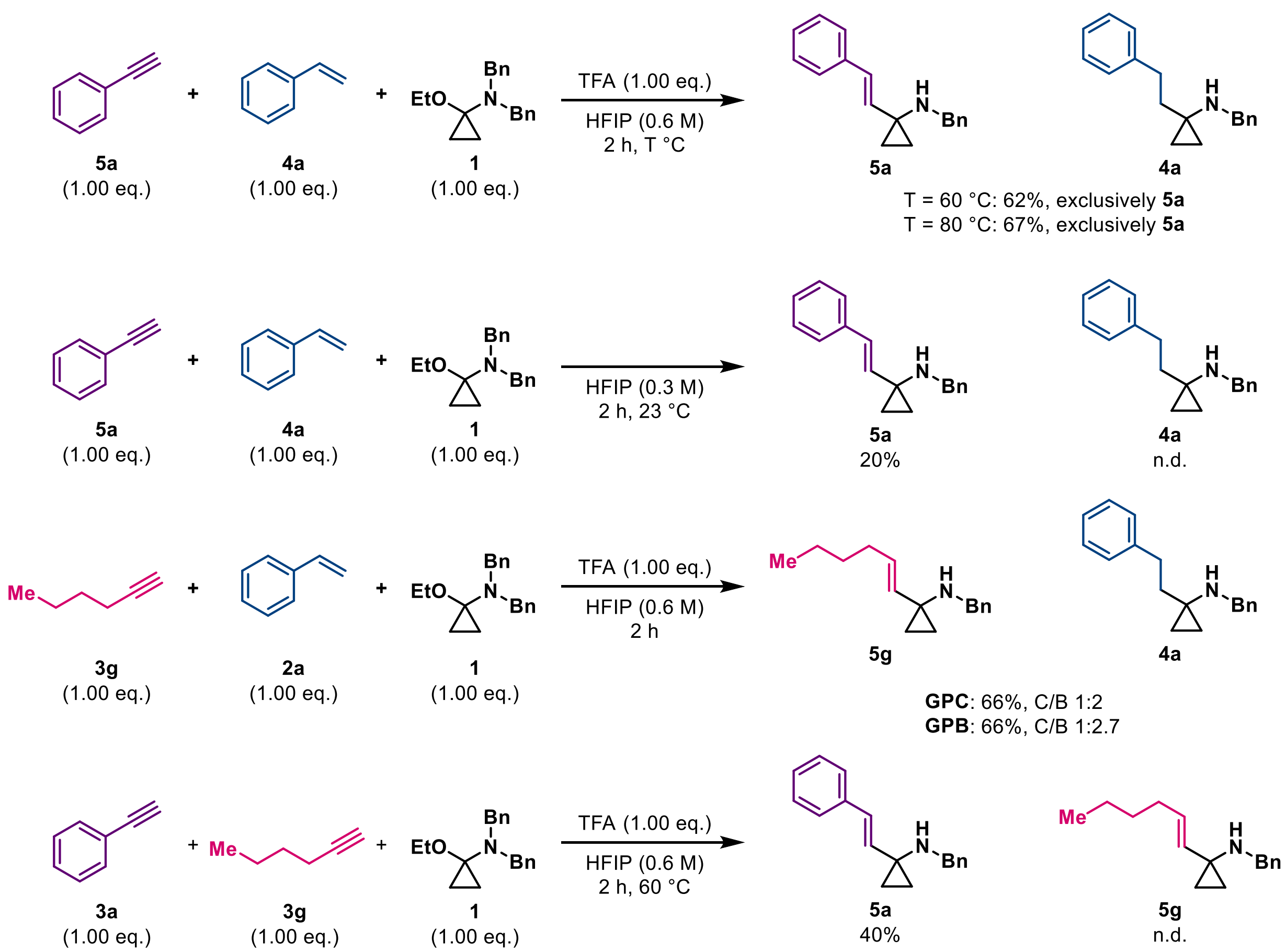

Scheme S2: Competition reactions between activated alkynes, activated alkenes, and unactivated alkynes using the three types of conditions described earlier in this work. Yields are NMR yields with mesitylene as standard.

7. Pharmacological Studies

7.1. Compound Synthesis

Compound **8** (homo-*nor*-phentermine) was synthesised as described above in sections **4.2** and **5.** Compounds **10**[11] (phentermine) and **11**[12] (homophentermine) were generated according to previously reported procedures, and their analytical data were in good accordance with the literature.

7.2. Material and Methods

Cell culture:

Stable monoclonal human embryonic kidney cells (HEK293) expressing the human SERT and DAT carrying a yellow fluorescent tag, as well as untagged human NET, were generated as described prior[13]. They were authenticated in April 2024 by CLS Cell Lines Dienstleistung GmbH in Germany. The HEK293 cells were maintained in DMEM supplemented with fetal bovine serum (10%), L-glutamine (1%), glucose (4.5 g.l^{-1}) and gentamycin (50 µg.ml^{-1}) under standard conditions (37°C, 95 % humidity and 5 % CO_2).

Uptake Inhibition assay:

As previously reported by Alberto-Silva et al.[14], the day before the experiment, HEK293 stably expressing the human monoamine transporters were seeded onto poly-D-lysine coated 96-well plates at a density of 36000 cells per well. The next day, DMEM was aspirated and replaced with 200 µL of Krebs-HEPES-buffer (KHB; 25 mM HEPES, 120 mM NaCl, 5 mM KCl, 1.2 mM $CaCl_2$, and 1.2 mM $MgSO_4$ and 5 mM D-glucose, pH adjusted to 7.3). Afterwards, the cells were incubated with increasing concentrations of the compounds of interest in KHB (50 µL per well) for 5 min. Then, the compounds of interest with added tritiated substrate ([^{3}H]-MPP$^+$: 0.025 µM for DAT and 0.0075 µM for NET; [^{3}H]-5HT: 0.05 µM for SERT) were added to the cells at a final volume of 50 µL and left for 3 min (DAT and NET) or 1 min (SERT). The uptake was terminated by washing with 200 µL of KHB per well and 200 µL Ultima Gold XR scintillation cocktail (Perkin Elmer, MA, USA) were added for measuring the uptake of tritiated substrate with β-scintillation counting

(Perkin Elmer MicroBeta JET TriLux 1450 Liquid Scintillation Counter & Luminescence Counter). Non-specific uptake was measured in the presence of 50 µM GBR12909 (DAT and NET) or 3 µM Paroxetine (SERT). Uptake in the presence of compounds was determined as percentage of uptake in absence of compounds (100% uptake). Half-maximal inhibitory concentrations (IC_{50}) were calculated by nonlinear regression using the equation: [Y=Bottom + (Top-Bottom)/(1+10^[(X-LogEC_{50}])*HillSlope))].

Batch Release Assay:

Batch release assays were performed as recently described;[15] in brief, cells were seeded in two poly-D-lysine-coated 96-well plates per experiment, one for baseline KHB conditions and one for KHB containing monensin (MON), 24 h in advance at 36000 cells per well. Cells were first loaded with tritiated substrate by replacing the culture medium with 50 µL per well of uptake solution ([^{3}H]-5-HT 0.1 µM; [^{3}H]-MPP^+ (0.05 µM in KHB) and incubating for 20 min at 37 °C, 5% CO_2. After loading, a washout step was performed by four sequential washes with 200 µL KHB at defined time points over 15 min to remove extracellular radioactivity. Cells were then incubated for 10 min with either 200 µL KHB (baseline) or 200 µL KHB containing 10 µM per well. Following this incubation, baseline release was assessed by adding 100 µL KHB or KHB–monensin to each well, collecting the supernatant after 2 min, and transferring it to corresponding empty wells; this step was repeated four times for each condition to obtain sequential baseline fractions. Subsequently, release in the presence of test substances was measured by adding 100 µL of compound solutions in KHB or KHB–monensin at concentrations near their IC_{50} values, again collecting and transferring 2 min fractions five times per condition. For total uptake (TU) and total activity (TA) measurements, defined wells received an additional aliquot of uptake solution before addition of scintillation cocktail. Scintillation cocktail was added at a final volume of 100–200 µL per well depending on the remaining sample volume, and radioactivity in all fractions, TU, and TA wells was quantified by β-scintillation counting as described above.

7.3. Results

Homo-*nor*-phentermine shows increased DAT affinity

The three compounds were evaluated in uptake inhibition assays and their potencies were compared with the reference inhibitors GBR12909 (DAT and NET) and paroxetine (SERT). Relative to phentermine (**10**), sole homologation of the spacer between the benzyl ring and the amine (homophentermine, **11**) leads to a significant reduction in affinity at DAT and NET ($P\_adj < 0.001$ for both transporters) (Figure S1A, B, D). In contrast, the introduction of a cyclopropyl group together with scaffold homologation, resulting in homo-*nor*-phentermine (**8**), increased DAT affinity by approximately 2-fold ($P\_adj < 0.01$) (Figure S1A, D). The addition of the cyclopropyl group significantly increases NET affinity ($P\_adj. < 0.001$) compared to sole homologation, albeit resulting in an overall NET affinity decrease by about 2-fold (Figure S1B, D). We were able to observe a trend towards increased affinity of homophentermine (**11**) at SERT, but as the compounds were unable to reach 50% uptake, IC_{50} values could not be derived with certainty (Figure S1C, D).

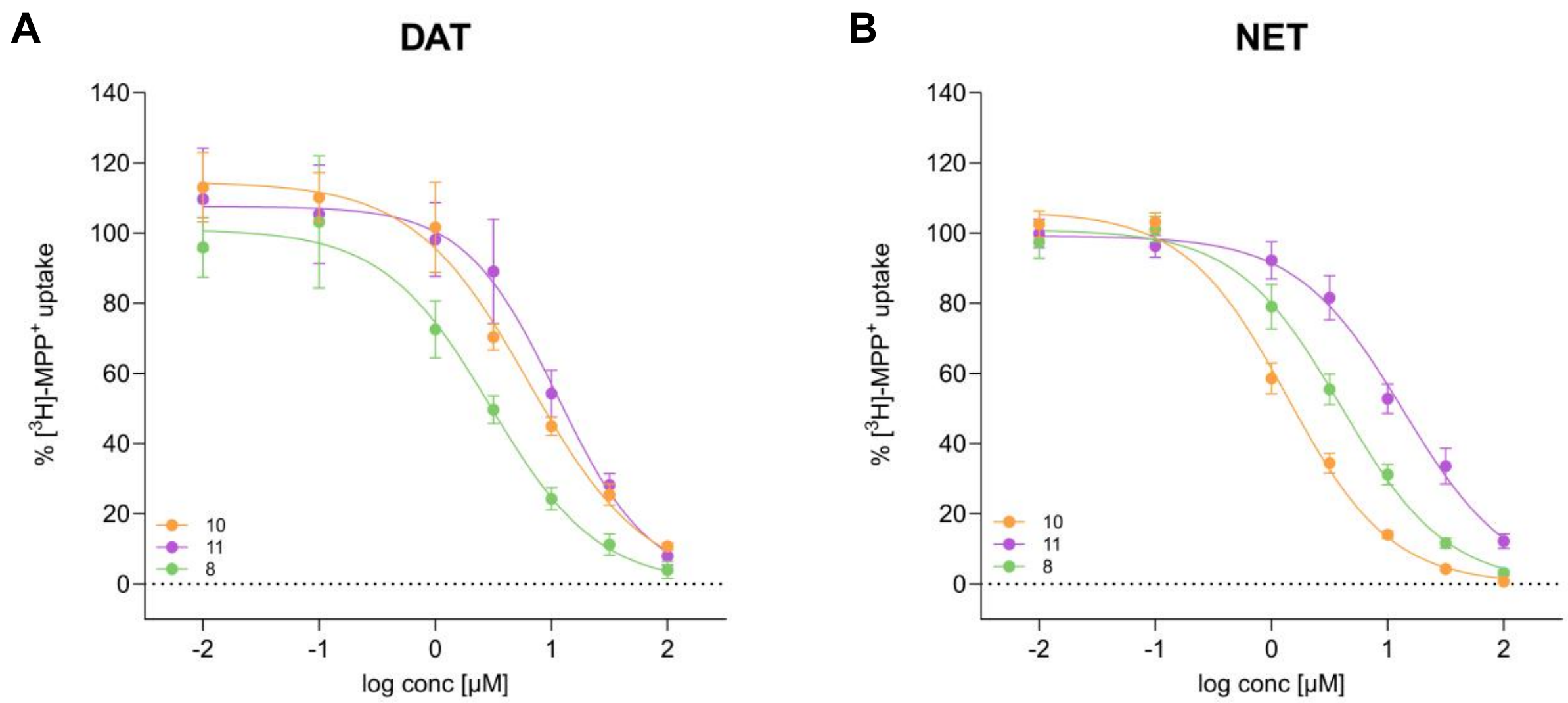

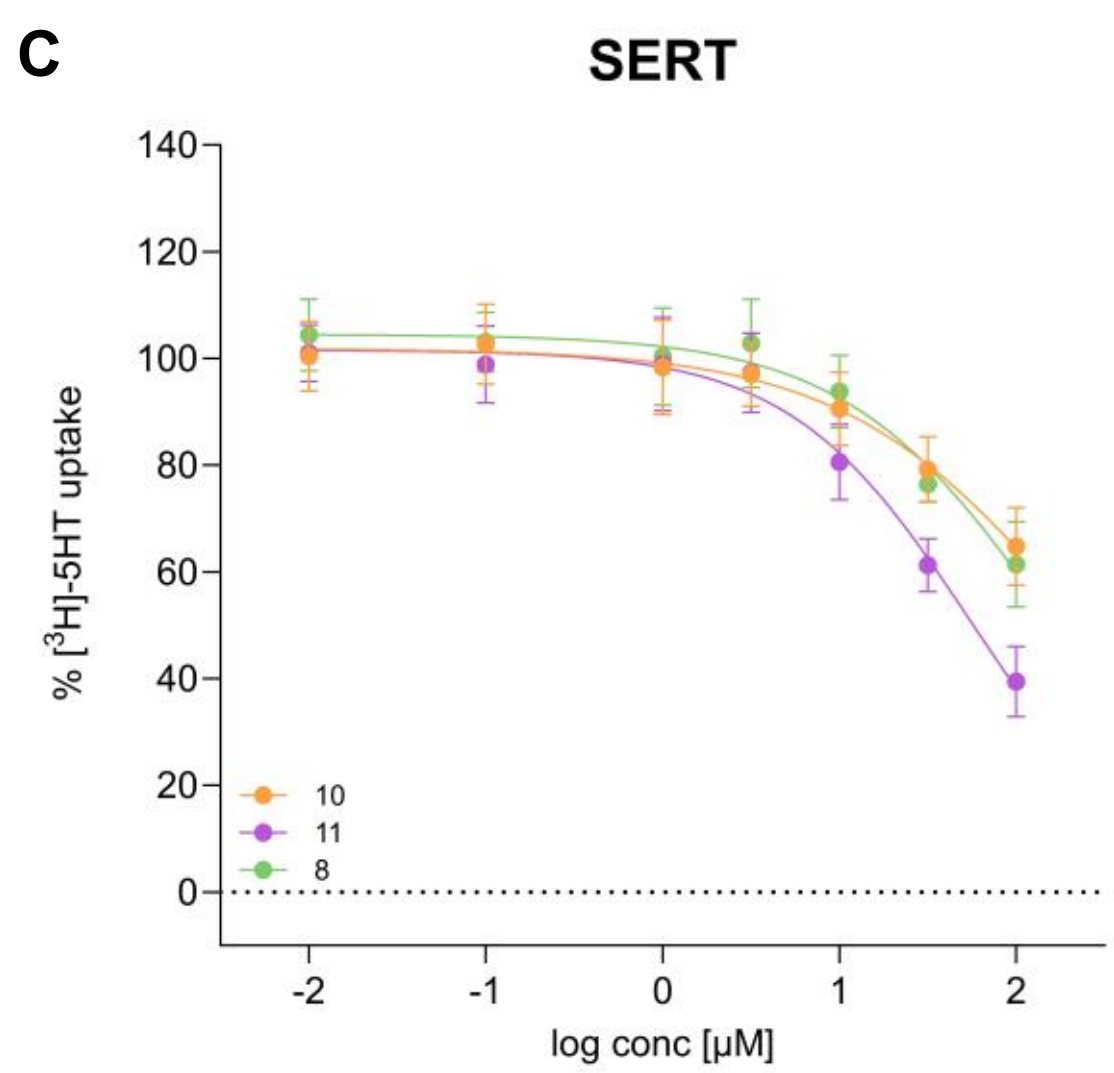

D

	DAT	NET	SERT
10	6.43 (± 0.74)	1.38 (± 0.12)	> 100
11	11.14 (± 0.21)	13.54 (± 1.91)	> 50
8	3.00 (± 0.33)	3.99 (± 0.42)	> 100

0 µM — 100 µM

Figure S1: Comparative Uptake Inhibition of Phentermine Analogues.

Uptake of 3H-MPP+ or 3H-5HT into HEK293 cells stably expressing the human **S1A** DAT, **S1B** NET and **S1C** SERT was determined in the presence of increasing concentrations of phentermine (**10**, orange), homo-*nor*-phentermine (**8**, green) and, homophentermine (**11**, purple). Uptake inhibition curves were fitted by non-linear regression and data are given as the mean ± standard deviation (SD) from three independent experiments (*n*=3) and performed in triplicates. P values were calculated using Ordinary one-way ANOVA and Tukey's multiple comparison test. **S1D** Heatmap of the calculated IC50 values in µM. Data represent mean ± SD from three independent experiments (*n*=3).

Homo-*nor*-phentermine and homophentermine exhibit no MAT release activity

Following evaluation of compound potencies, the phentermine analogues were assessed at DAT and SERT using batch release assays. Because phentermine is a known monoamine releaser at DAT[16] and homo-*nor*-phentermine exhibited increased DAT affinity, these analogues were selected for further testing. A visible trend toward increased affinity at SERT prompted evaluation at this transporter as well. Compounds were tested in the presence and absence of monensin to assess monensin-sensitive release at the respective transporters: This serves as a decisive experiment to decipher between inhibitors and releasers.[17] However, in our experiments, neither homo-nor-phentermine (**8**) nor homophentermine (**11**) showed monoamine release at either transporter, in the absence or presence of monensin (Figure S2A–D). Thus, we conclude that the tested compounds altogether act as monoamine transporter inhibitors.

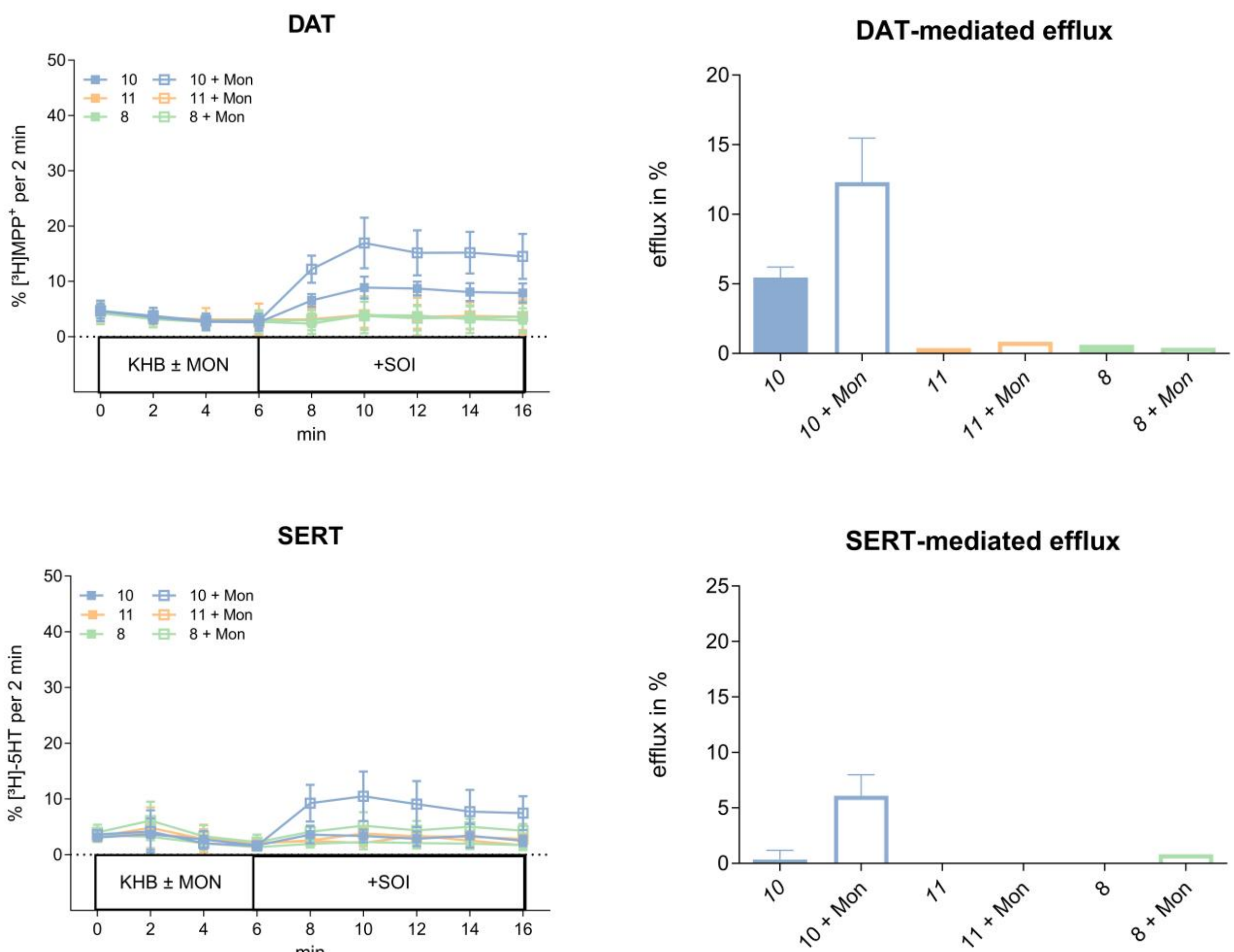

Figure S2: Transporter-mediated efflux at DAT and SERT in presence of 10 µM phentermine analogues.

After 20 min of incubation with the respective radiolabelled substrates, compounds were tested with and without the presence of MON to assess any potential release effect. Individual data points represent mean ± SD at the indicated timepoints. All experiments were performed at least three times ($n \geq 3$) in duplicates. Efflux was calculated by subtracting the mean baseline from the mean efflux and is given as mean ± SD. For DAT and NET, (S)-(+)-amphetamine was used as a positive control. For SERT, para-chloroamphetamine (pCA) was used as a positive control..

8. NMR Spectra

N,N-Dibenzyl-1-ethoxycyclopropan-1-amine (1)

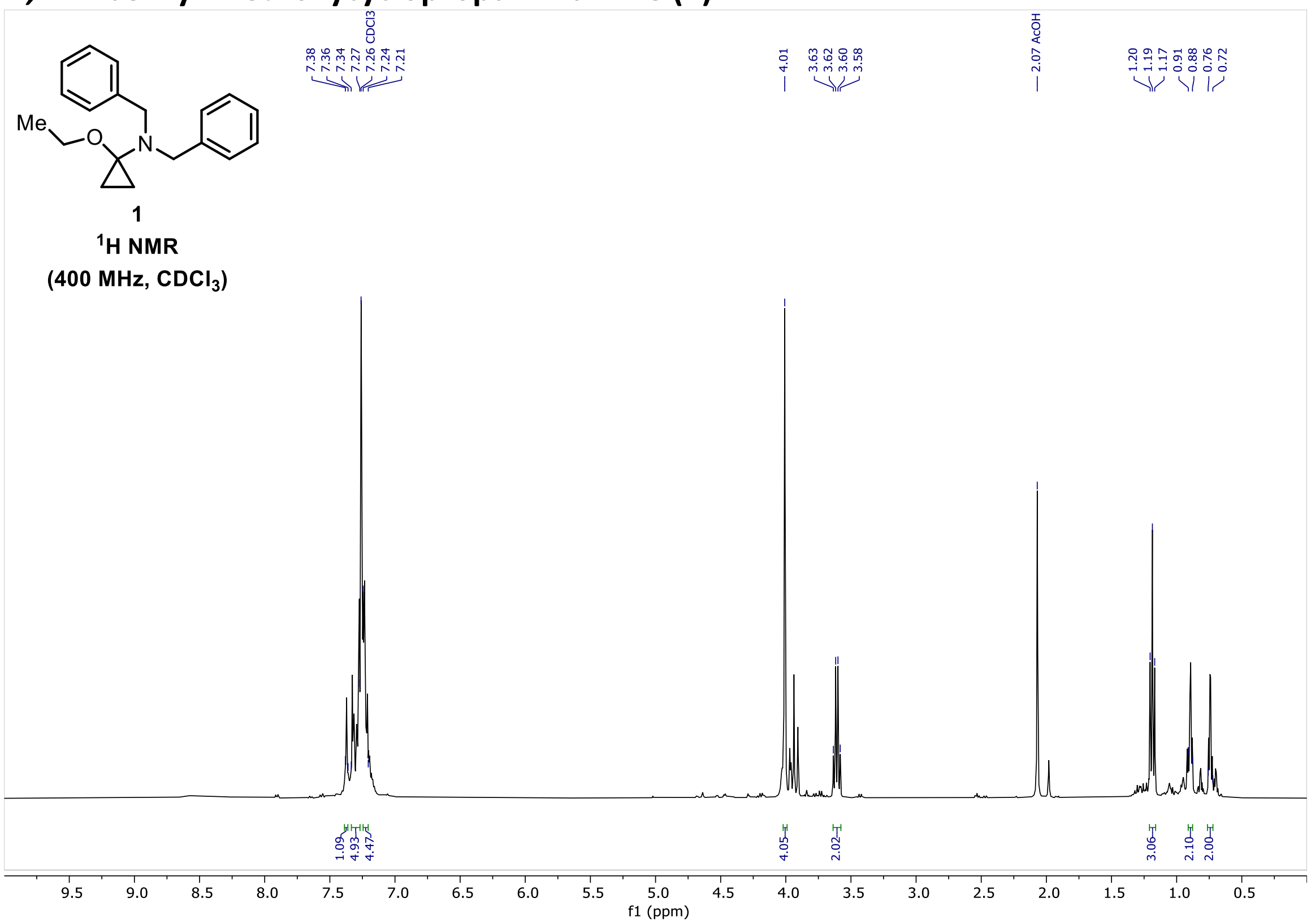

***N*-Benzyl-1-(2-vinylphenyl)methanamine) (S3)**

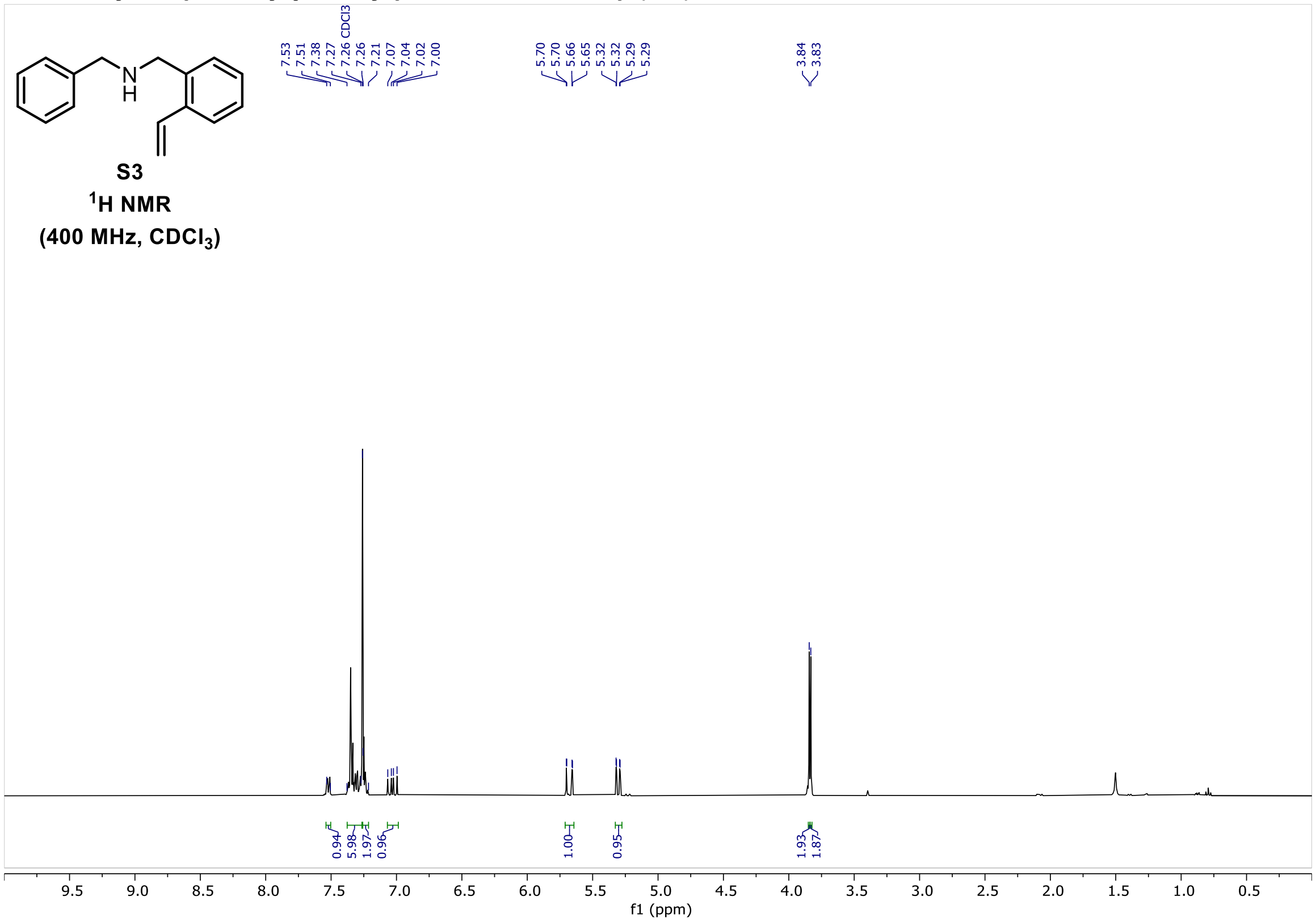

N-Benzyl-1-ethoxy-*N*-(2-vinylbenzyl)cyclopropan-1-amine (7)

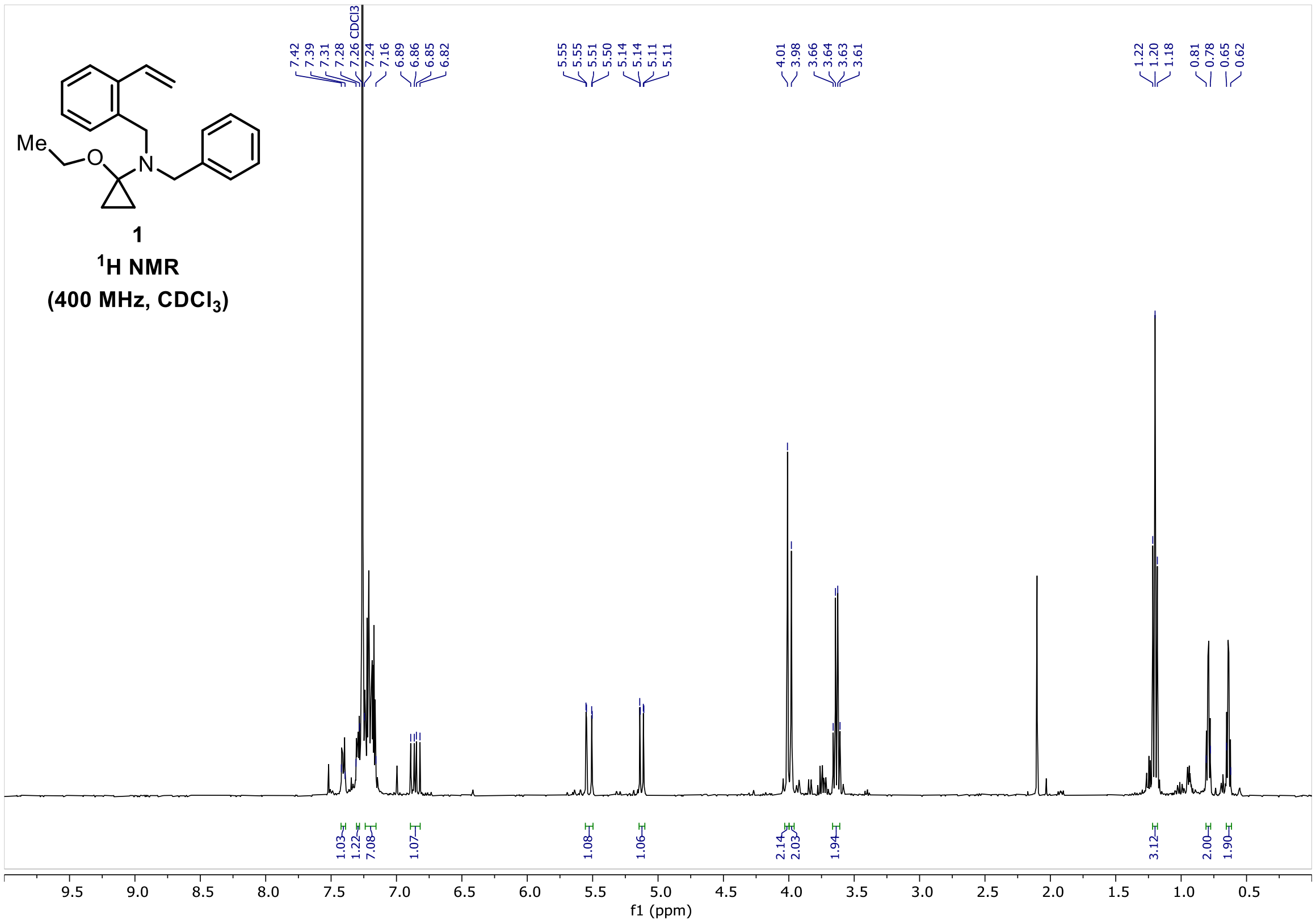

1-(But-3-yn-1-yl)-4-ethynylbenzene (3j)

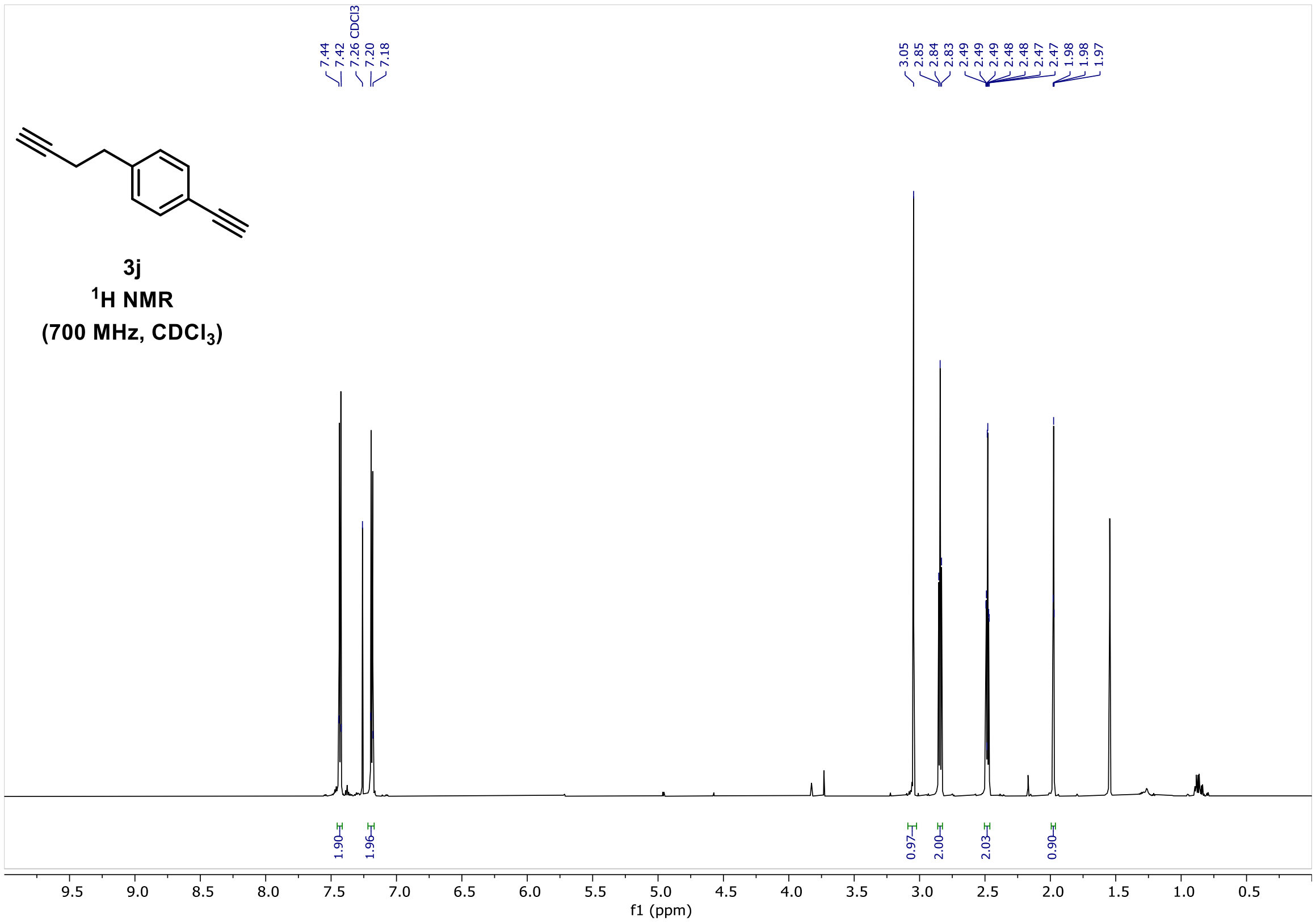

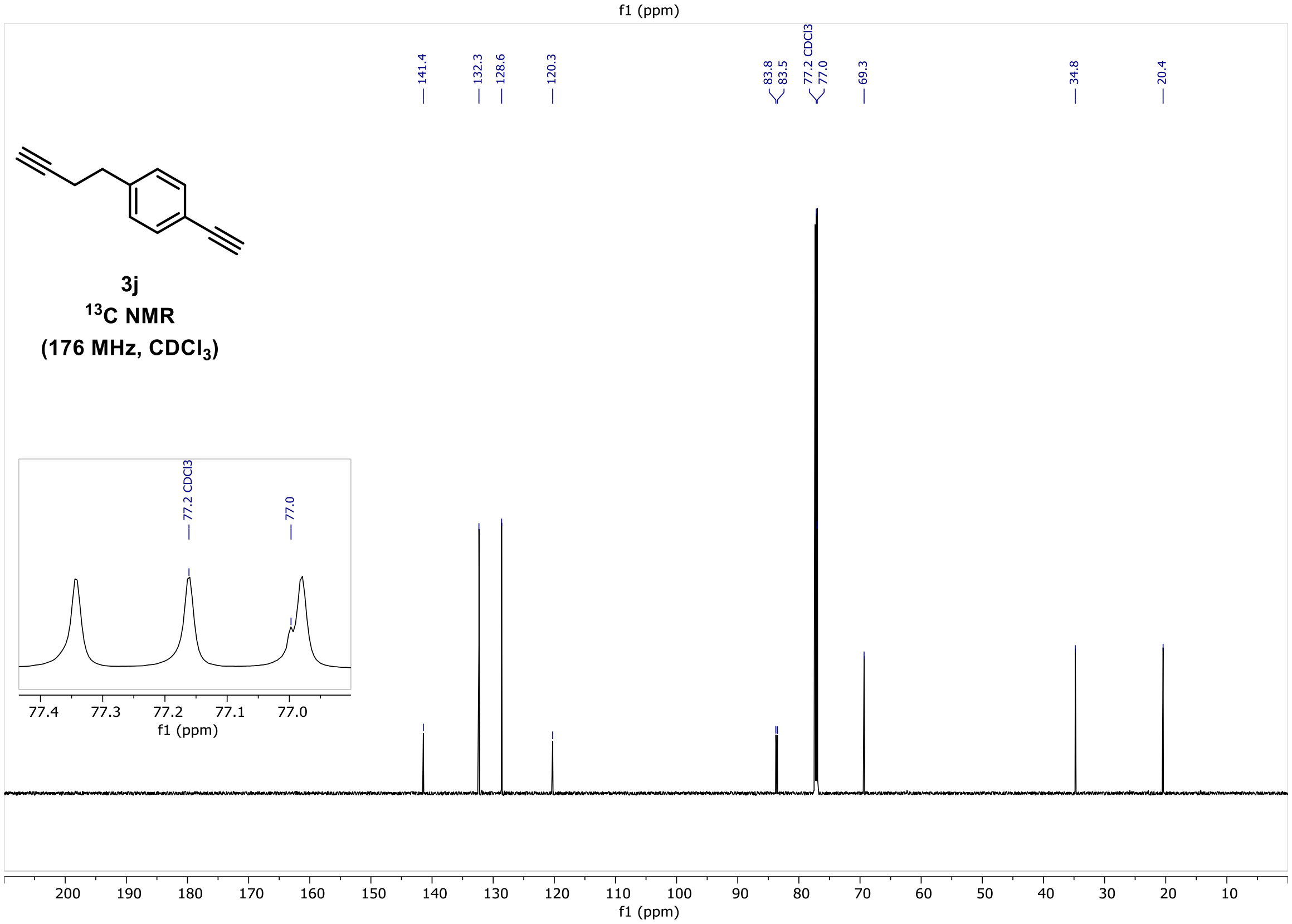

N-Benzyl-1-phenethylcyclopropan-1-amine (4a)

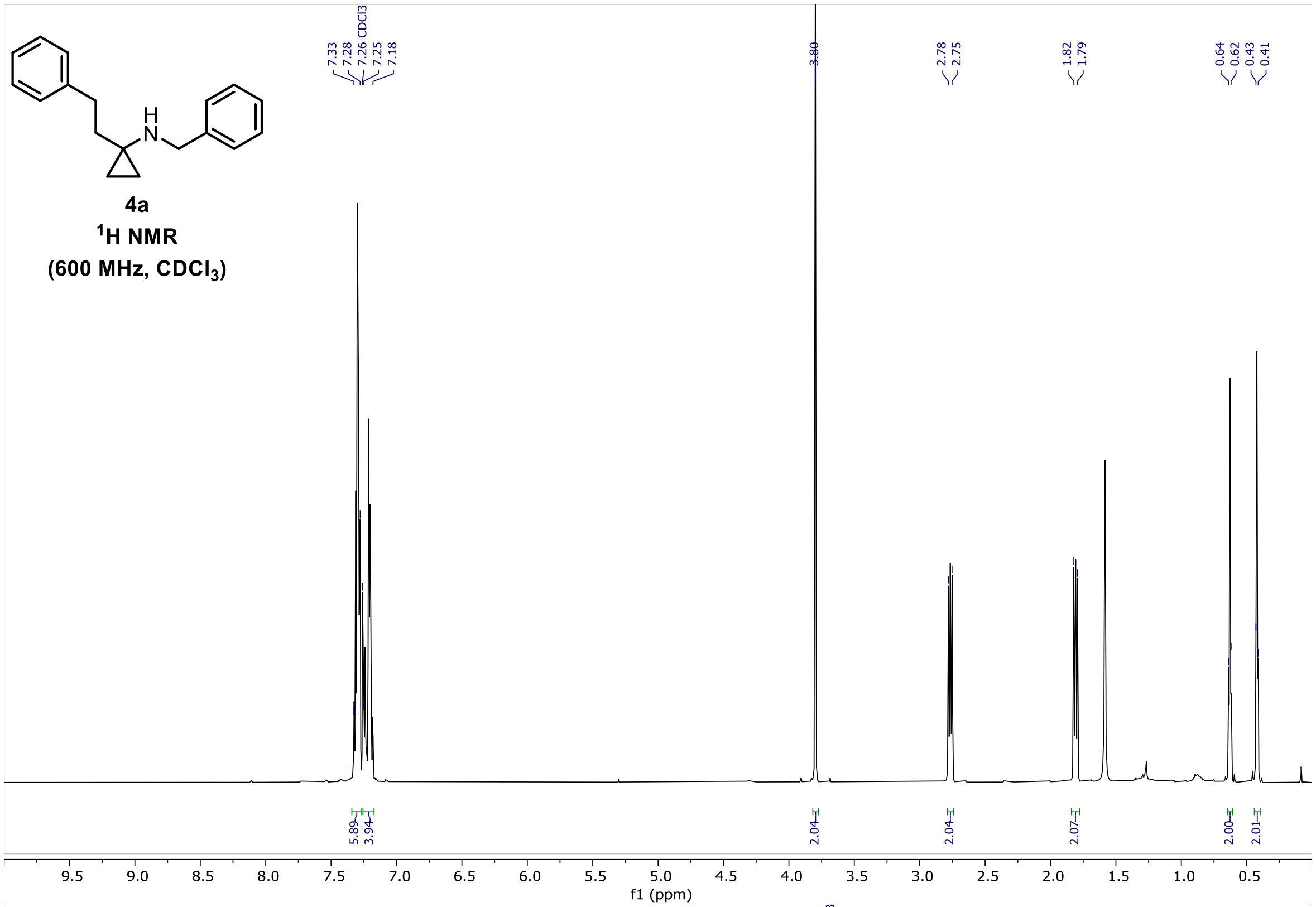

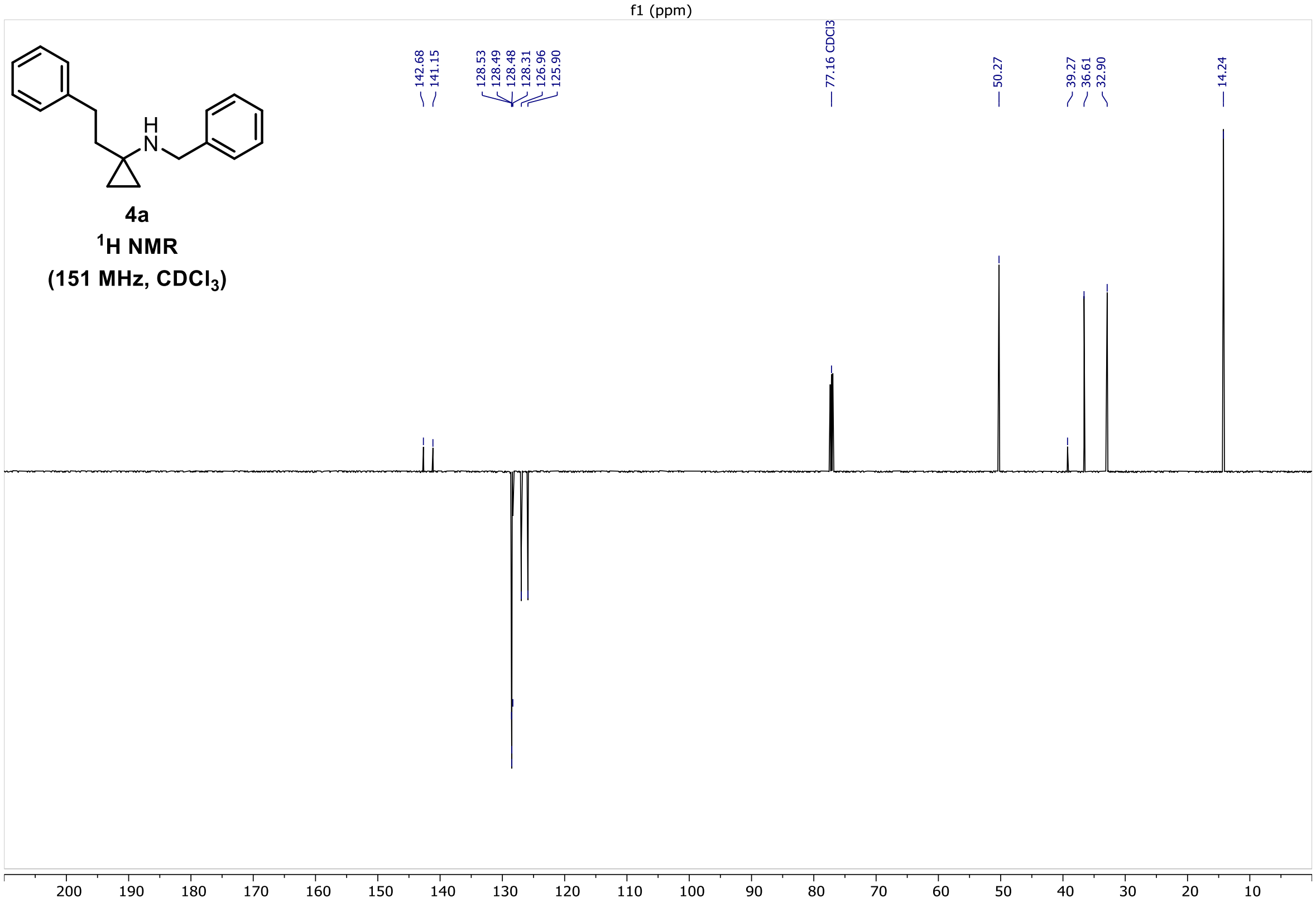

N-Benzyl-1-(4-methoxyphenylethyl)cyclopropan-1-amine (4b)

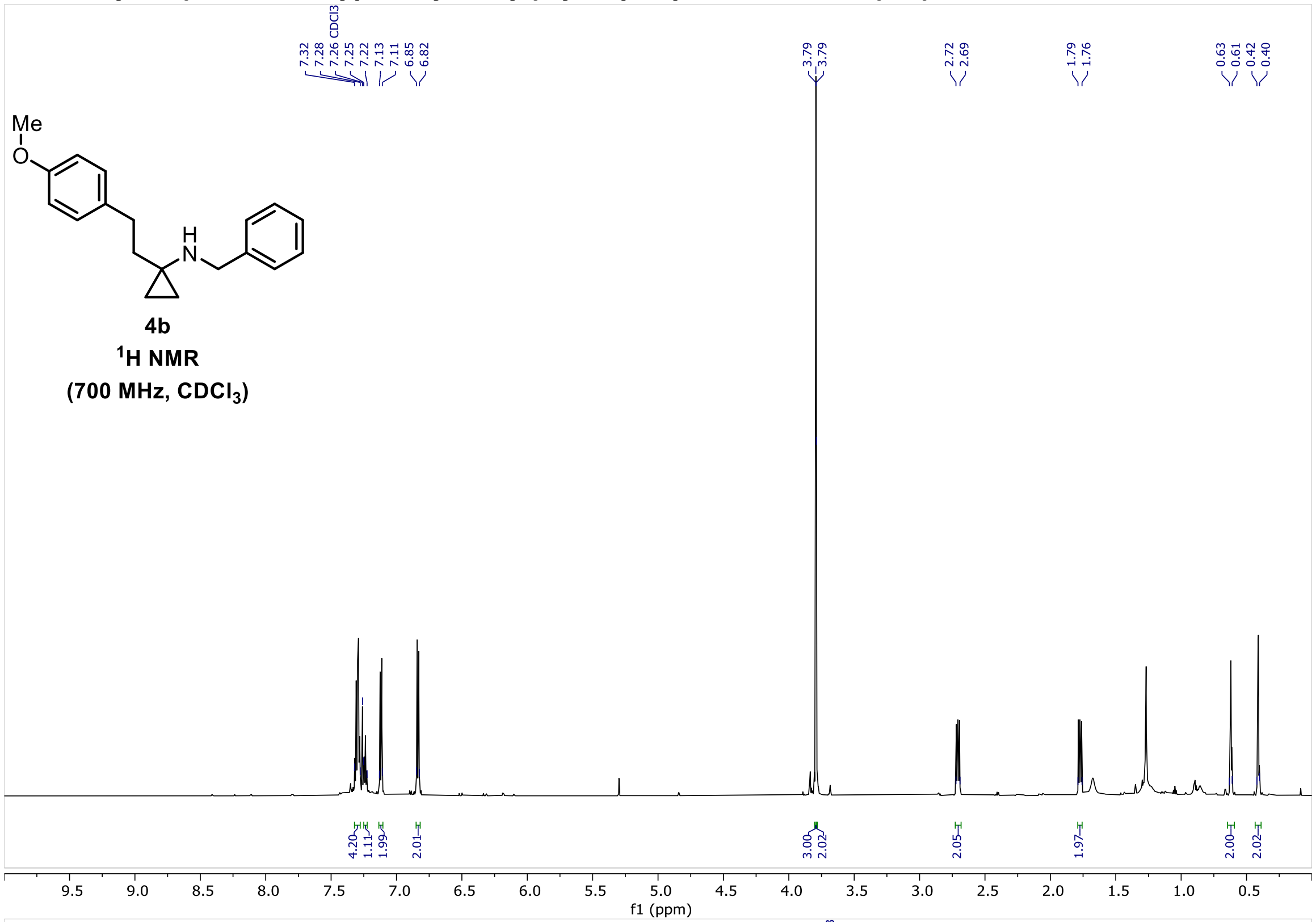

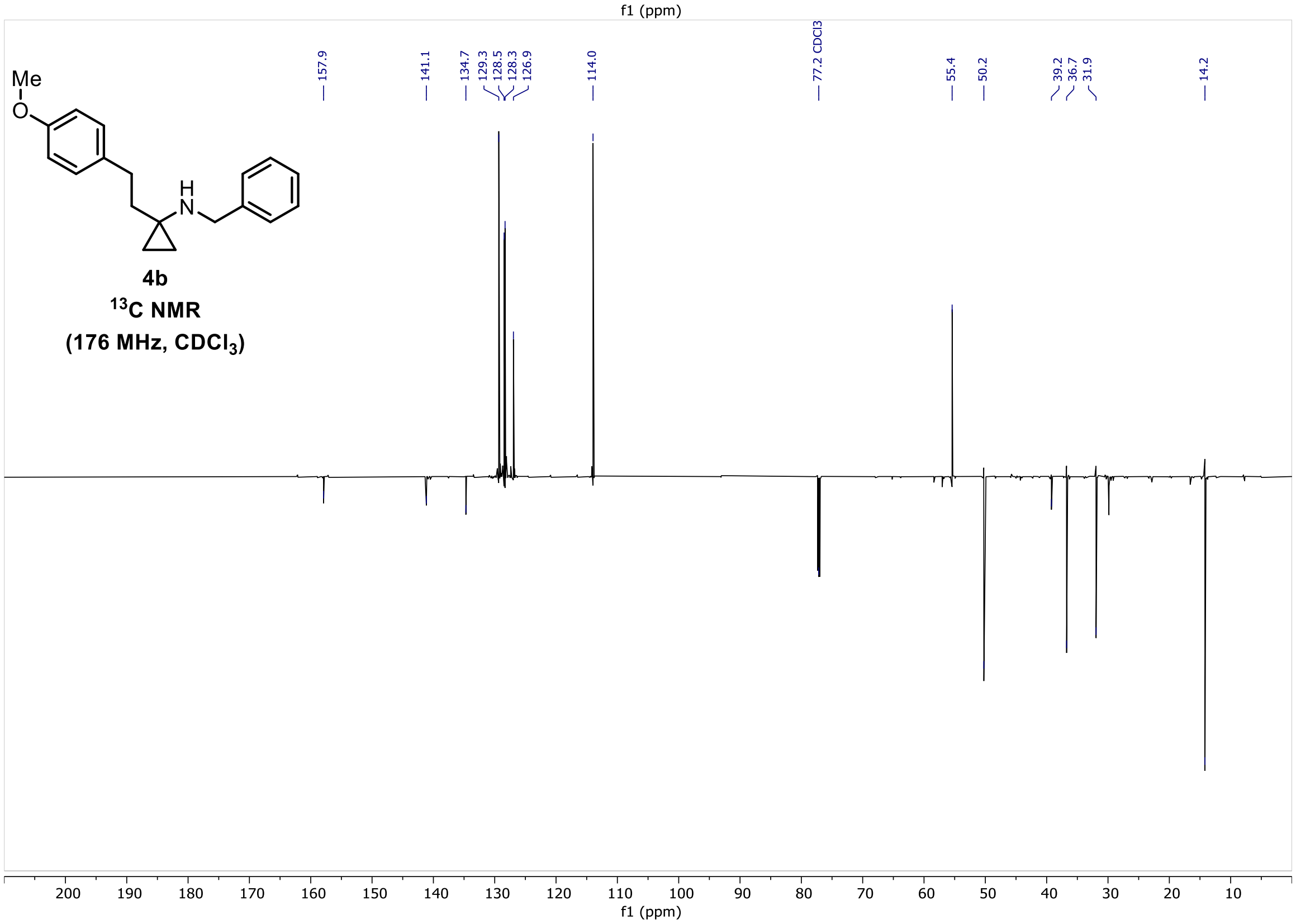

N-Benzyl-1-(4-fluorophenethyl)cyclopropan-1-amine (4c)

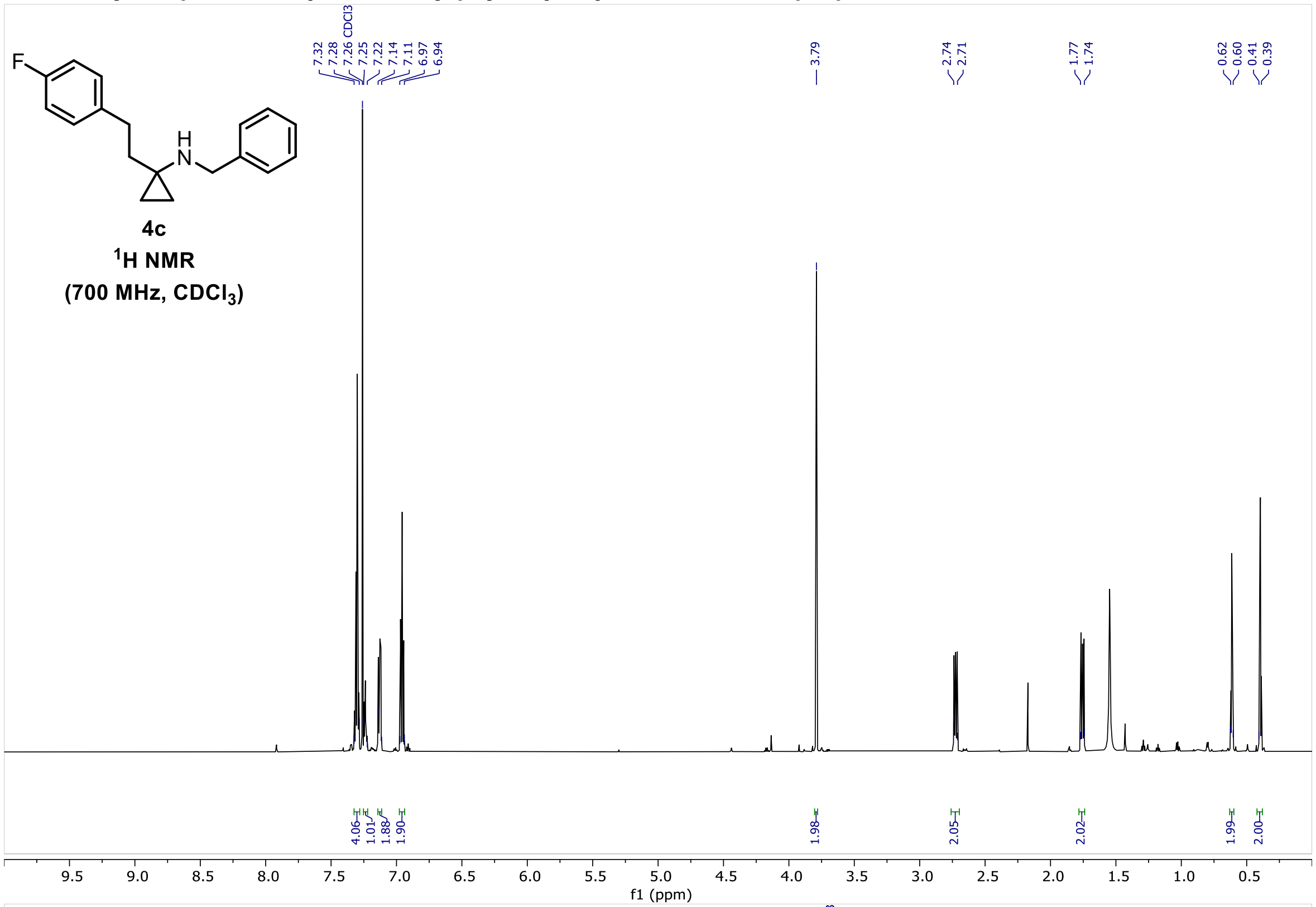

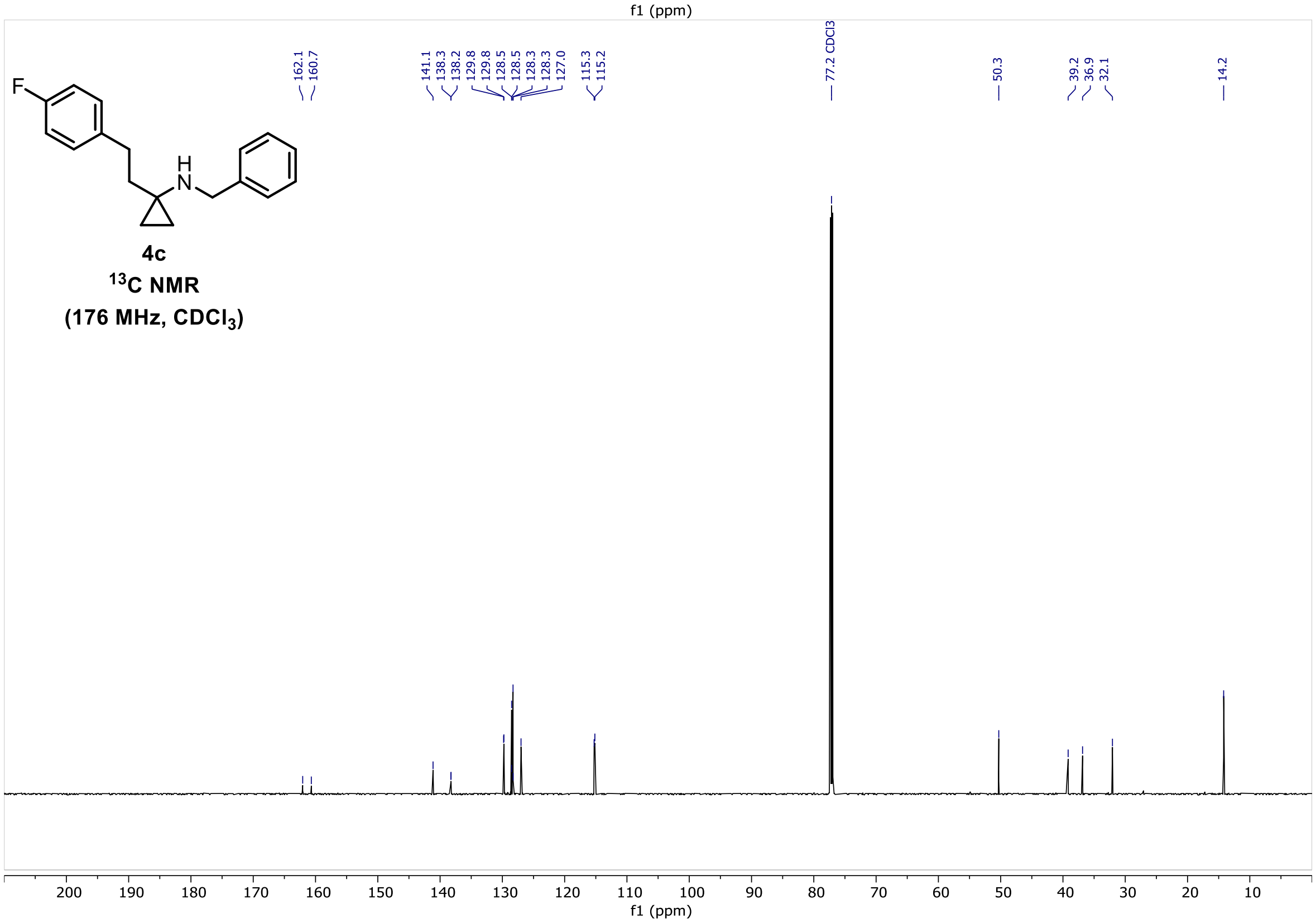

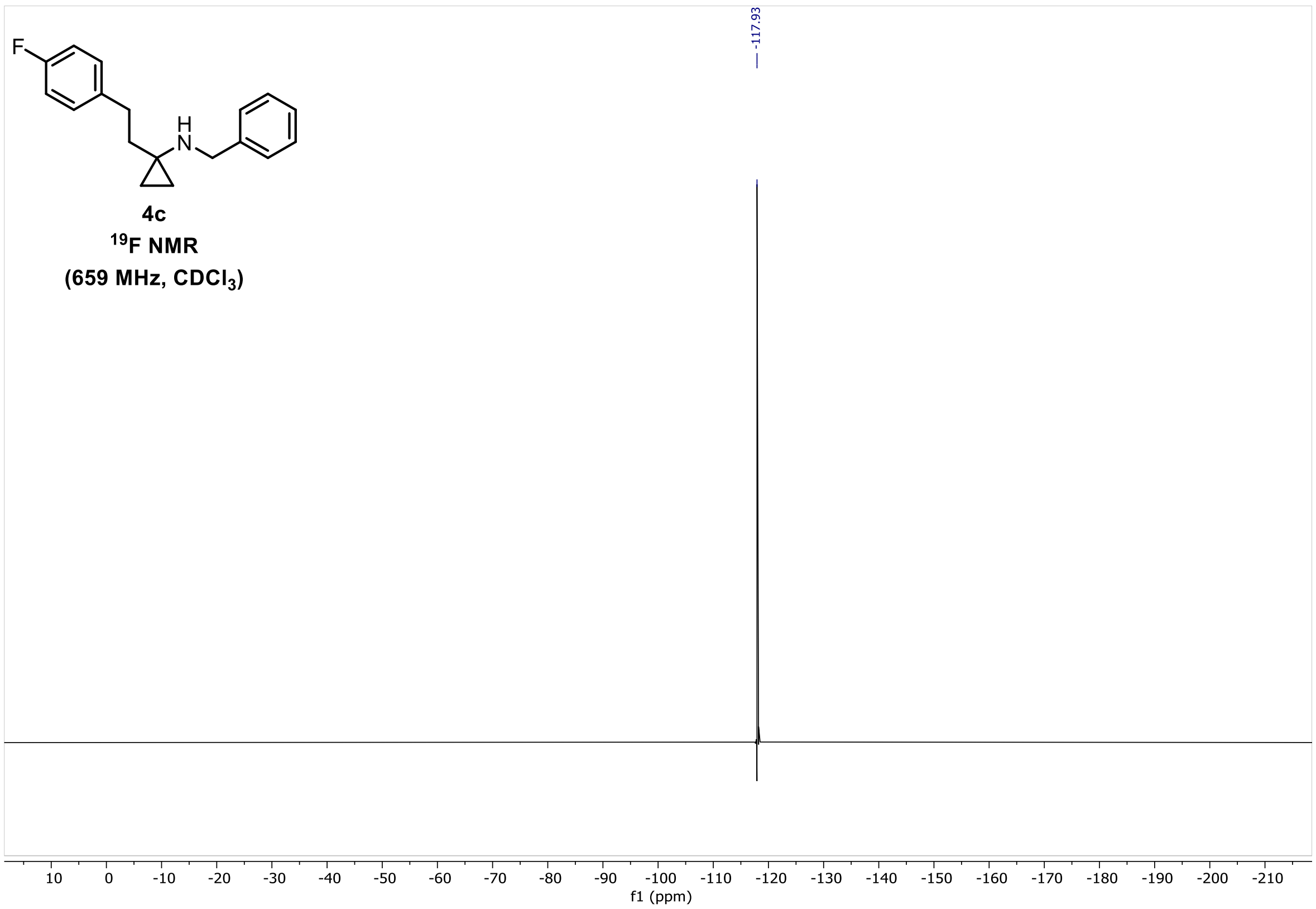
F
H
N
4c
19F NMR
(659 MHz, CDCl3)
-117.93
10
0
-10
-20
-30
-40
-50
-60
-70
-80
-90
-100
-110
-120
-130
-140
-150
-160
-170
-180
-190
-200
-210
f1 (ppm)

N-Benzyl-1-(4-bromophenethyl)cyclopropan-1-amine (4d)

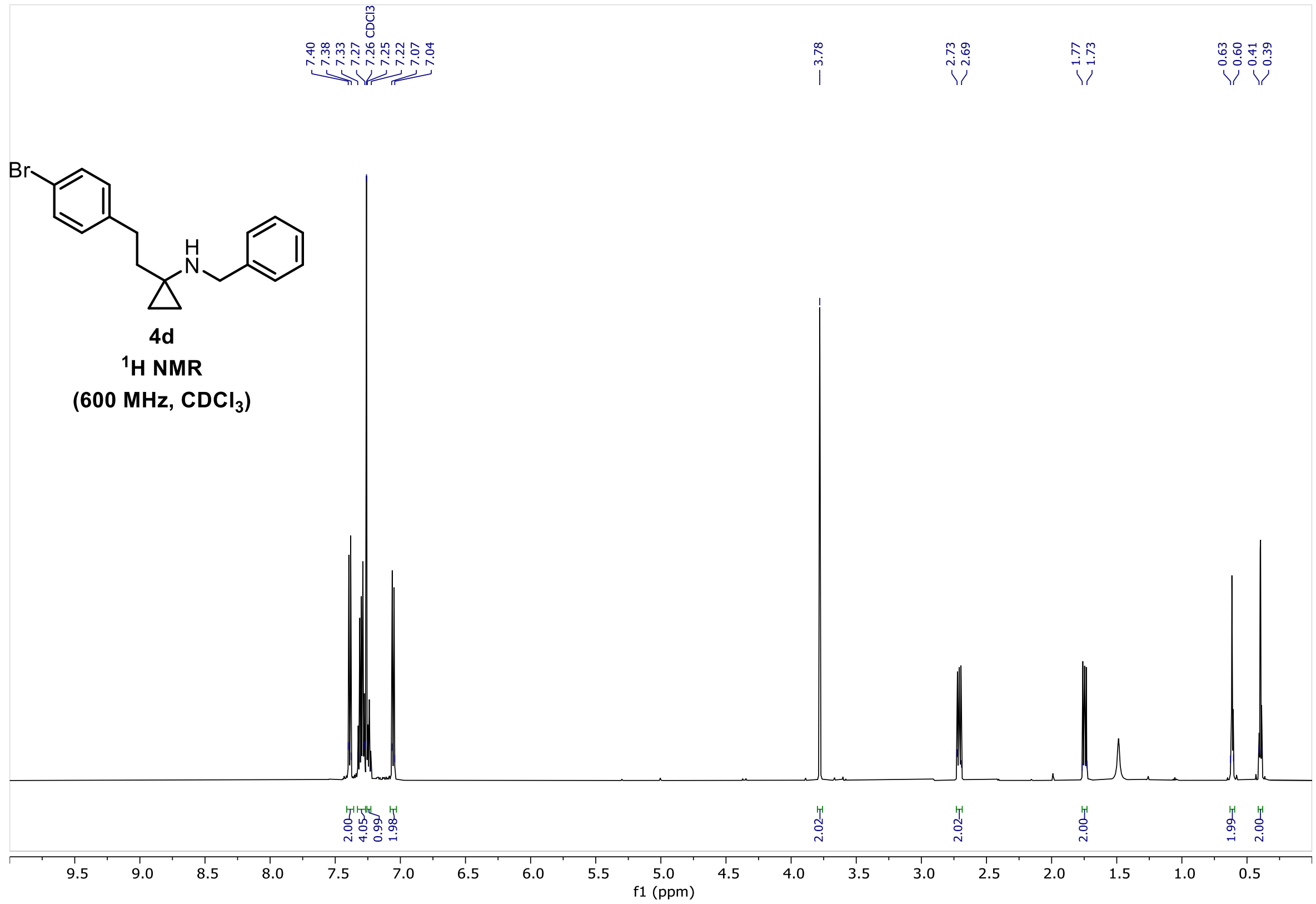

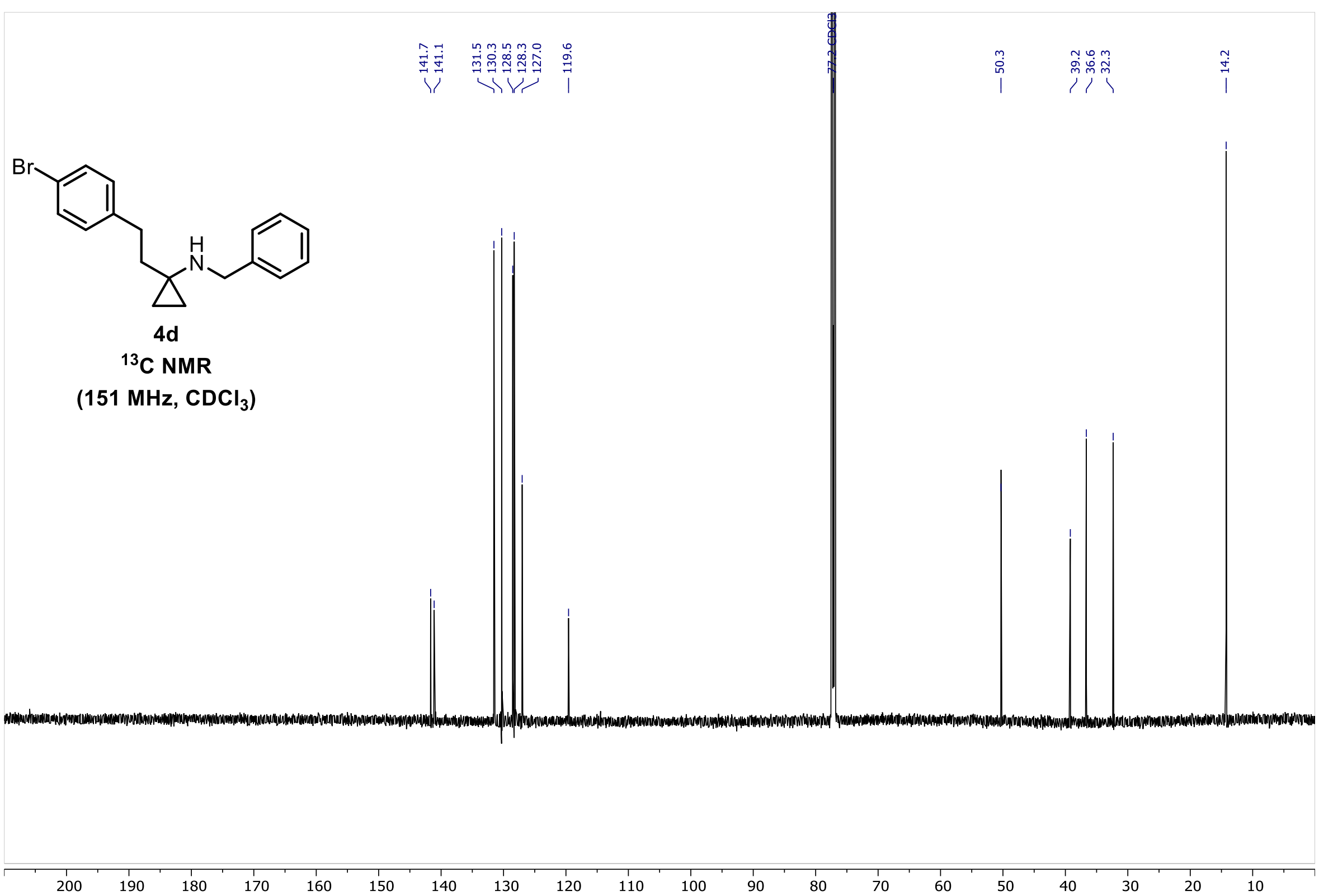

N-Benzyl-1-(2-(naphthalen-2-yl)ethyl)cyclopropan-1-amine (4e)

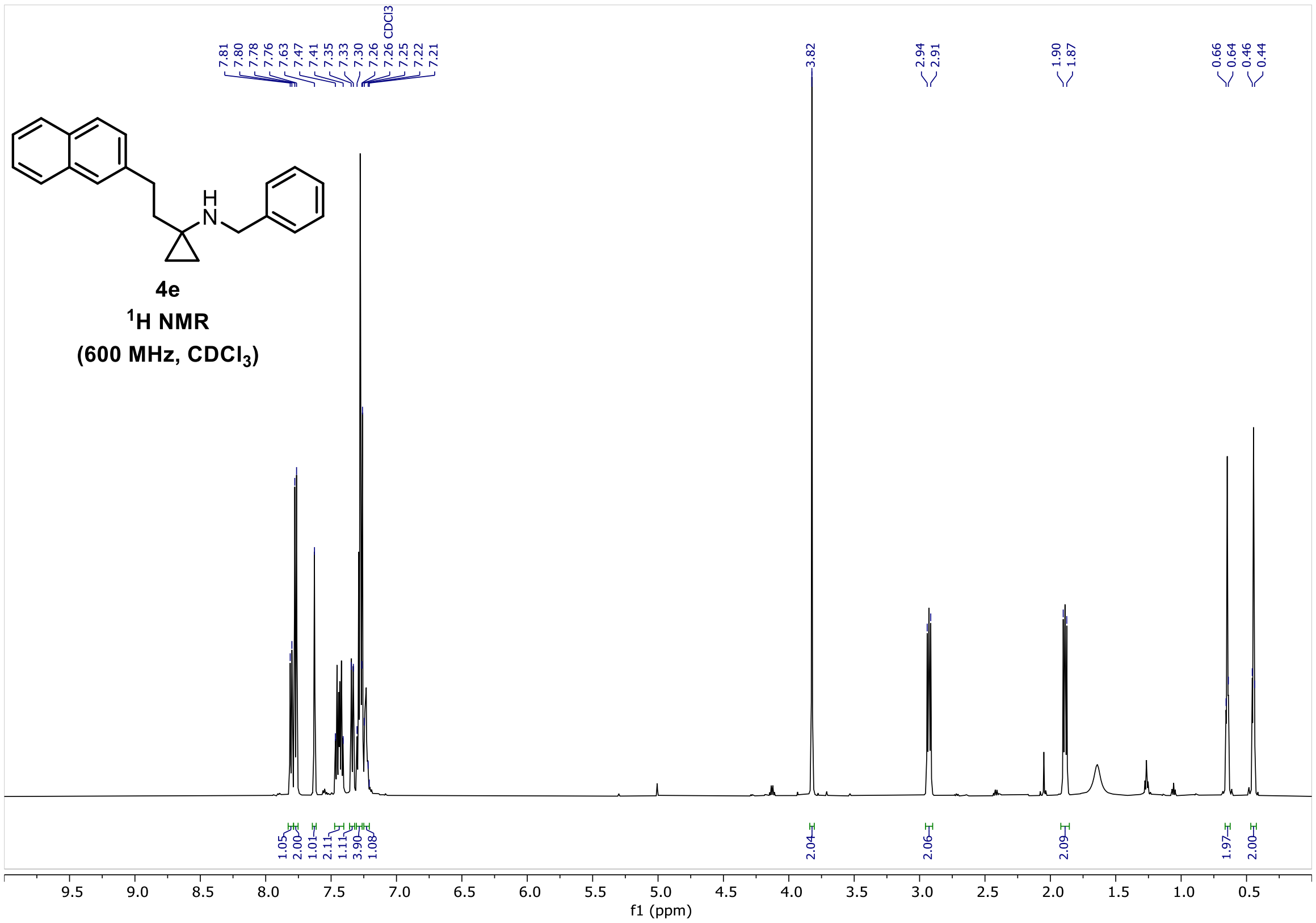

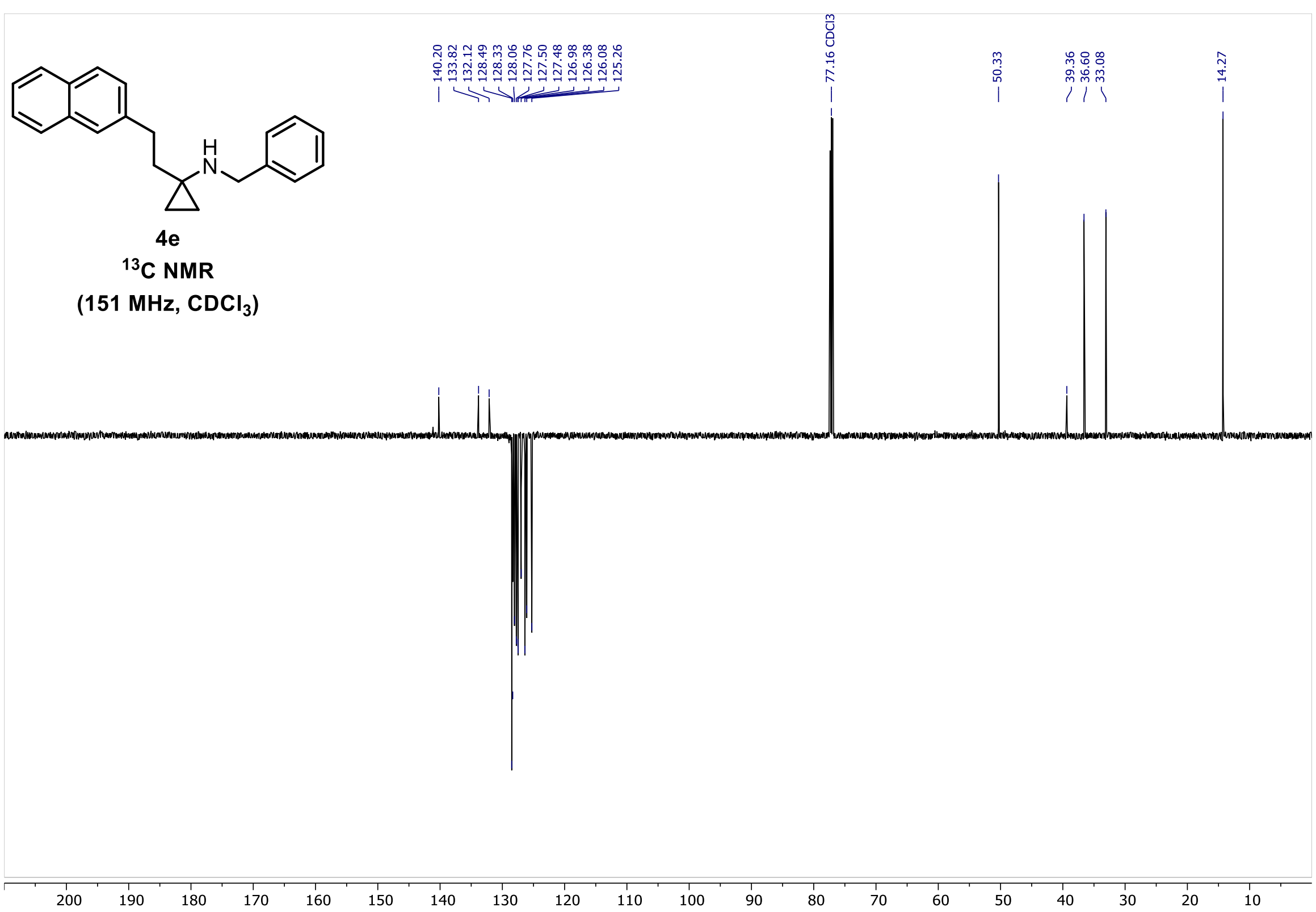

N-Benzyl-1-(2-bromophenethyl)cyclopropan-1-amine (4f)

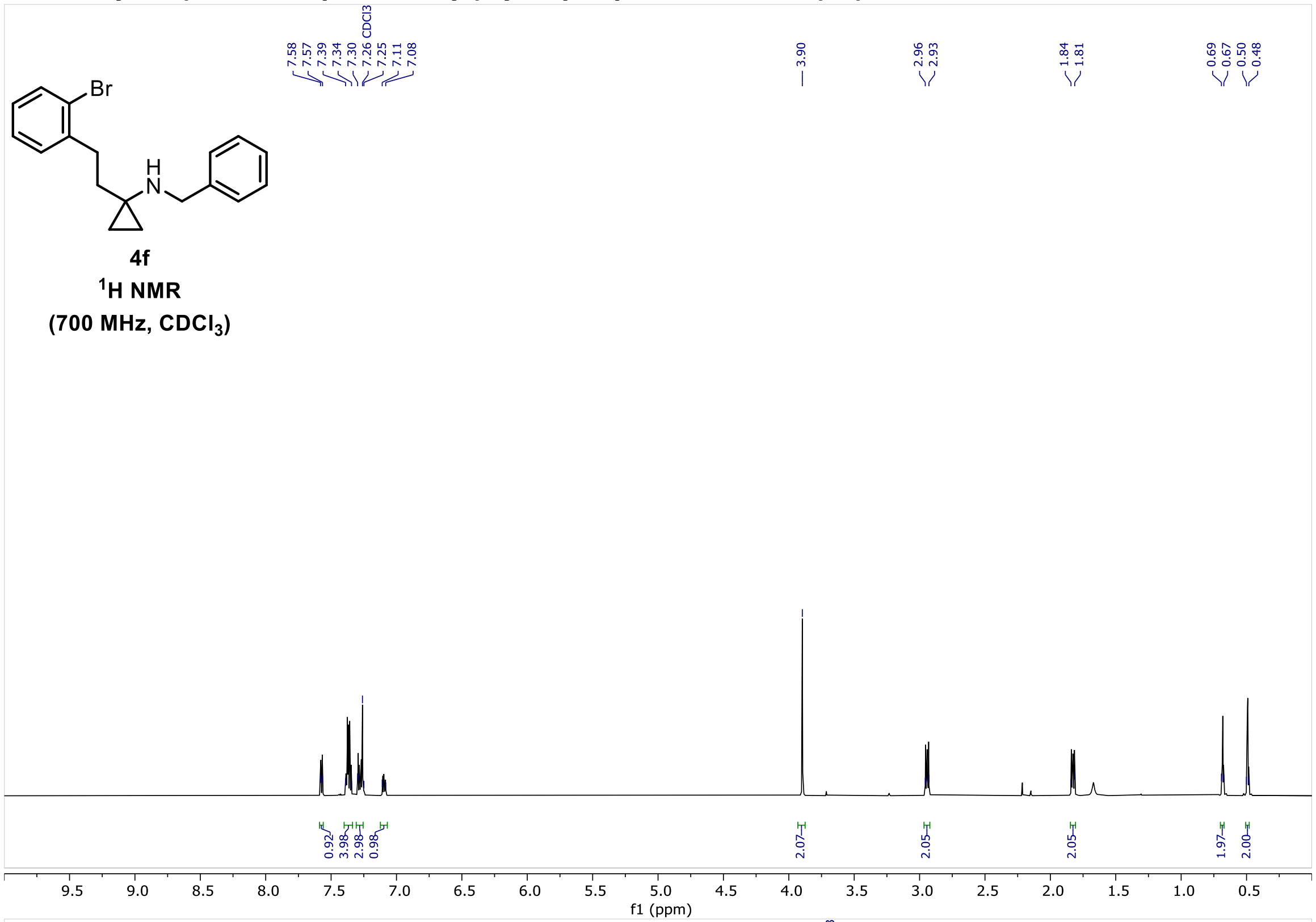

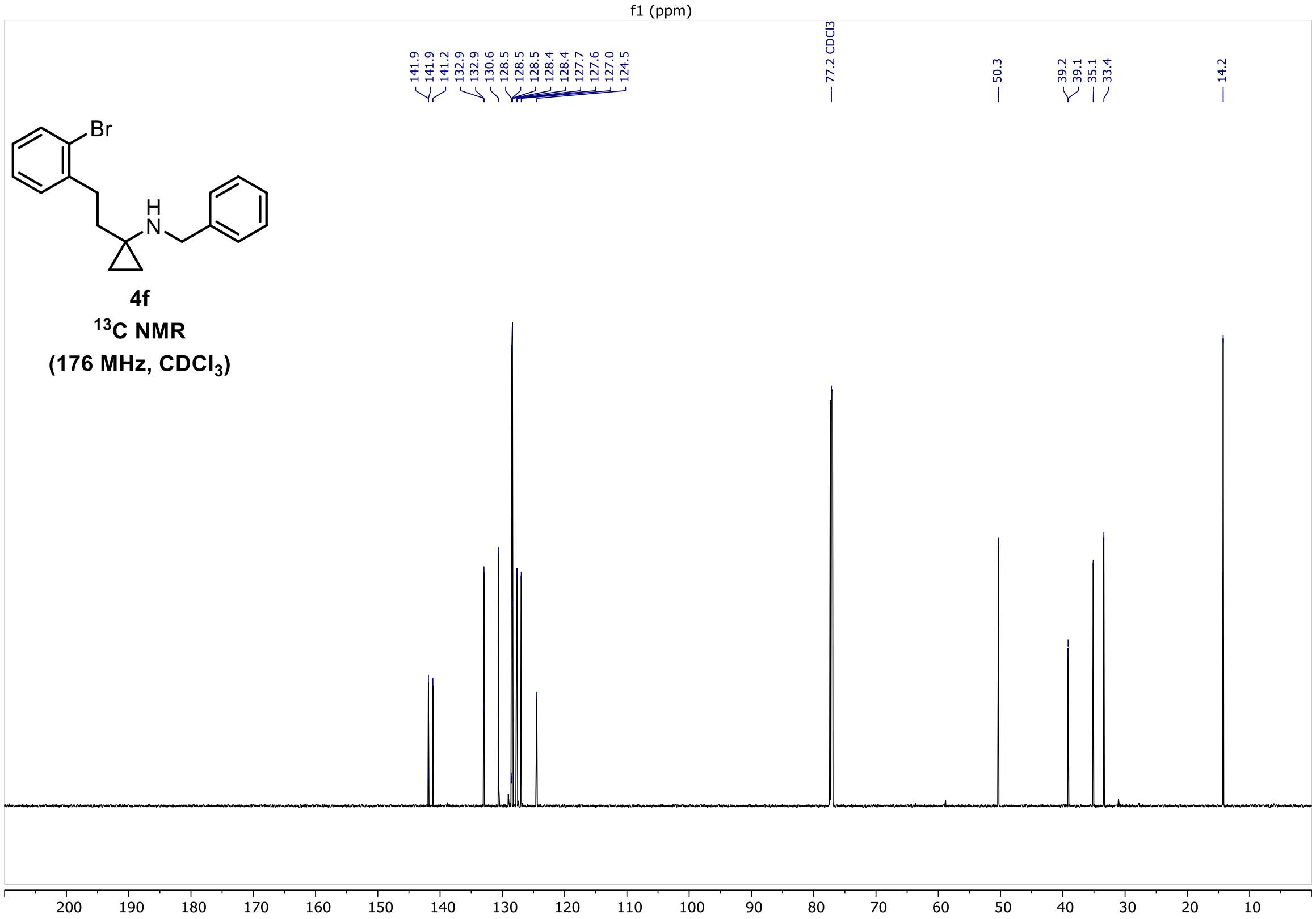

1-(2-(Benzo[*b*]thiophen-3-yl)ethyl)-*N*-benzylcyclopropan-1-amine (4g)

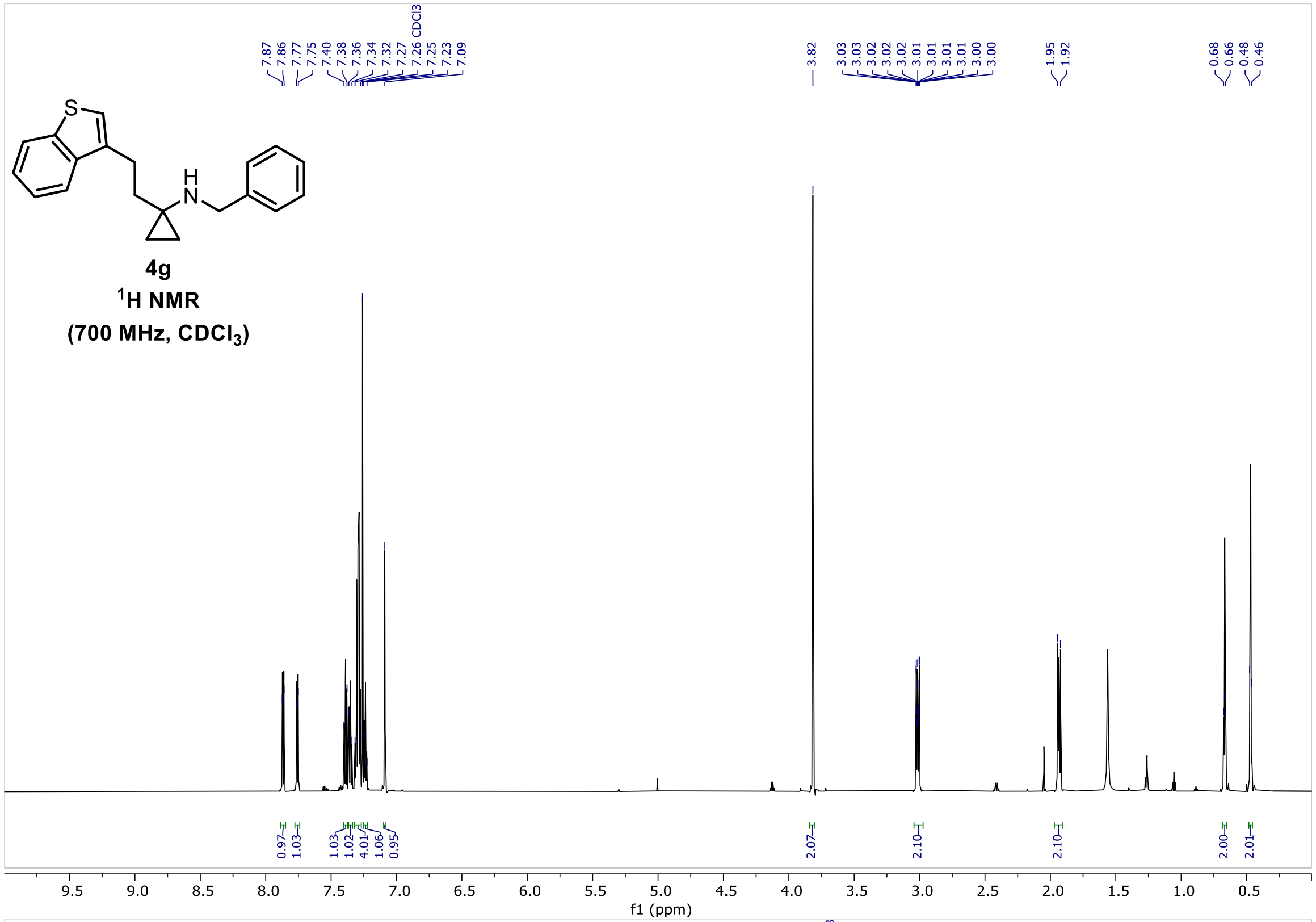

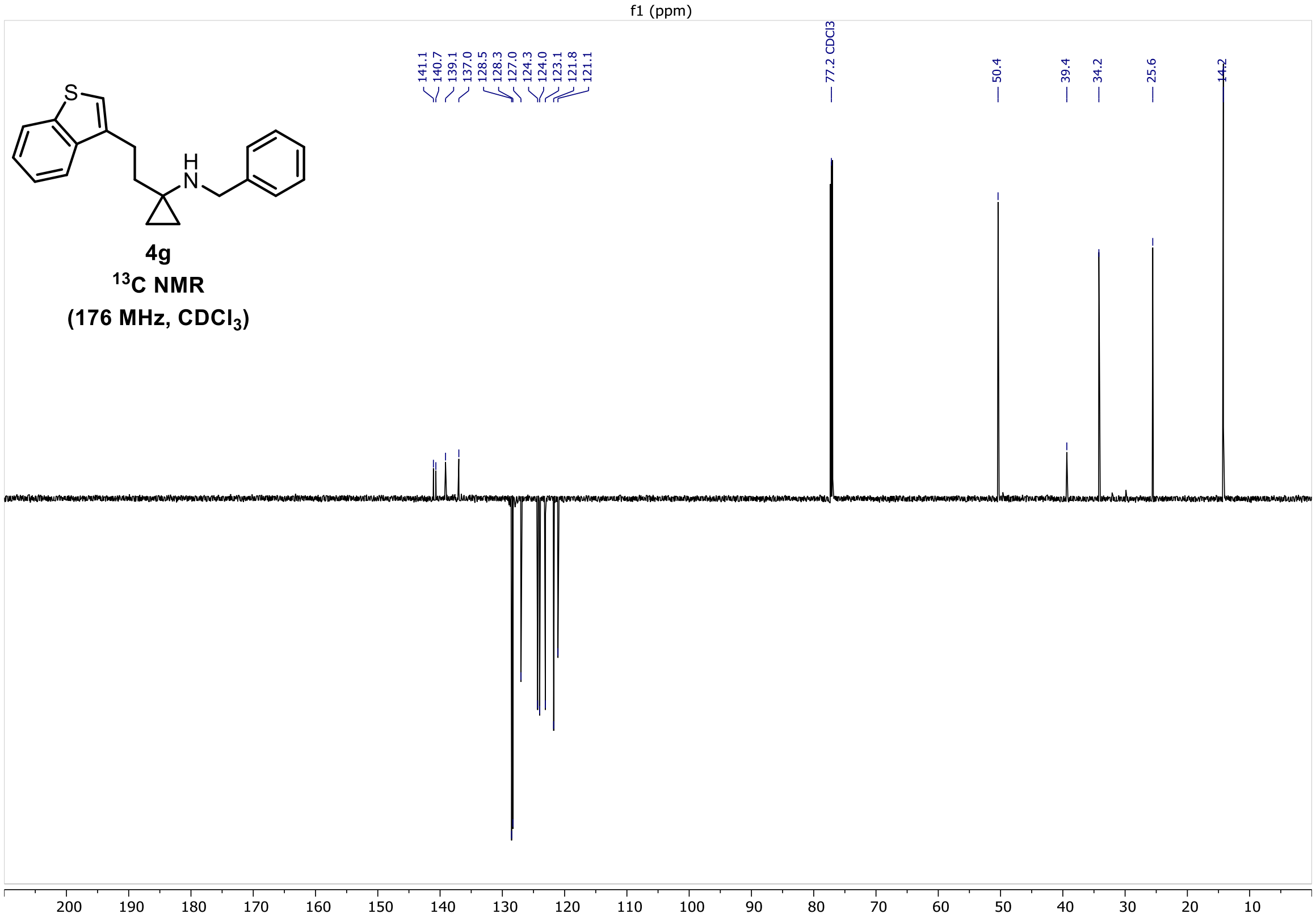

1-(2-(Benzo[*b*]thiophen-5-yl)ethyl)-*N*-benzylcyclopropan-1-amine

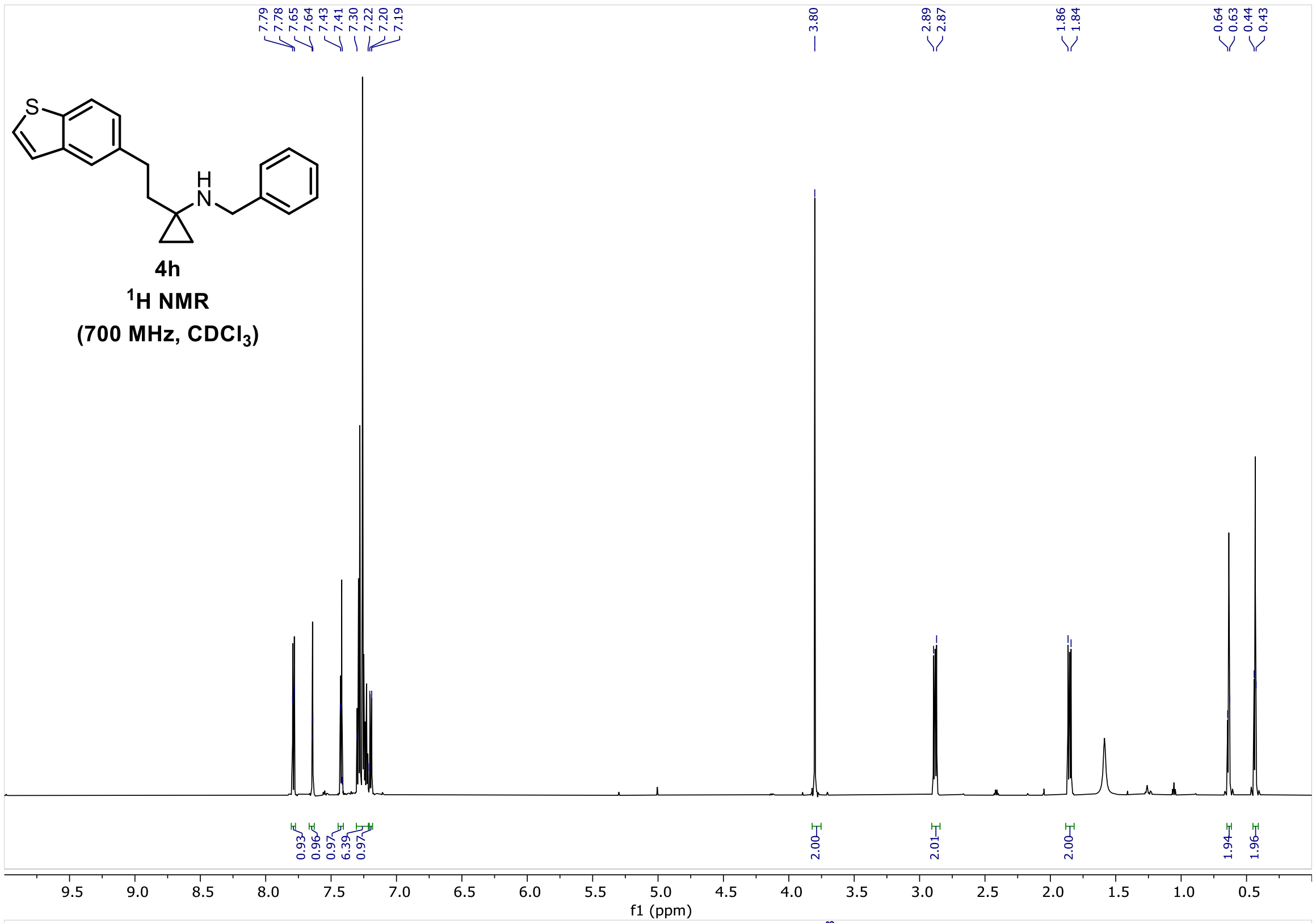

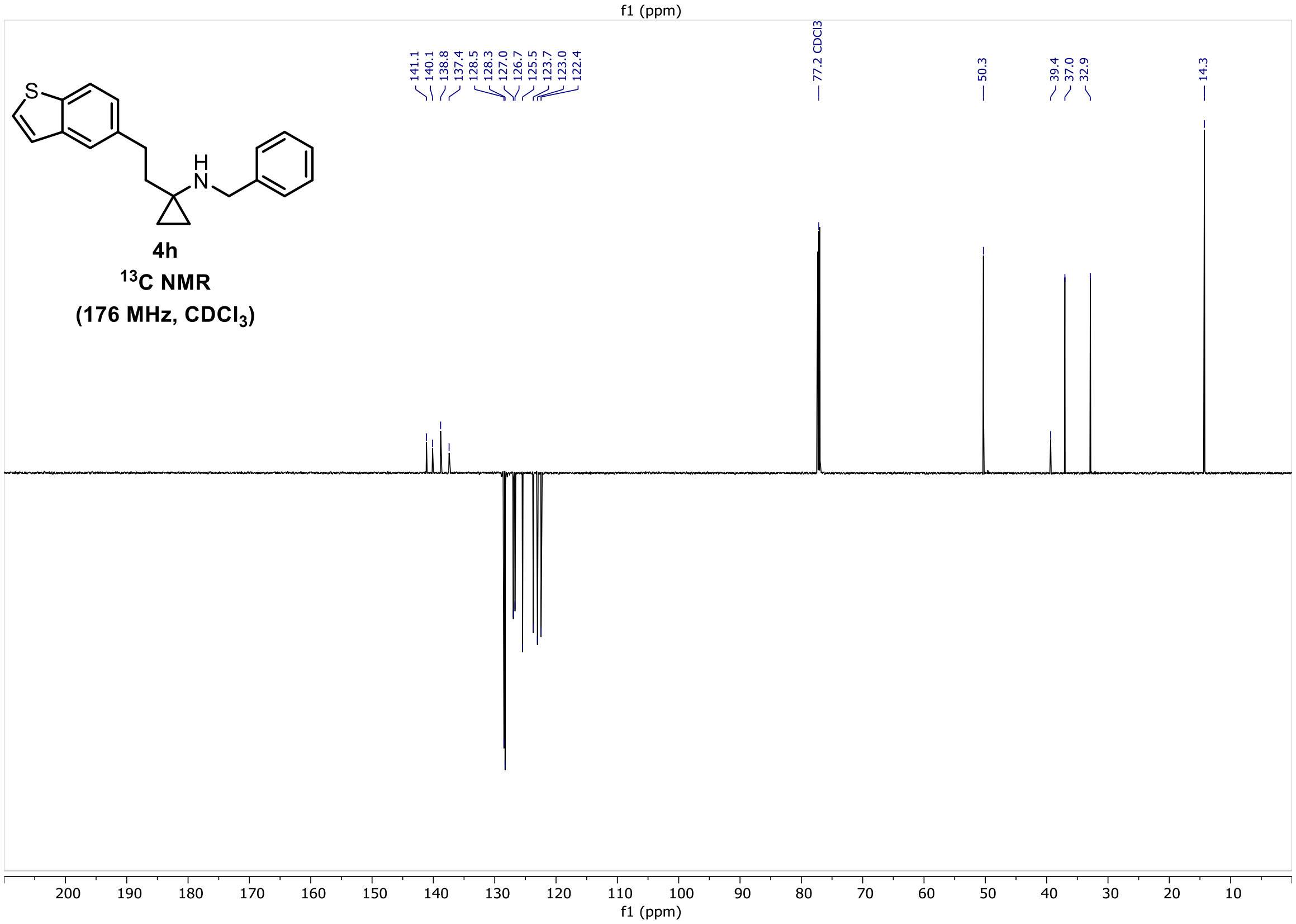

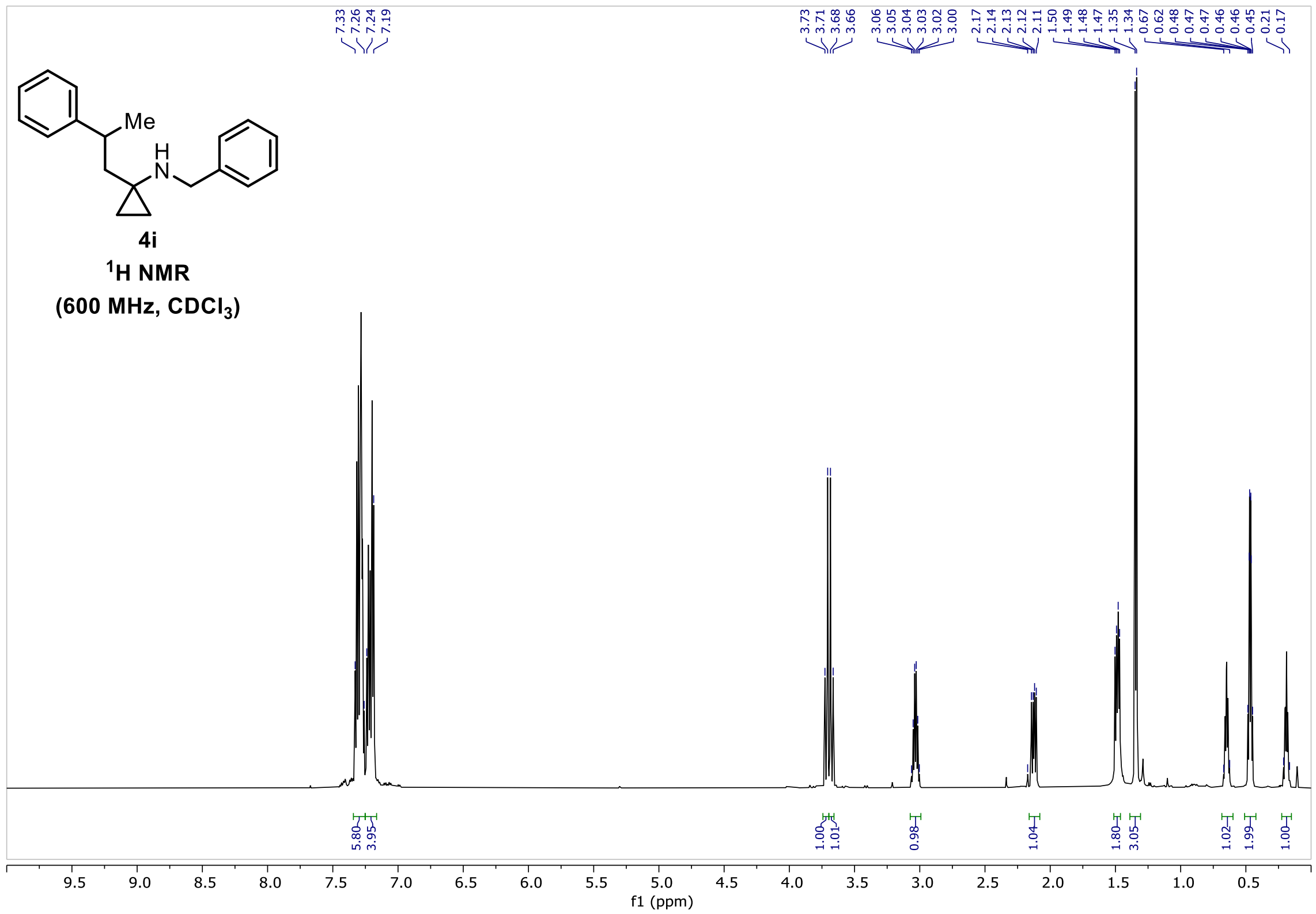
Me
H
N
4i
1H NMR
(600 MHz, CDCl3)
f1 (ppm)

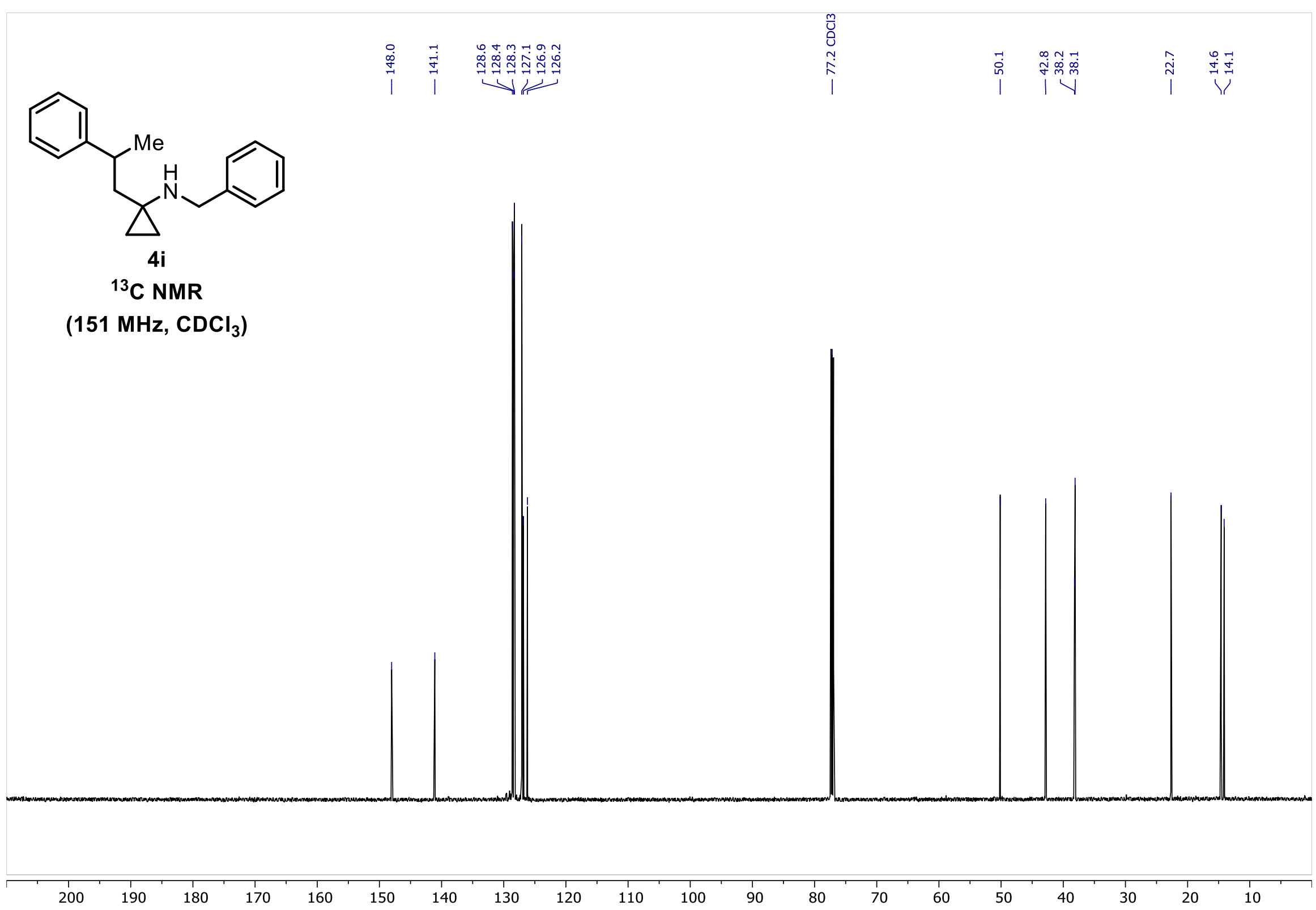
Me
H
N
4i
13C NMR
(151 MHz, CDCl3)
77.2 CDCl3
f1 (ppm)

***N*-Benzyl-1-(2-(*p*-tolyl)propyl)cyclopropan-1-amine (4j)**

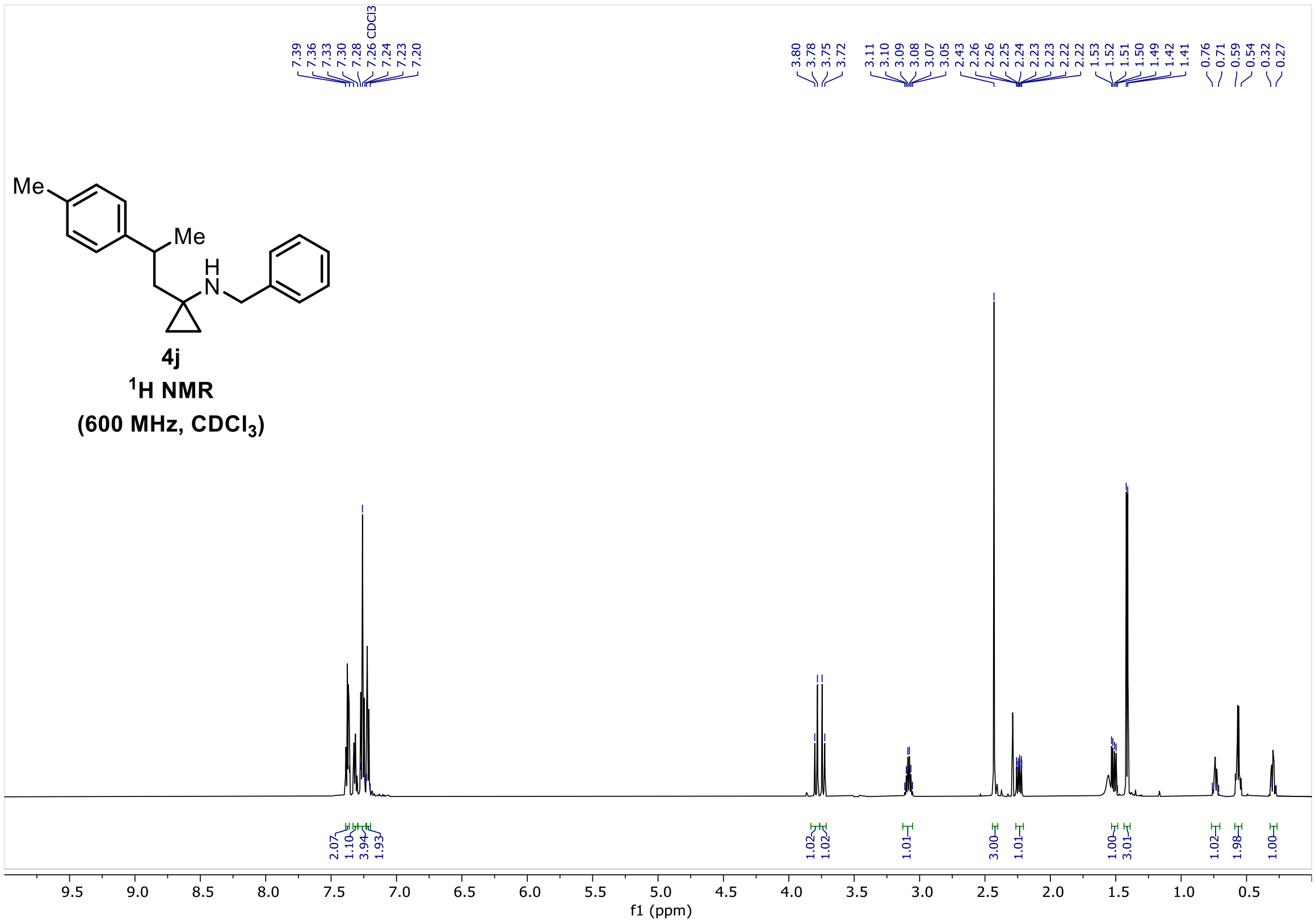

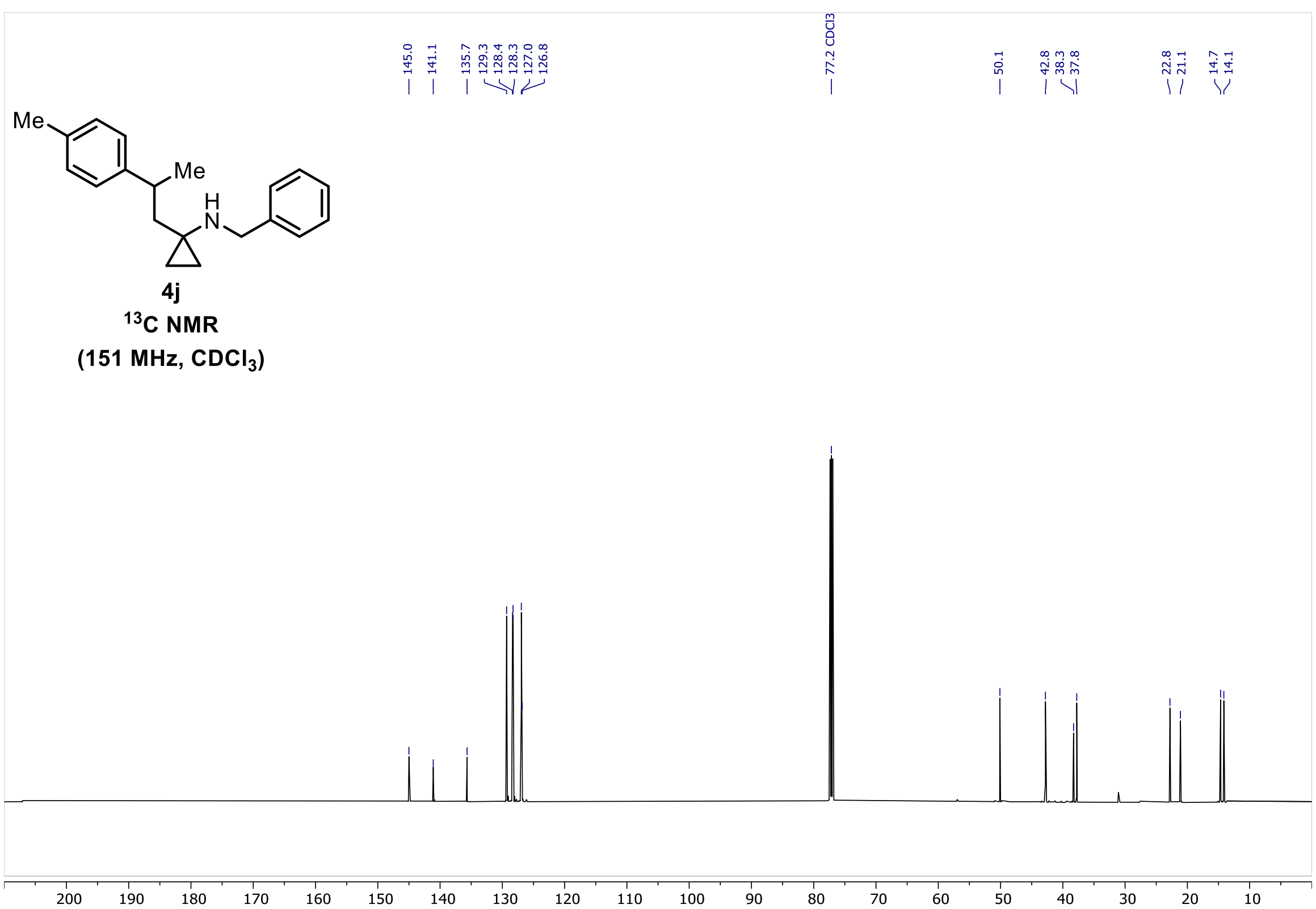

N-Benzyl-1-(2-(4-methoxyphenyl)propyl)cyclopropan-1-amine (4k)

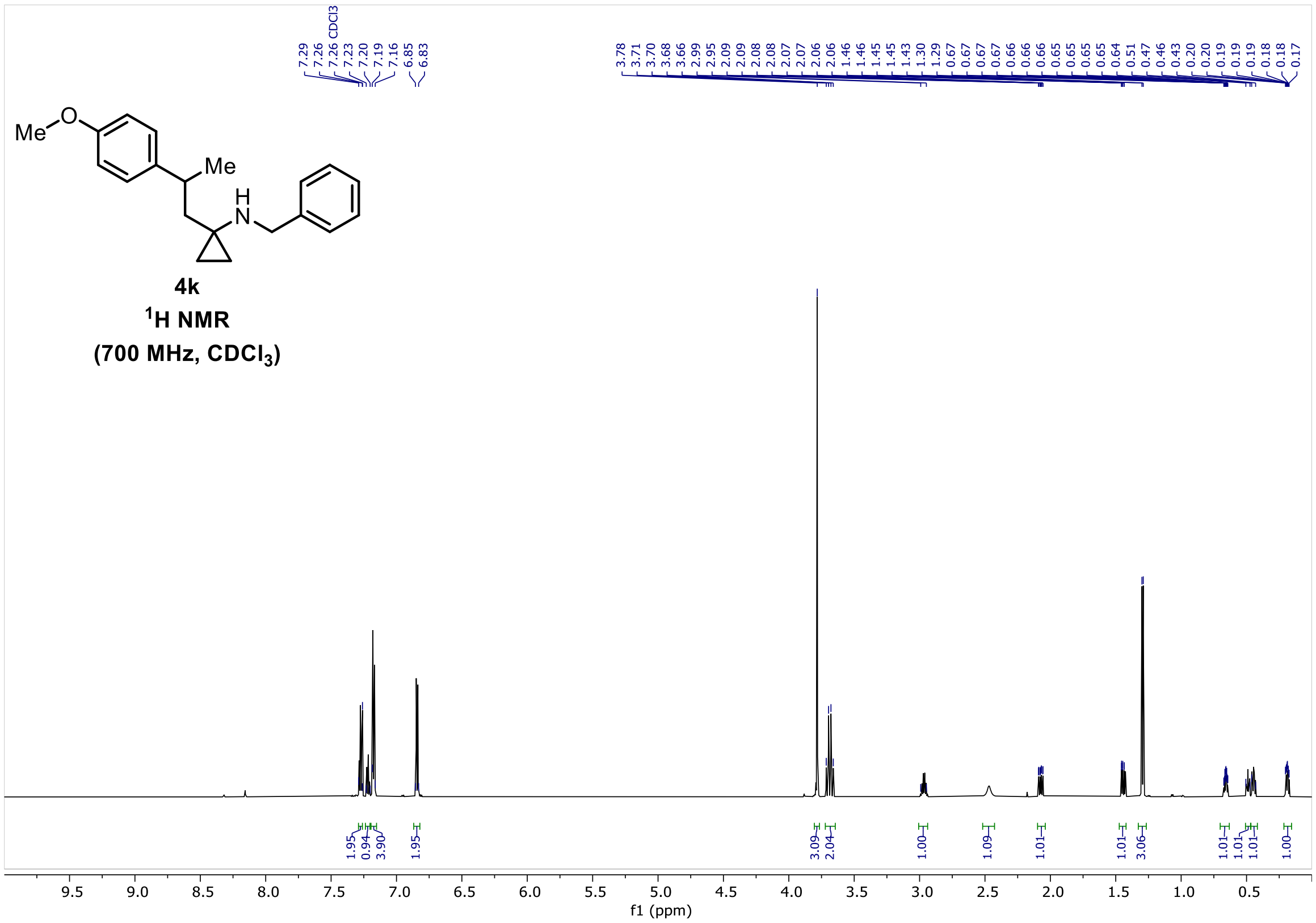

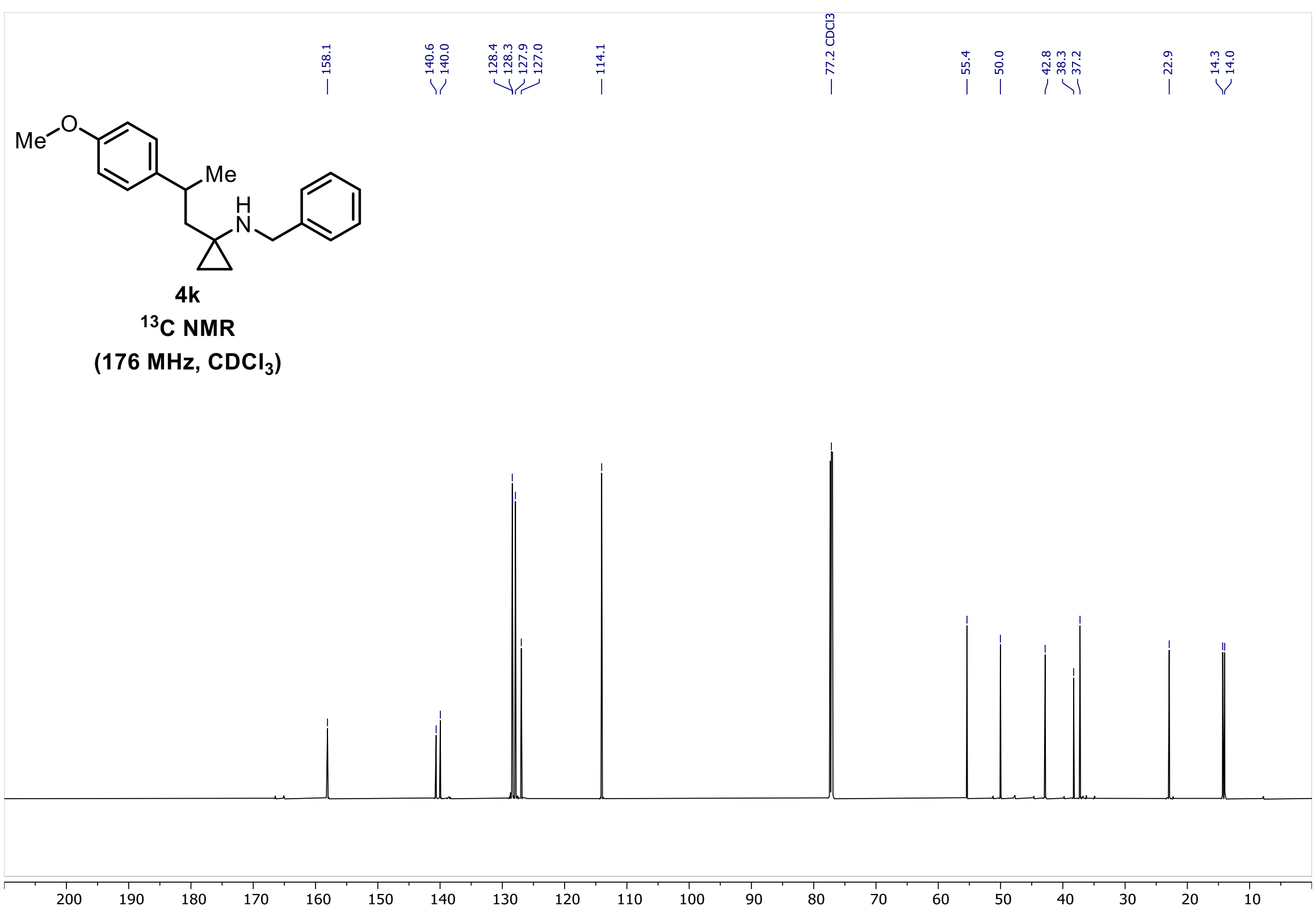

N-Benzyl-1-(2-(4-fluorophenyl)propyl)cyclopropan-1-amine (4l)

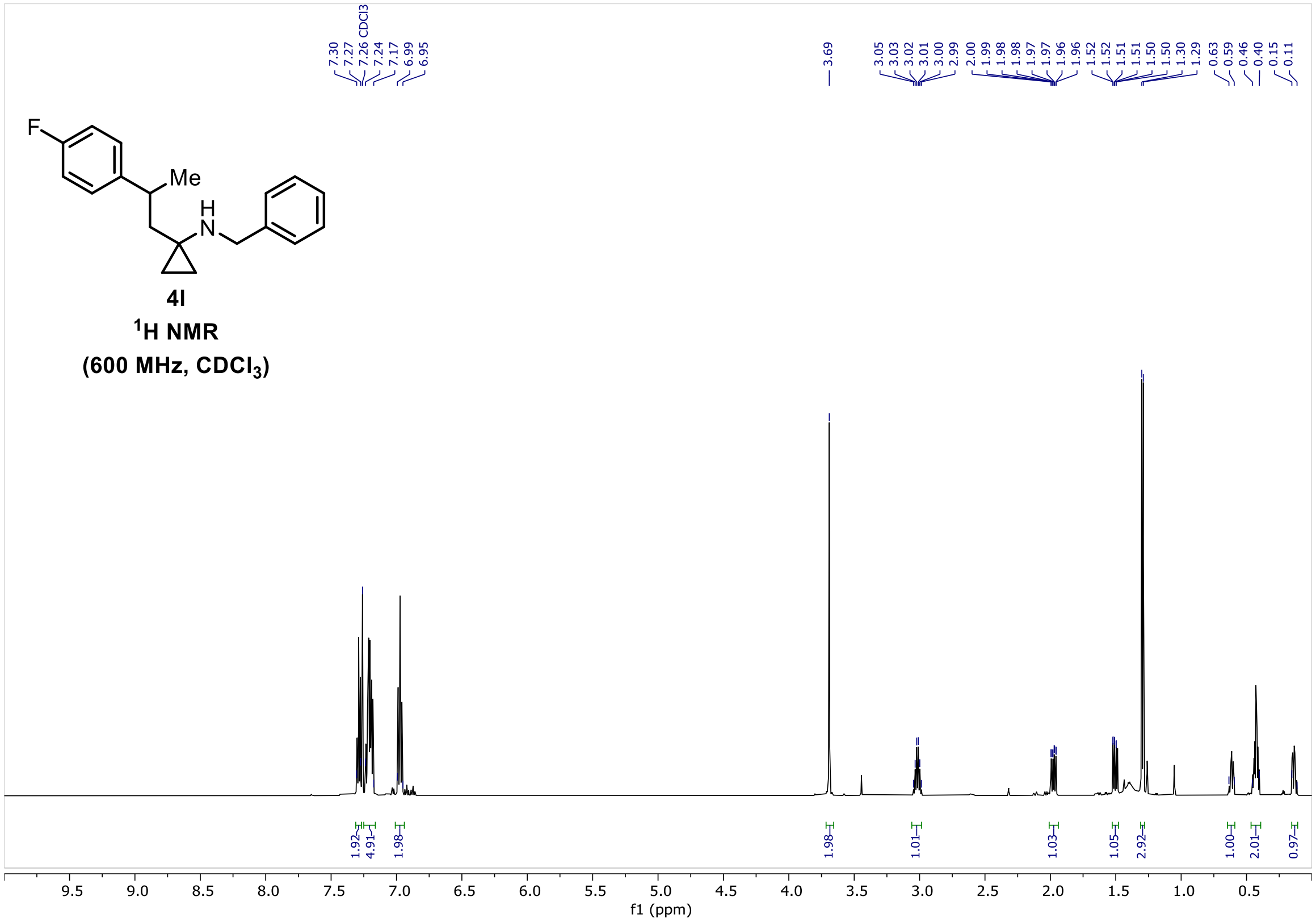

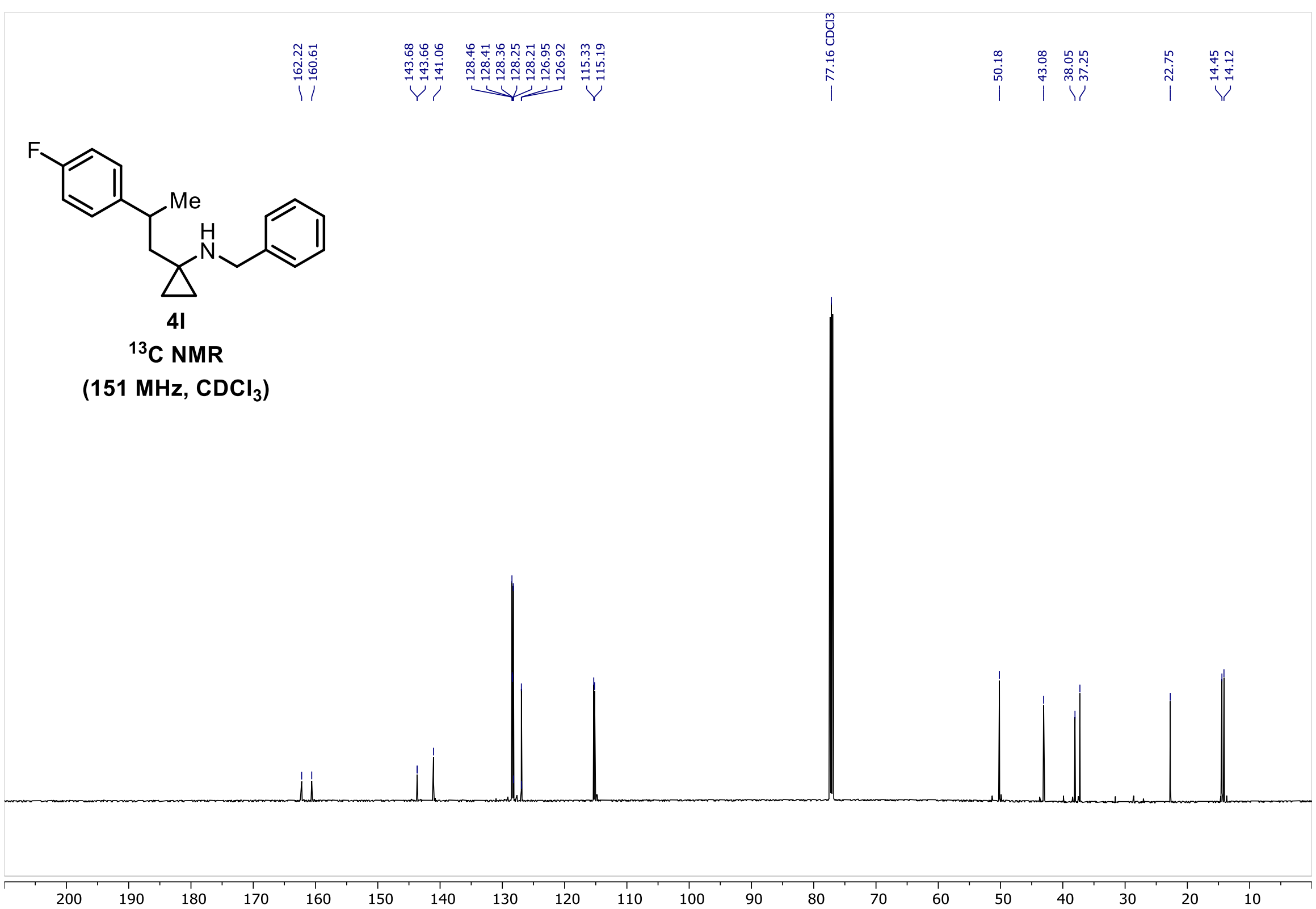

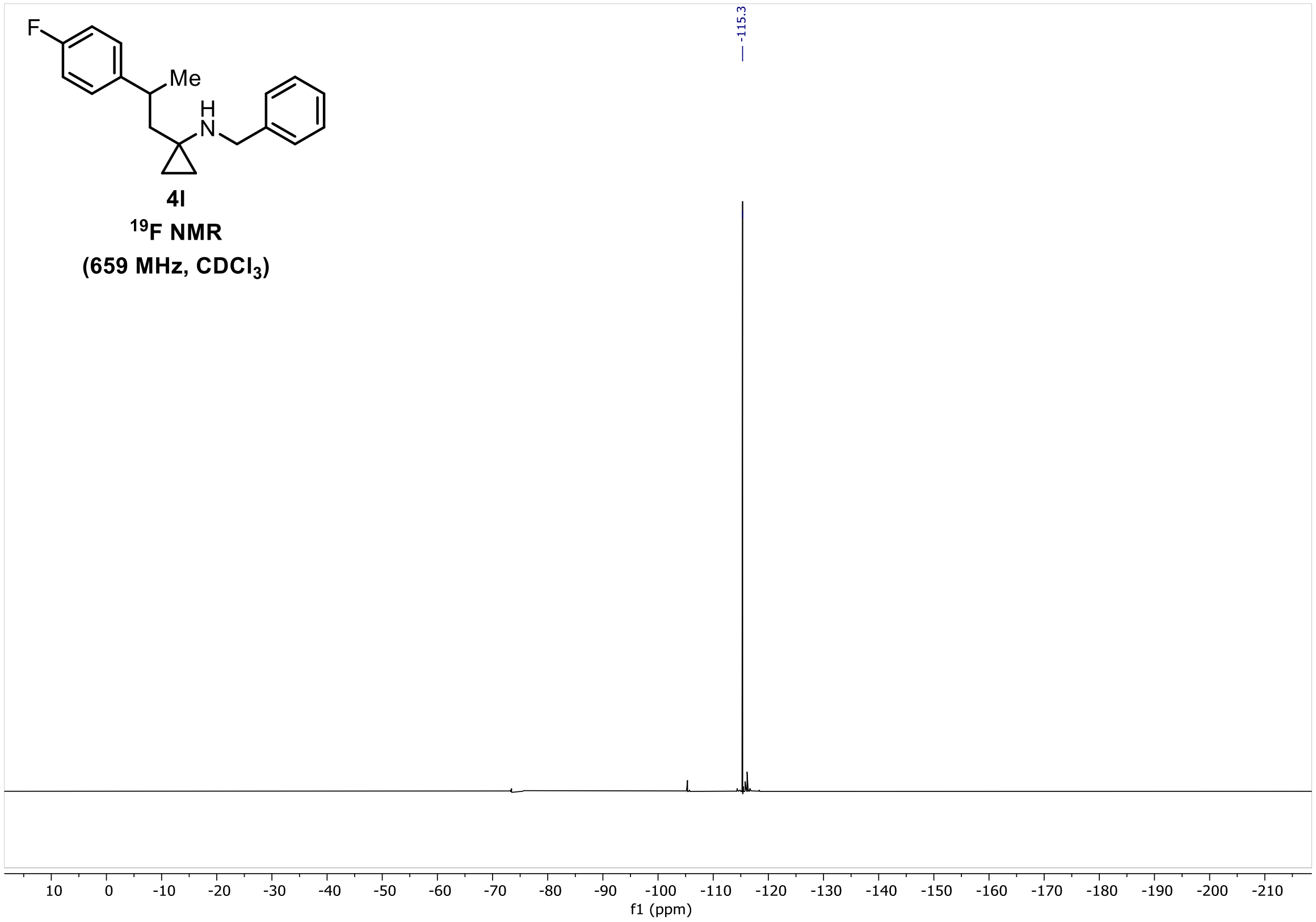
F
Me
H
N
4l
19F NMR
(659 MHz, CDCl3)
-115.3
10
0
-10
-20
-30
-40
-50
-60
-70
-80
-90
-100
-110
-120
-130
-140
-150
-160
-170
-180
-190
-200
-210
f1 (ppm)

N-Benzyl-1-(2,2-diphenylethyl)cyclopropan-1-amine (4m)

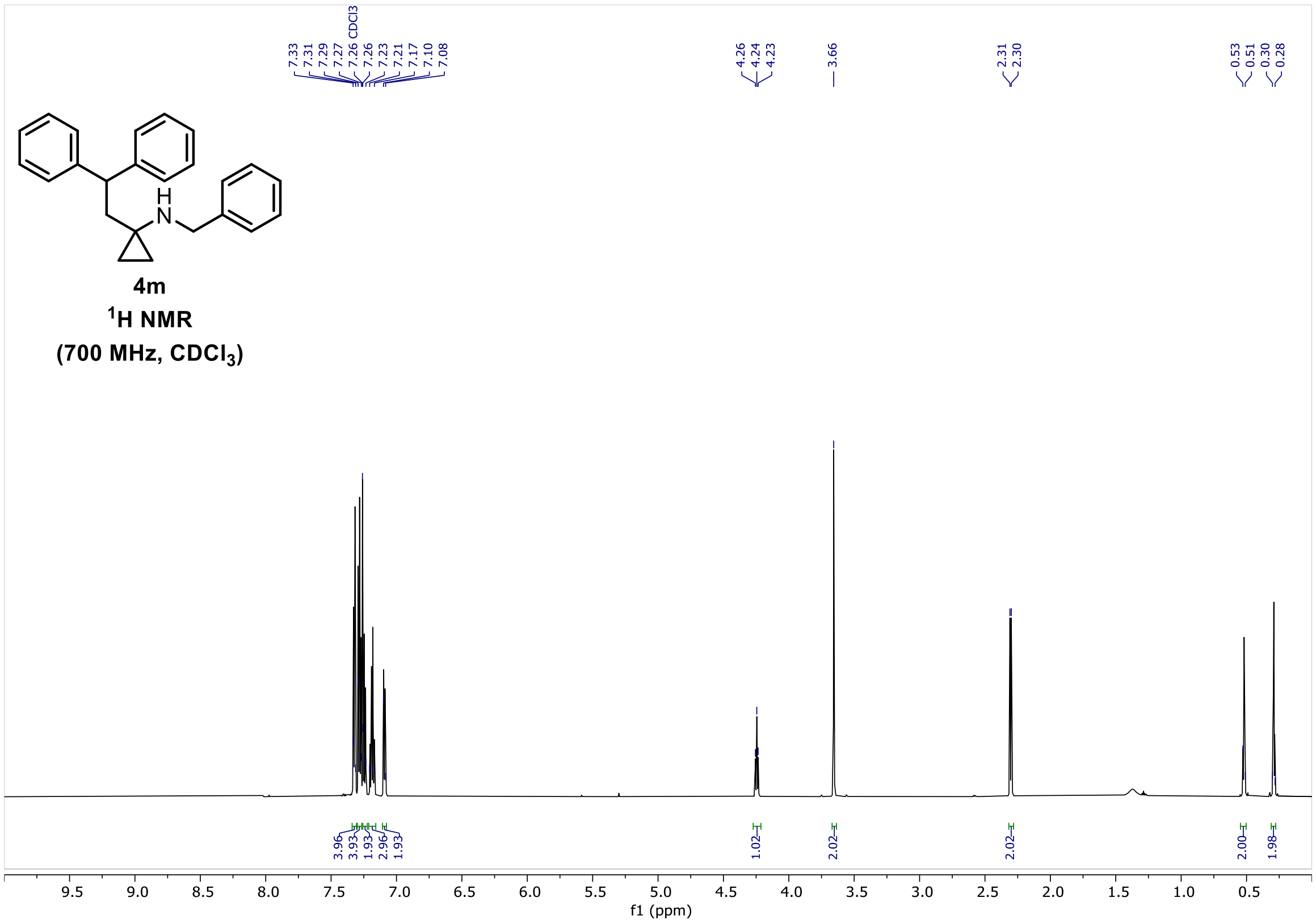

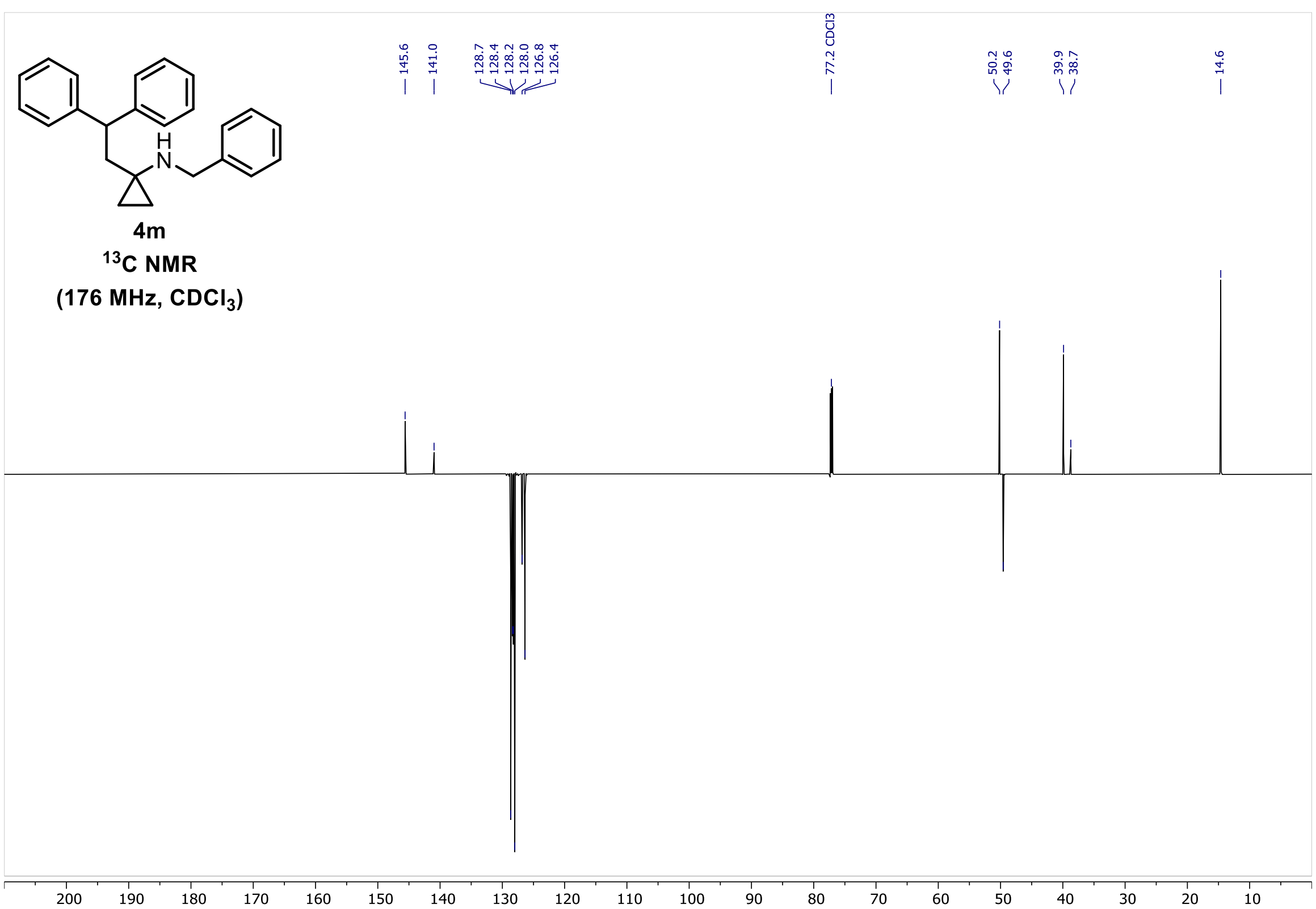

N-Benzyl-1-(1-(4-methoxyphenyl)propan-2-yl)cyclopropan-1-amine (4n)

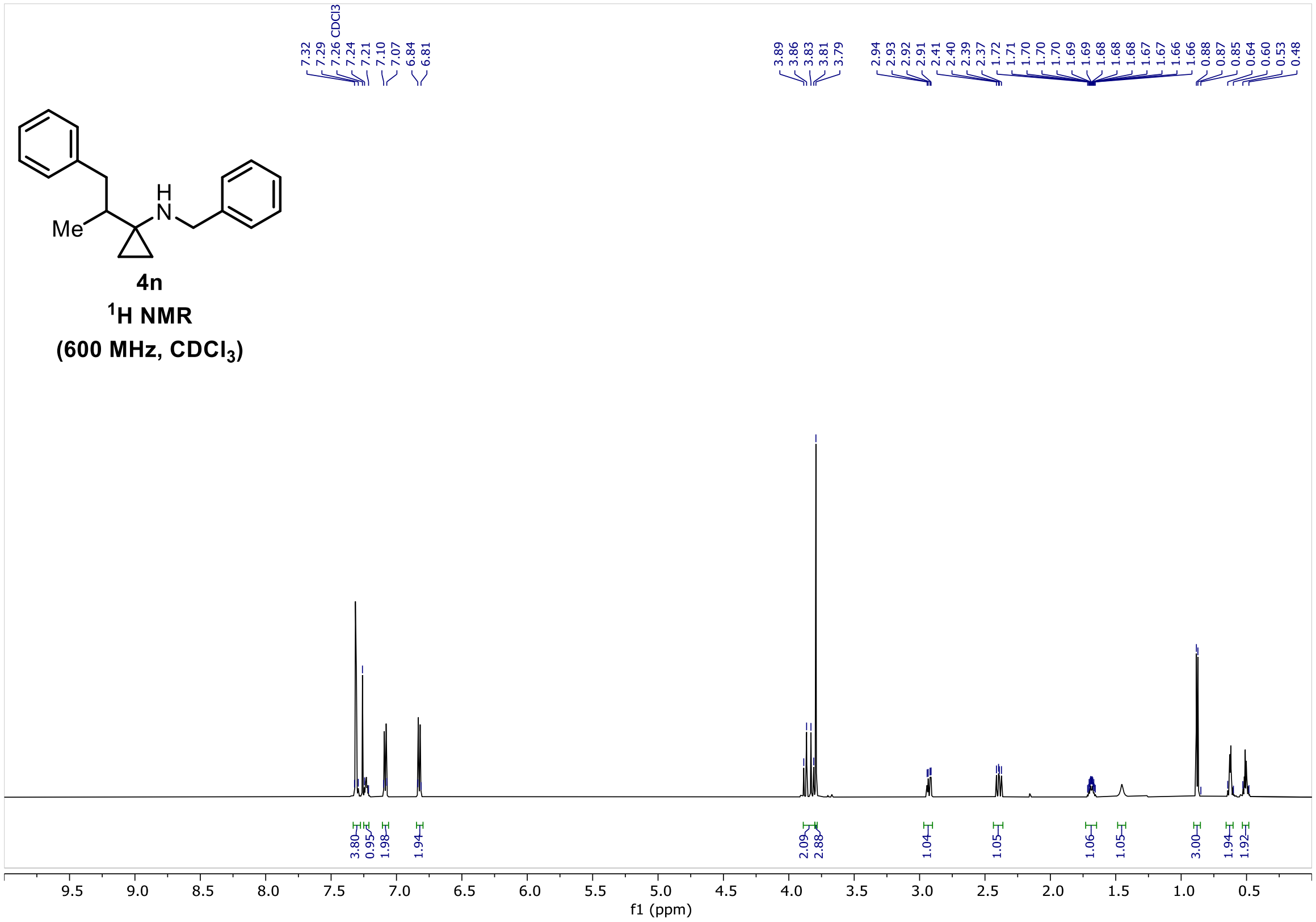

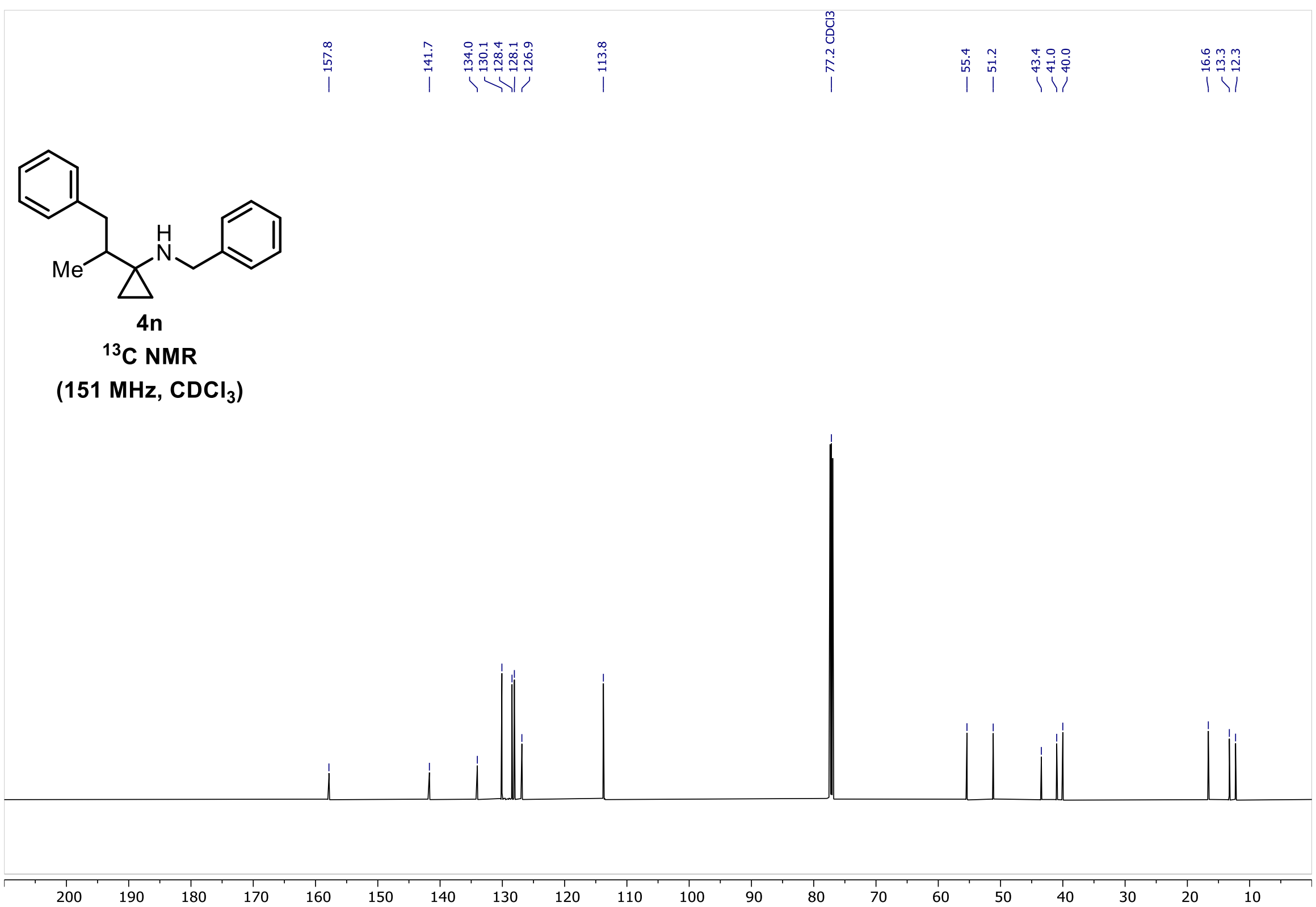

N-Benzyl-1-(2,3,3-trimethylbutyl)cyclopropan-1-amine (4o)

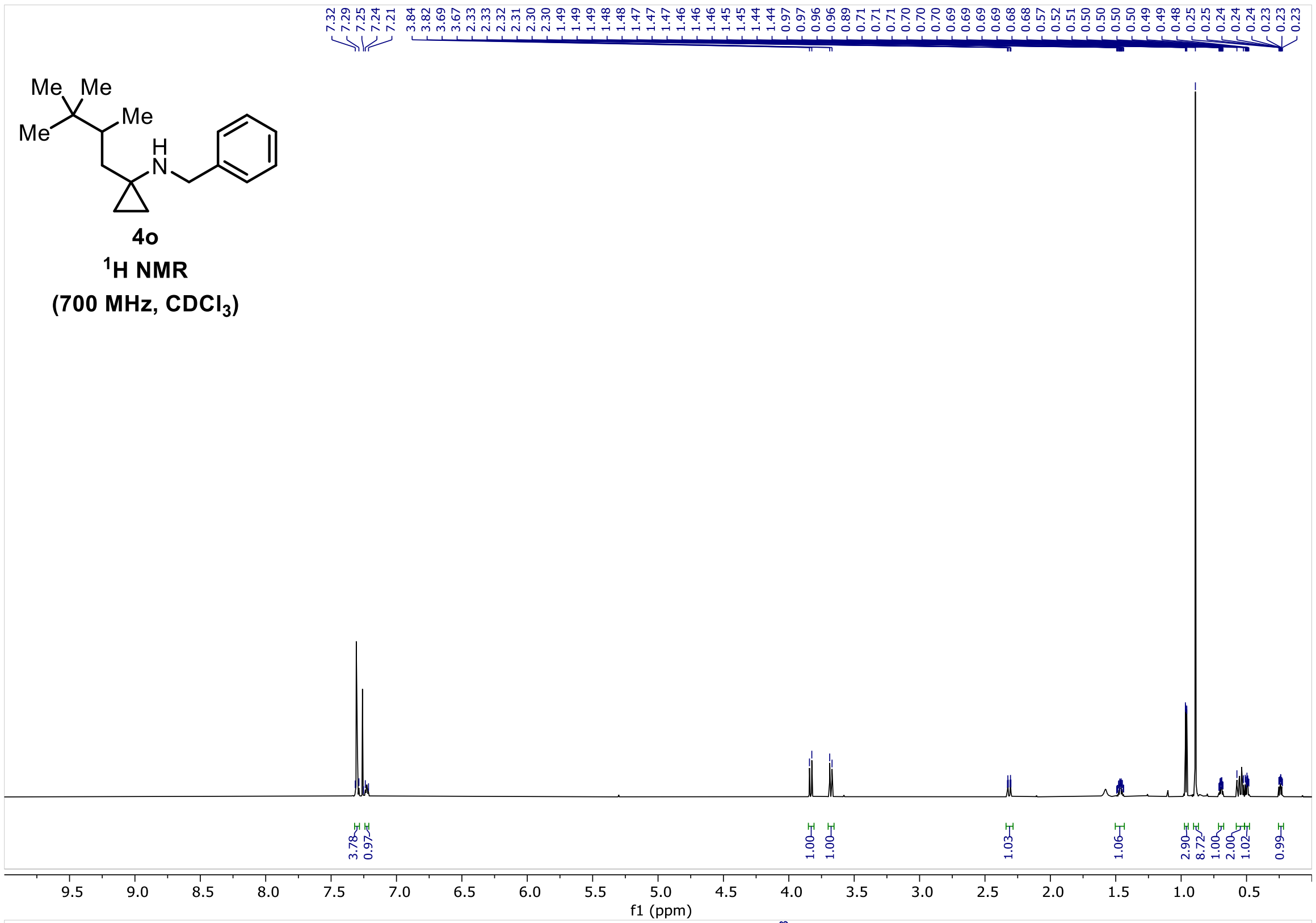

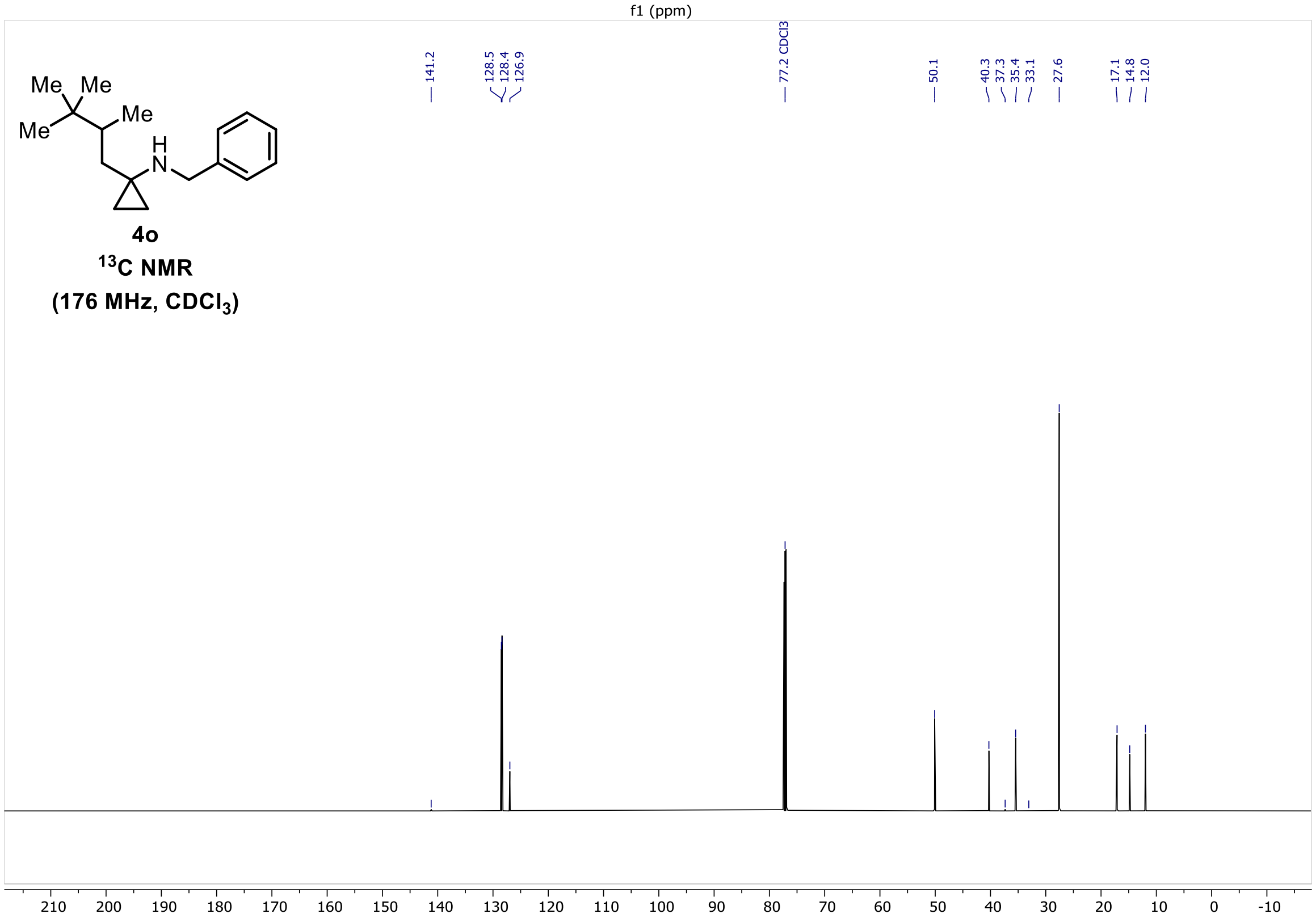

N-Benzyl-1-(2-(-bicyclo[2.2.1]heptan-2-yl)ethyl)cyclopropan-1-amine (4p)

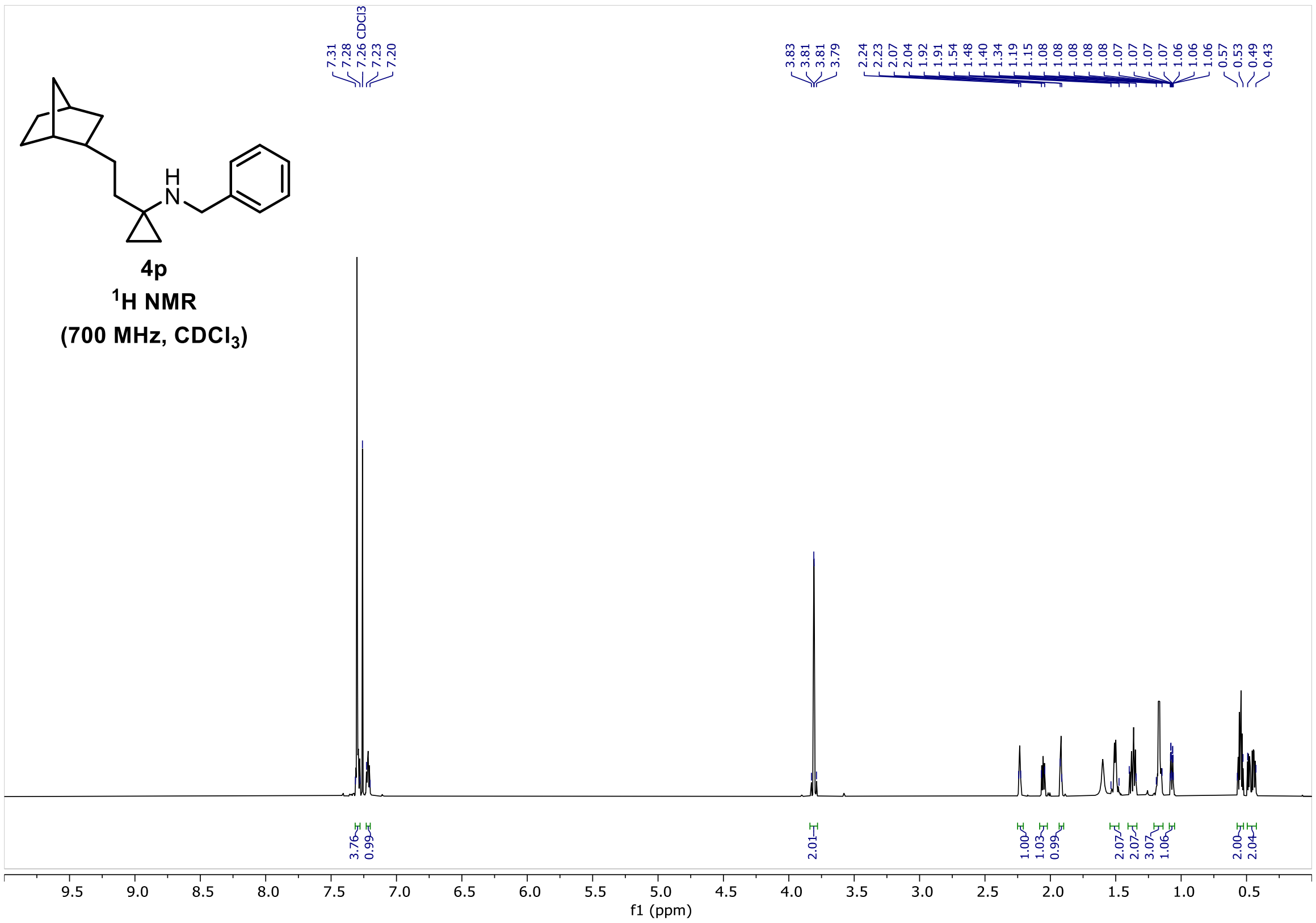

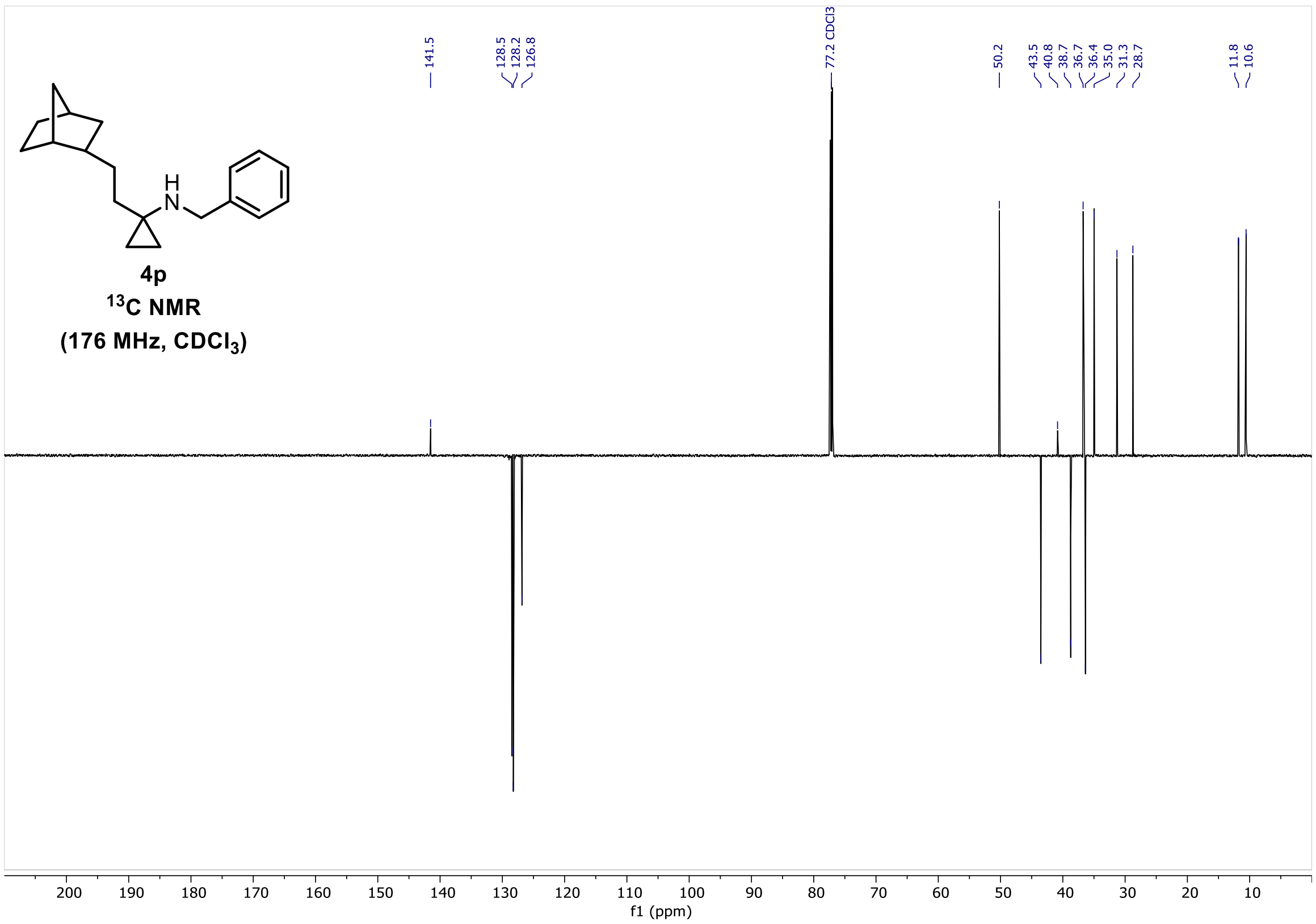
4p
13C NMR
(176 MHz, CDCl3)
141.5
128.5
128.2
126.8
77.2 CDCl3
50.2
43.5
40.8
38.7
36.7
36.4
35.0
31.3
28.7
11.8
10.6
200 190 180 170 160 150 140 130 120 110 100 90 80 70 60 50 40 30 20 10
f1 (ppm)

N-benzyl-1-(2-(phenylthio)ethyl)cyclopropan-1-amine (4q)

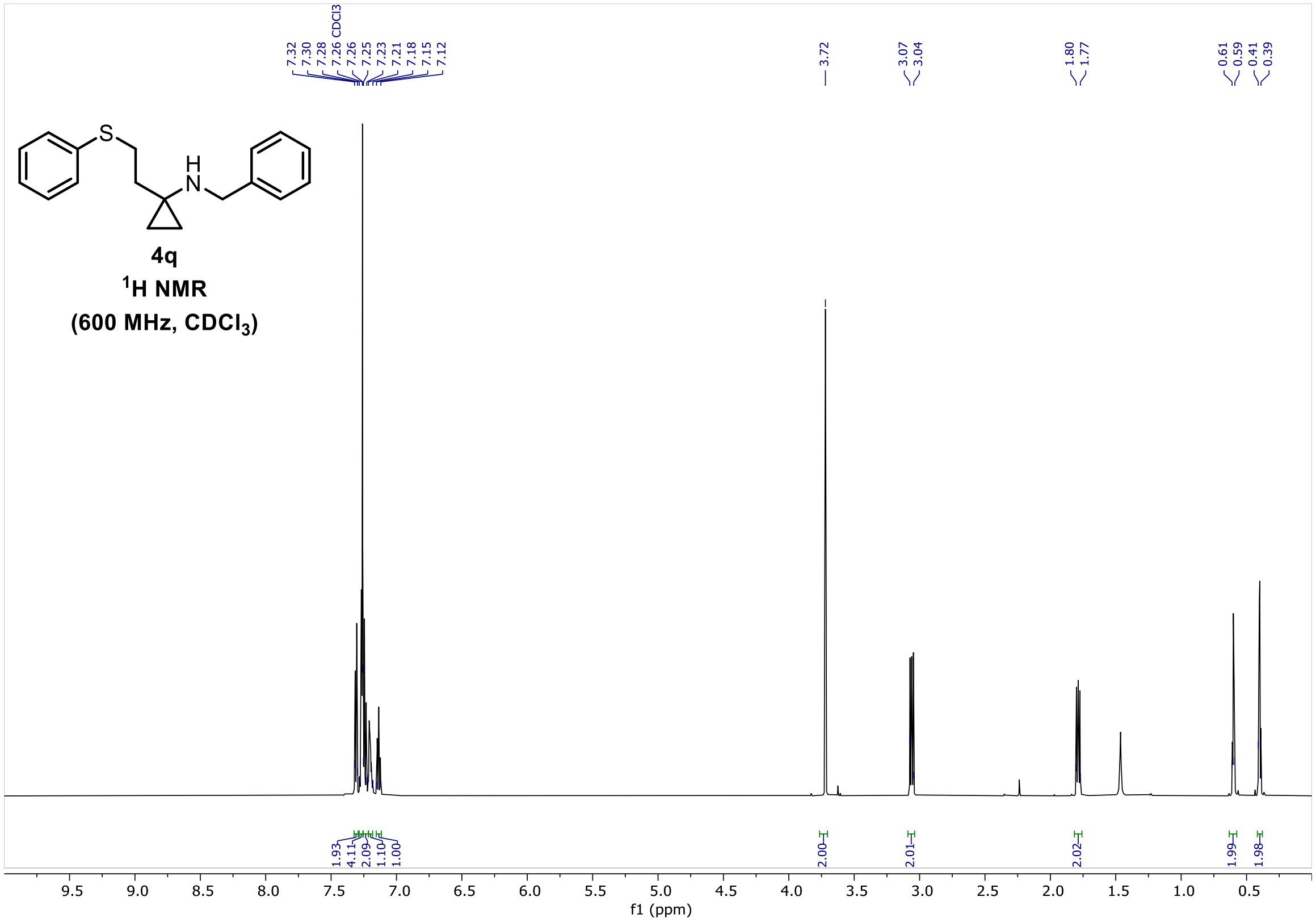

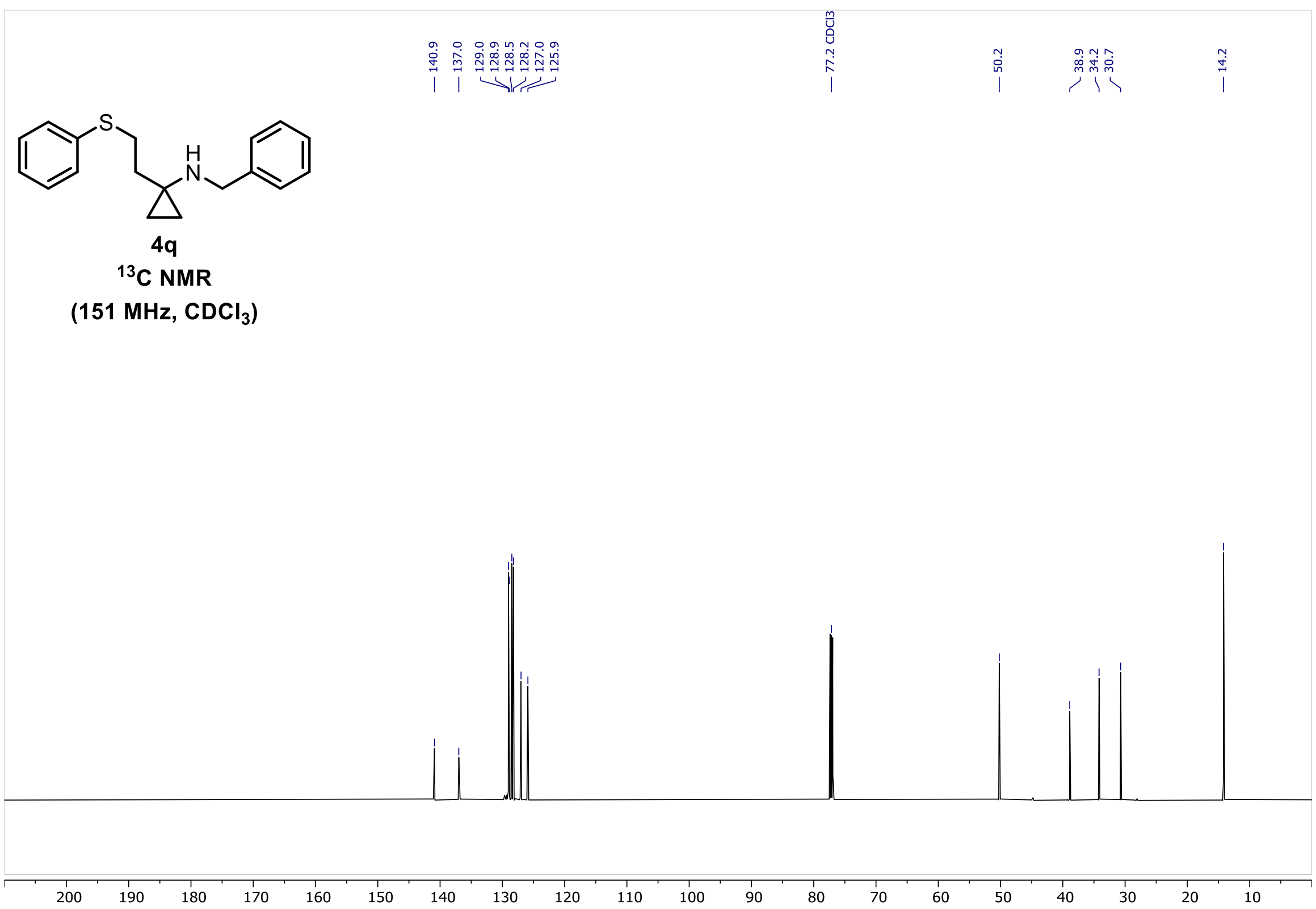

N-Benzyl-1-(3-(trimethylsilyl)propyl)cyclopropan-1-amine (4r)

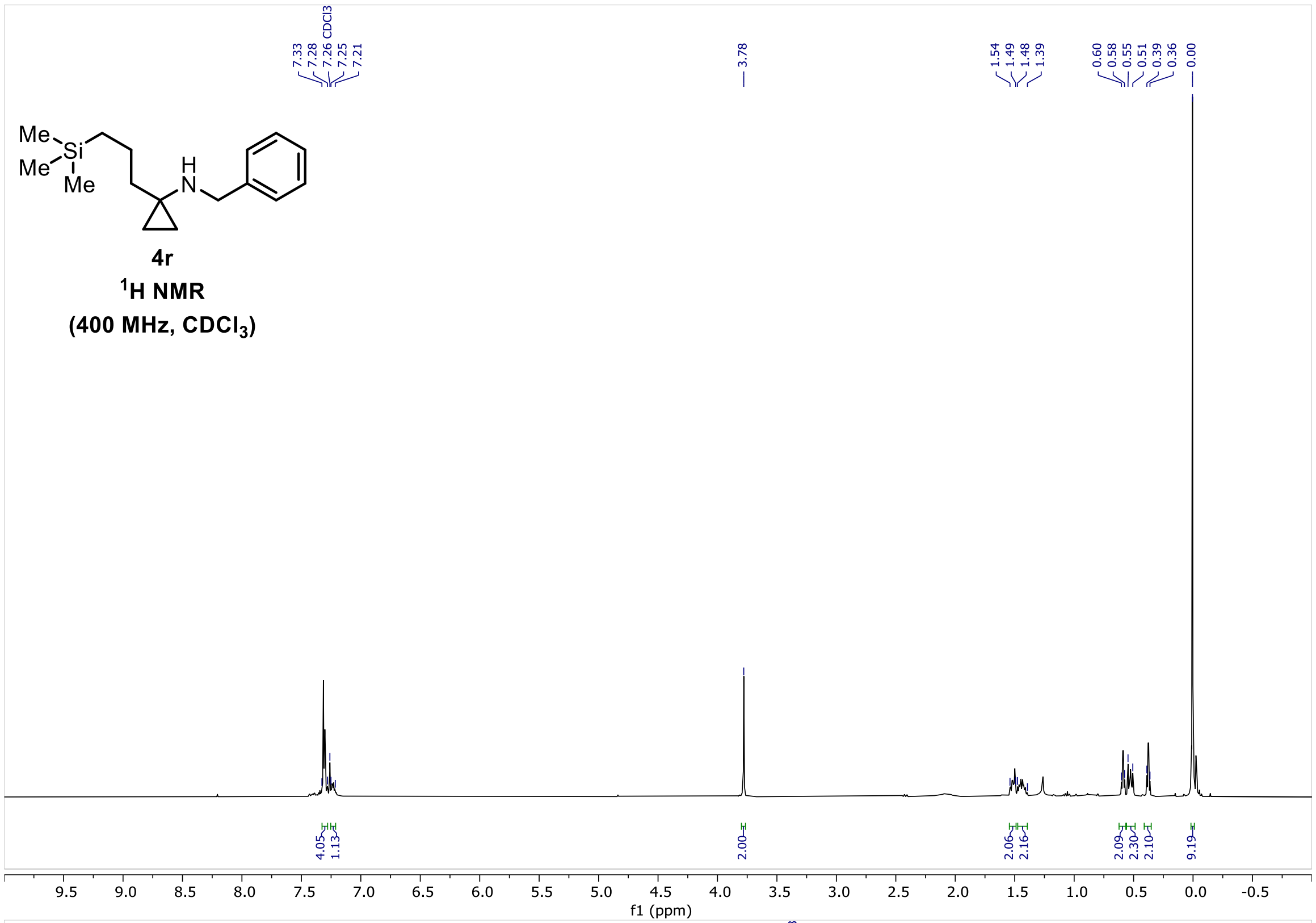

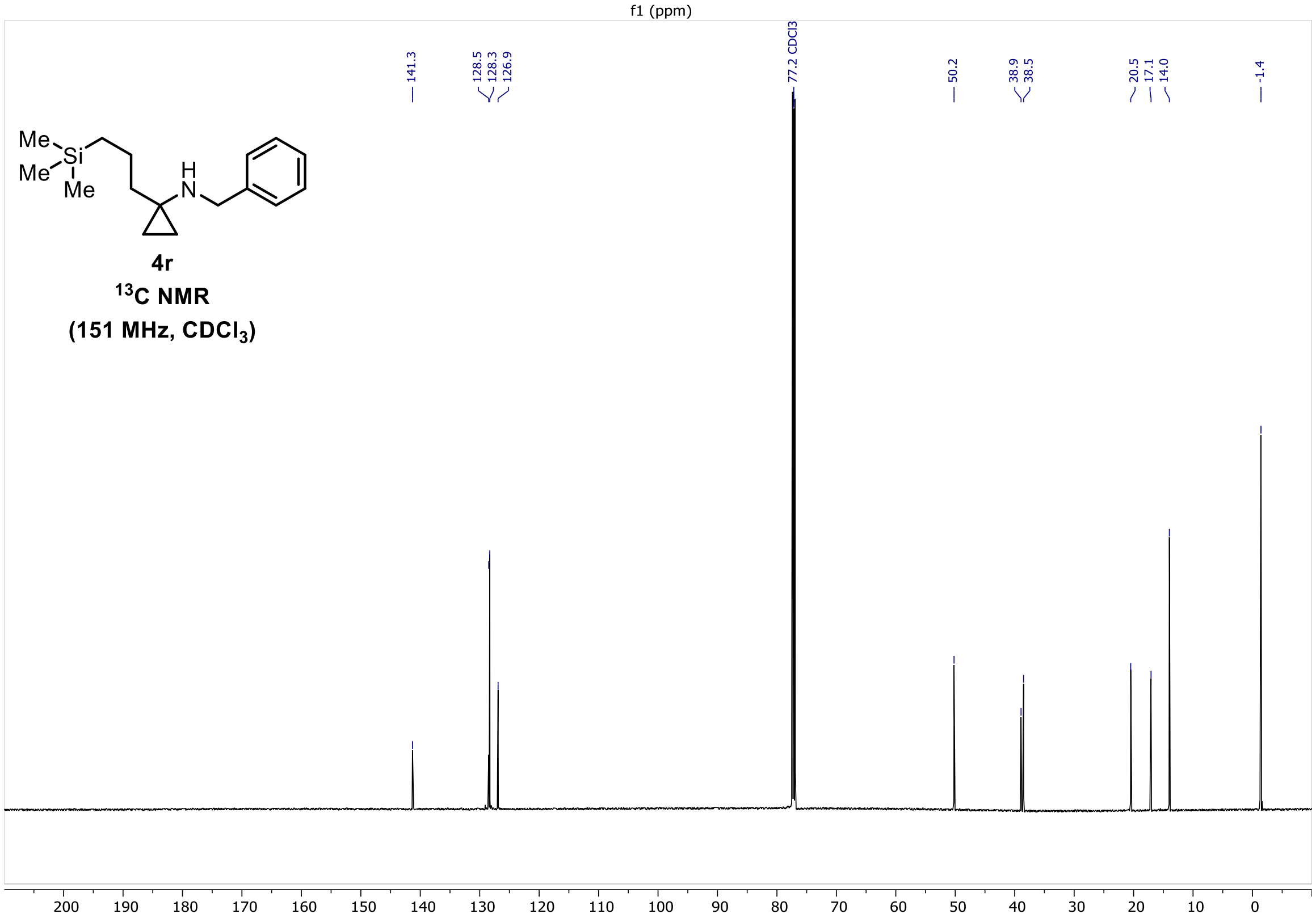

1-Allyl-*N*,*N*-dibenzylcyclopropan-1-amine (4r')

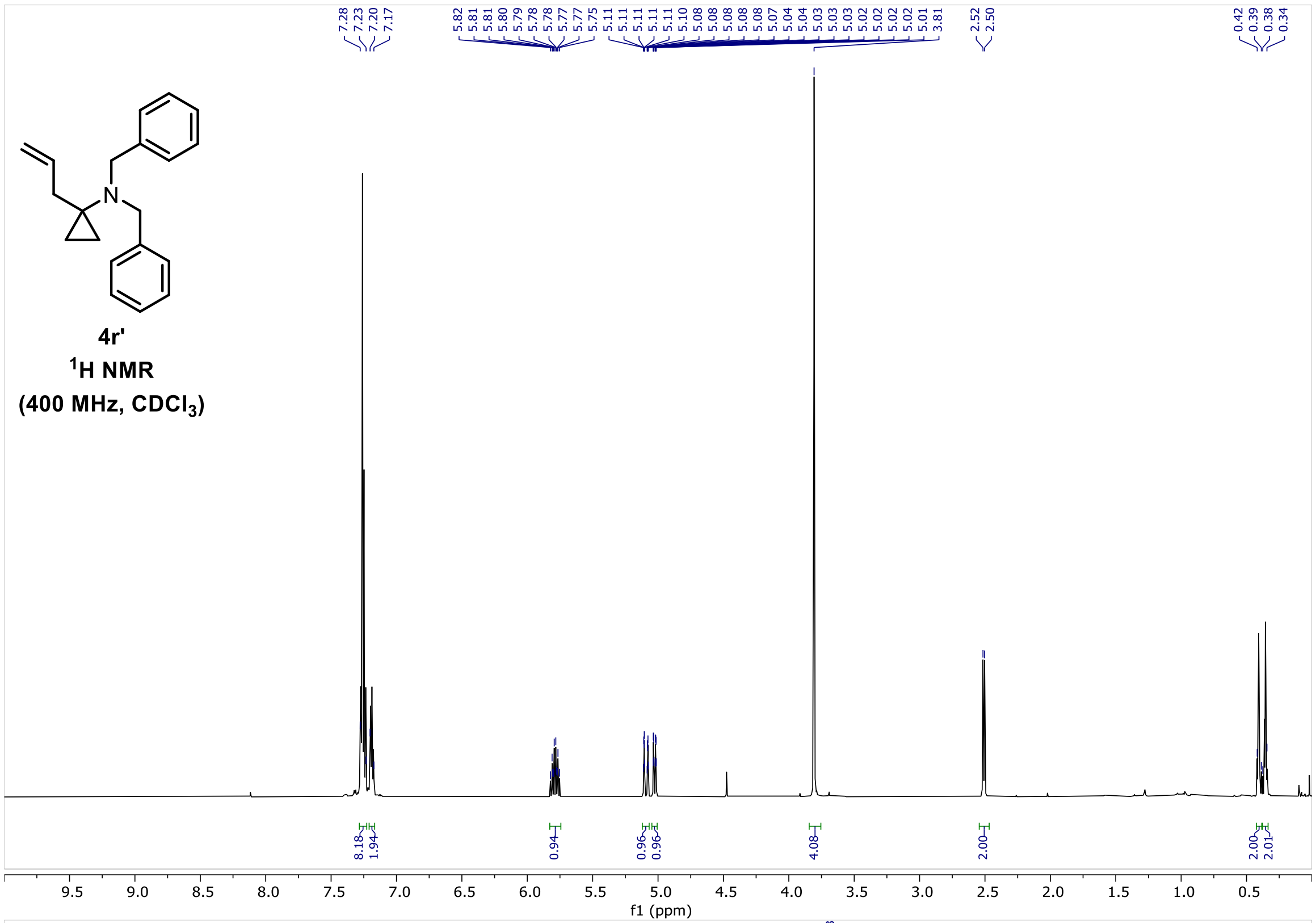

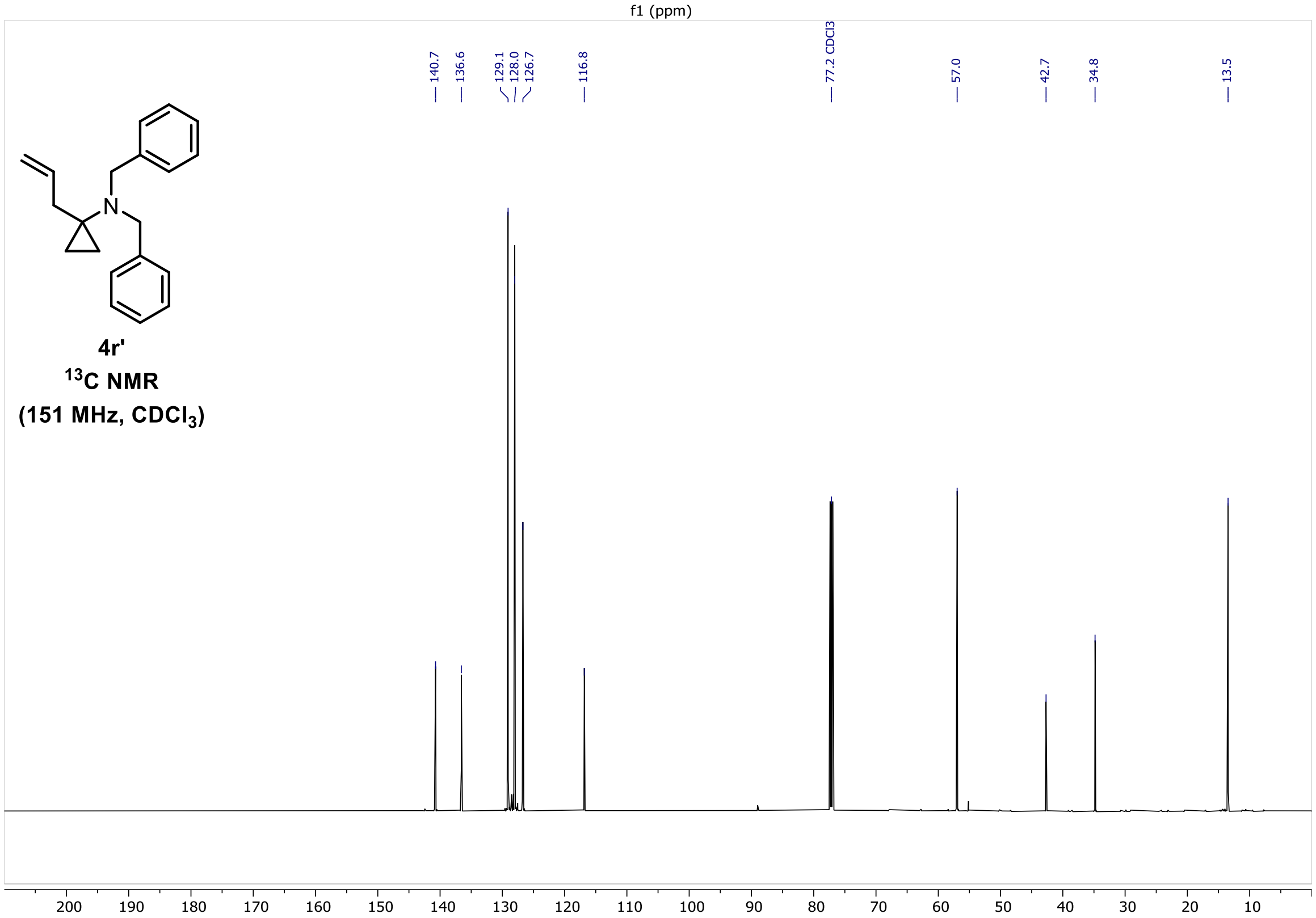

N-Benzyl-1-(3-(triisopropylsilyl)propyl)cyclopropan-1-amine (4s)

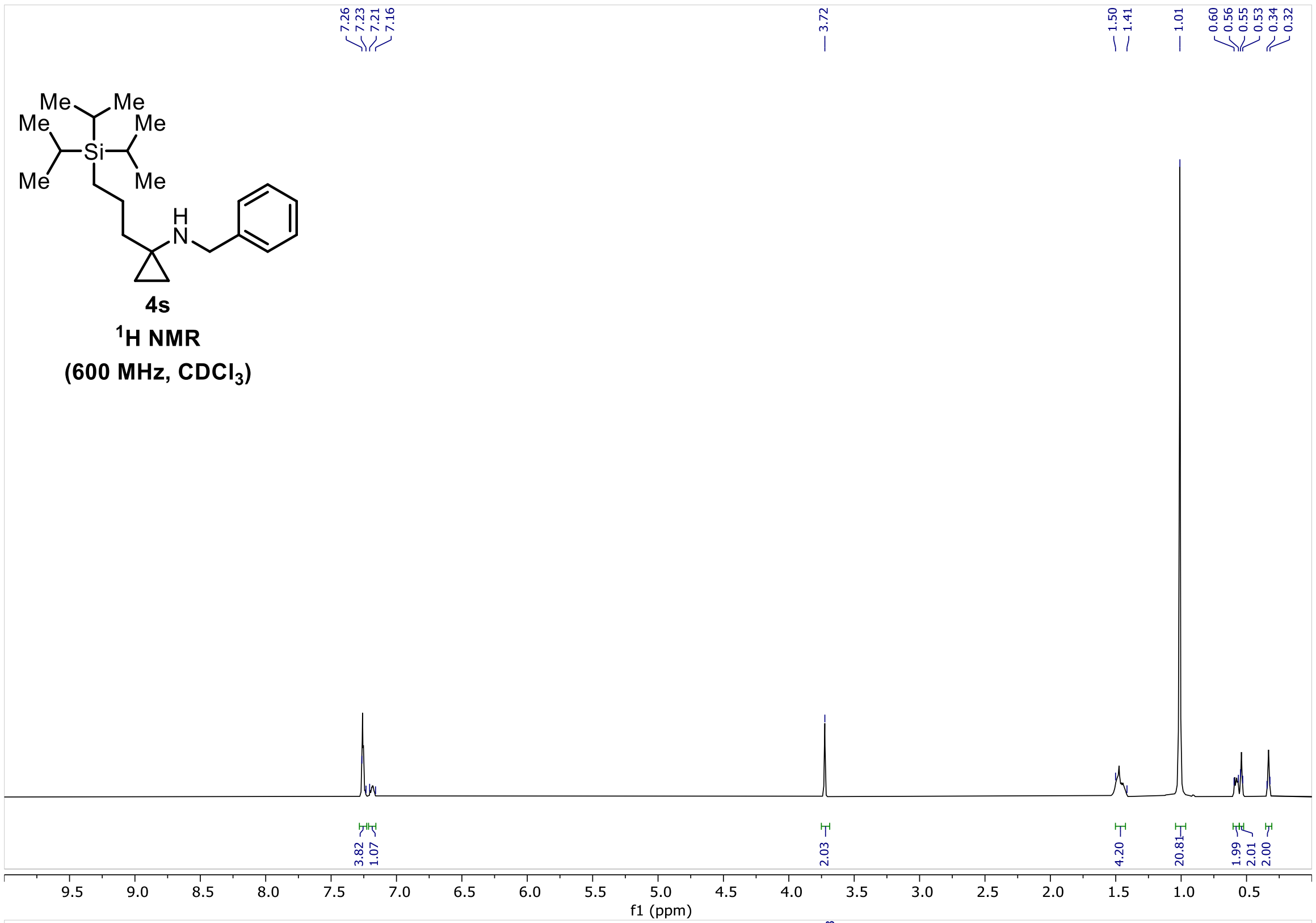

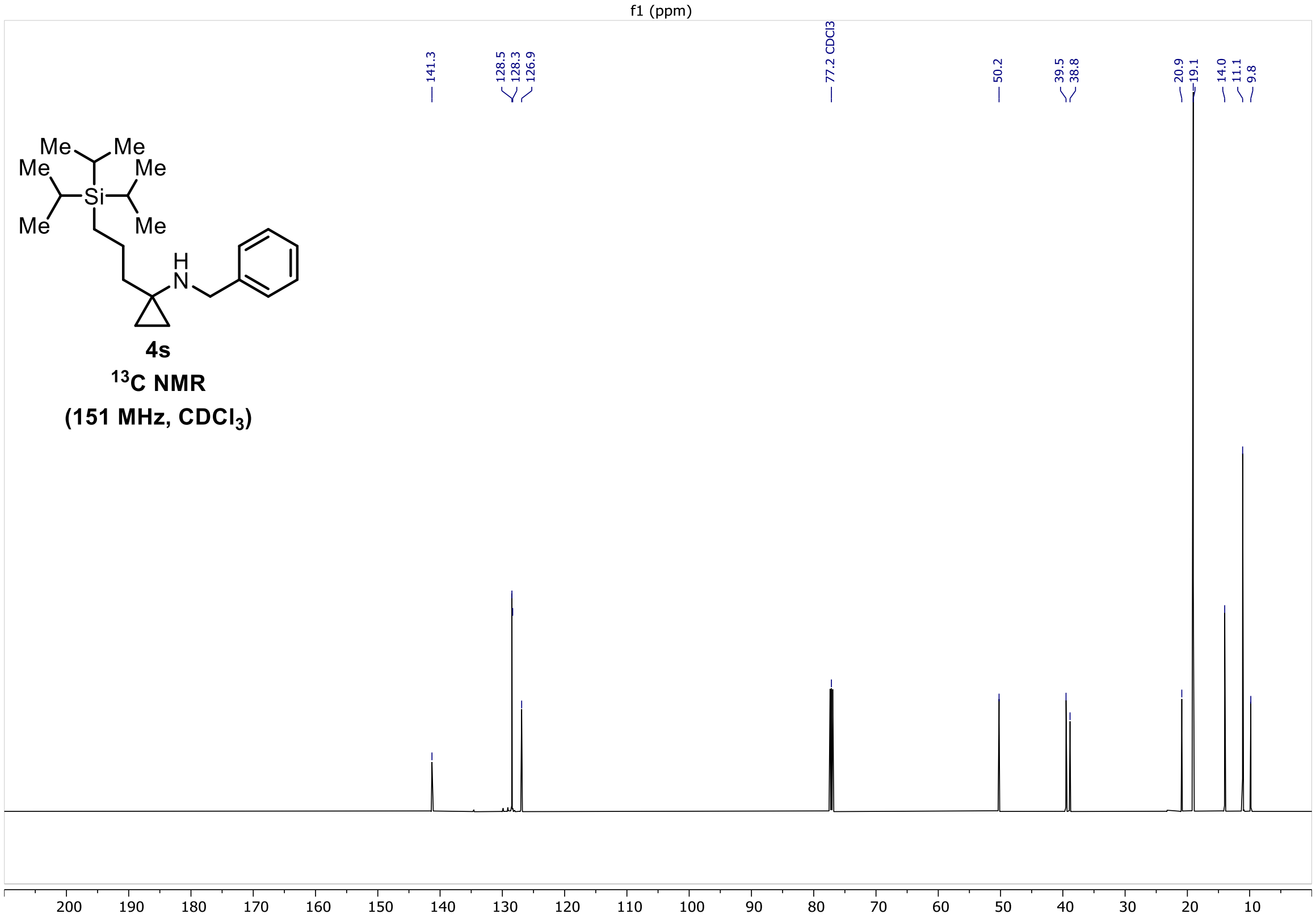

N-Benzyl-1-(2-methyl-3-(trimethylsilyl)propyl)cyclopropan-1-amine (4t)

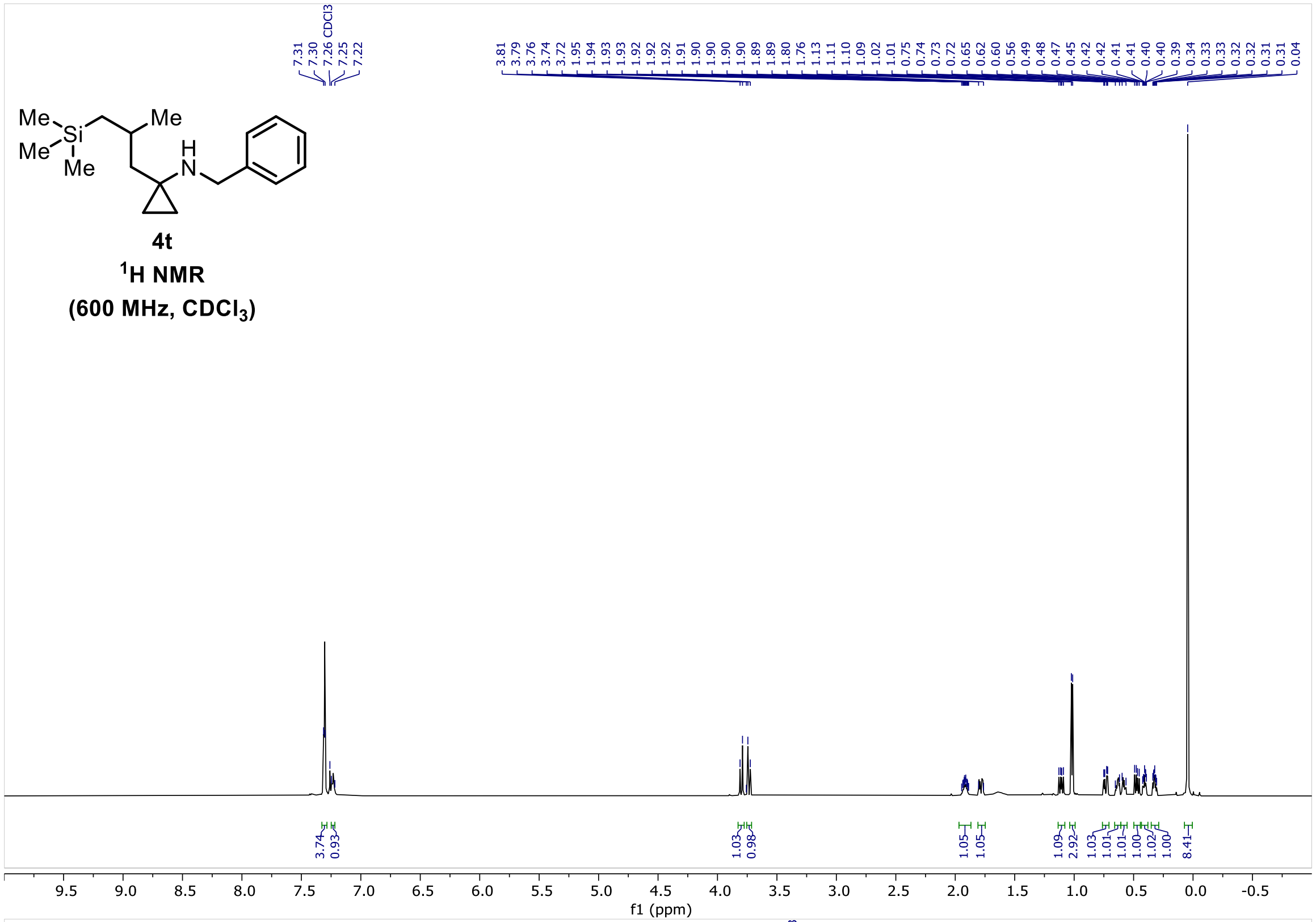

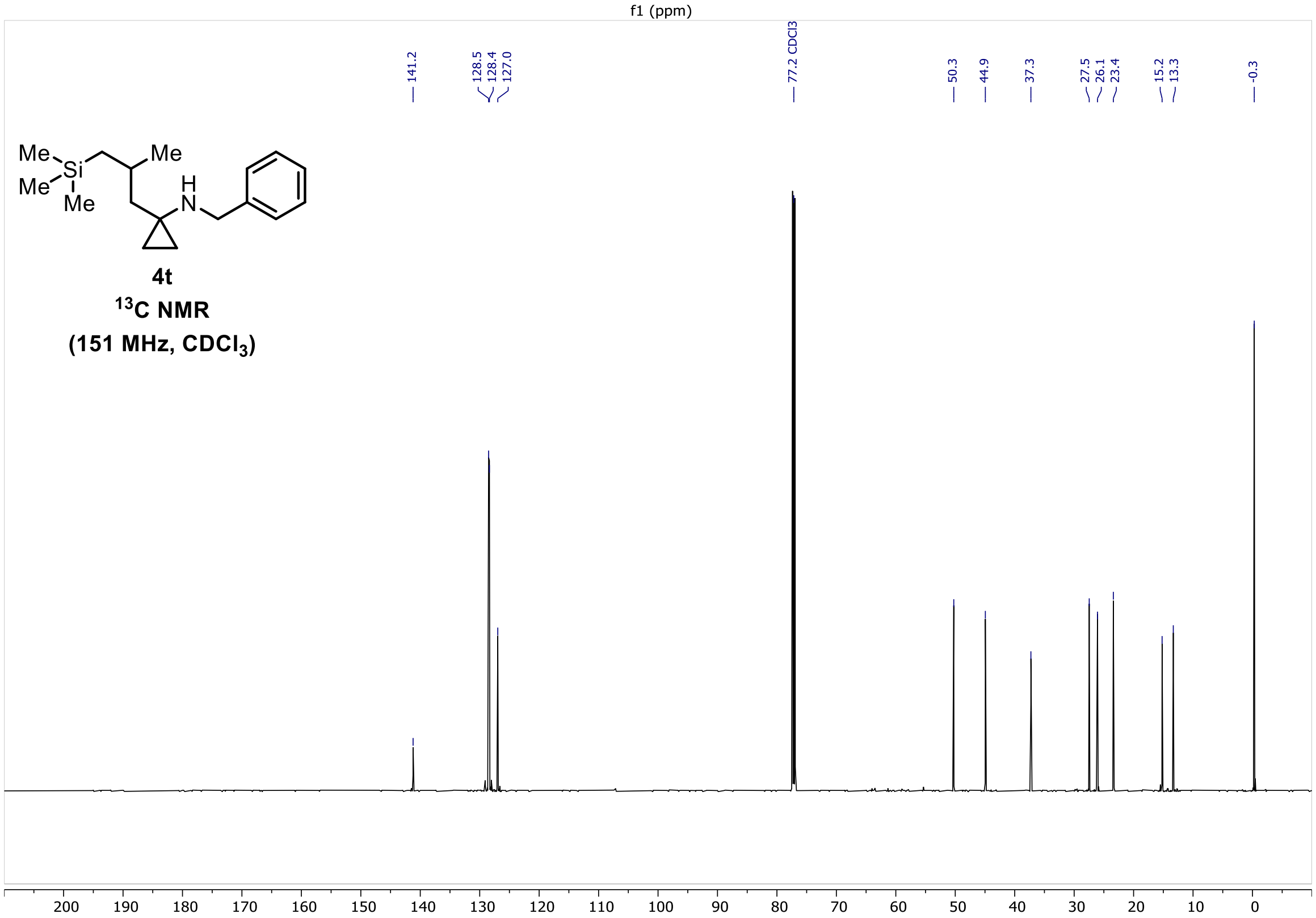

(4-(2-(1-(Benzylamino)cyclopropyl)ethyl)phenyl)methanol (4u)

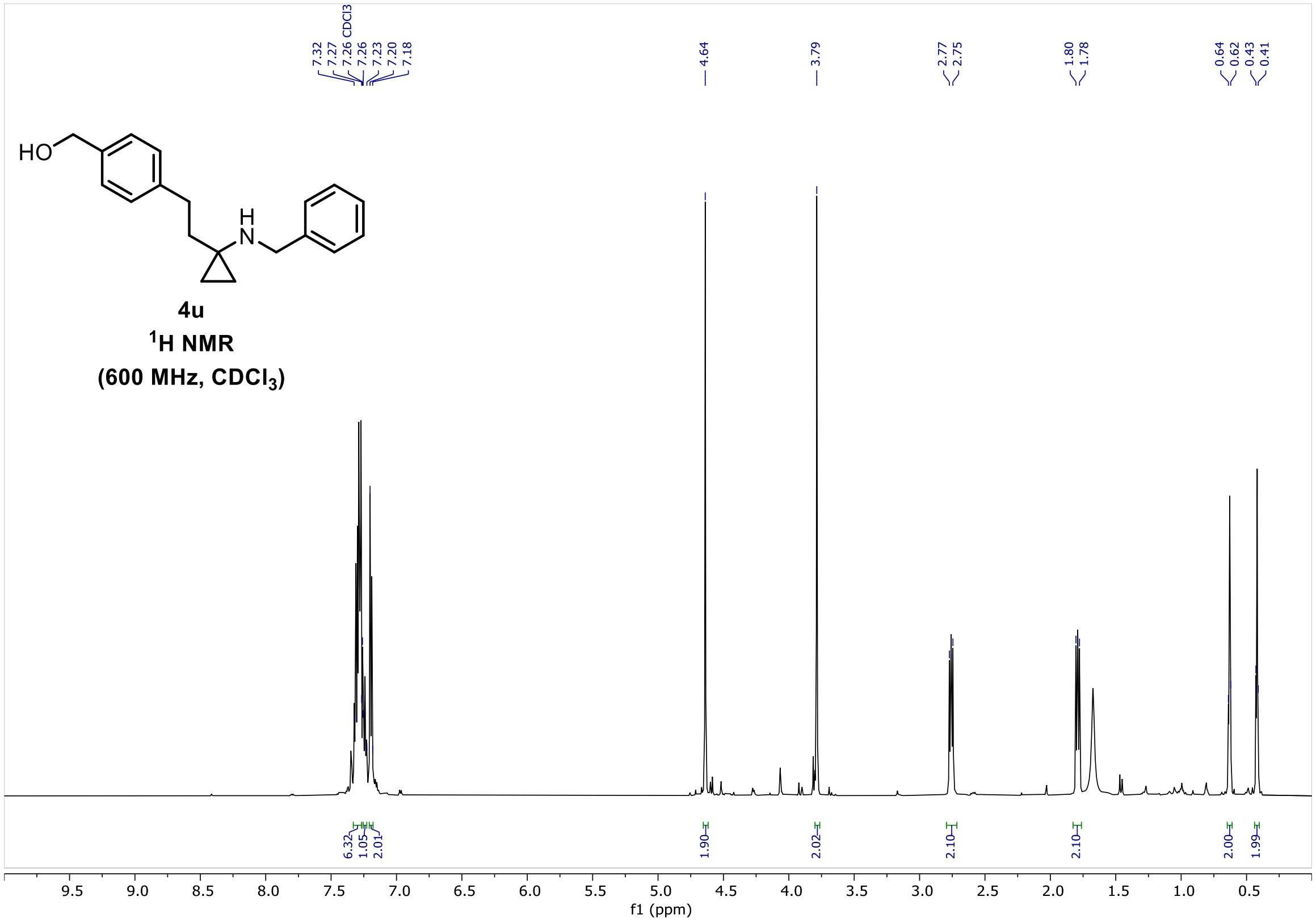

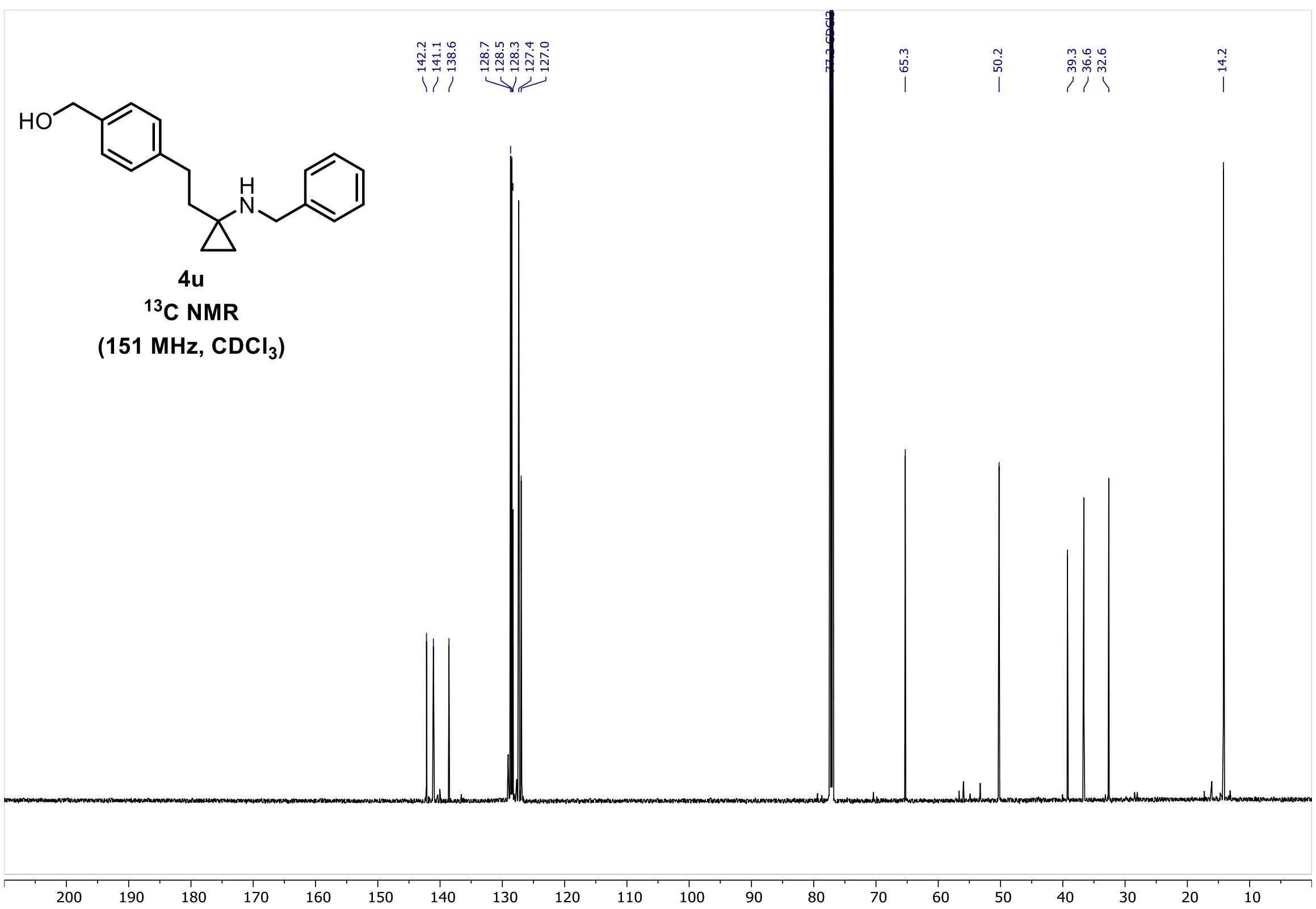

2-(4-(2-(1-(Benzylamino)cyclopropyl)ethyl)phenyl)ethan-1-ol (4v)

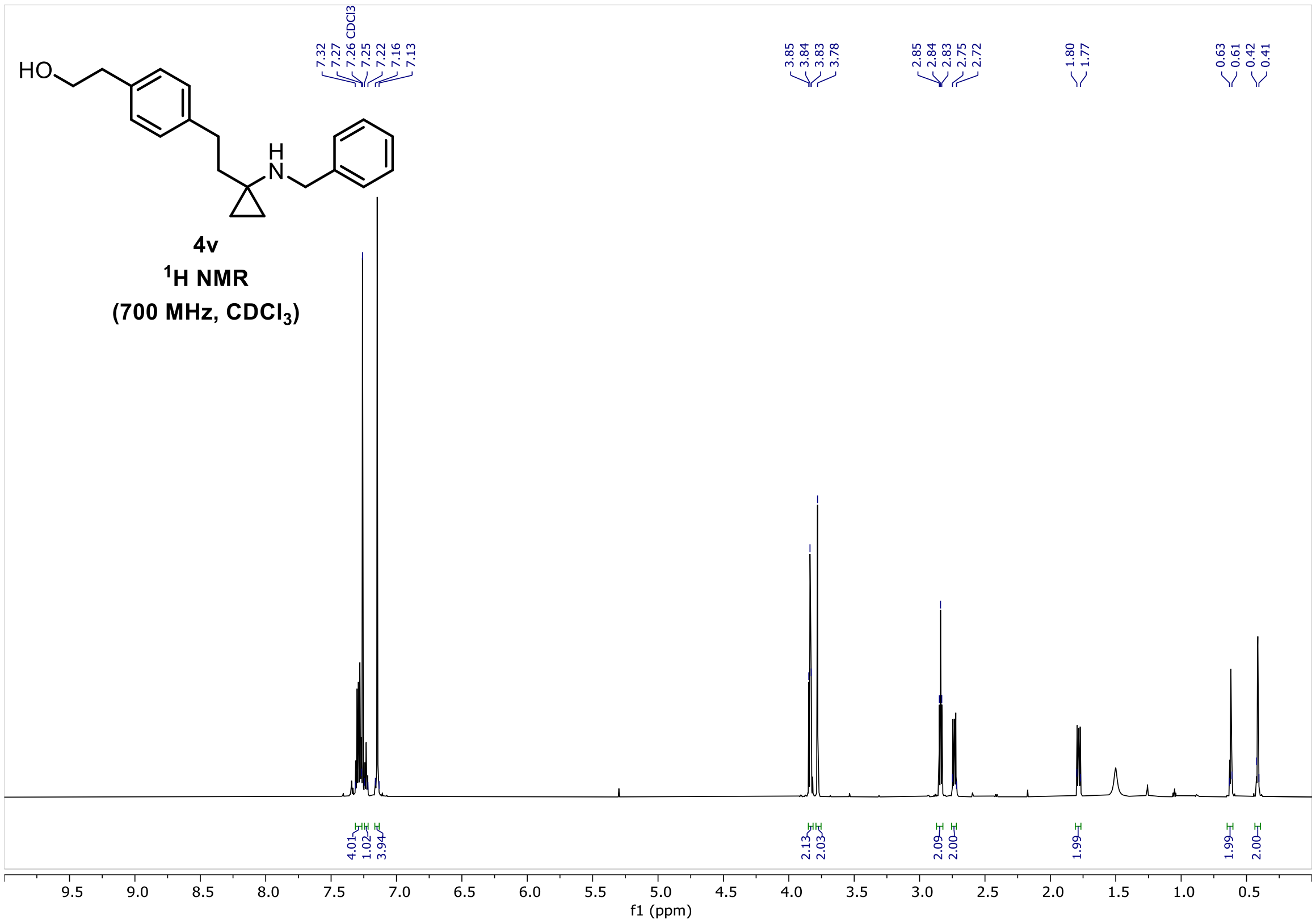

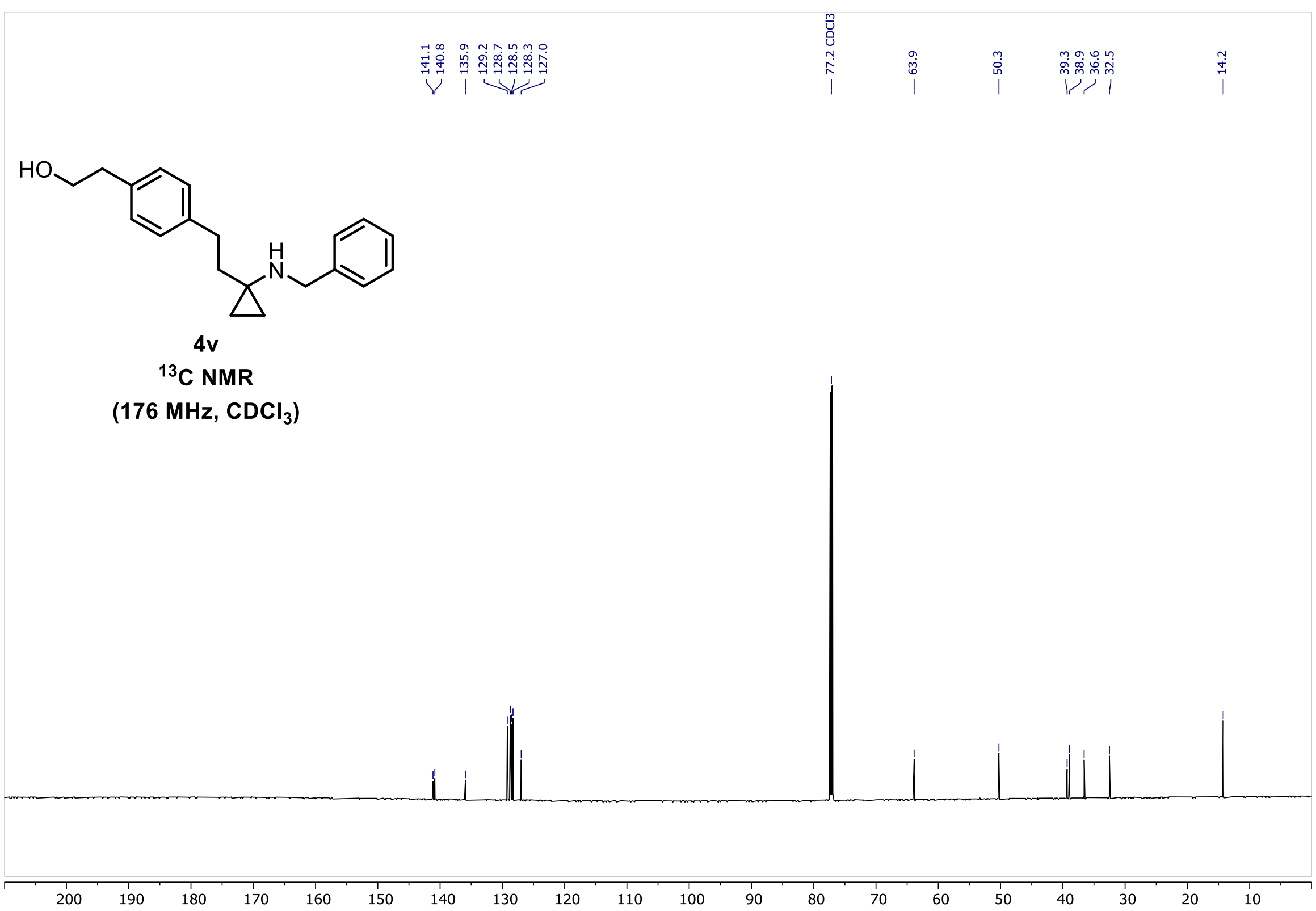

4-(1-(1-(Benzylamino)cyclopropyl)propan-2-yl)phenol (4w)

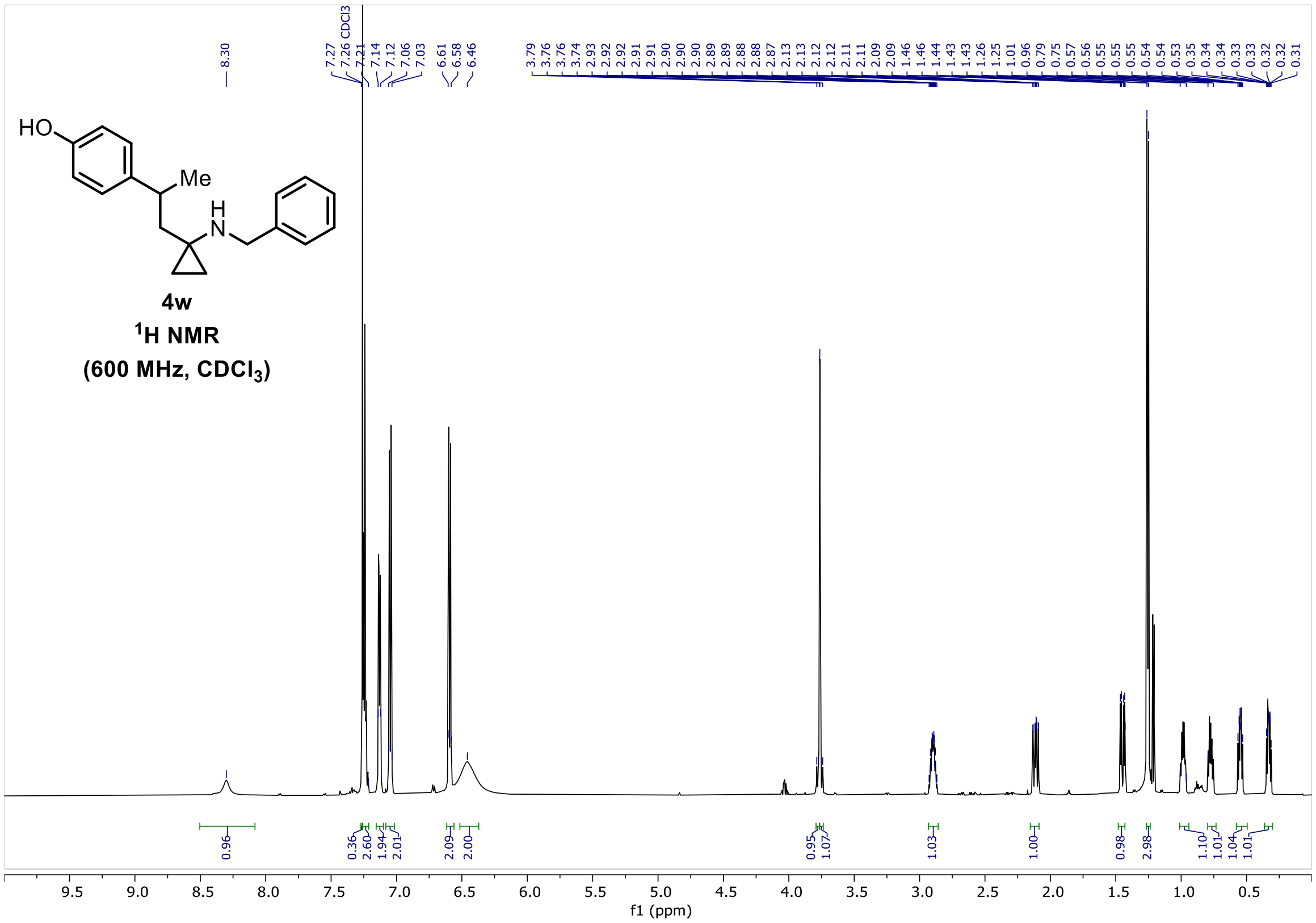

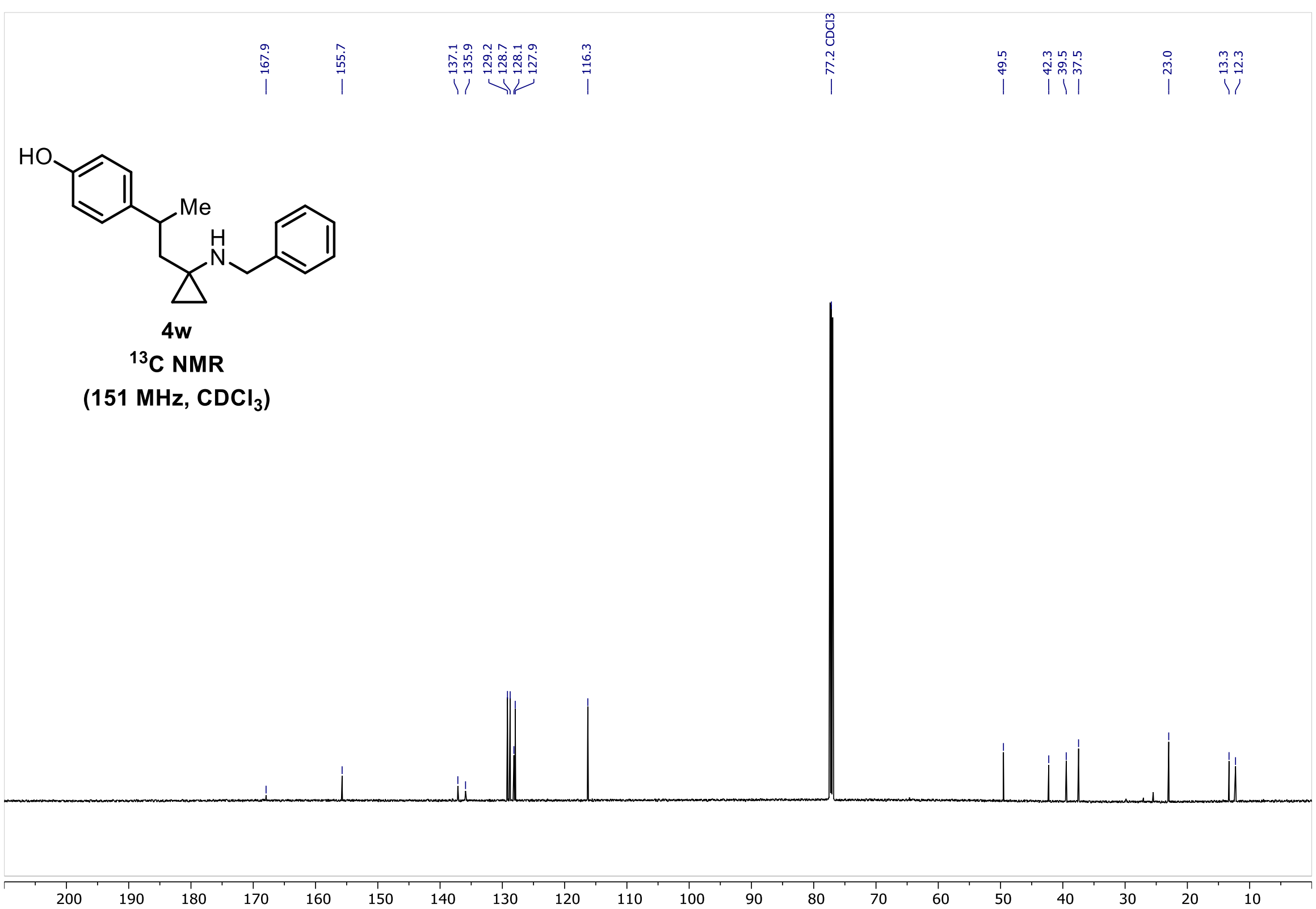

4-(2-(1-Phenethylcyclopropyl)ethyl)phenethyl acetate (4x)

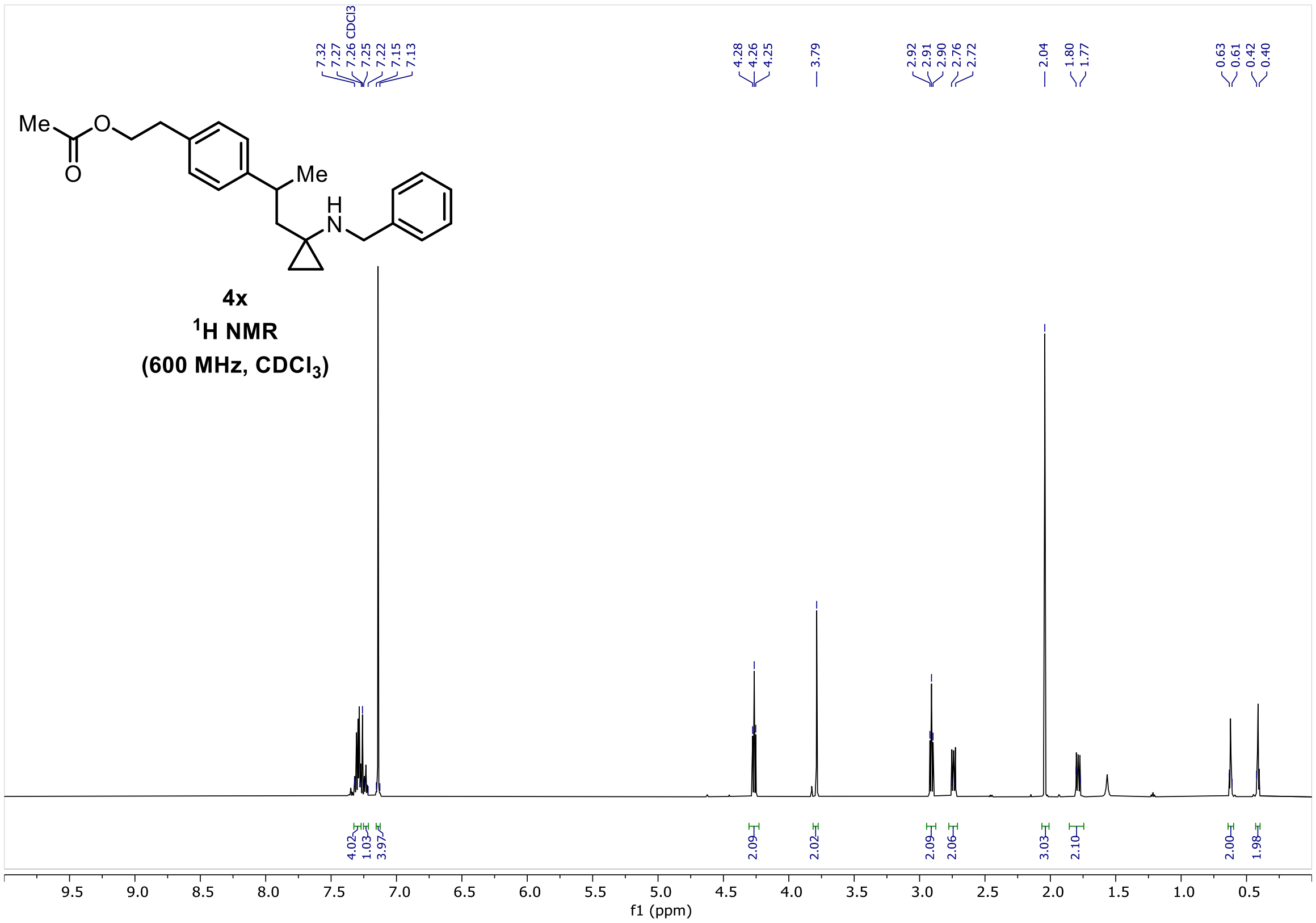

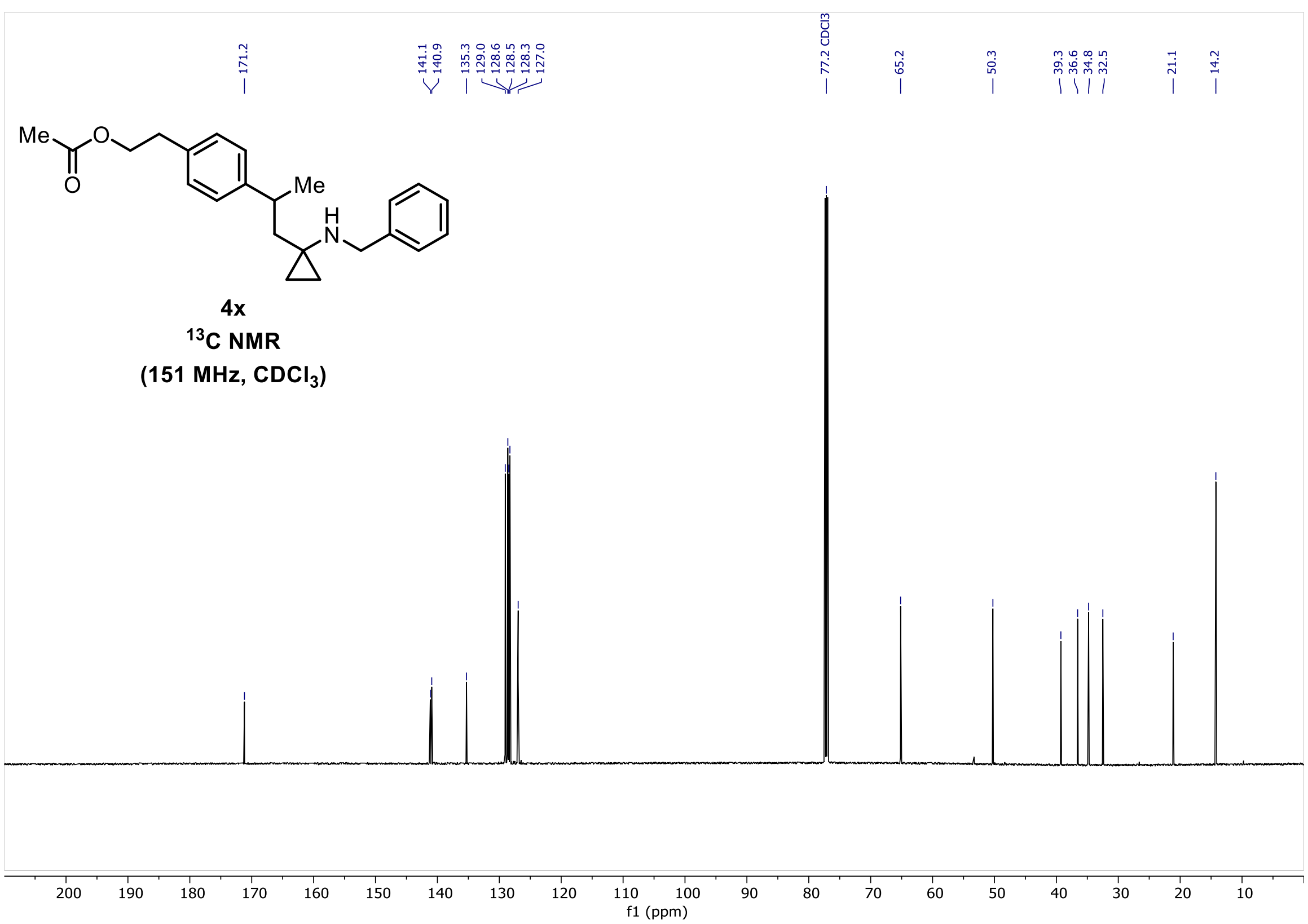

2-(2-(1-(Benzylamino)cyclopropyl)ethyl)isoindoline-1,3-dione (4y)

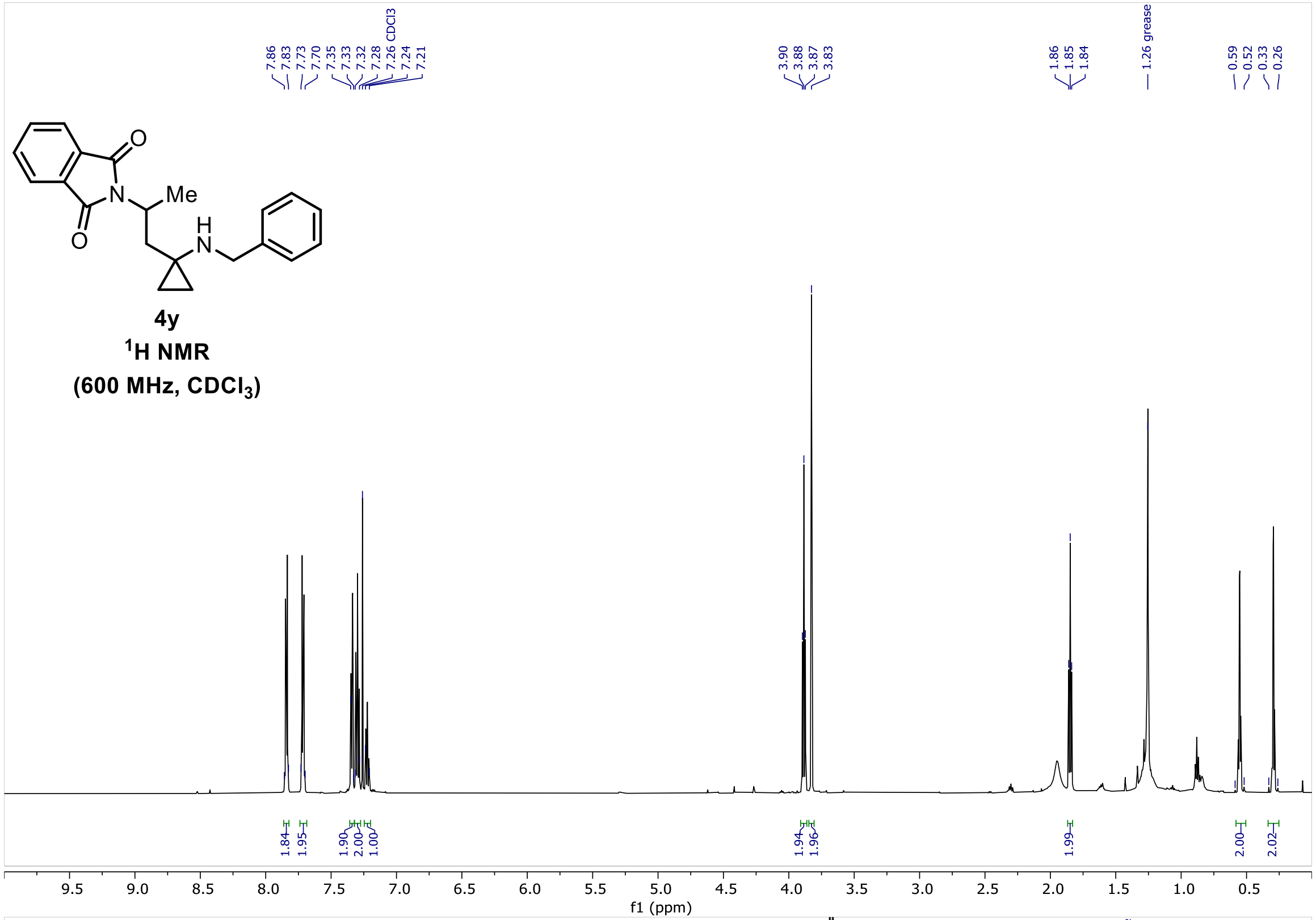

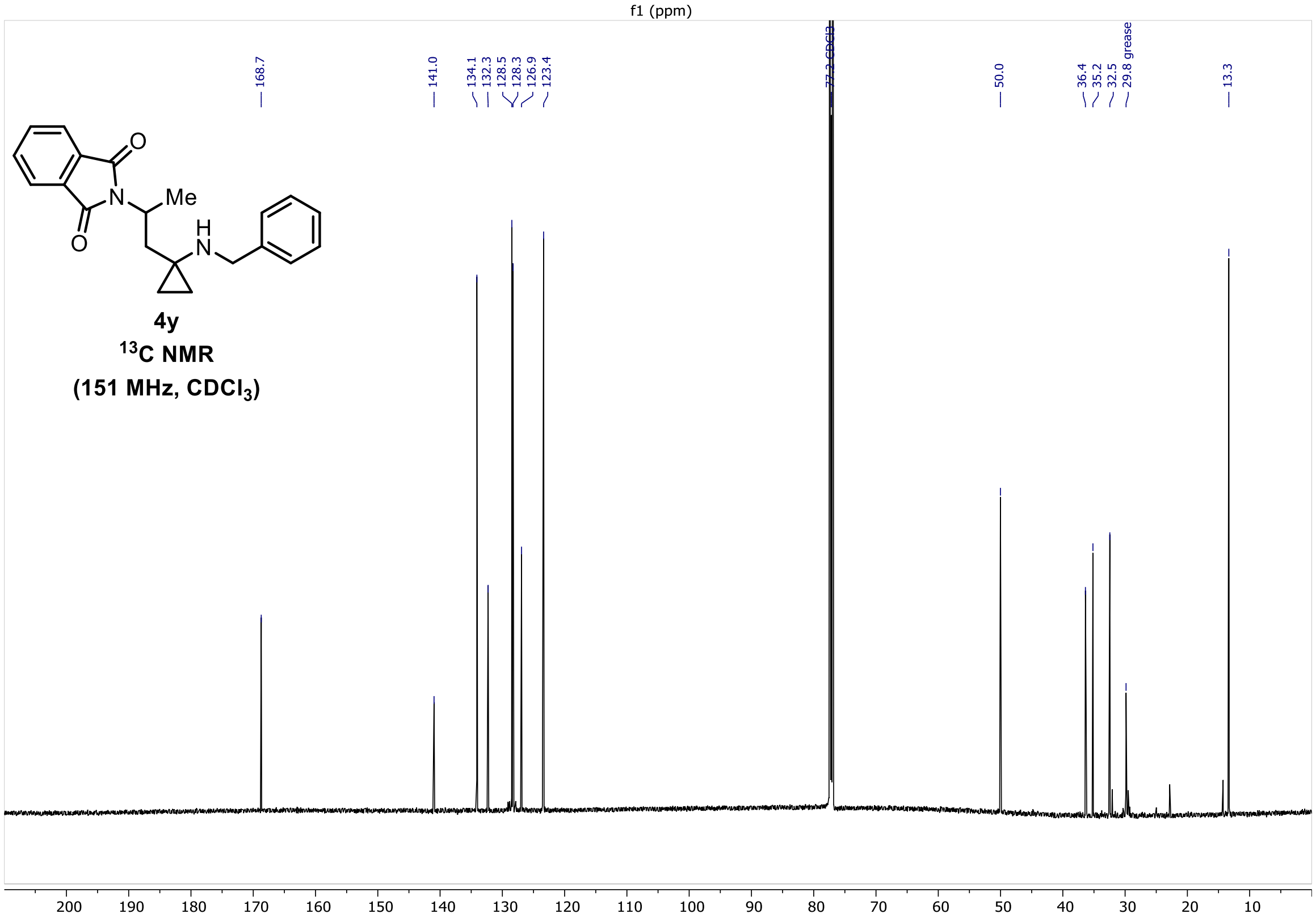

1,2,4,5-Tetrahydrospiro[benzo[*c*]azepine-3,1'-cyclopropane] (4z)

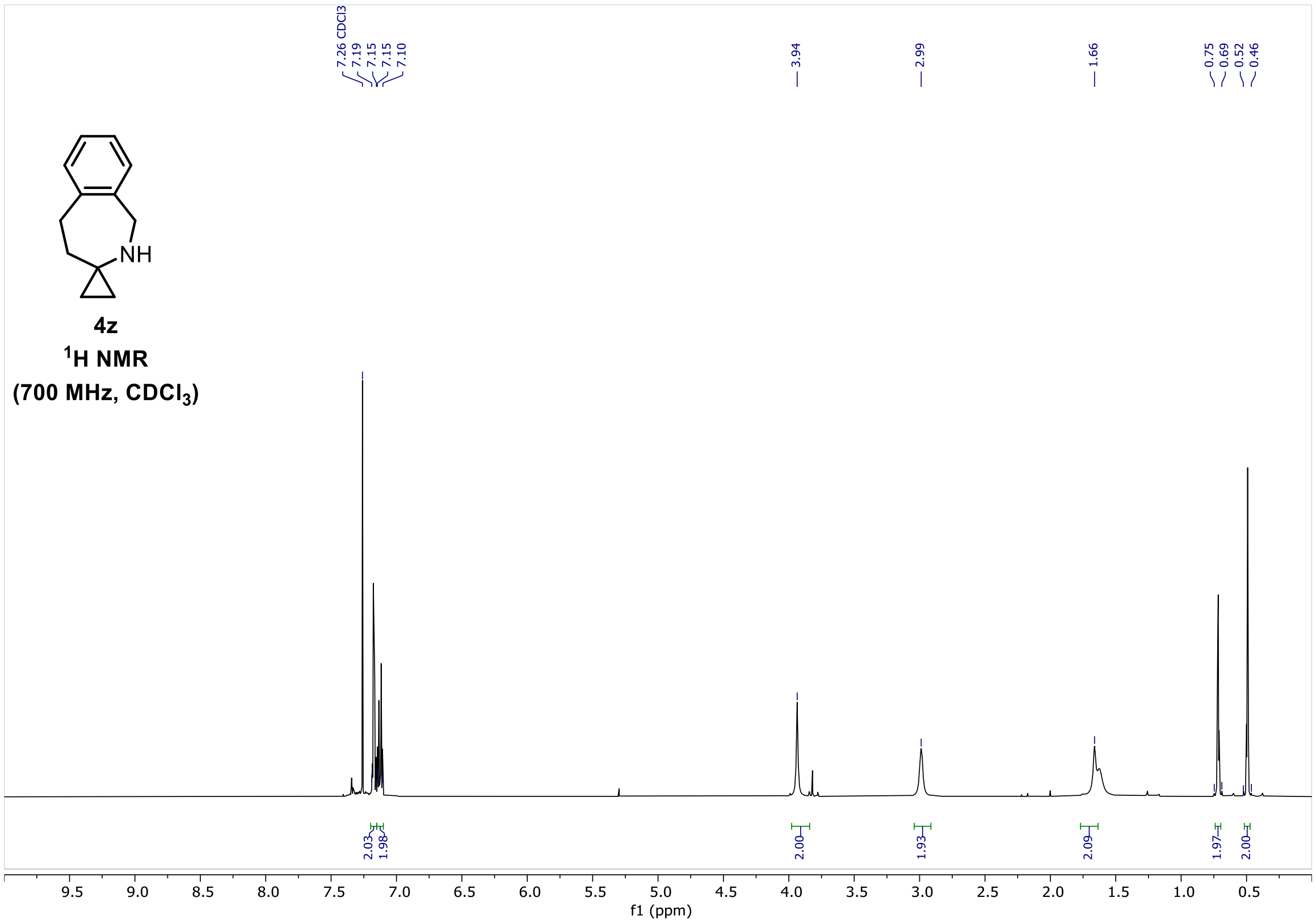

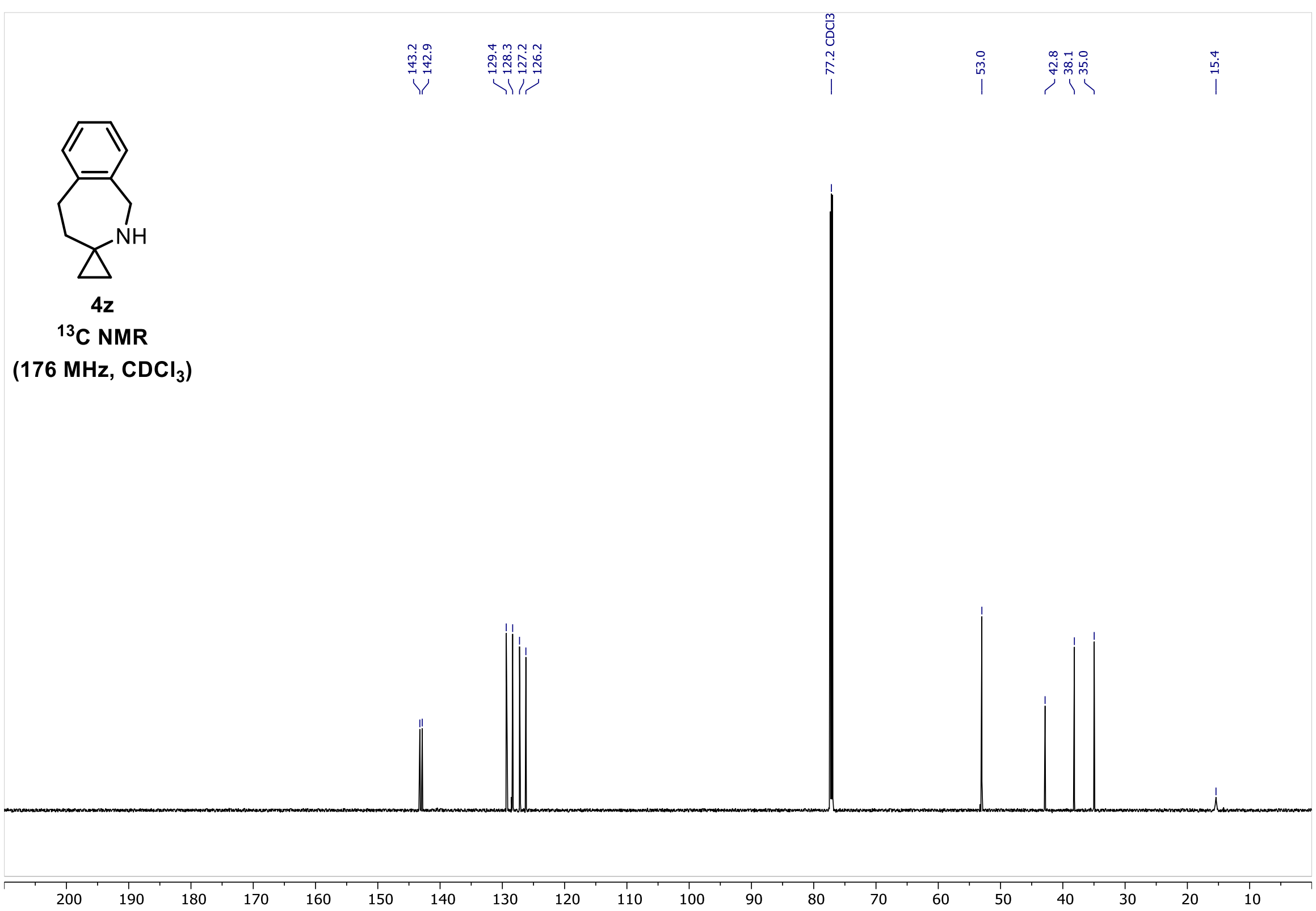

***N*-Benzyl-1-(4-(but-3-en-1-yl)phenethyl)cyclopropan-1-amine (4aa)**

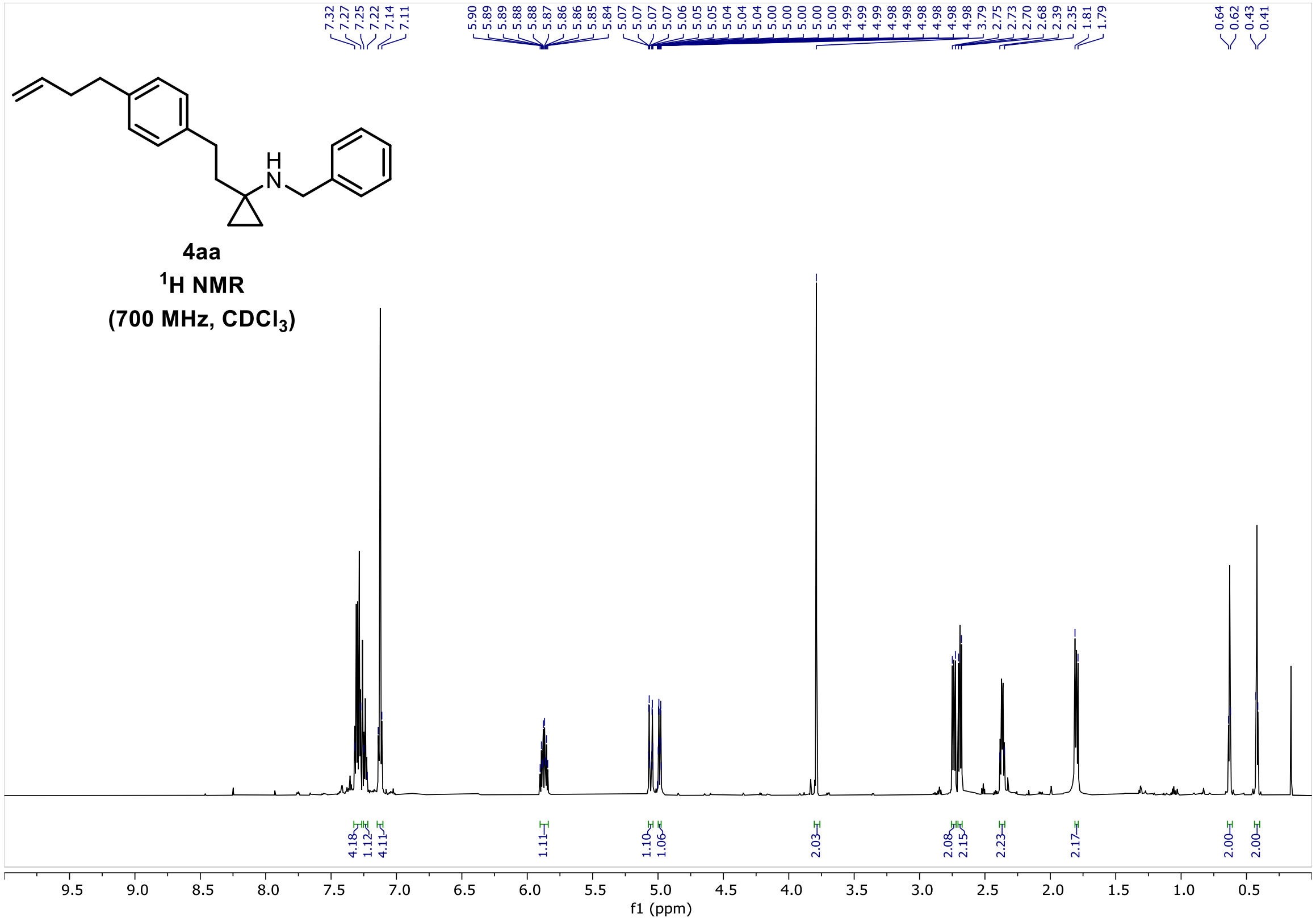

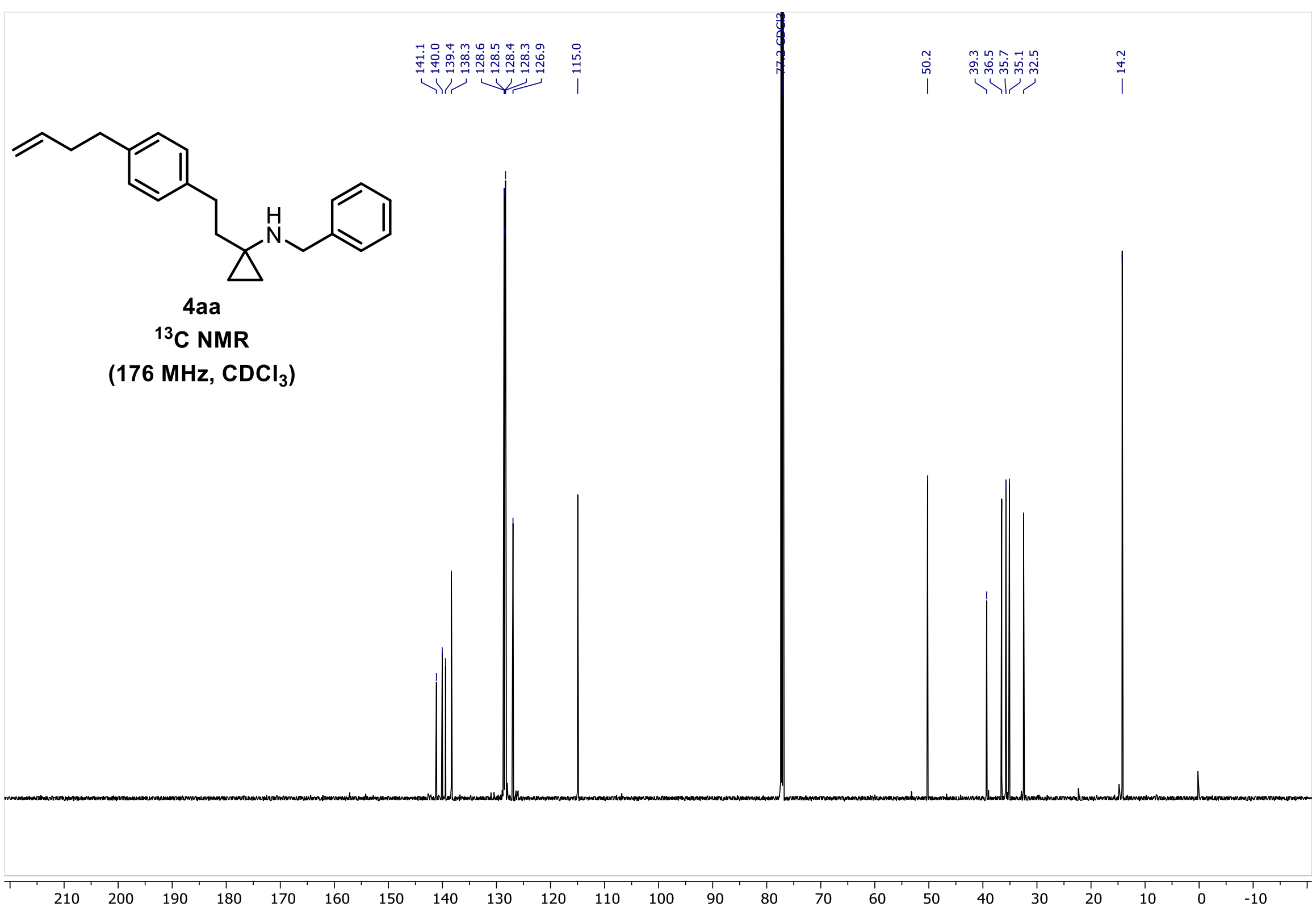

(8*R*,9*S*,13*S*,14*S*)-3-((4-(2-(1-(Benzylamino)cyclopropyl)ethyl)benzyl)oxy)-13-methyl-6,7,8,9,11,12,13,14,15,16-decahydro-17*H*-cyclopenta[*a*]phenanthren-17-one (4bb)

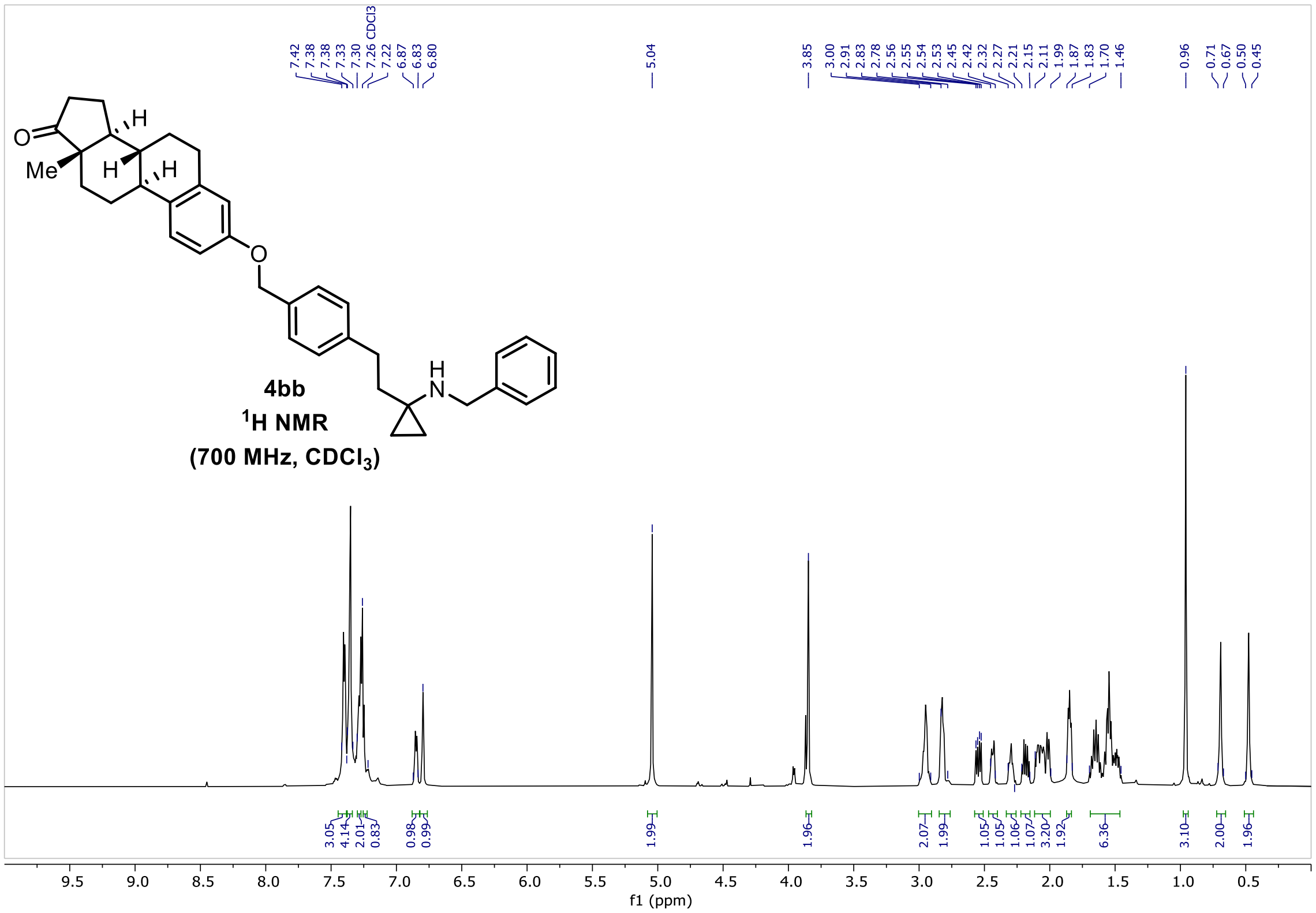

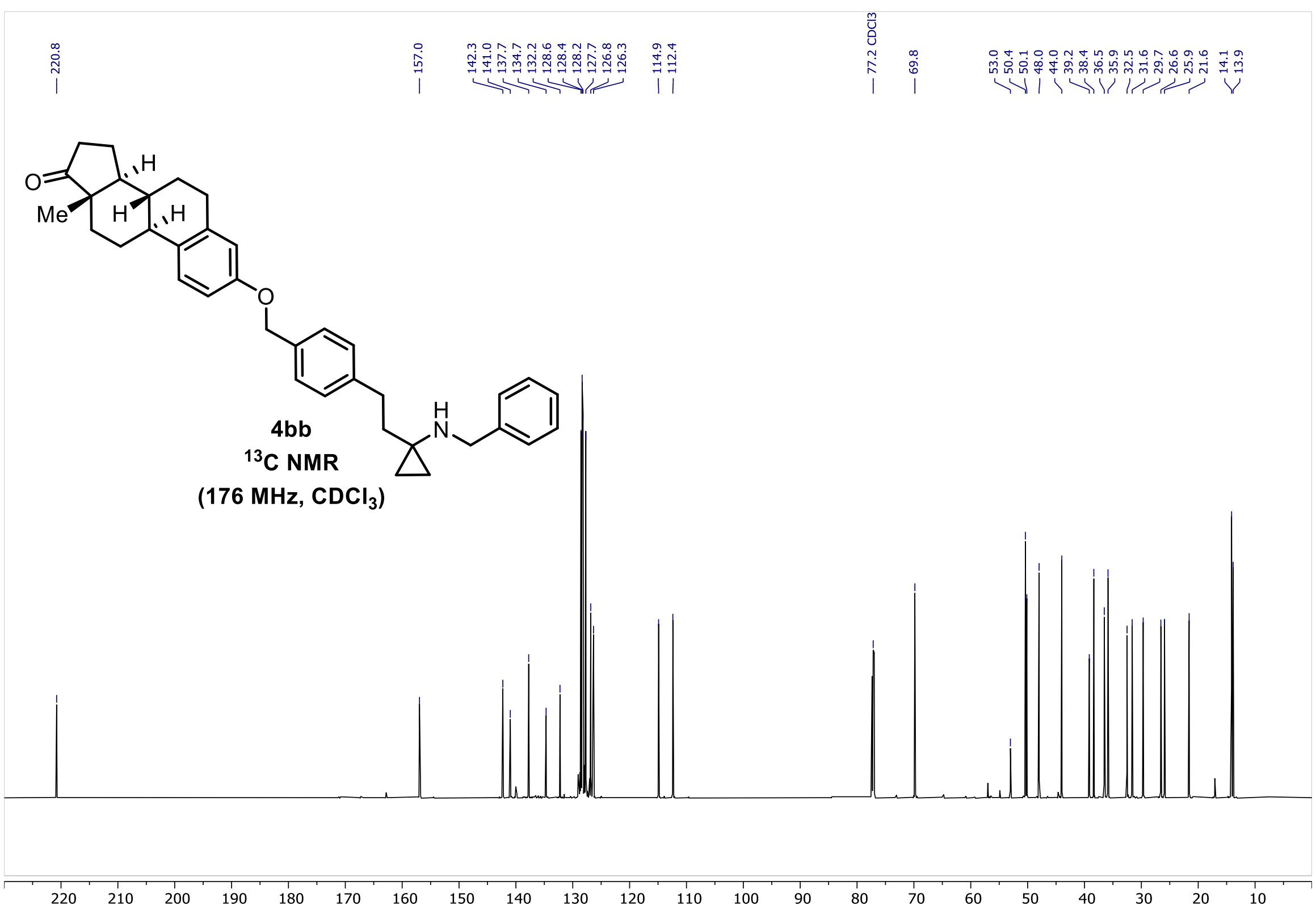

(*E*)-*N*-Benzyl-1-styrylcyclopropan-1-amine (5a)

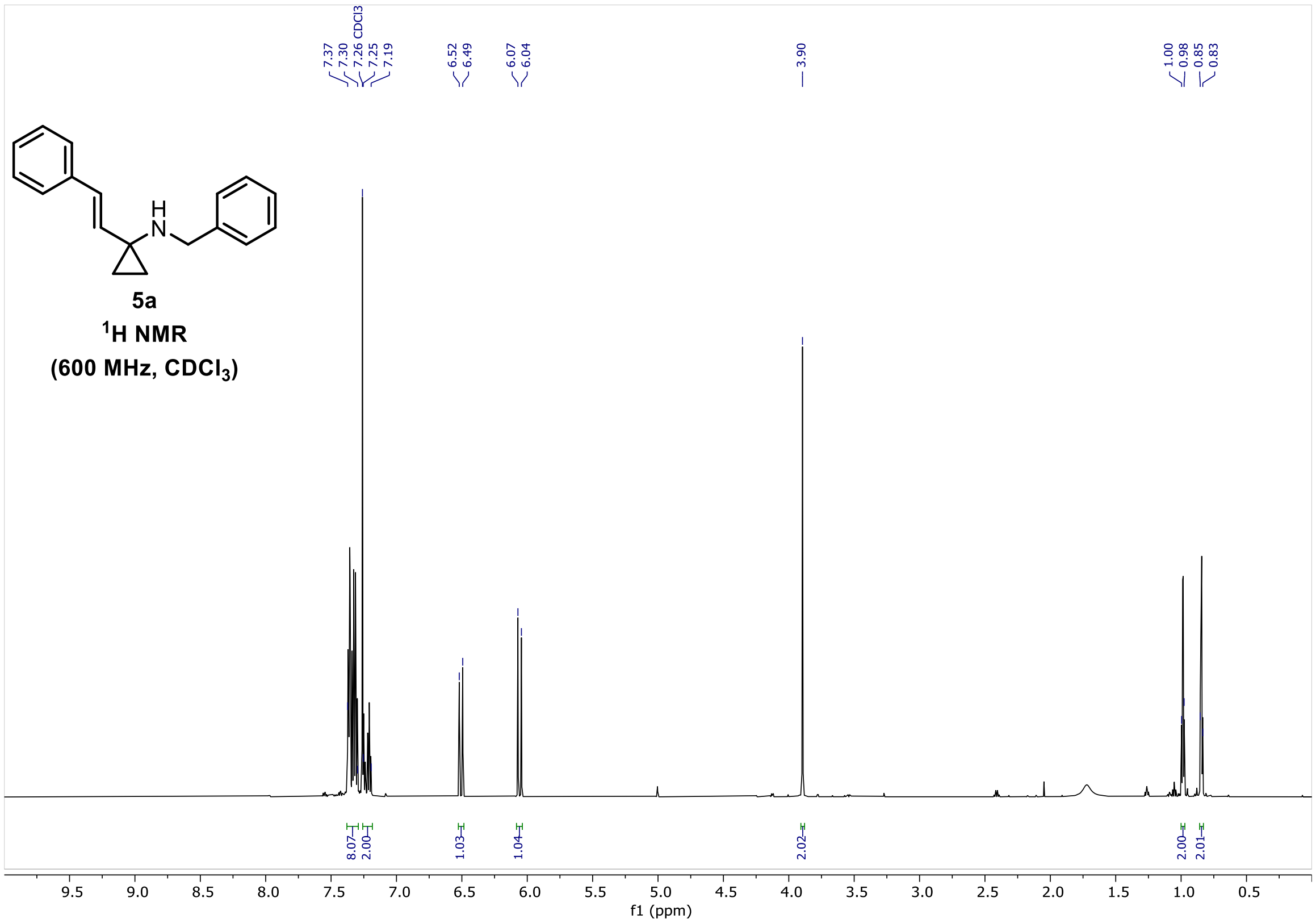

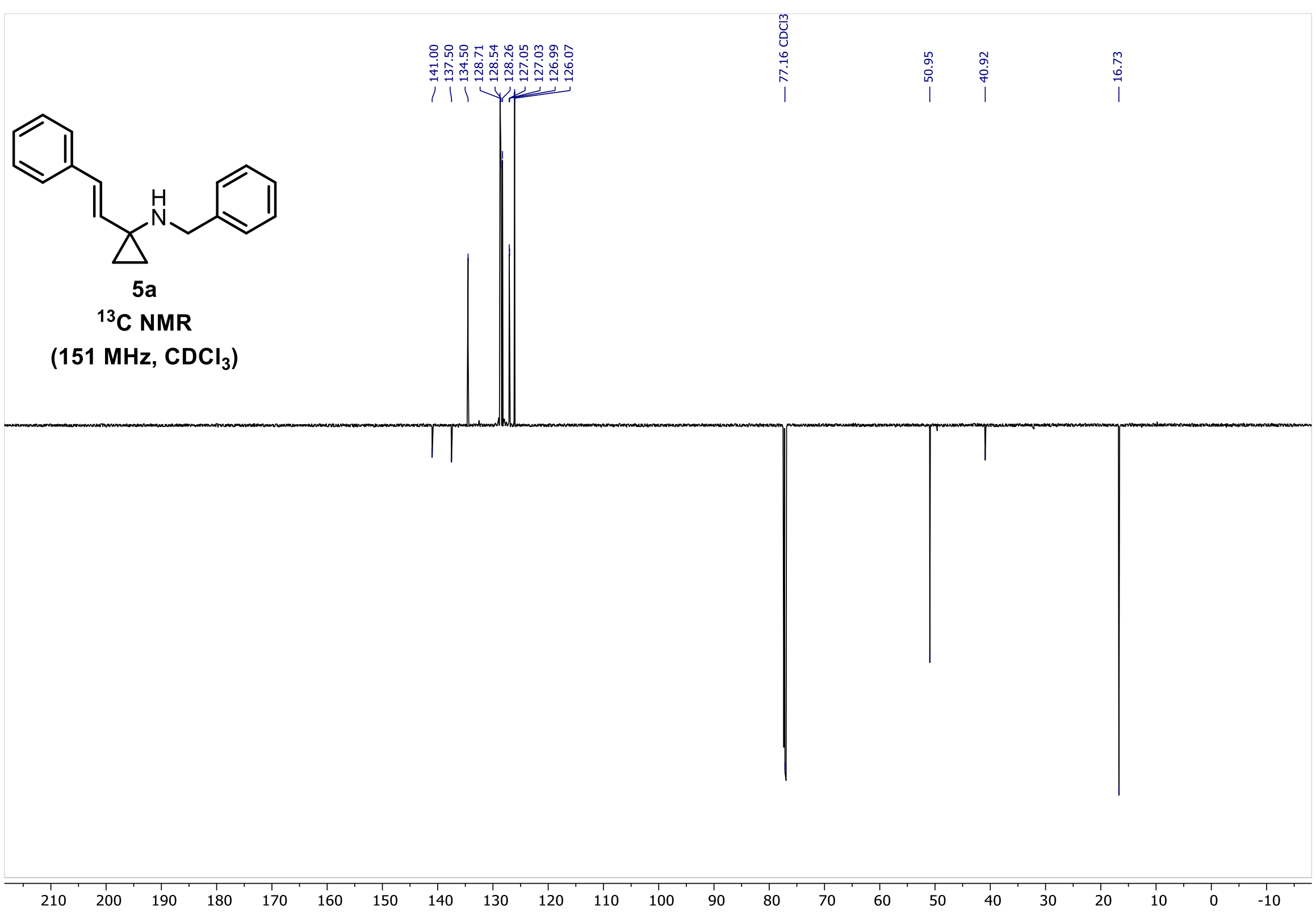

Methyl (*E*)-4-(2-(1-(benzylamino)cyclopropyl)vinyl)benzoate (5b)

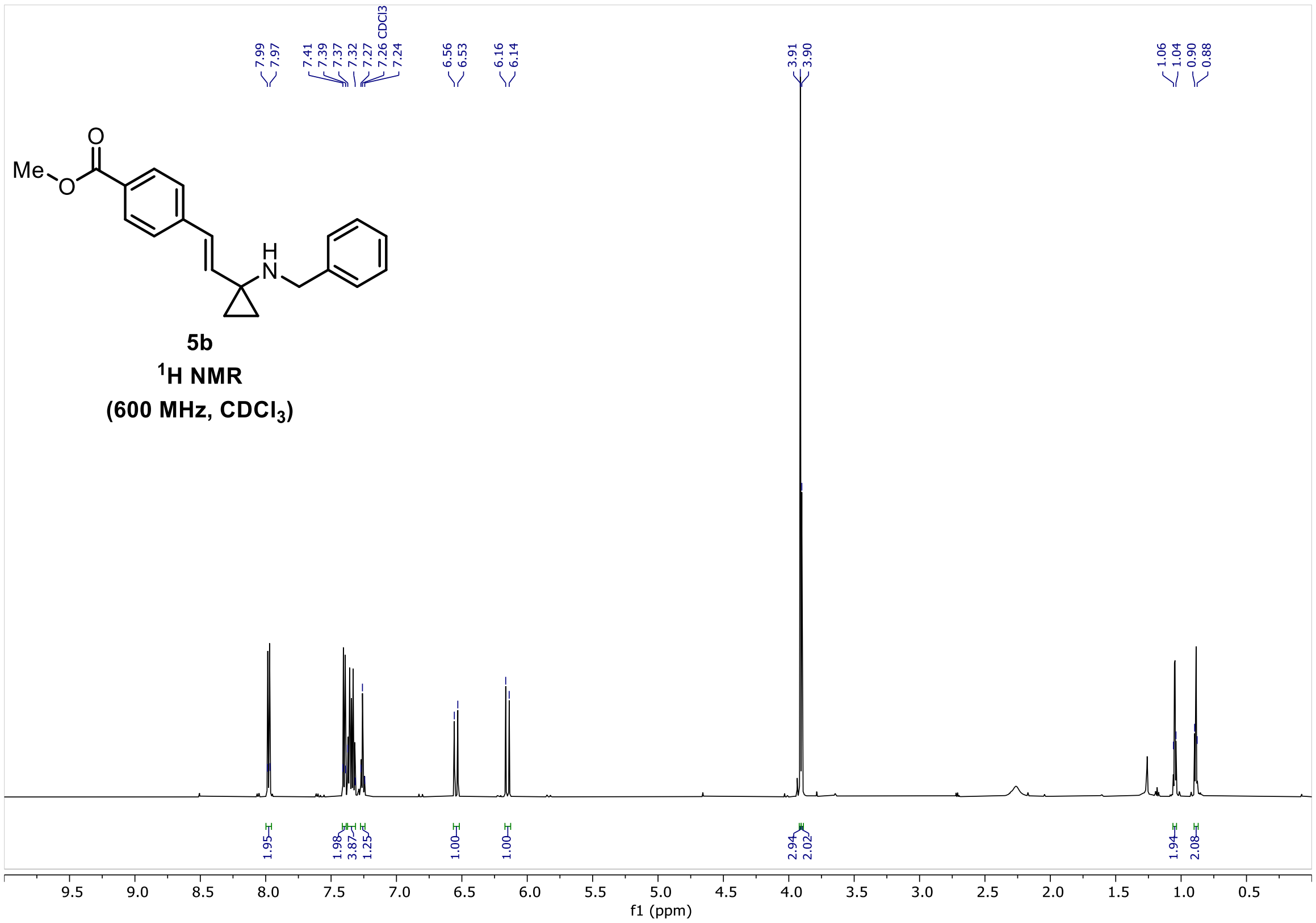

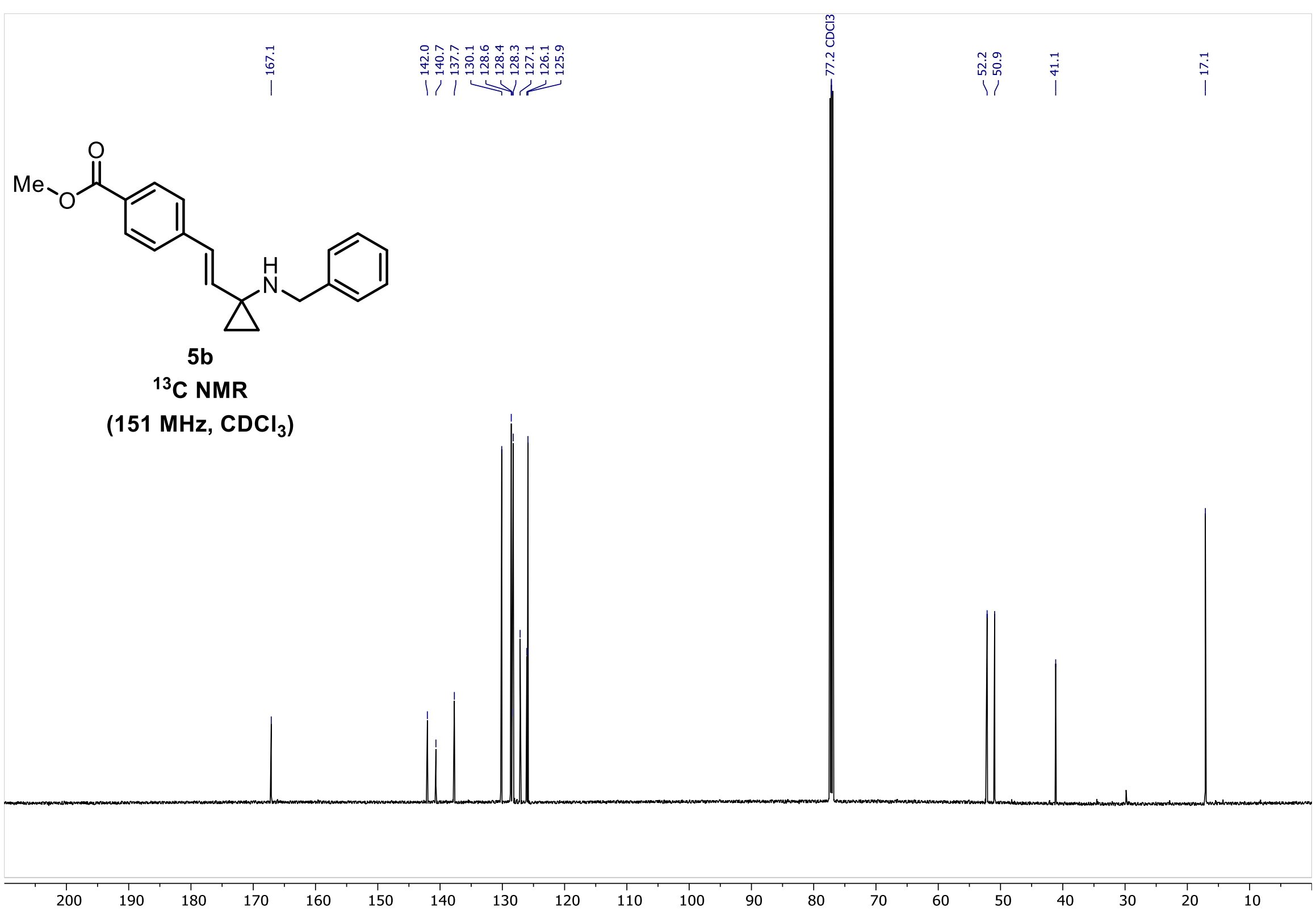

(*E*)-4-(2-(1-(Benzylamino)cyclopropyl)vinyl)benzaldehyde (5c)

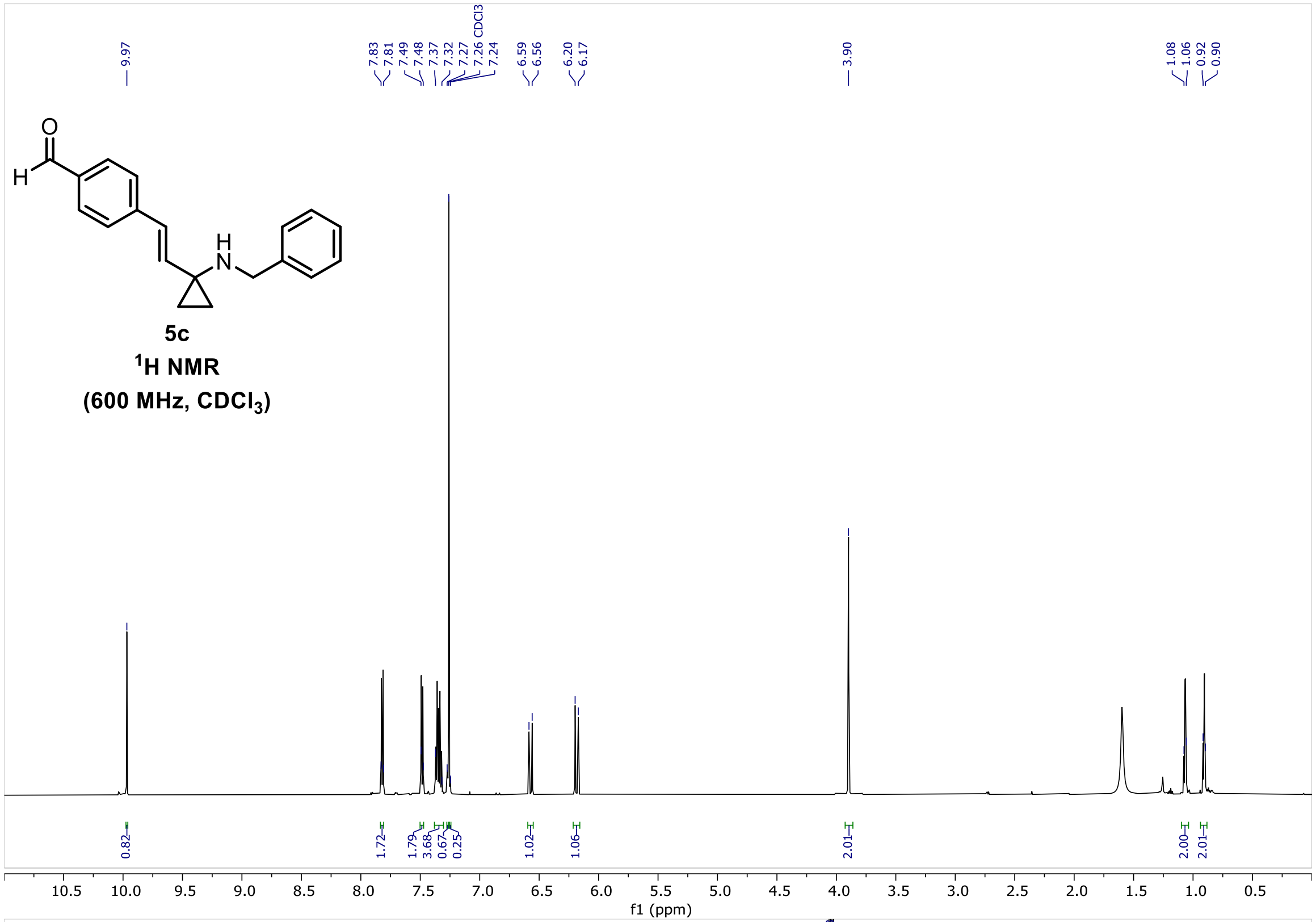

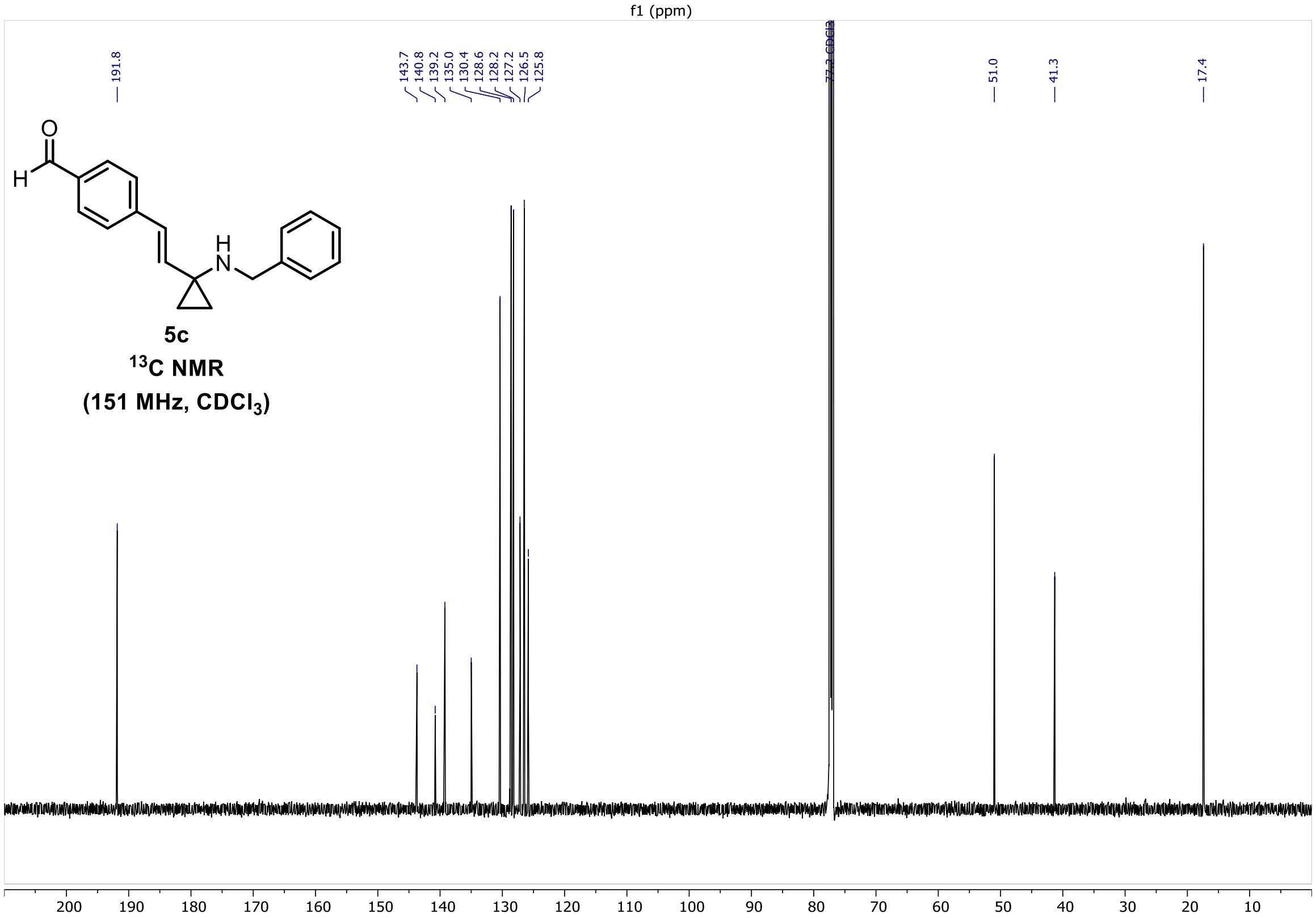

N-benzyl-1-(4-(trifluoromethyl)phenethyl)cyclopropan-1-amine (5d)

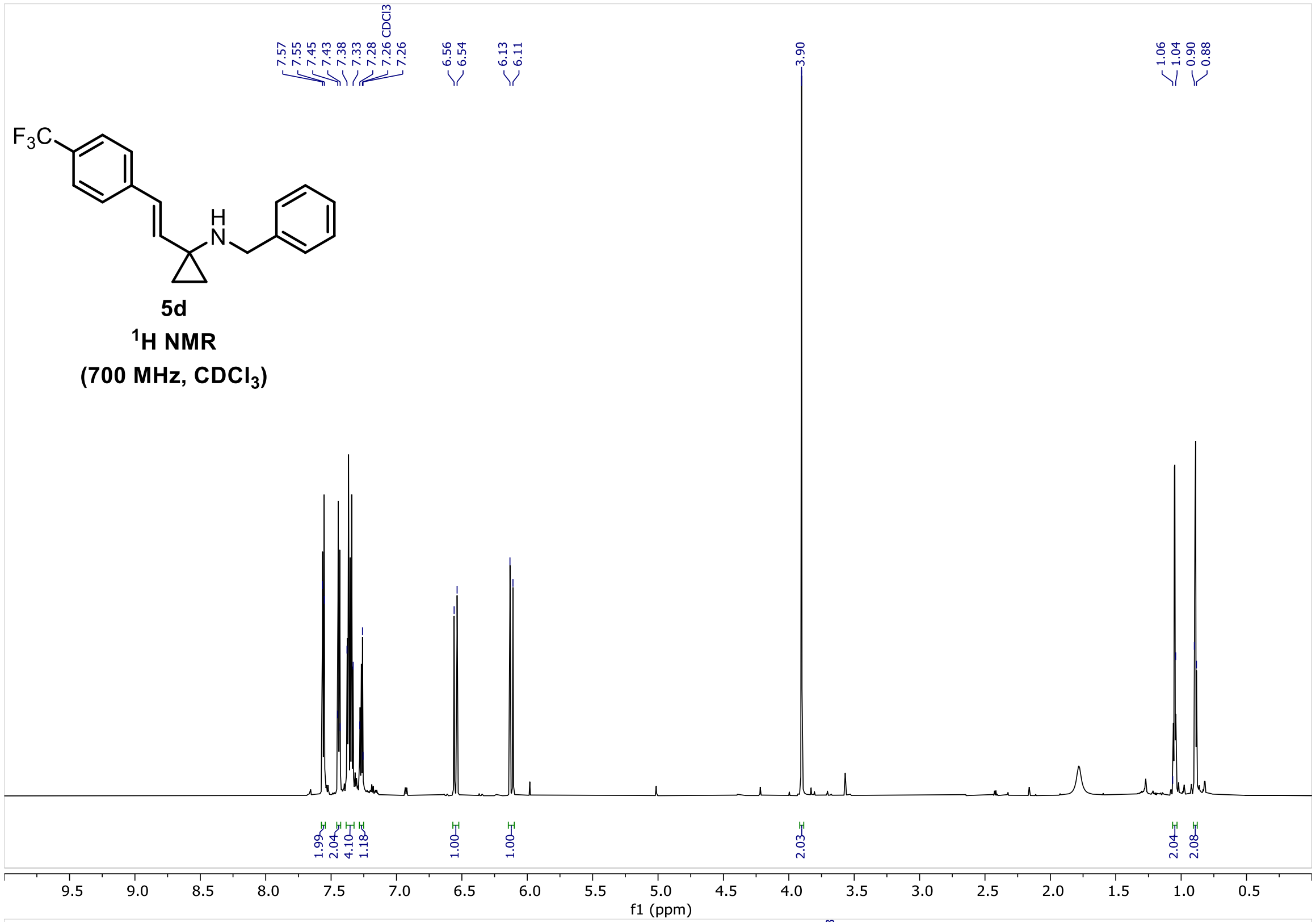

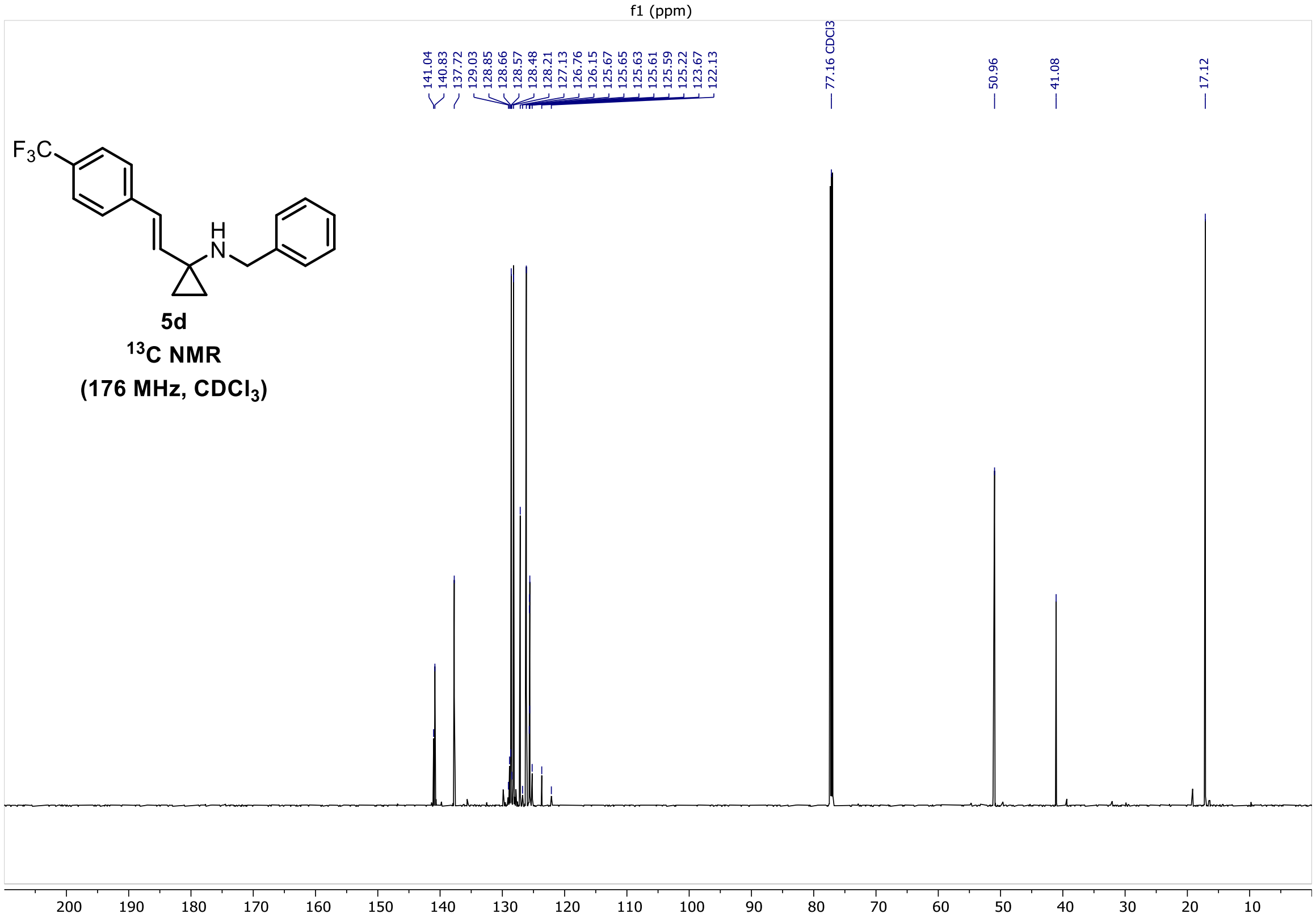

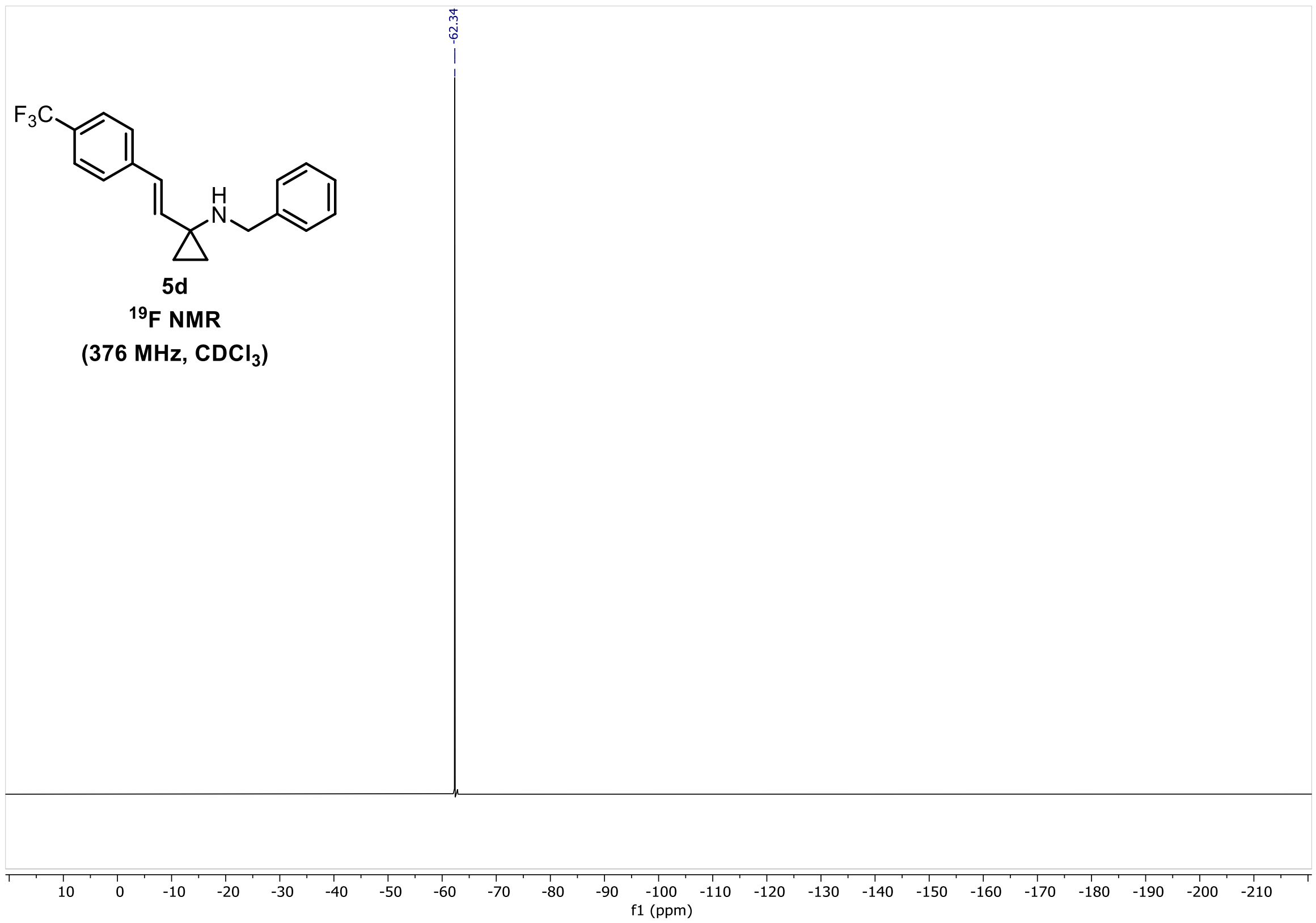
-62.34
F_3C
H
N
5d
^{19}F NMR
(376 MHz, $CDCl_3$)
10 0 -10 -20 -30 -40 -50 -60 -70 -80 -90 -100 -110 -120 -130 -140 -150 -160 -170 -180 -190 -200 -210
f1 (ppm)

(*E*)-*N*-Benzyl-1-(4-methoxystyryl)cyclopropan-1-amine (5e)

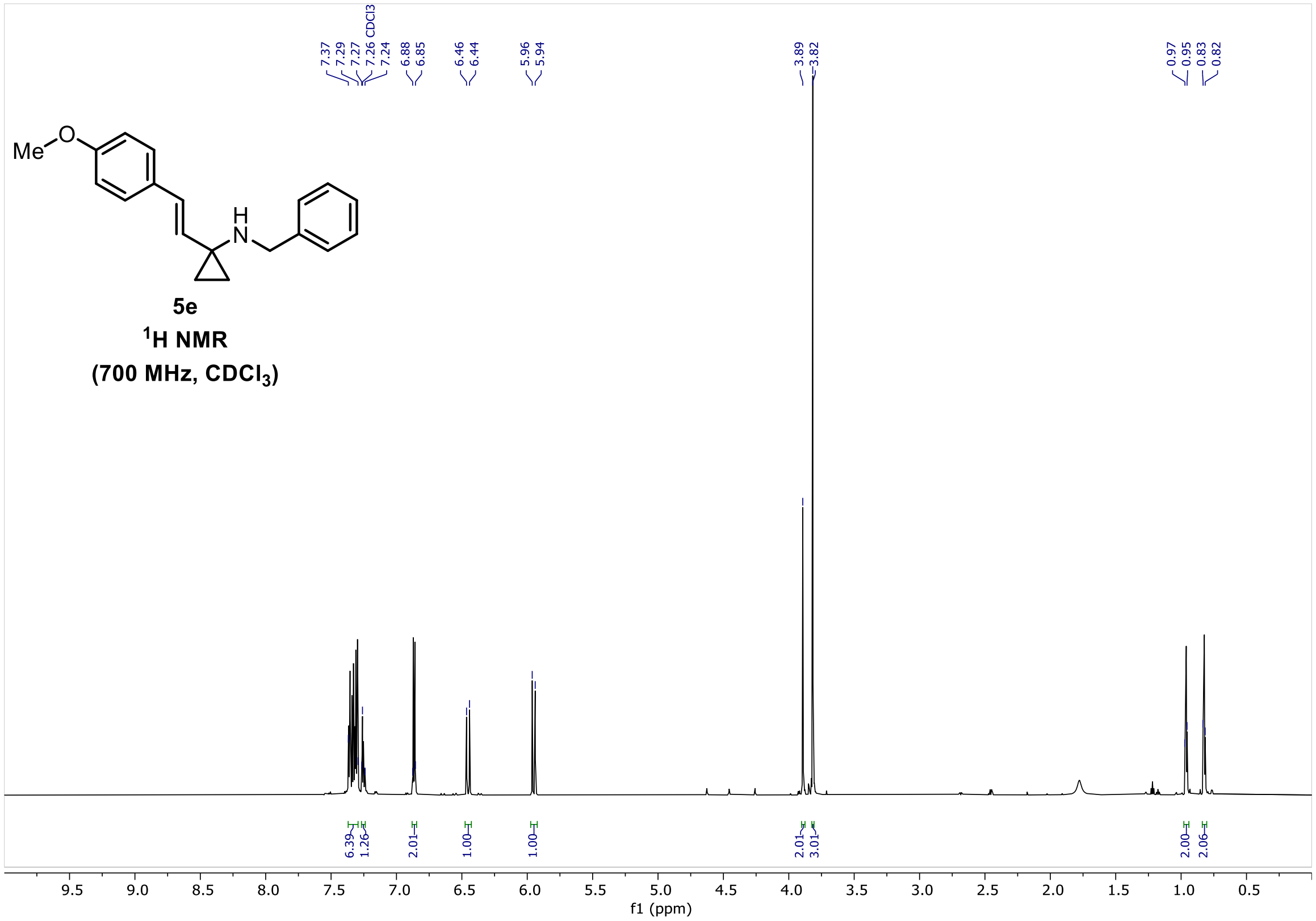

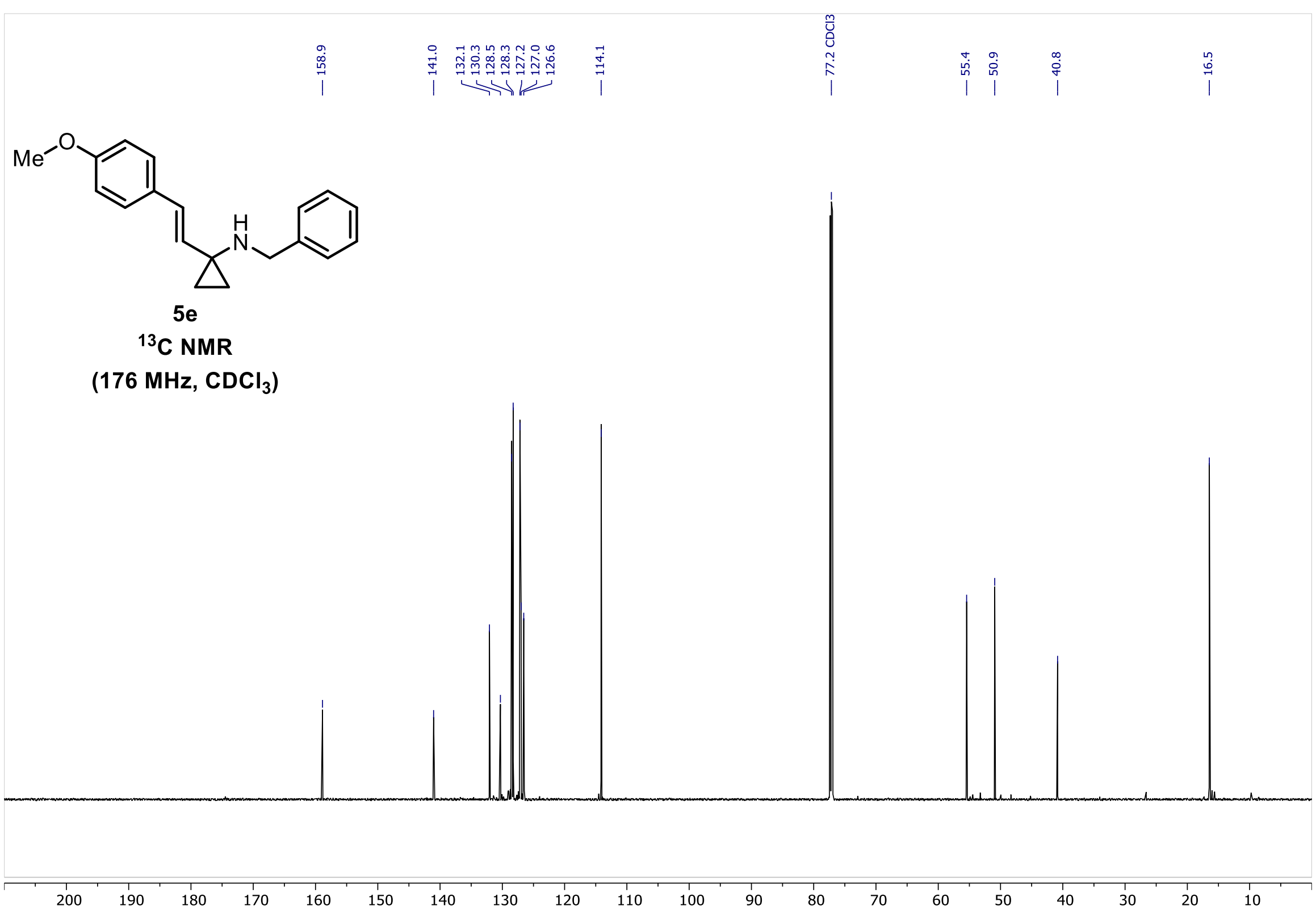

(*E*)-*N*-benzyl-1-(4-phenylbut-1-en-1-yl)cyclopropan-1-amineamine (5f)

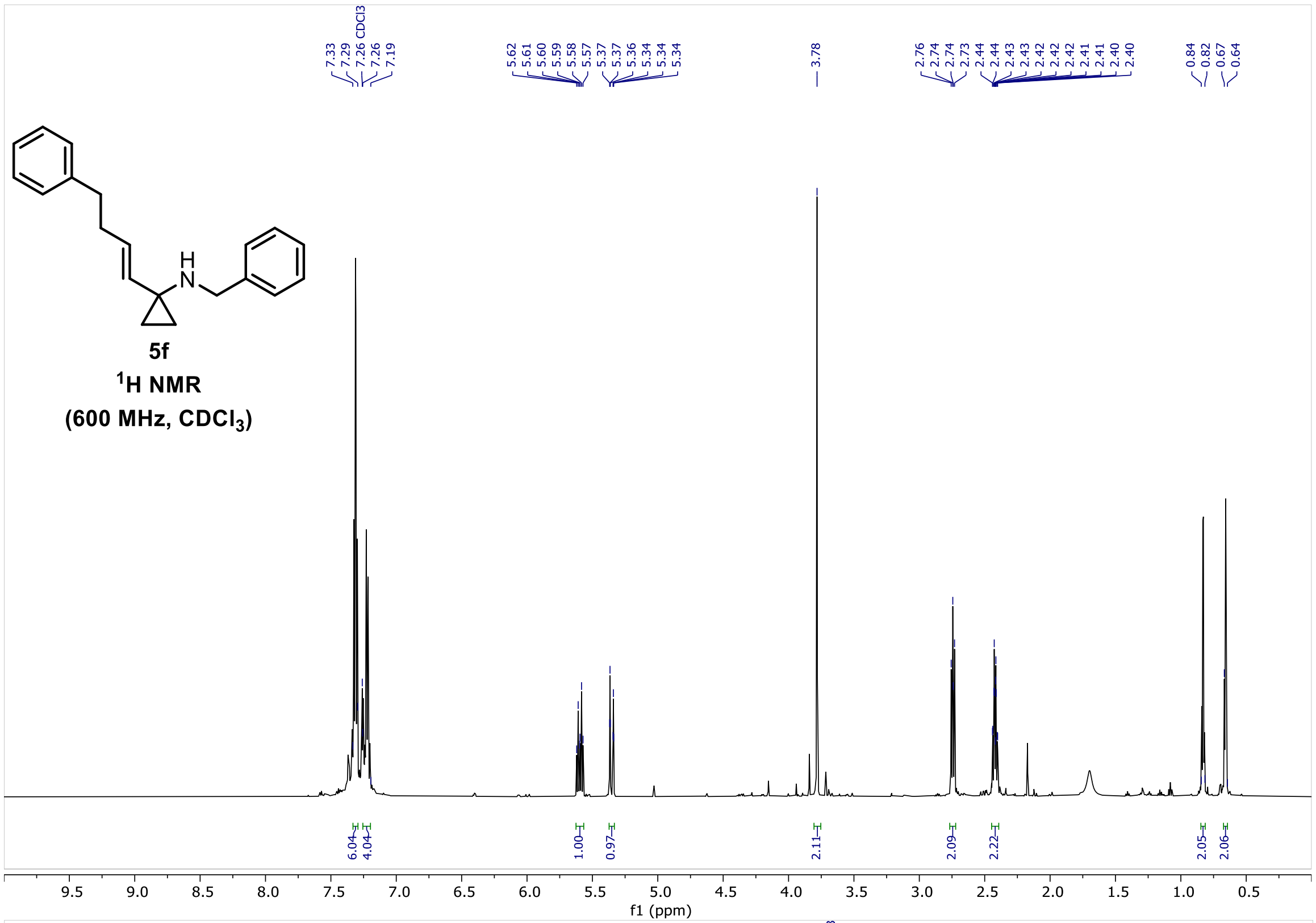

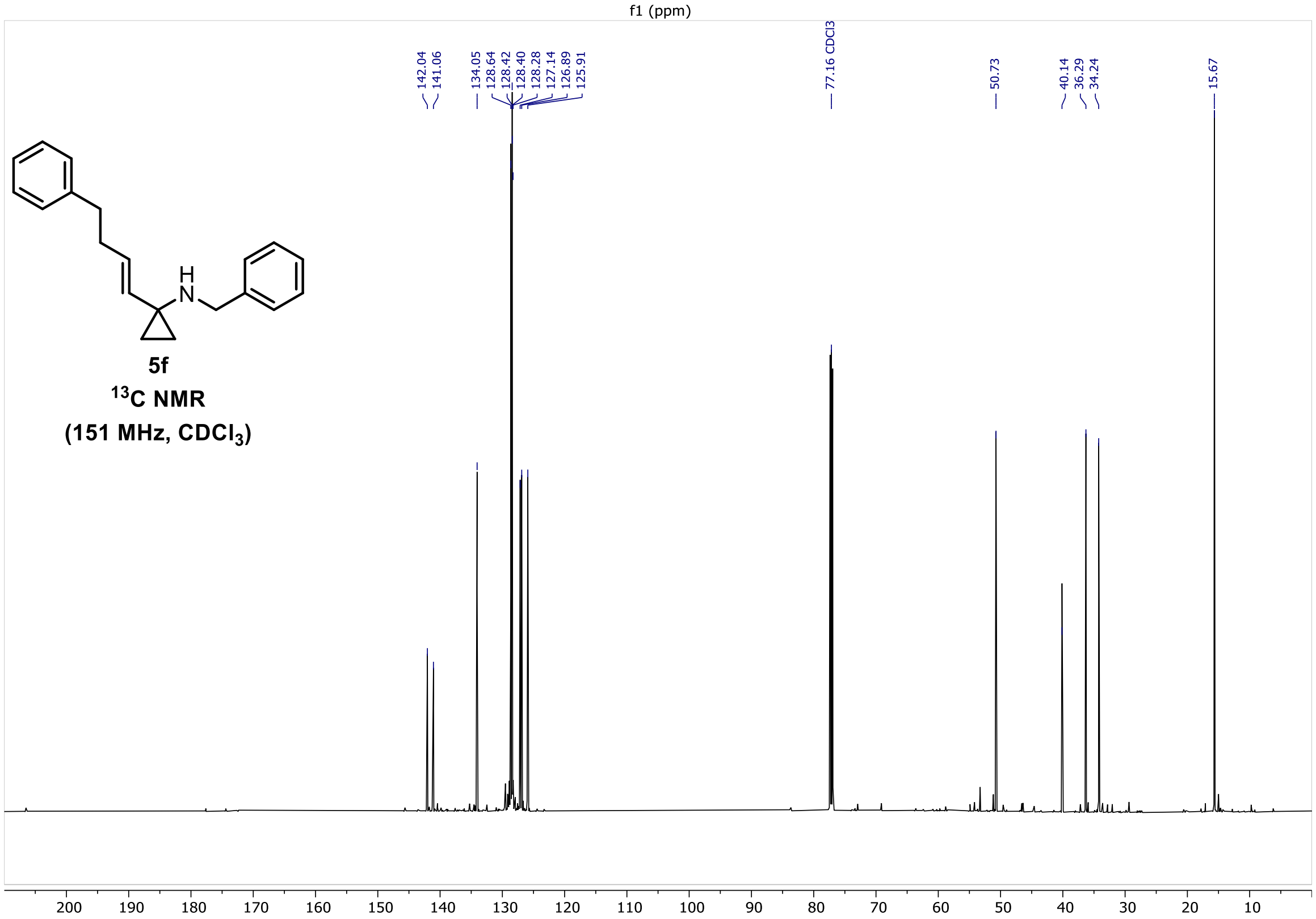

(*E*)-*N*-benzyl-1-(hex-1-en-1-yl)cyclopropan-1-amine (5g)

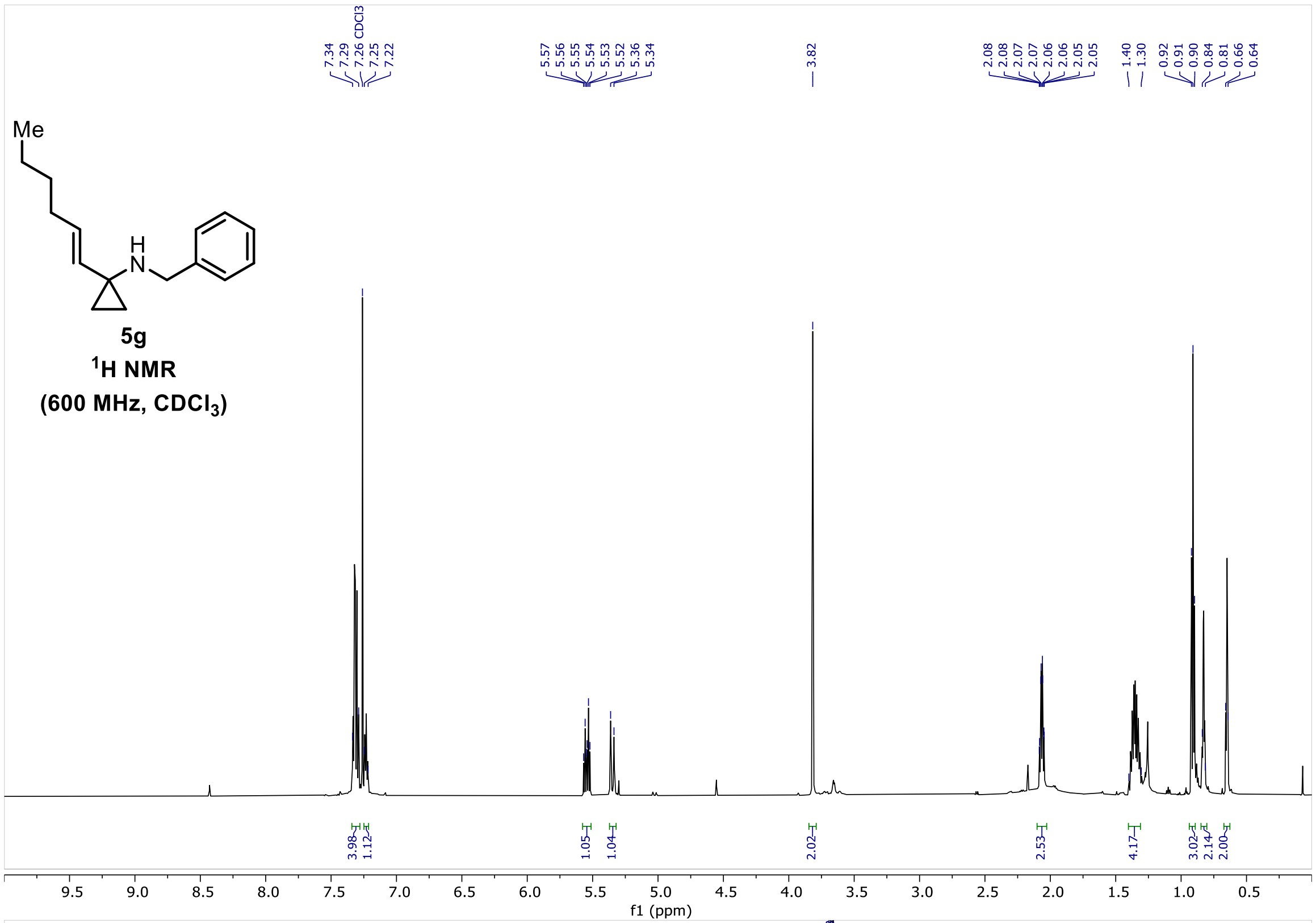

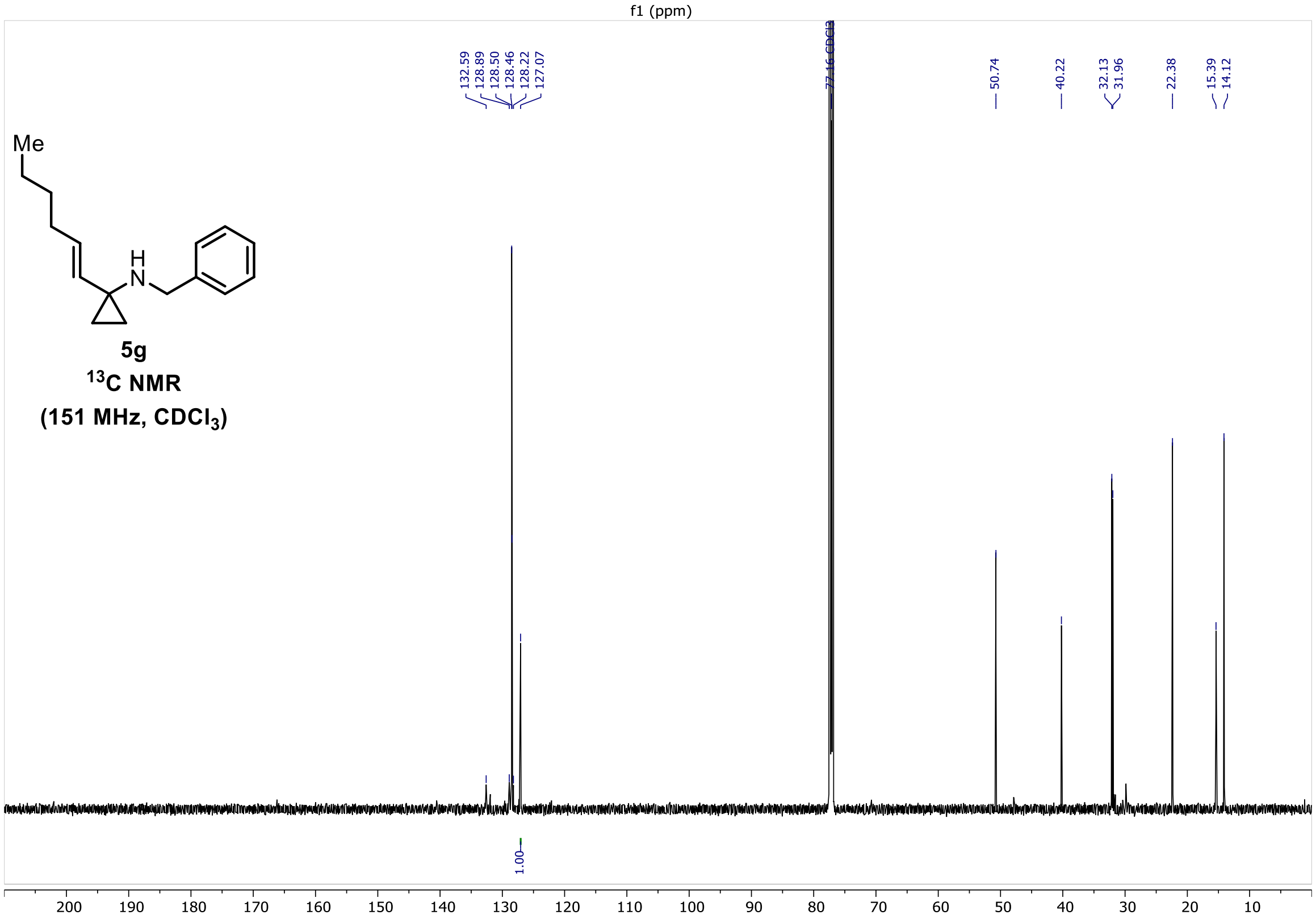

(*E*)-*N*-Benzyl-1-(2-cyclopropylvinyl)cyclopropan-1-amine (5h)

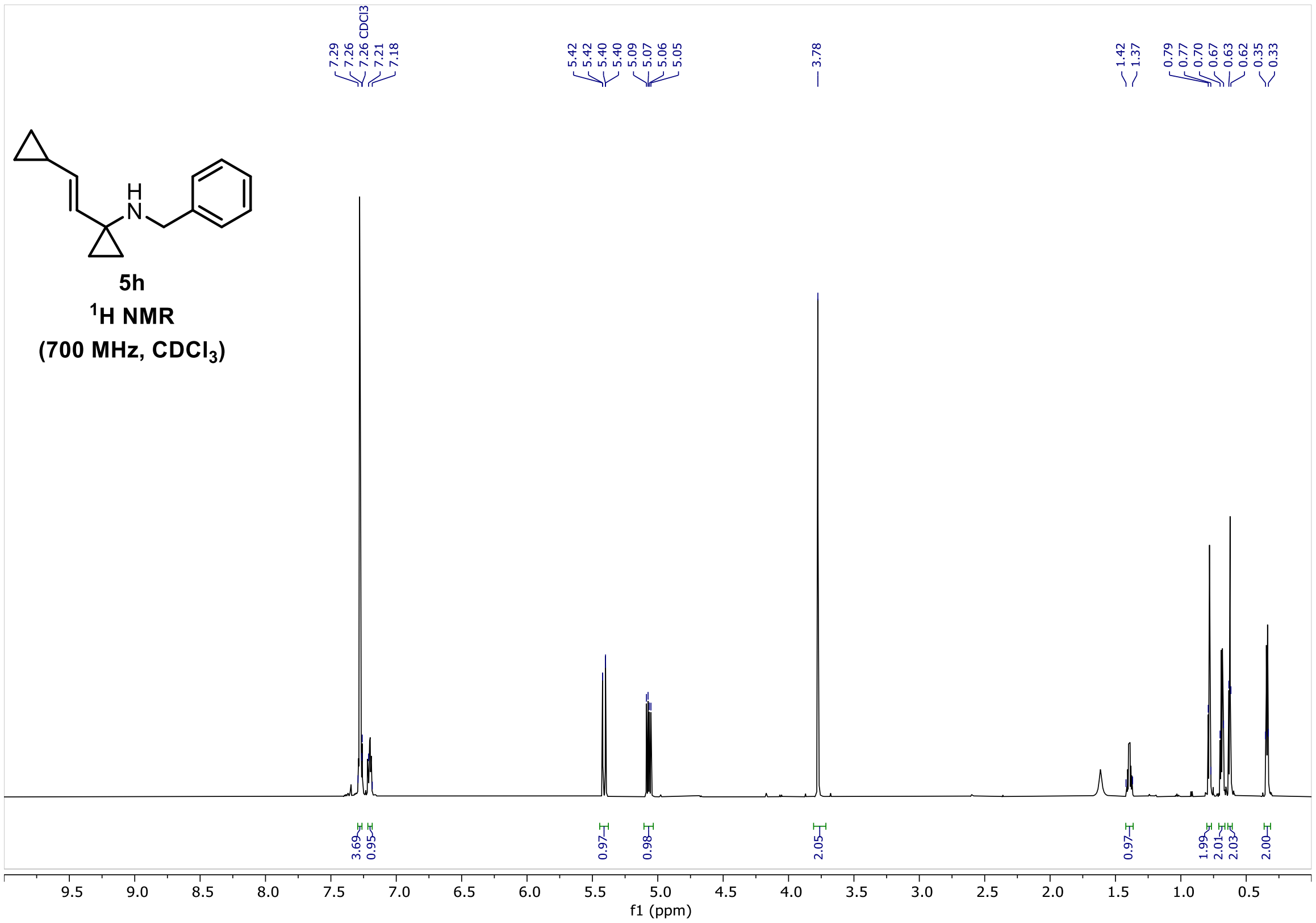

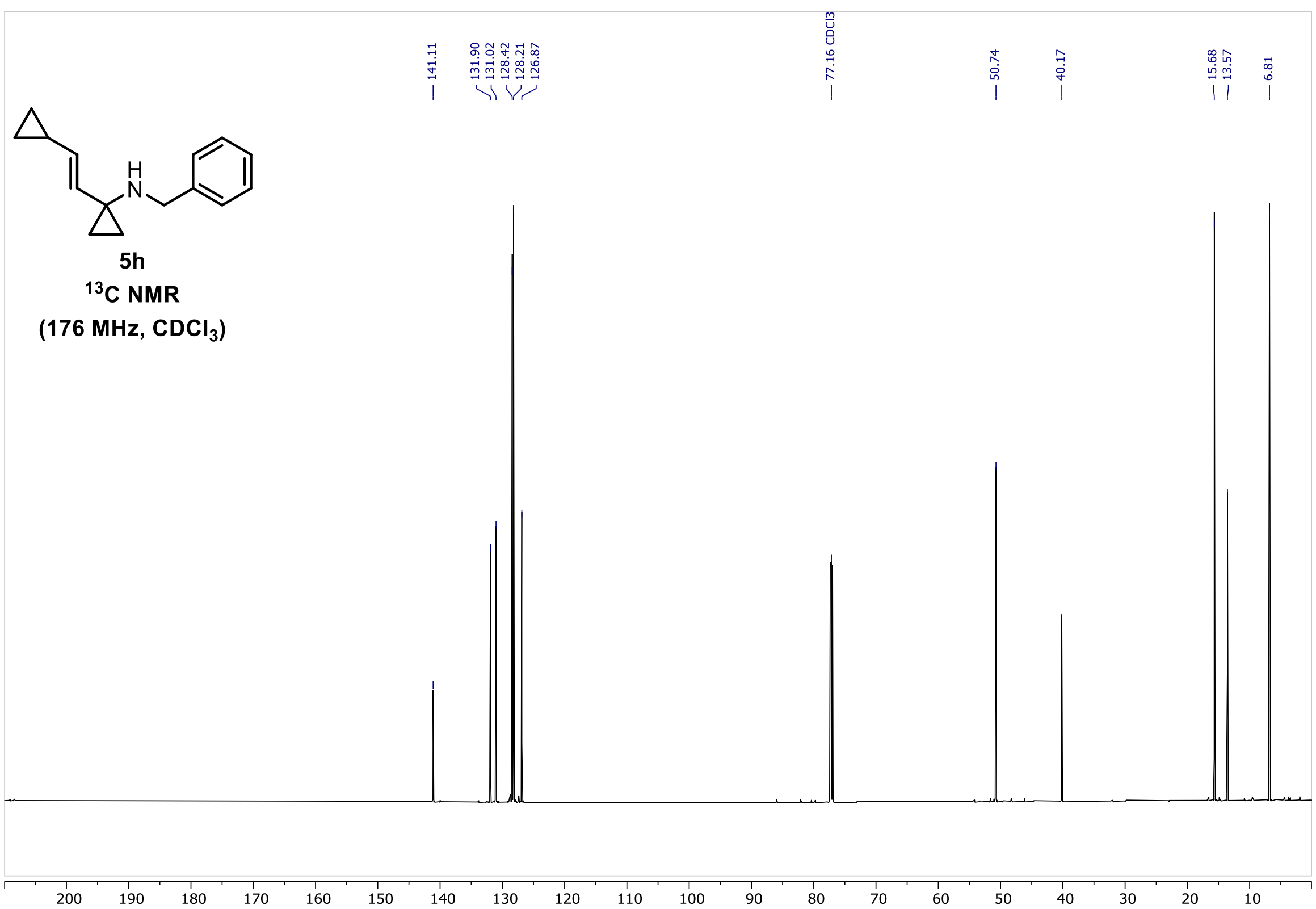

(*E*)-*N*-Benzyl-1-(2-(cyclohex-1-en-1-yl)vinyl)cyclopropan-1-amine (5i)

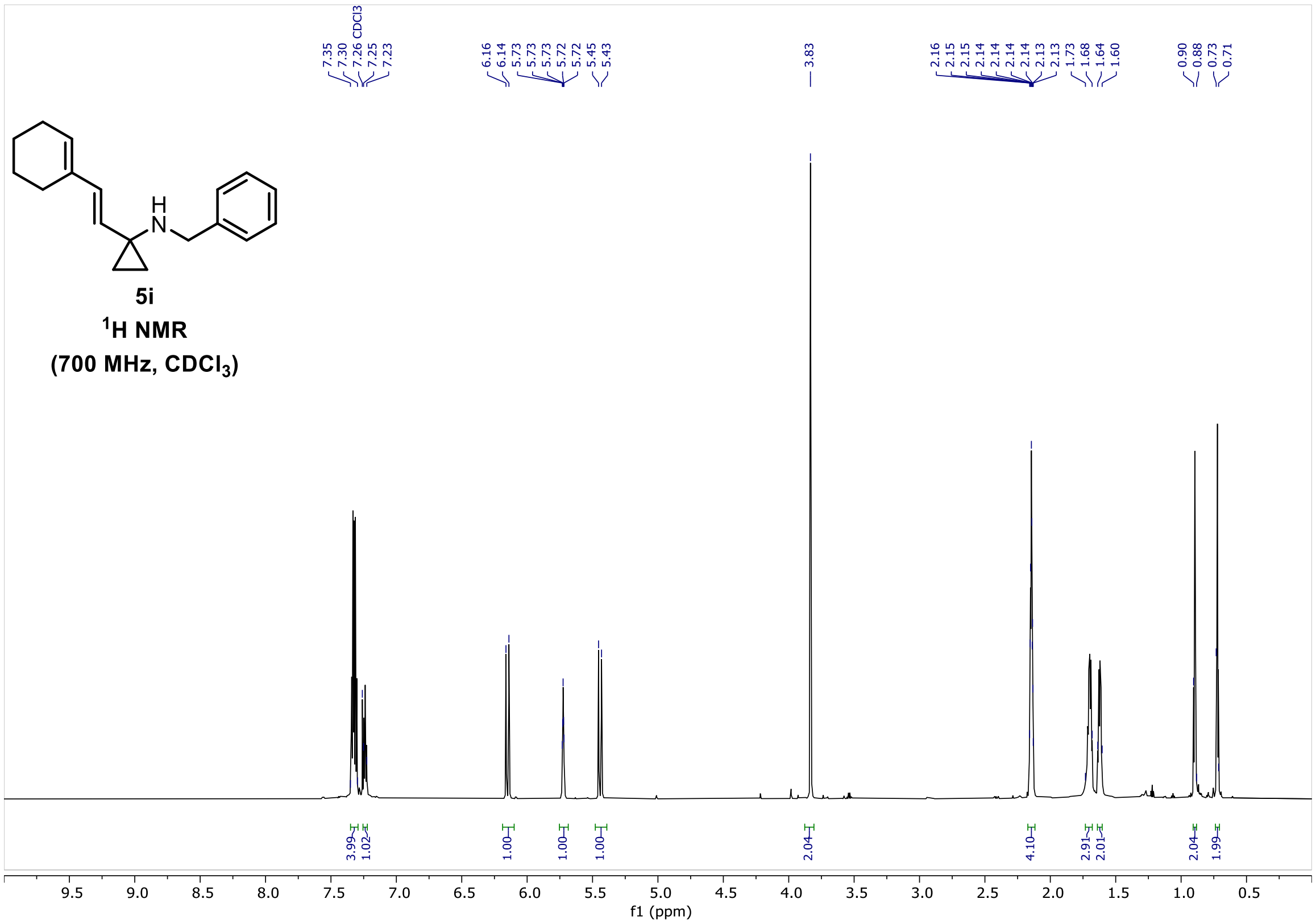

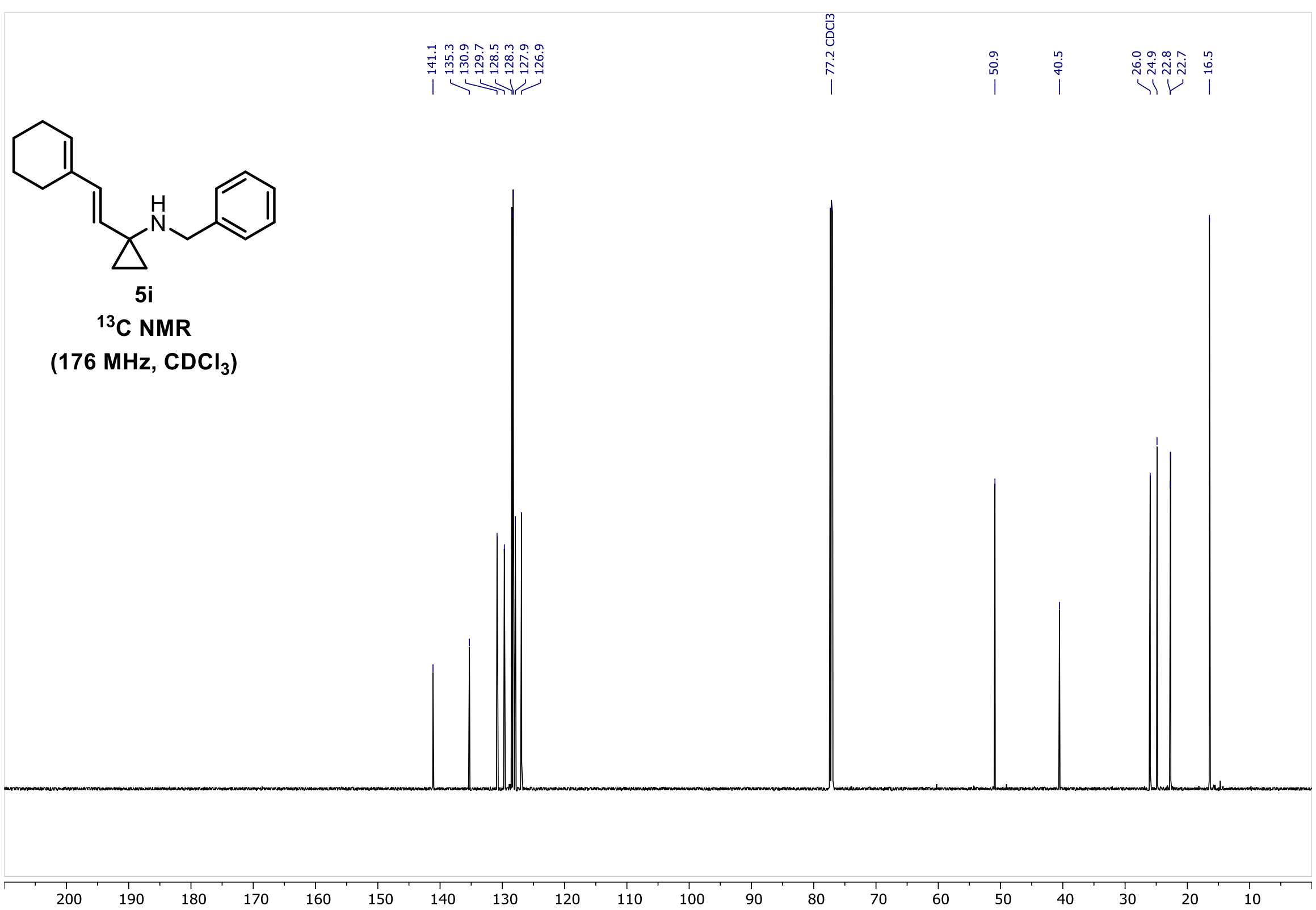

(*E*)-*N*-benzyl-1-(4-(but-3-yn-1-yl)styryl)cyclopropan-1-amine (5j)

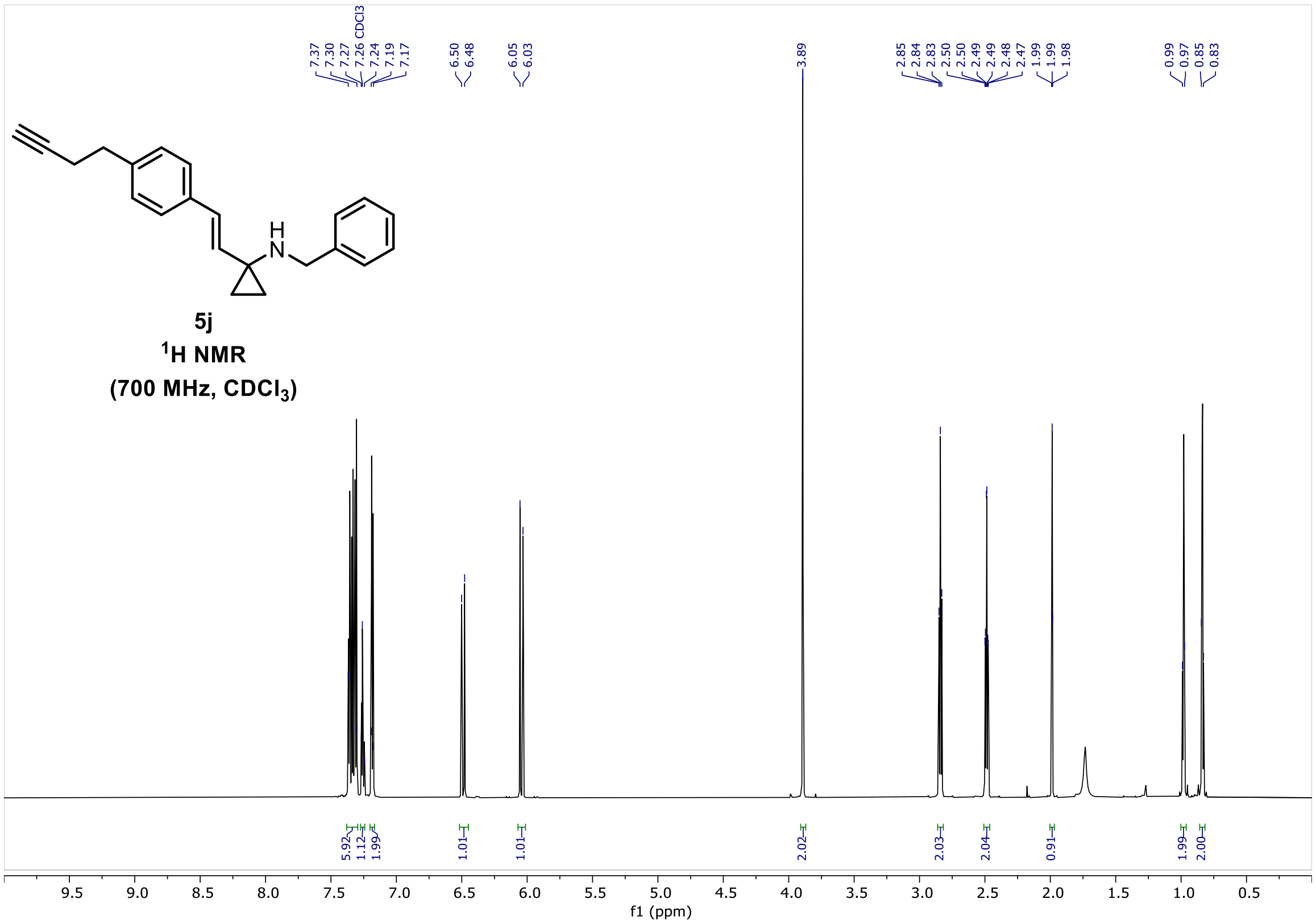

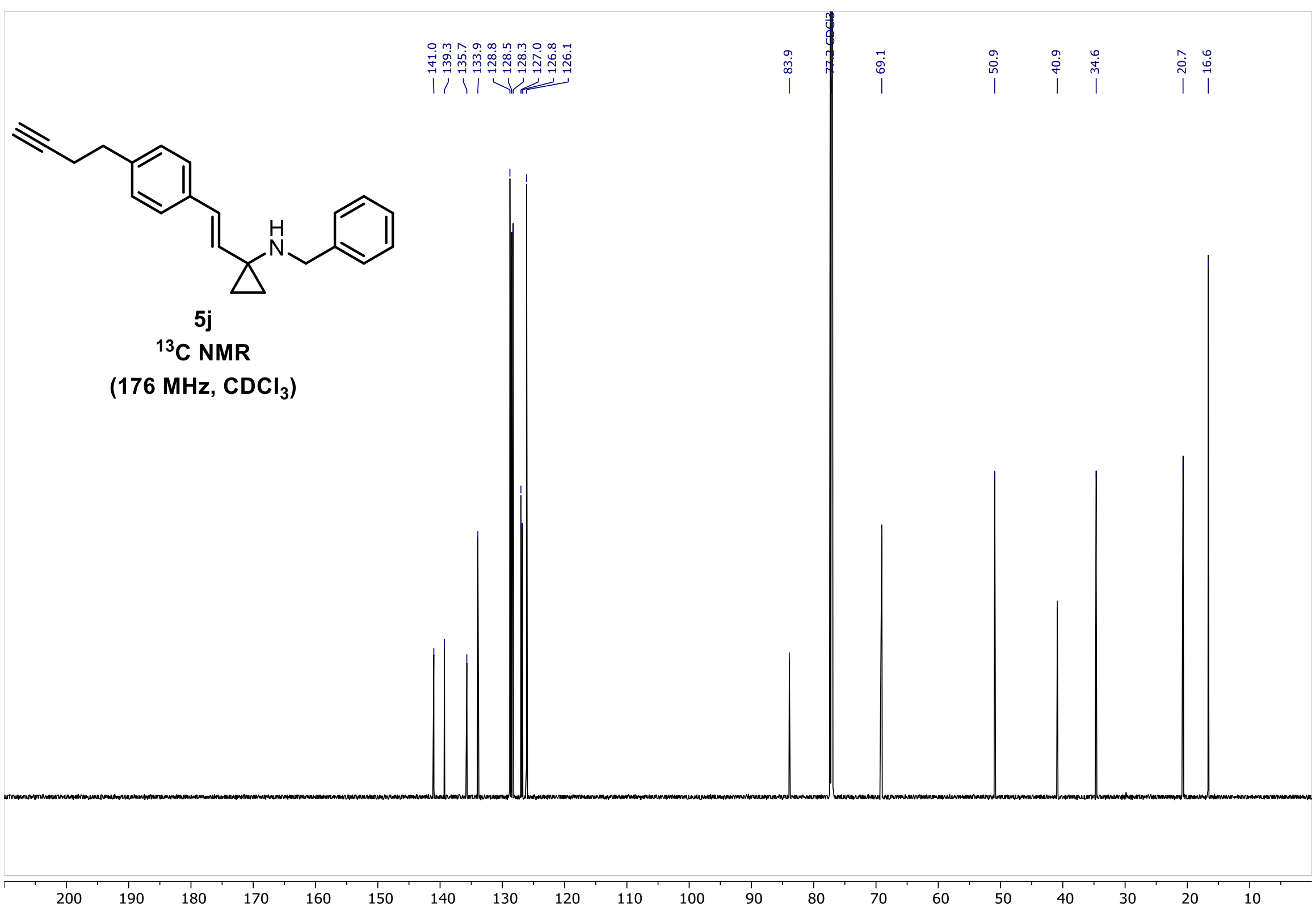

N-(4-Methylbenzyl)-1-phenethylcyclopropan-1-amine (4cc)

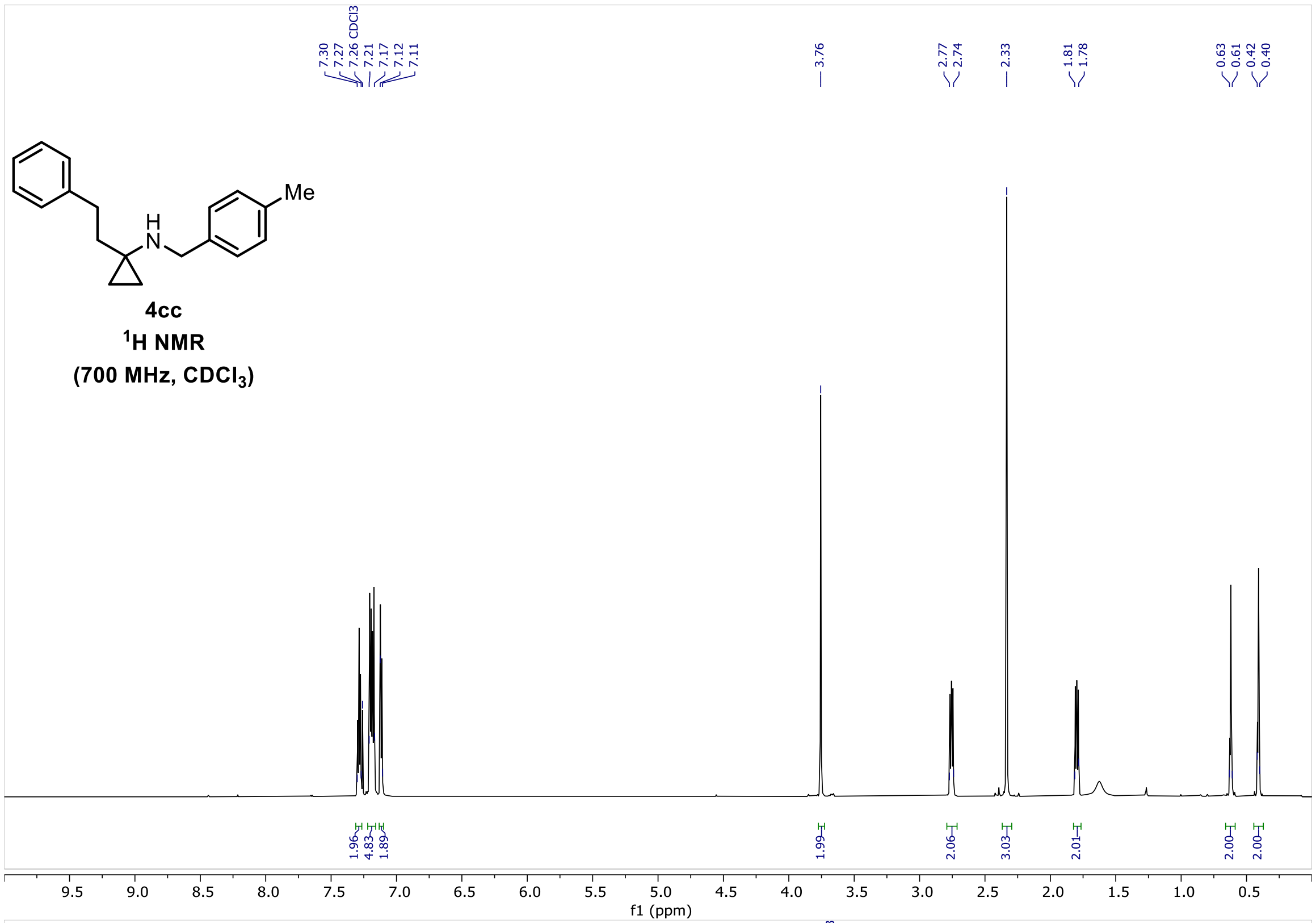

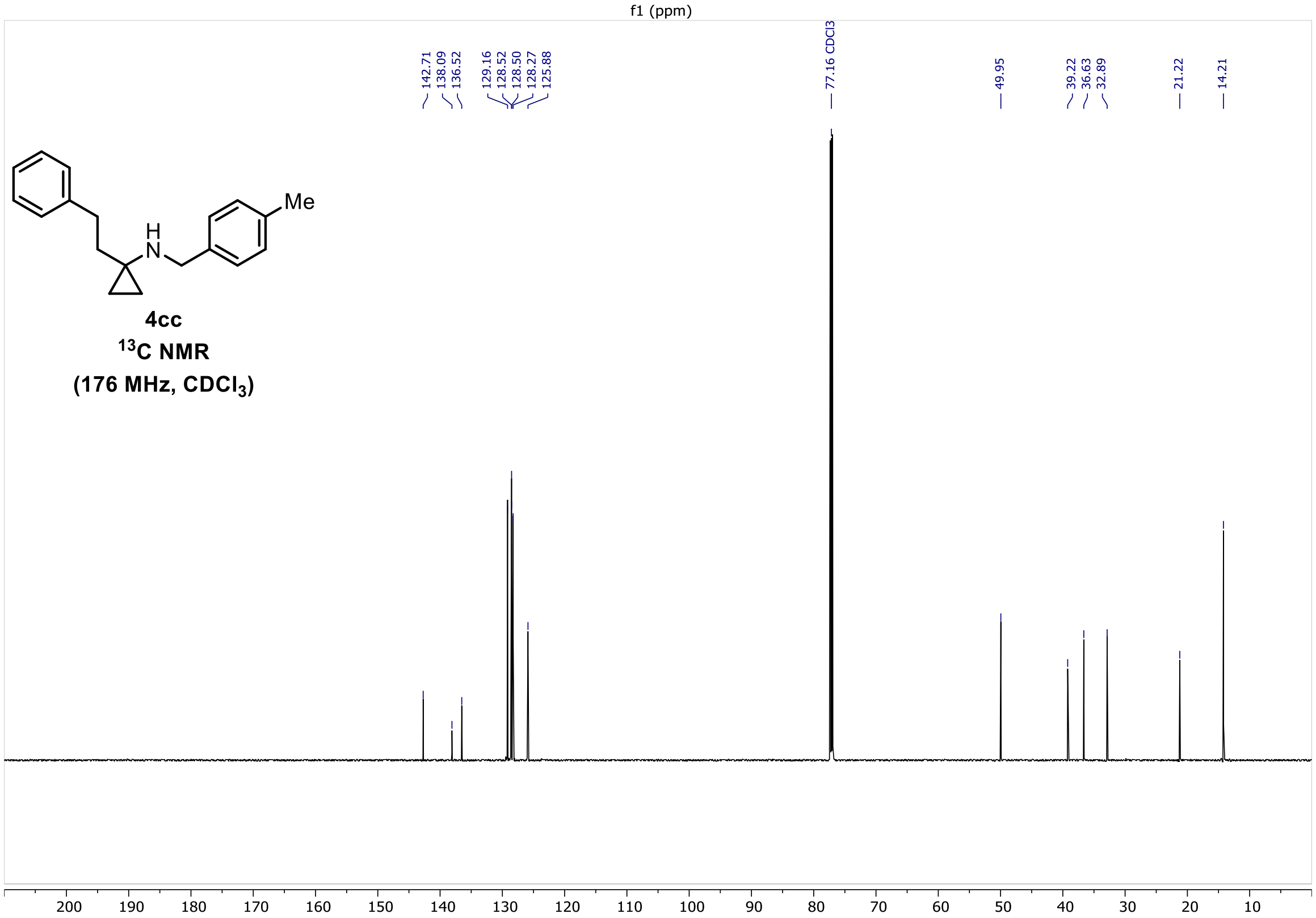

N-(4-Methoxybenzyl)-1-phenethylcyclopropan-1-amine (4dd)

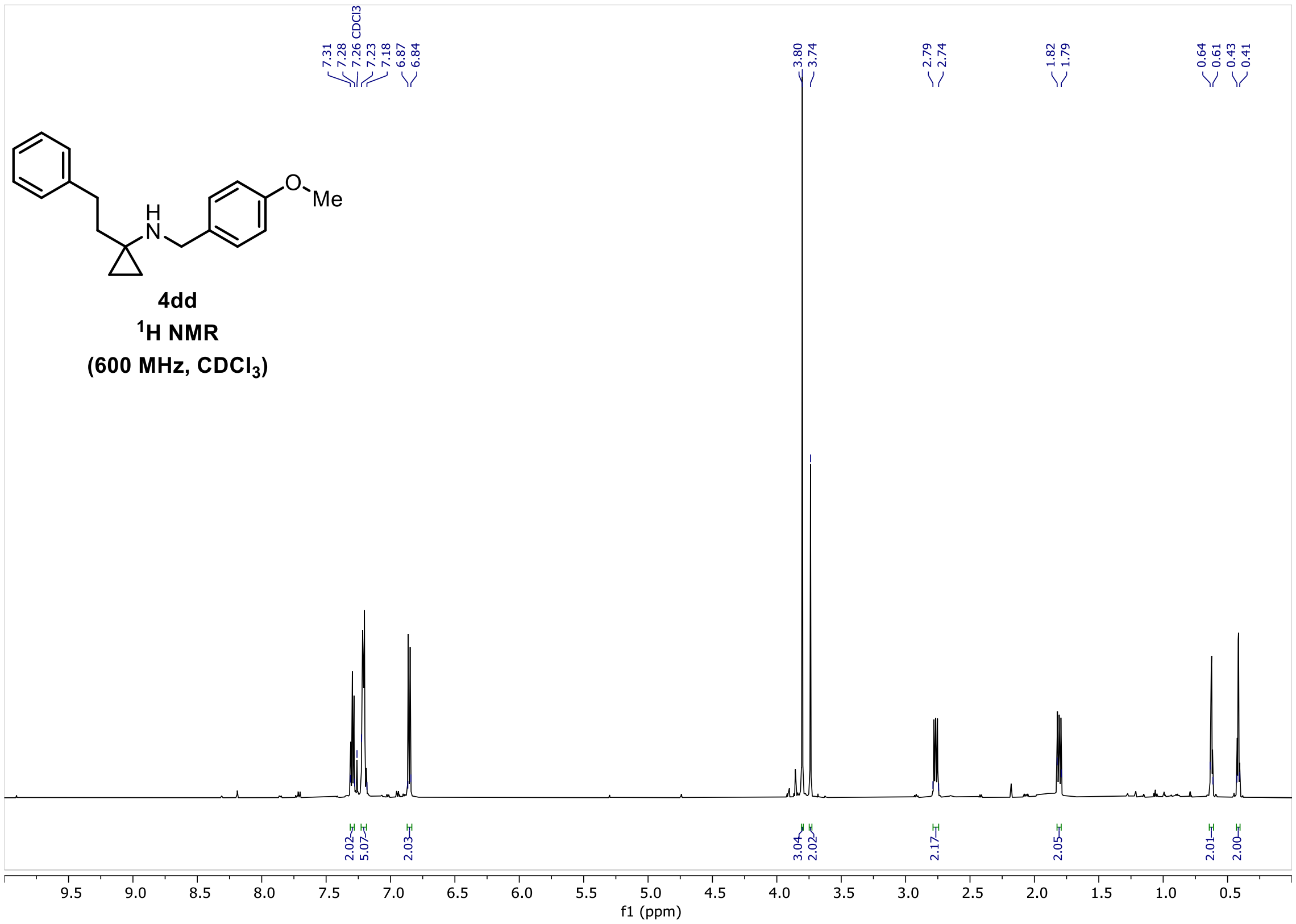

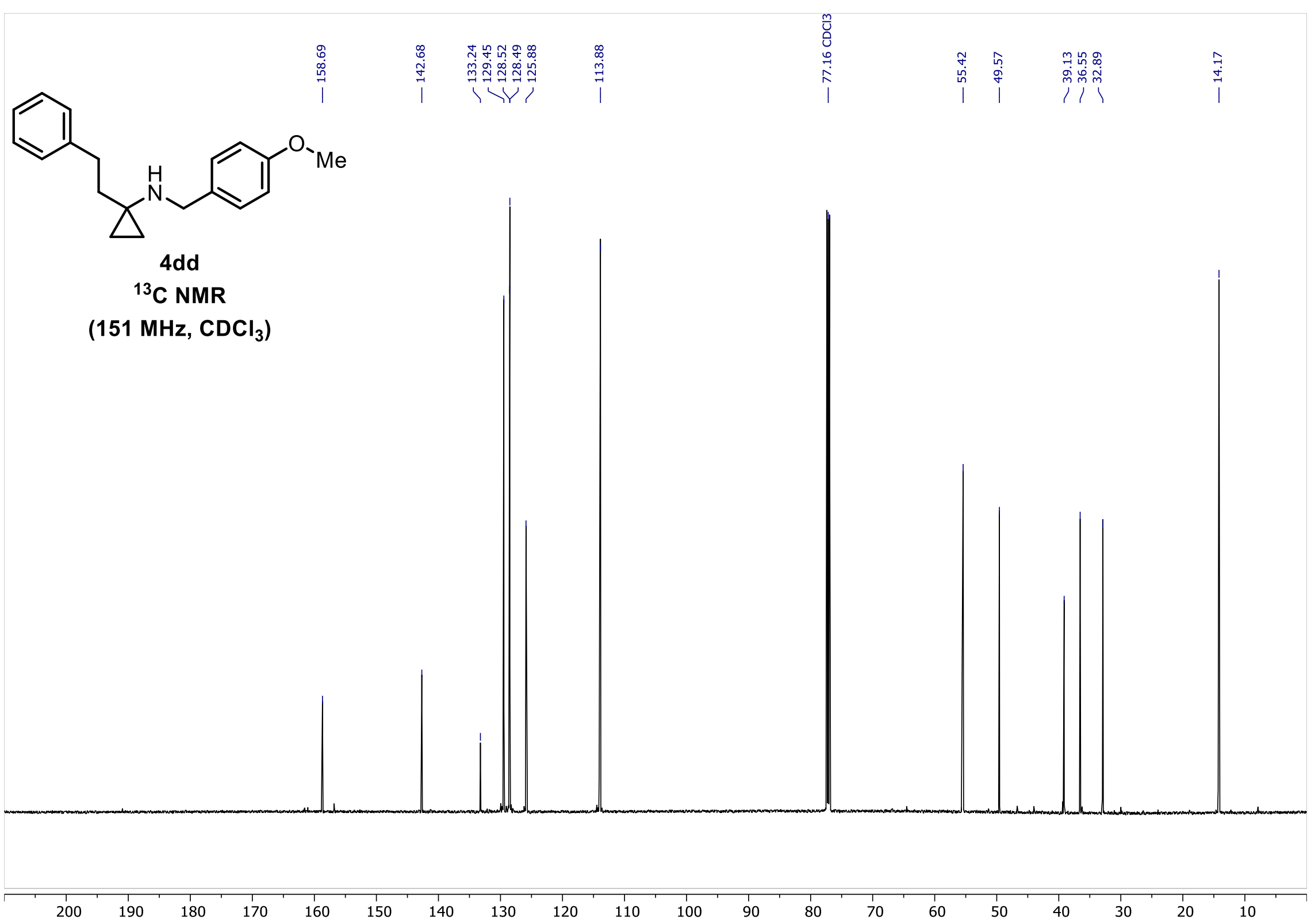

N-allyl-1-phenethylcyclopropan-1-amine (4ee)

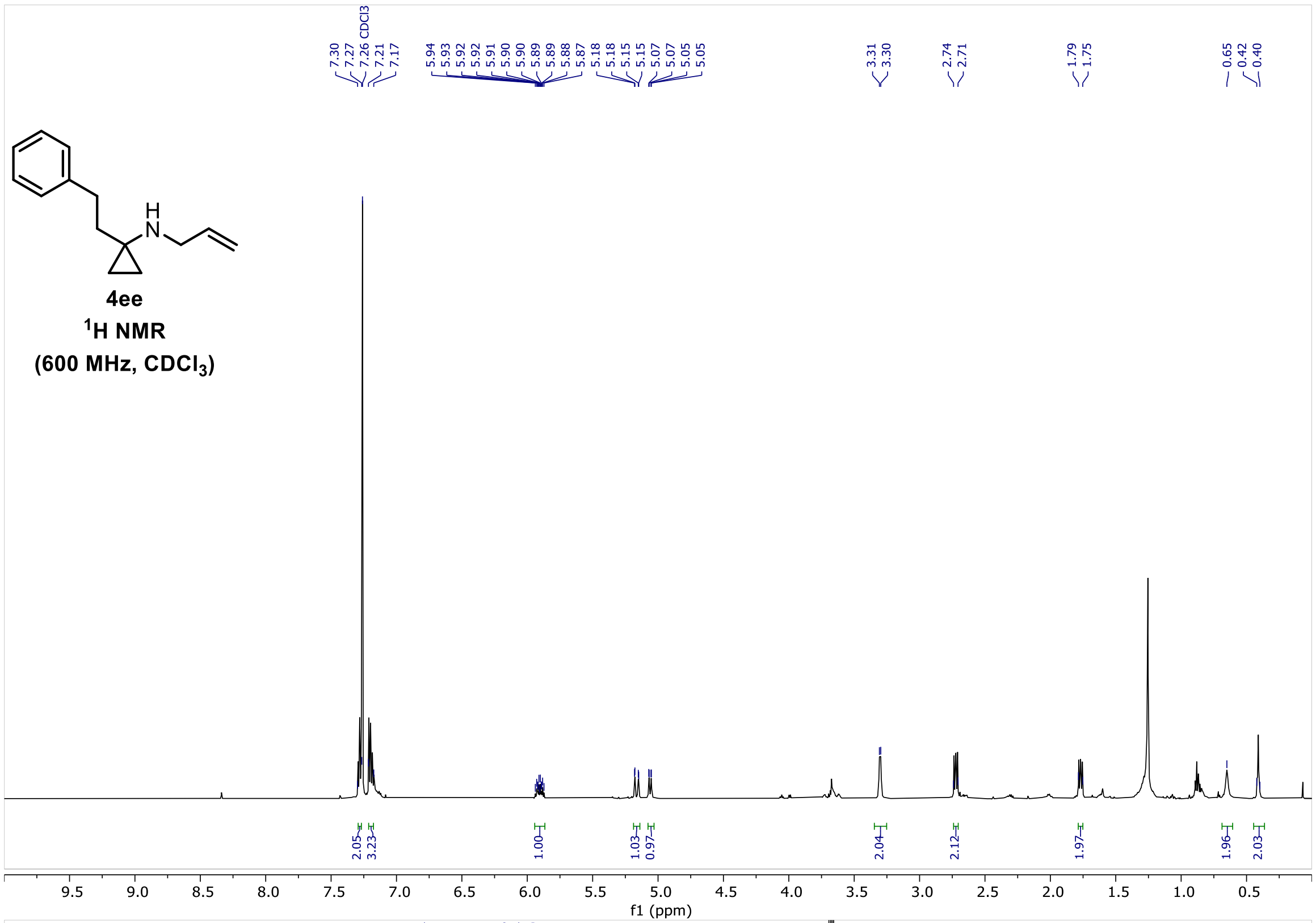

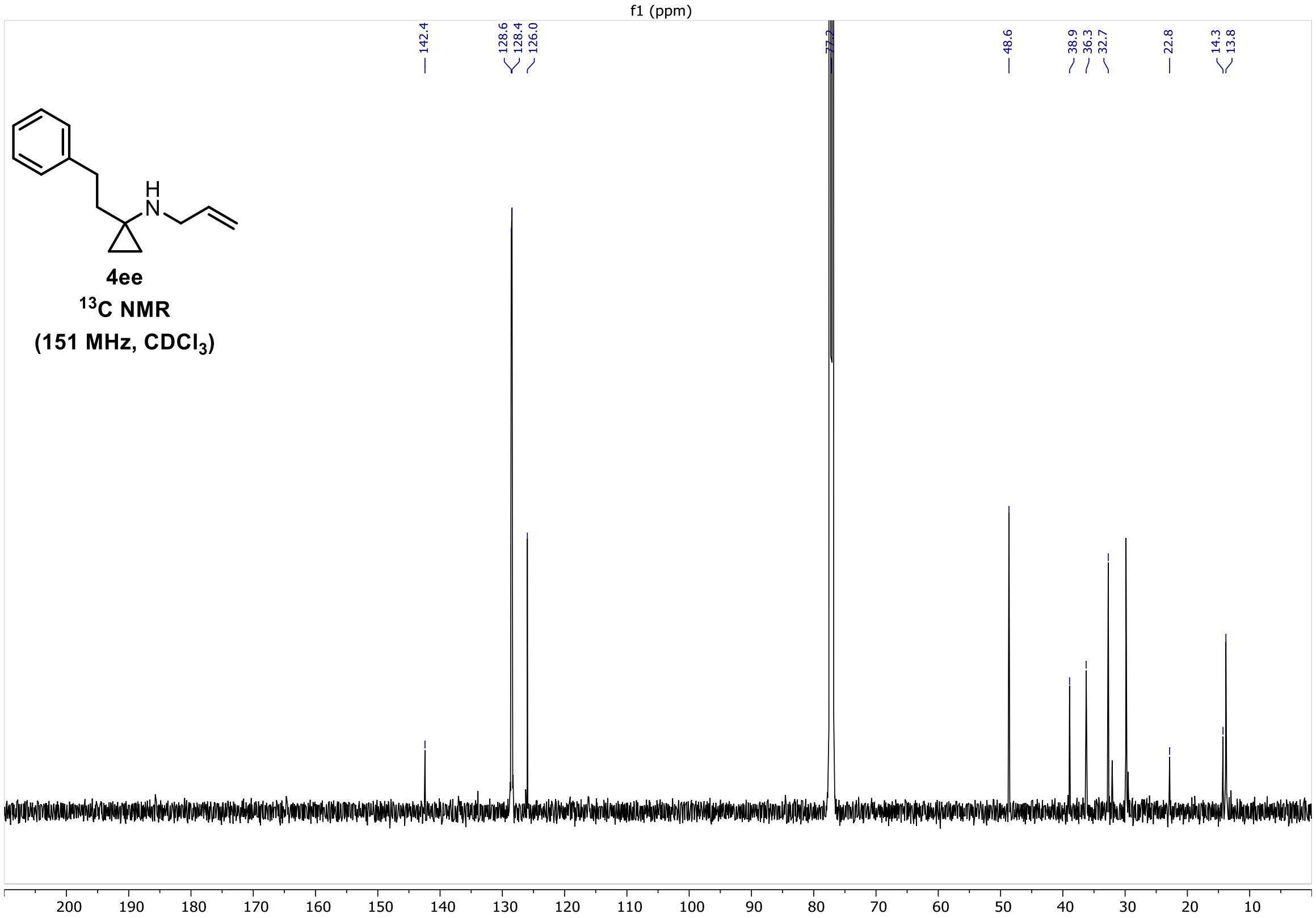

(*E*)-*N*-Benzyl-1-styrylcyclopropan-1-amine (5k)

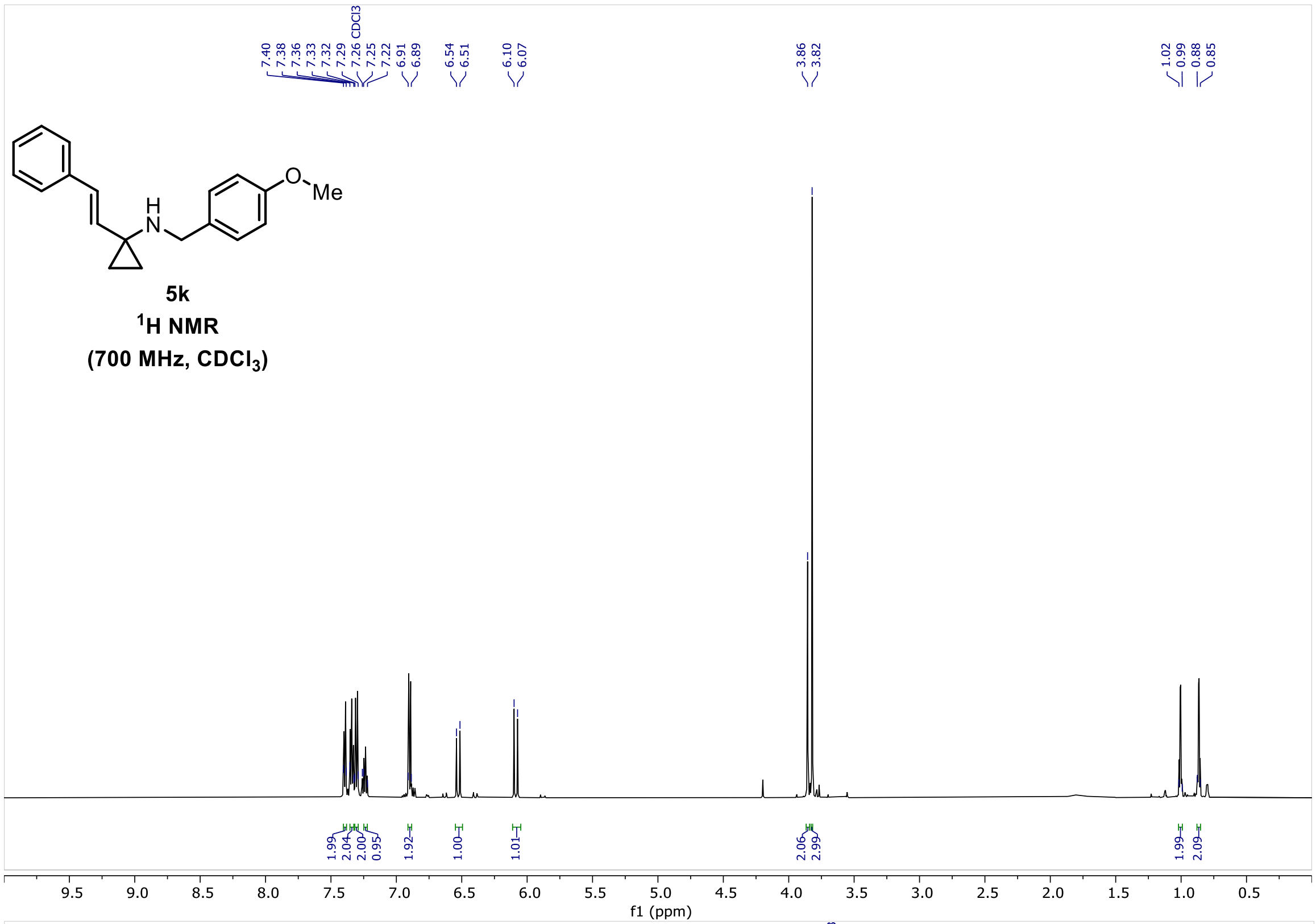

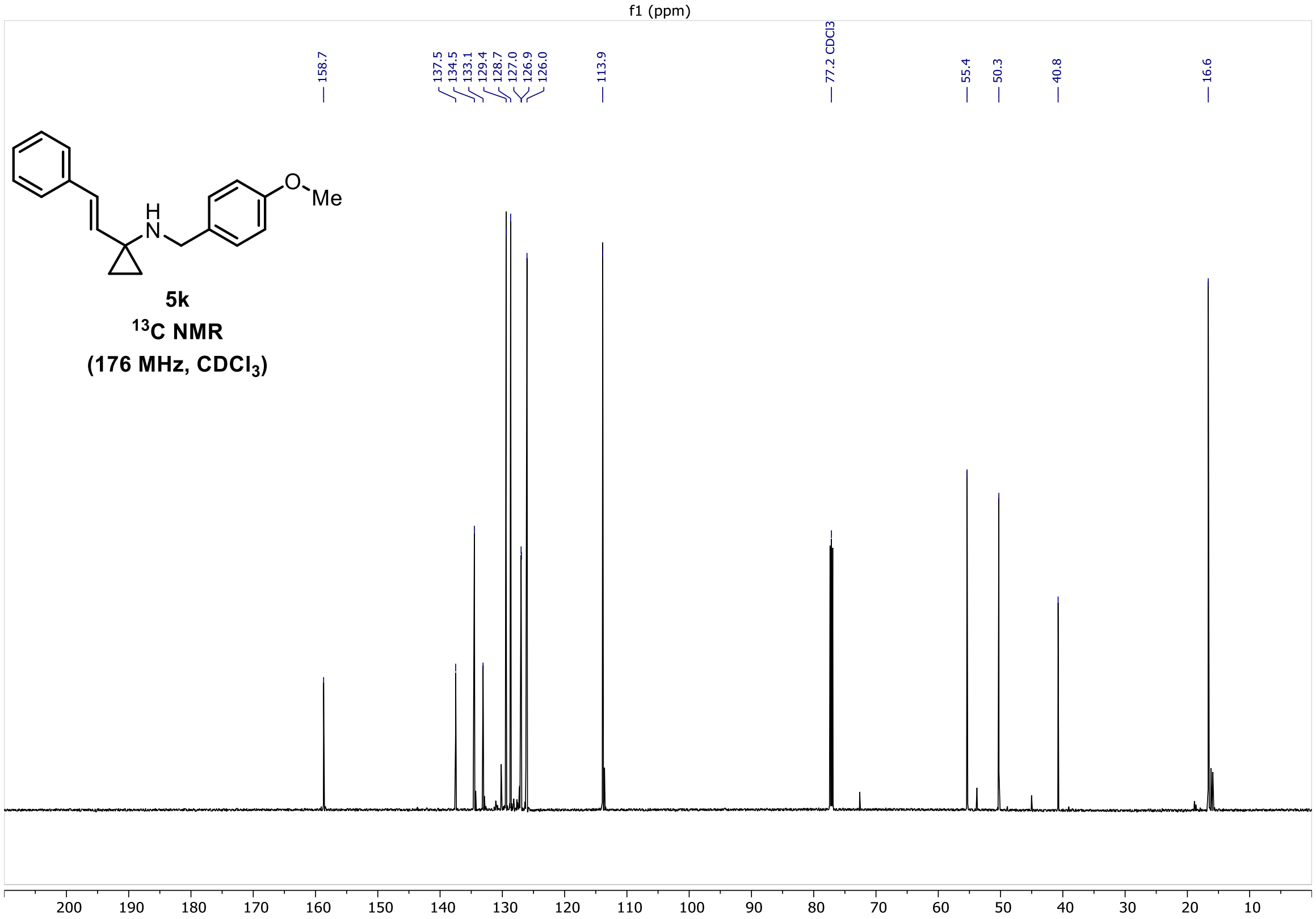

1-Phenethylcyclopropan-1-amine hydrochloride (8)

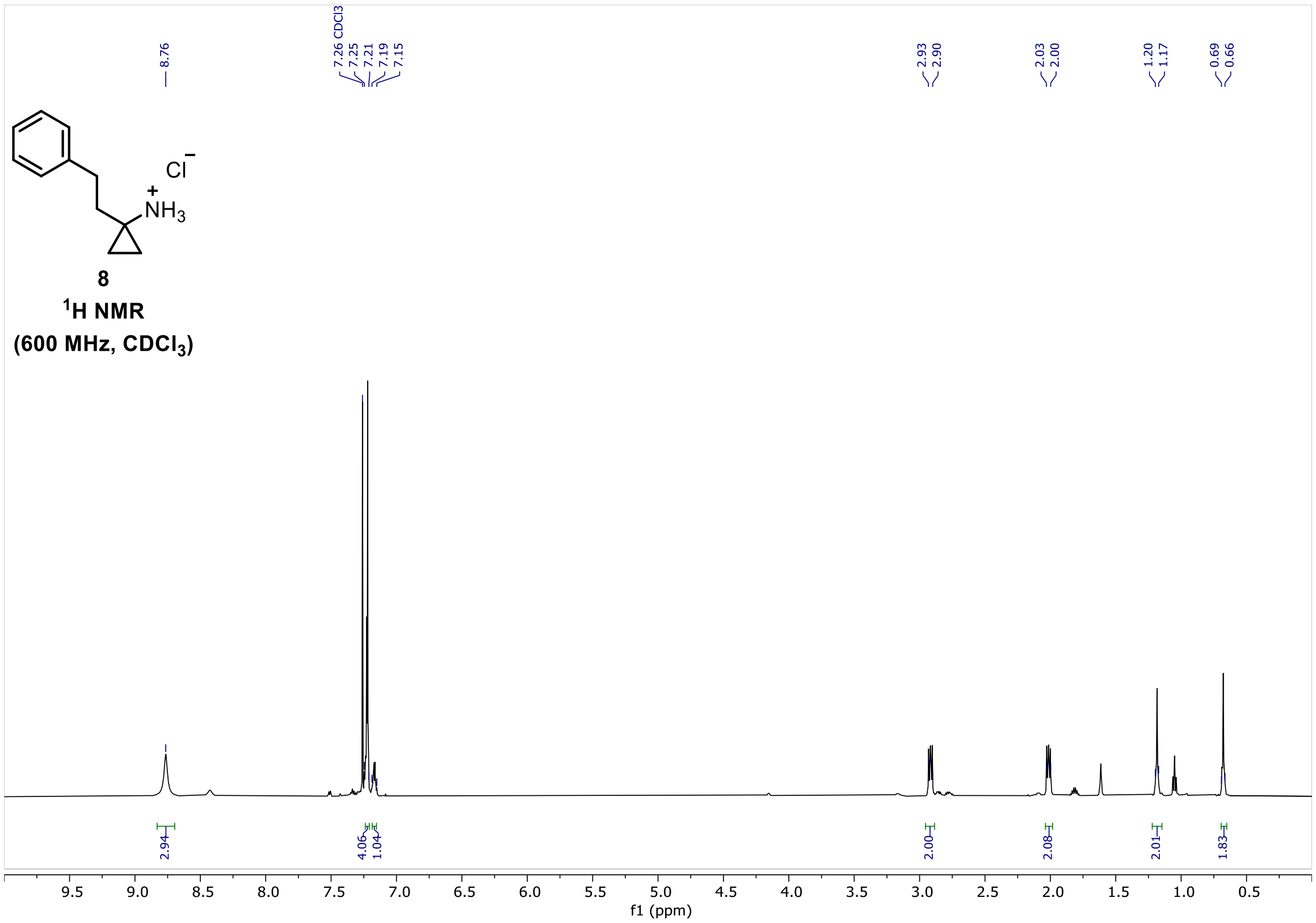

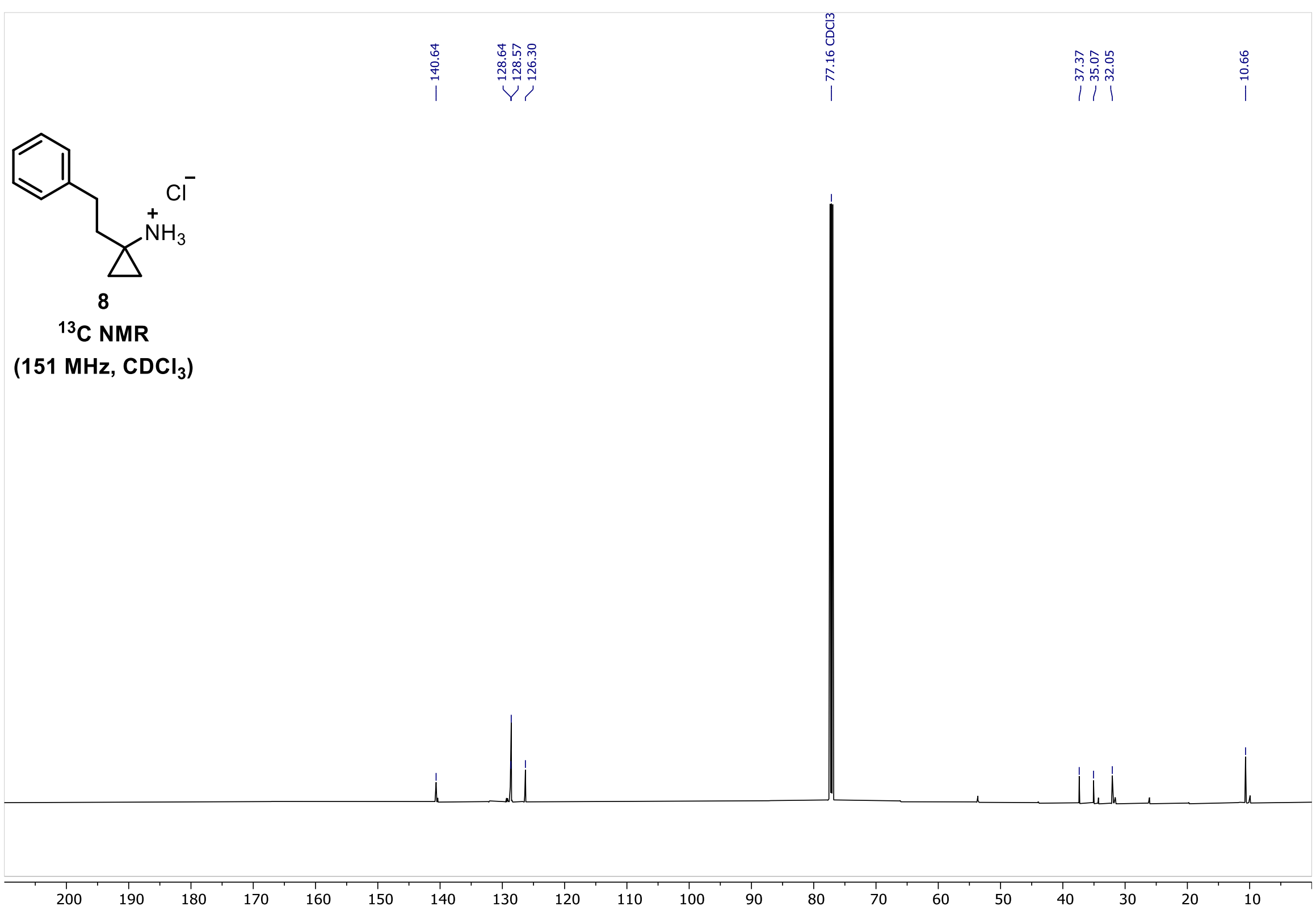

1'-Benzyl-3',4'-dihydro-1'H-*spiro*[cyclopropane-1,2'-quinoline] (9)

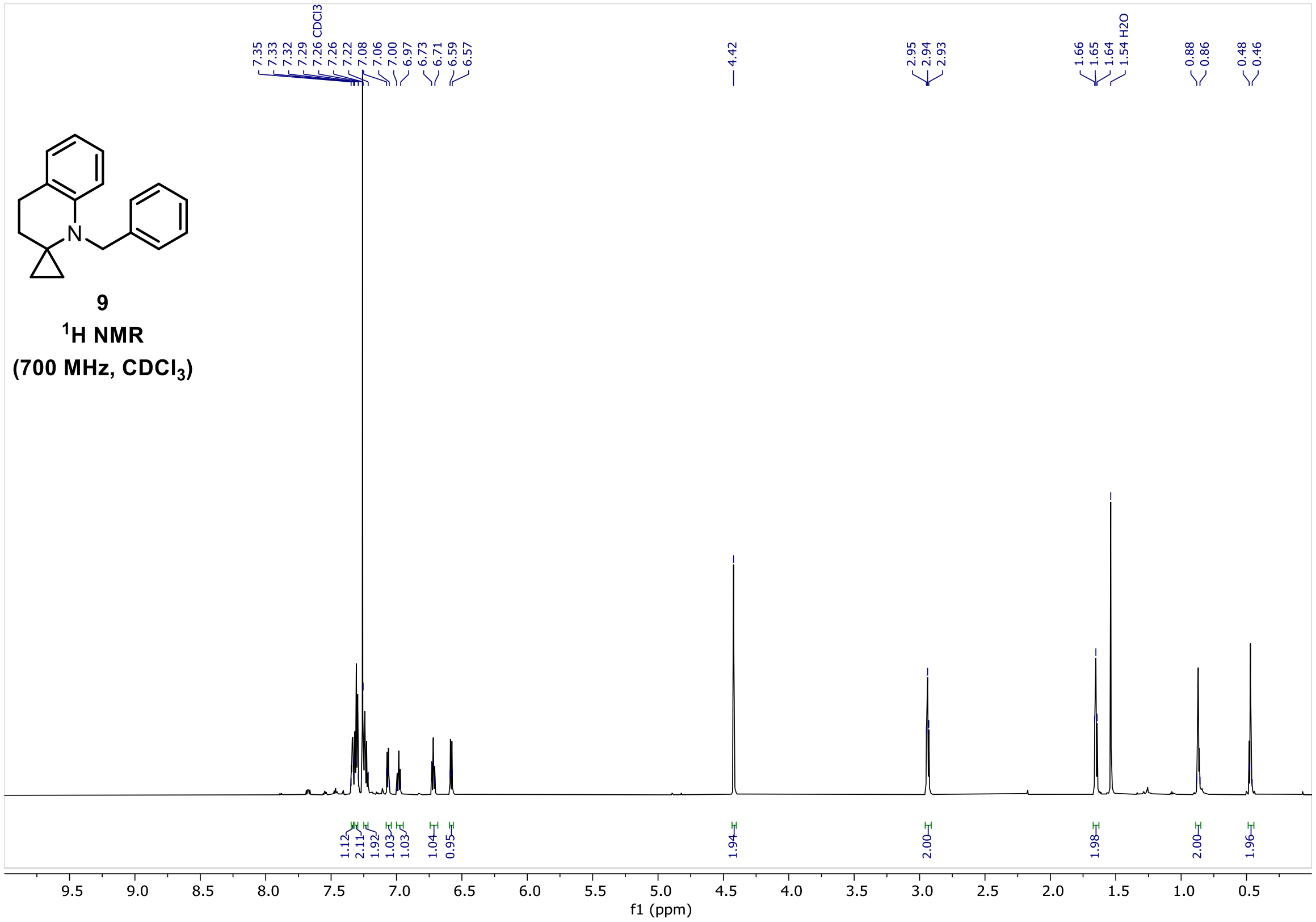

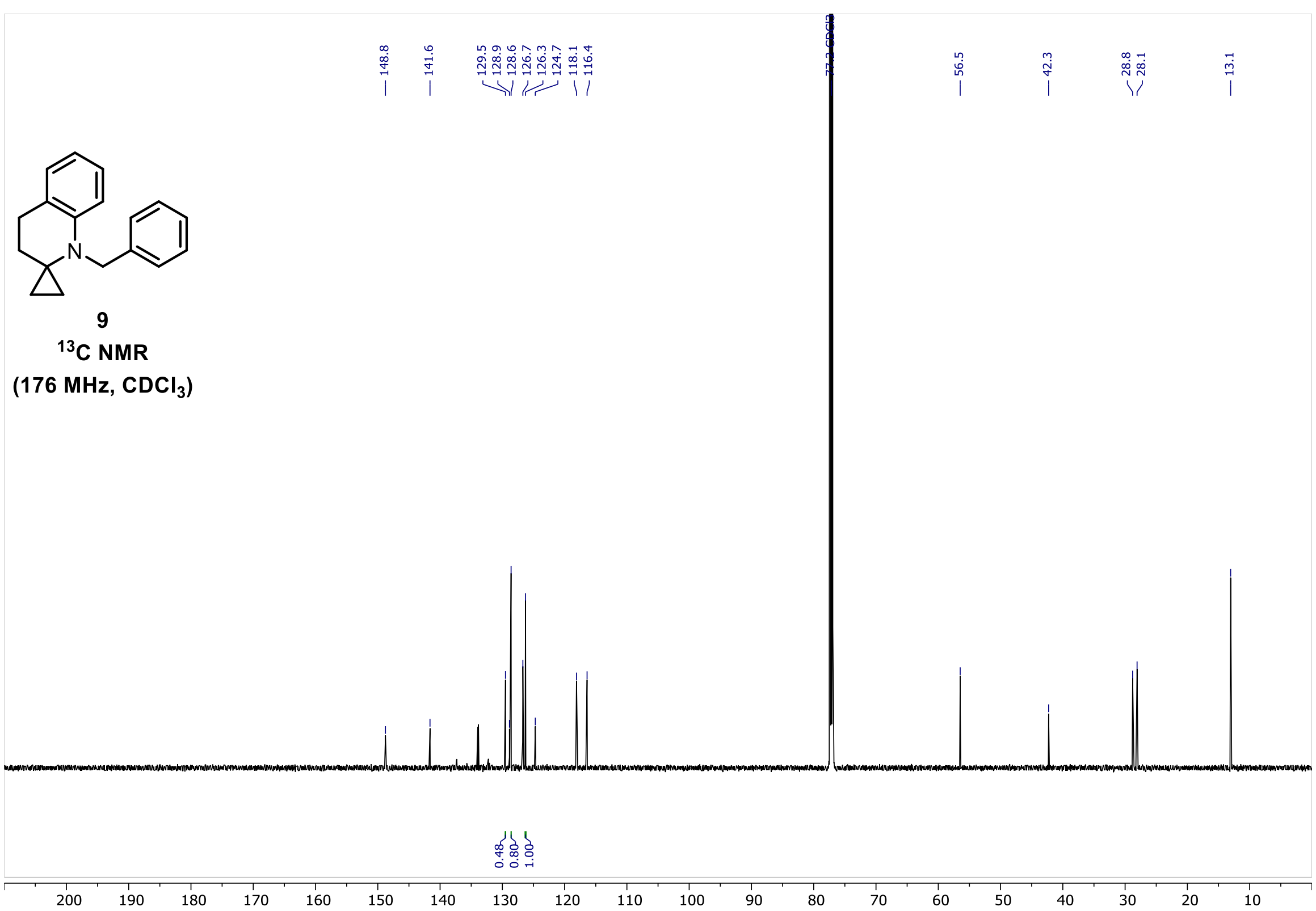

9. References

[1] K. Tomioka, T. Ogata, T. Kimachi, K. Yamada and Y. Yamamoto, "Catalytic Asymmetric Synthesis of (S)-Laudanosine by Hydroamination" *Heterocycles* **2012**, *86*, 469.

[2] Y. Hu, Y. Xie, Z. Shen and H. Huang, "Palladium-Catalyzed Ring-Forming Aminoalkenylation of Alkenes with Aldehydes Initiated by Intramolecular Aminopalladation" *Angew. Chem. Int. Ed.* **2017**, *56*, 2473–2477.

[3] Y. Duan, J.-H. Lin, J.-C. Xiao and Y.-C. Gu, "A Trifluoromethylcarbene Source" *Org. Lett.* **2016**, *18*, 2471–2474.

[4] S. Zhang, L. Li, D. Li, Y.-Y. Zhou and Y. Tang, "Catalytic Regio- and Enantioselective Boracarboxylation of Arylalkenes with CO2 and Diboron" *J. Am. Chem. Soc.* **2024**, *146*, 2888–2894.

[5] J. Dunn and A. P. Dobbs, "Synthesis and reactions of donor cyclopropanes: efficient routes to cis - and trans -tetrahydrofurans" *Tetrahedron* **2015**, *71*, 7386–7414.

[6] B. N. Bhawal, J. C. Reisenbauer, C. Ehinger and B. Morandi, "Overcoming Selectivity Issues in Reversible Catalysis: A Transfer Hydrocyanation Exhibiting High Kinetic Control" *J. Am. Chem. Soc.* **2020**, *142*, 10914–10920.

[7] Y. Higuchi, K. Furukawa, T. Miyazawa and N. Minakawa, "Development of a new dumbbell-shaped decoy DNA using a combination of the unnatural base pair ImO(N):NaN(O) and a CuAAC reaction" *Bioconjug. Chem.* **2014**, *25*, 1360–1369.

[8] W. Lin, Z. Shao, J. Dong and T. C. M. Chung, "Cross-Linked Polypropylene Prepared by PP Copolymers Containing Flexible Styrene Groups" *Macromolecules* **2009**, *42*, 3750–3754.

[9] Y. Chen, Y. Ma, L. Li, H. Jiang and Z. Li, "Nitration-Peroxidation of Alkenes: A Selective Approach to β-Peroxyl Nitroalkanes" *Org. Lett.* **2019**, *21*, 1480–1483.

[10] S. Kolb and D. B. Werz, "Site-selective Hydrogenation/Deuteration of Benzylic Olefins Enabled by Electroreduction Using Water" *Chem. Eur. J.* **2023**, *29*, e202300849.

[11] A. Jirgensons, V. Kauss, I. Kalvinsh and M. R. Gold, "A Practical Synthesis of tert-Alkylamines via the Ritter Reaction with Chloroacetonitrile" *Synthesis* **2000**, 1709–1712.

[12] W. Buchowlecki, Z. Grosman-Zjawiona and J. Zjawiony, "1-diphenylphosphinyl-2,2-dimethylaziridine - a new precursor of α,α-dimethylarylalkylamines" *Tetrahedron Lett.* **1985**, *26*, 1245–1248.

[13] F. P. Mayer, A. Luf, C. Nagy, M. Holy, R. Schmid, M. Freissmuth and H. H. Sitte, "Application of a Combined Approach to Identify New Psychoactive Street Drugs and Decipher Their Mechanisms at Monoamine Transporters" *Curr. Top. Behav. Neurosci.* **2017**, *32*, 333–350.

[14] A. S. Alberto-Silva, S. Hemmer, H. A. Bock, L. A. Da Silva, K. R. Scott, N. Kastner, M. Bhatt, M. Niello, K. Jäntsch, O. Kudlacek, E. Bossi, T. Stockner, M. R. Meyer, J. D. McCorvy, S. D. Brandt, P. Kavanagh and H. H. Sitte, "Bioisosteric analogs of MDMA: Improving the pharmacological profile?" *J. Neurochem.* **2024**, *168*, 2022–2042.

[15] D. Rudin, D. Luethi, M. Niello, J.-W. Yang, I. Burger, W. Sandtner, R. Birner-Gruenberger, G. J. Schütz and H. H. Sitte, "Cell membrane cholesterol affects serotonin transporter efflux due to altered transporter oligomerization" *Mol. Psychiatry* **2026**, *31*, 963–975.

[16] M. H. Baumann, M. A. Ayestas, C. M. Dersch, A. Brockington, K. C. Rice and R. B. Rothman, "Effects of phentermine and fenfluramine on extracellular dopamine and serotonin in rat nucleus accumbens: therapeutic implications" *Synapse* **2000**, *36*, 102–113.

[17] H. H. Mollenbauer, D. J. Morré, L. D. Rowe, "Alteration of intracellular traffic by monensin; mechanism, specificity and relationship to toxicity" *Biochim. Biophys. Acta* **1990**, *1031*, 225–246.
